# Anti-miR-17 therapy delays tumorigenesis in MYC-driven hepatocellular carcinoma (HCC)

**DOI:** 10.18632/oncotarget.22342

**Published:** 2017-11-09

**Authors:** Renumathy Dhanasekaran, Meital Gabay-Ryan, Virginie Baylot, Ian Lai, Adriane Mosley, Xinqiang Huang, Sonya Zabludoff, Jian Li, Vivek Kaimal, Priya Karmali, Dean W. Felsher

**Affiliations:** ^1^ Division of Gastroenterology and Hepatology, Department of Medicine, Stanford University School of Medicine, Stanford, CA, USA; ^2^ Division of Oncology, Department of Medicine, Stanford University School of Medicine, Stanford, CA, USA; ^3^ Division of Oncology, Department Pathology, Stanford University School of Medicine, Stanford, CA, USA; ^4^ Regulus Therapeutics, San Diego, CA, USA

**Keywords:** liver cancer, HCC, miR, MYC, lipid nanoparticle (LNP)

## Abstract

Hepatocellular carcinoma (HCC) remains a significant clinical challenge with few therapeutic options. Genomic amplification and/or overexpression of the MYC oncogene is a common molecular event in HCC, thus making it an attractive target for drug therapy. Unfortunately, currently there are no direct drug therapies against MYC. As an alternative strategy, microRNAs regulated by MYC may be downstream targets for therapeutic blockade. MiR-17 family is a microRNA family transcriptionally regulated by MYC and it is commonly overexpressed in human HCCs. In this study, we performed systemic delivery of a novel lipid nanoparticle (LNP) encapsulating an anti-miR-17 oligonucleotide in a conditional transgenic mouse model of MYC driven HCC. Treatment with anti-miR-17 *in vivo*, but not with a control anti-miRNA, resulted in significant de-repression of direct targets of miR-17, robust apoptosis, decreased proliferation and led to delayed tumorigenesis in MYC-driven HCCs. Global gene expression profiling revealed engagement of miR-17 target genes and inhibition of key transcriptional programs of MYC, including cell cycle progression and proliferation. Hence, anti-miR-17 is an effective therapy for MYC-driven HCC.

## INTRODUCTION

Hepatocellular carcinoma (HCC) is a generally lethal cancer with increasing frequency in the Unites States and world-wide [1]. Conventional chemotherapy has limited efficacy. Targeted therapies like Sorafenib or Regorafenib improve life expectancy only by a few months [2, 3] Several other drugs have failed in phase 3 clinical trials for HCC likely due to the failure to identify subpopulations amenable to targeted therapy [4]. Therefore, it is imperative to develop novel effective therapeutics and biomarker stratification strategies for selecting the right drug for the right patient.

MYC genomic amplification and/or overexpression is a common molecular event in HCC [5]. MYC is a transcription factor that modulates the gene expression of thousands of genes that regulate many programs which are hallmarks of cancer including: metabolism, proliferation, self-renewal, and survival [6, 7]. Experimentally, inactivation of MYC oncogene in HCC is sufficient to lead to tumor regression associated with proliferative arrest, differentiation, and apoptosis [8]. Hence, therapies against MYC have the potential to be highly effective treatment for liver cancer. However, no existing therapies directly inactivate the MYC oncogene, so identifying critical downstream gene products that are essential for MYC to maintain a neoplastic state can help us indirectly target MYC.

Recently, we reported that MYC’s ability to maintain proliferation, survival and self-renewal were regulated via its induction of miR17∼92 cluster [9]. The miR 17 family (miR 17, miR 20a, miR 20b, miR106a, miR106b, miR 93) is a part of this cluster and few studies have shown that over-expression of miR-17 family promotes HCC progression and cancer metastasis [10, 11]. We therefore hypothesized that miR-17 is a promising therapeutic target for MYC-driven HCCs.

Targeting microRNAs has been considered a desired approach in cancer therapeutics [12]. However, several challenges exist in achieving drug stability and ensuring tissue-specific drug delivery [13, 14]. Recently, a novel approach for targeting miR-17 with a tough decoy (TuD) antisense miR17 delivered via systemic lipid nanoparticle (LNP) has been shown to be effective in HCC cell lines [15]. We have evaluated the feasibility of liver-specific anti-miR delivery to achieve sustained target de-repression in the liver tumor without general liver toxicity. We found that an anti-miR-17 LNP, in a transgenic mouse model of MYC-driven HCC, impeded tumor progression without overt signs of hepatic or systemic toxicity. Our results demonstrate that anti-miR-17 therapy may have efficacy for the treatment of MYC-driven HCC.

## RESULTS

### The miR-17 family is overexpressed in MYC-driven human HCC tumors

**Figure 1:**
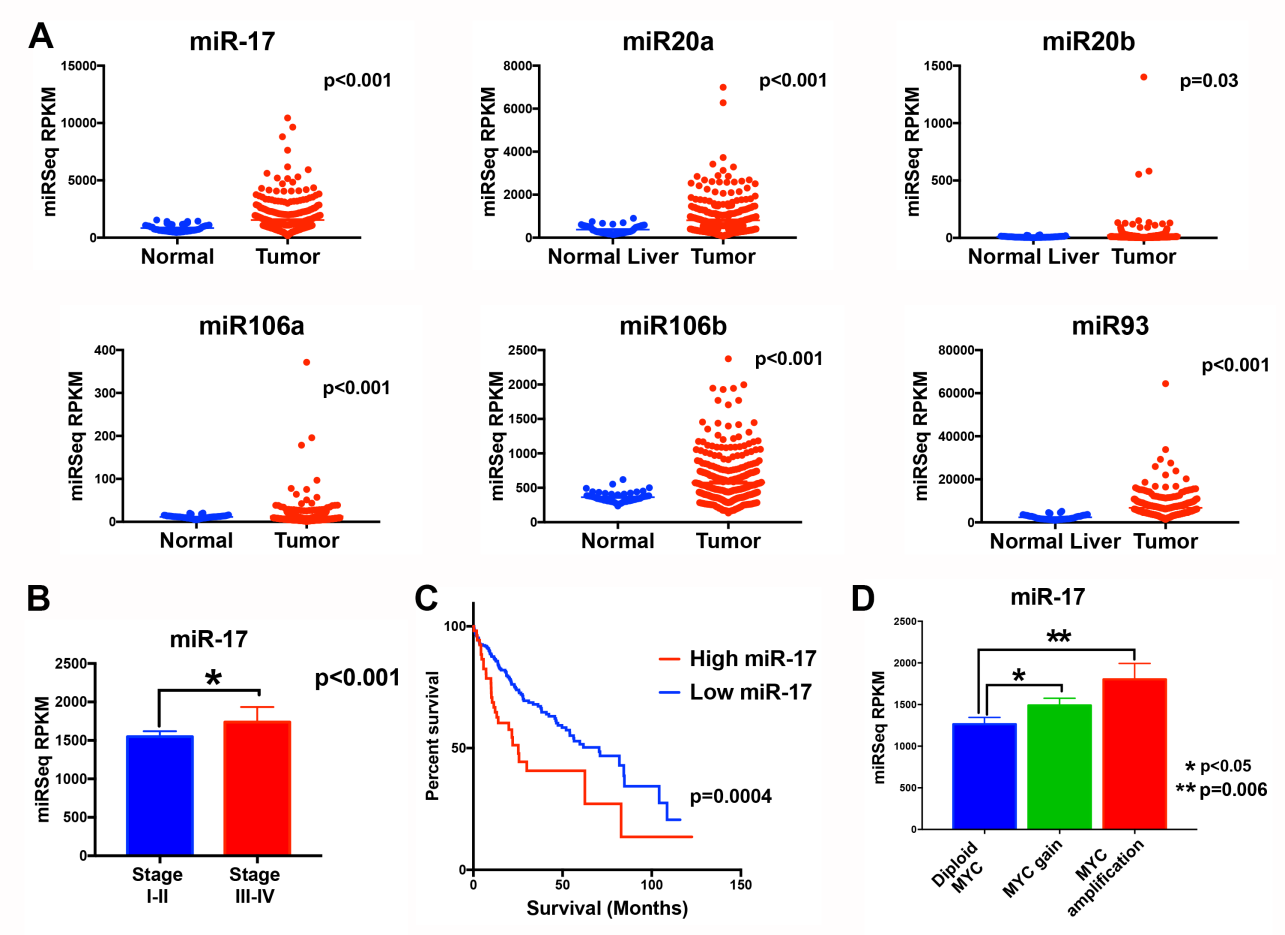
The miR-17 family is overexpressed in MYC-driven human HCC tumors **A**. TCGA analysis of microRNA expression identifies six members of the miR-17 (miR-17, miR-20a, miR-20b, miR-93, miR-106a and miR 106b) in normal liver and human HCC tumor tissue. **B**. MiR-17 expression is higher in tumors of higher stage than lower stage HCC. **C**. Patients with tumors with higher versus lower miR-17 family expression had worse survival. **D**. MiR-17 expression is higher in tumors with MYC genomic amplification than in those with normal diploid MYC.

The miR-17 family (miR-17, miR-20a, miR 20-b, miR 106-a, miR 106-b, miR-93) and MYC expression was examined in human HCC from the cancer genome atlas (TCGA) [16]. All six miRNAs were overexpressed in HCC when compared to the adjacent normal liver, with miR-17 and miR-106b (*p* value<0.001) being the most overexpressed and miR20-b being minimally overexpressed (p value=0.03) (Figure 1A). Tumors could be classified into two groups based on the composite expression of the six miR-17 levels using clustering analysis. The miR-17 family was overexpressed in 60 tumors (16.0%). MiR-17 expression was correlated with higher stage tumor (36.7%) compared to those with lower miR-17 family expression (22%) (p<0.001) (Figure 1B). Median survival in patients with low miR-17 family expression was 70.5 months (95% CI 50.2-90.8) while those with high miR-17 family expression had a significantly poorer median survival of 25.2 months (95% CI 18.5-31.9) (p<0.0001; HR 2.1 (1.4-3.2)). Genomic amplification of MYC oncogene was found in 17.4% (65/374) of HCCs. The miR-17 family members exhibited higher expression in tumors with MYC amplification (Figure 1C and Supplementary Figure 1). Also, expression of miR-17 was significantly correlated with MYC mRNA expression (p=0.0017). Thus, our analysis in this large cohort of human HCCs shows that the miR-17 family is commonly overexpressed in MYC-driven HCC and correlates with a worse clinical outcome.

### Anti-miR-17 therapy impeded MYC-driven tumorigenesis

Anti-miR-17 therapy was examined in an autochthonous transgenic mouse model of MYC-driven HCC (*LAP-tTA/tet-O-MYC*) [17]. A lipid nanoparticle (LNP) encapsulating anti-miR-17 family oligonucleotide was utilized, as has been described previously (RL01-17(5))[15]. In *LAP-tTA/tet-O-MYC* transgenic mice MYC expression was induced at 4 weeks after birth leading to tumorigenesis. MRI imaging was used to identify individual tumor nodules and their size.

**Figure 2:**
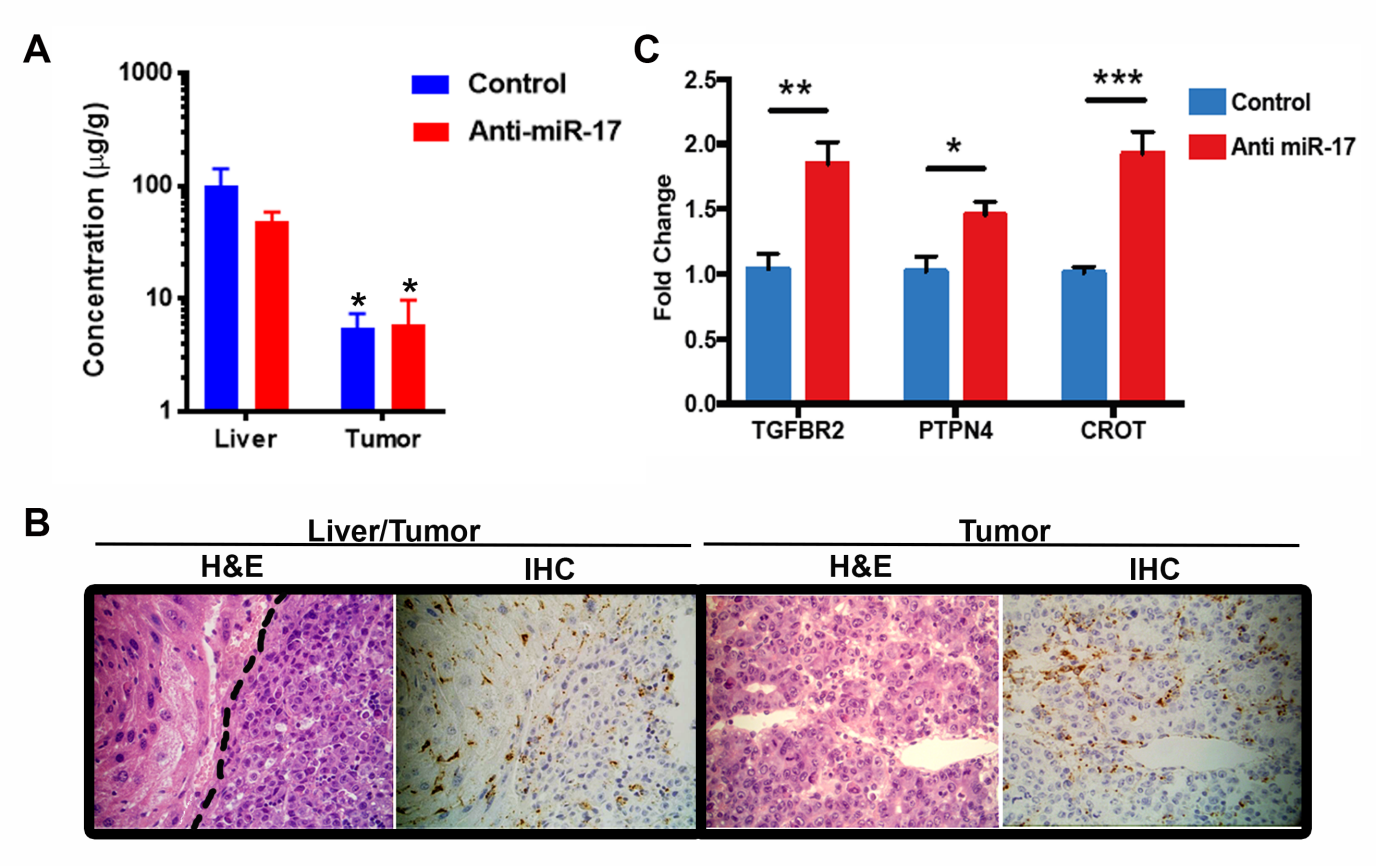
Lipid nanoparticle anti-miR-17 was effectively delivered to the liver **A**. The control and anti-miR-17 drugs were equally detected in both the liver and in the tumor The concentration in the tumor was lower than in the liver (*p<0.05). **B**. Immunohistochemistry for control and anti-miR-17 confirmed drug delivery to both the liver and the tumor. **C**. Anti-miR-17 but not control de-repressed known miR-17 family mRNA targets -TGFBR2, PTPN4, CROT, in MYC-driven HCC tumors (*p<0.05, **p<0.01, ***p<0.001).

First, we showed that LNP anti-miR-17 oligonucleotide can be delivered to the liver. Tumor-bearing mice were treated either with anti-miR-17 oligonucleotide (n=3) or with control oligonucleotide (n=3) for three doses before the mice were sacrificed and the concentration of the oligonucleotide was determined by mass spectrometry (LC/MS). Both the control and anti-miR-17 oligonucleotide compound were detected in both the liver and the tumor, but the concentration in the tumor (mean 5.7 microgm/gm) was lower than in the normal liver (mean 25.4 microgm/gm) (p<0.05) (Figure 2A). Immunohistochemistry using an antibody that recognizes the phosphorothioate backbone of the oligonucleotide confirmed delivery both to the liver and the tumor (Figure 2B). Functional drug delivery was confirmed based on predicted transcriptional changes in liver tumors. Anti-miR-17 oligonucleotide but not control oligonucleotide de-repressed known miR-17 family mRNA targets like TGFBR2 [18], PTPN4 [19] and CROT 15] in MYC-driven HCC tumors (Figure 2C). Hence, LNP delivery of anti-miR17 is indeed distributed to HCC tumors and associated with de-repression of miR-17 targets.

**Figure 3:**
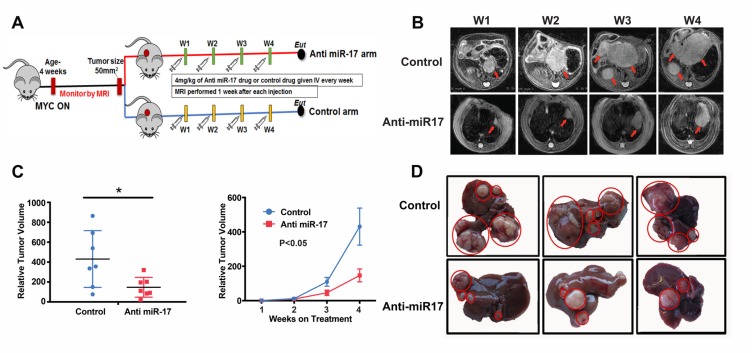
Anti-miR-17 therapy impeded MYC-driven tumorigenesis **A**. Schematic representation of experimental methods. MYC HCCs were induced by activating MYC at 4 weeks of age. Mice were treated with control or Anti-miR-17 oligonucleotide once tumor volume of 50 mm3 was reached. Mice were imaged every week with MRI and tumor volume measured. Mice were sacrificed after they received 4 doses of therapy at weekly intervals. **B**. Serial MRI images of representative mouse from control and anti-miR 17 groups showing delayed tumor progression in latter group. Red arrows point to the tumors. **C**. Tumor volume from mice treated with control of anti-miR-17 oligonucleotide at week 4 was normalized to tumor volume at week one. At week 4, tumors were larger in the control group than in the anti-miR-17 group. Also, the growth curve of the tumors from week one to week 4 has been plotted to demonstrate the difference. **D**. Gross morphology of liver tumors after 4 doses of treatment shows that mice treated with control oligonucleotide had larger tumors and more numerous tumors than those treated with anti-miR-17 therapy.

Next, therapeutic efficacy of anti-miR-17 therapy was determined. MYC-induced transgenic tumors were treated when tumors had reached a size of at least 50mm^3^, as measured by MRI. Intravenous delivery of anti-miR-17 LNP (4 mg/kg) (n=7) or control oligonucleotide (n=7) once a week for 4 weeks was performed. The treatment was well tolerated and there was no significant weight loss in either group. Treatment with anti-miR-17 versus control impeded tumorigenesis (Figure 3B). Three dimensional tumor volume assessment by MRI showed that normalized tumor volume at week 4 was significantly higher in the mice treated with control compound (430±107 mm^3^) than the mice treated with anti-miR-17 oligonucleotide (mean 147±37 mm^3^) (p=0.028) (Figure 3C; Supplementary Figure 2A). Also, anti-miR-17 treated transgenic mice had smaller and fewer liver tumors (4.1±0.32 grams) than control mice (6.2±0.7 grams) (p=0.03) (Figure 3D, supplementary Figure 2B). Our results demonstrate that systemic delivery of LNP-encapsulated anti-miR-17 family oligonucleotide significantly delayed tumor progression in a mouse model of MYC-driven HCCs.

### Anti-miR-17 inhibits HCC cell proliferation and increases apoptosis

**Figure 4:**
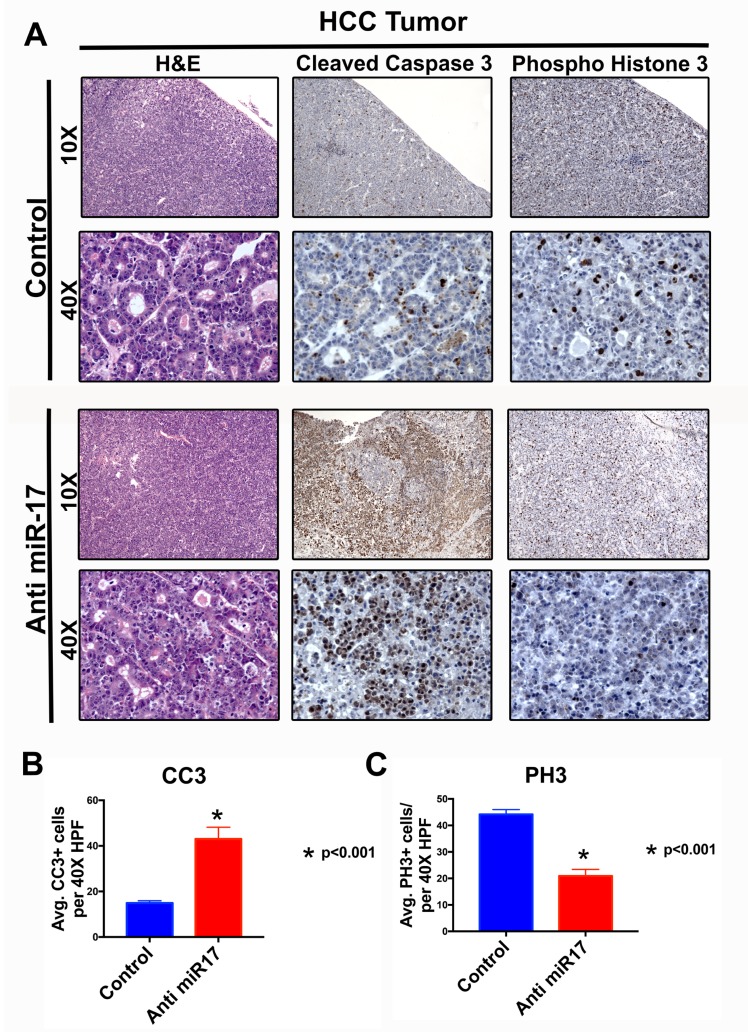
Anti-miR-17 inhibited HCC cell proliferation and increased apoptosis **A**. H&E, IHC staining for cleaved caspase 3 (CC3) and phospho histone 3 (PH3) at 10X and 40X magnification from representative tumor samples from mice treated with control or anti-miR-17 therapy. **B**. Quantification of CC3 and pH3 staining by determining the average staining in five 40X magnification fields shows apoptosis is increased with anti-miR-17 therapy and proliferation is decreased.

We examined the mechanism by which miR-17 inhibition blocked MYC-induced HCC tumorigenesis. Mice treated with anti-miR-17 therapy versus control demonstrated significantly increased apoptosis as measured by activated caspase staining and decreased cellular proliferation as measured by phospho histone3 staining by immunohistochemistry (Figure 4A and 4B). Further, we observed no overt toxic effects on normal liver tissue (Supplementary Figure 3). MYC and miR-17∼92 have been implicated in modulation of anti-tumor immune response [20, 21]. To see if miR-17 suppression influenced the immune response or if the LNP led to any deleterious immunostimulatory effects, we measured T-cells (CD4+) and macrophages (F4/80) infiltration in anti-miR-17 versus control treated MYC-induced HCC and found no significant differences (Supplementary Figure 4).

### Anti-miR-17 therapy leads to de-repression of miR-17 targets

**Figure 5:**
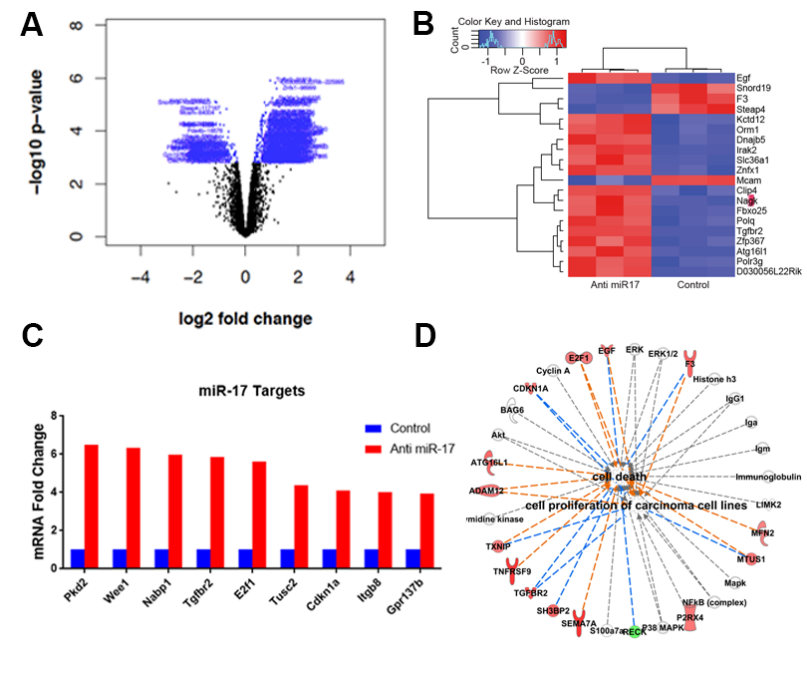
Anti-miR-17 therapy leads to de-repression of miR-17 targets **A**. Volcano plot shows genes differentially expressed with anti-miR-17 treatment. Genes colored in blue represent the differentially expressed genes. **B**. Heat map of the top 20 genes differentially expressed upon treatment with control versus anti-miR-17 oligonucleotide. **C**. De-repression of several known targets of miR-17 was seen including E2f1, Tgfbr2 and Cdkna1 in tumors treated with anti-miR-17 therapy. **D**. Pathway analysis of differentially expressed genes revealed that enhancement of cell death and inhibition of cellular proliferation (p=1.9X10^-5^) were key functional changes induced by anti-miR17 therapy. The lines in orange represent inhibition and the lines in blue show activation.

We further examined the effects of anti-miR-17 therapy using MYC-induced HCC tumor derived cell lines. Notably, we confirmed that MYC regulates miR-17, but not miR-19 or miR-20 expression (Supplementary Figure 5), as has also been described by us previously [9]. Next, we evaluated the influence on gene expression of miR-17 inhibitor TuD to inhibit endogenous miR-17 family activity. A total of 1323 genes were differentially expressed between the anti-miR-17 treated and control groups (Figure 5A) (p<0.05) (Supplementary Table 1). The top 20 most differentially expressed genes are shown in Figure 5B. Pathway analysis revealed activation of several pathways (Supplementary Figure 6A) involved in apoptosis and cell cycle arrest like PTEN pathway and p53 signaling pathway (Supplementary Figure 6B). Furthermore, by TargetScan and Sylamer analysis, we found global de-repression of transcripts that contain putative miR-17 binding sites in their 3’-UTRs (Supplementary Figure 7). A total of 743 genes considered to be potential direct targets of miR-17 family were identified (Supplementary Table 2). The de-repression of several known targets of miR-17 [22-24] like E2f1, Tgfbr2 and Cdkna1 was observed (Figure 5C). Network analysis of differentially expressed genes revealed that enhancement of cell death and inhibition of cellular proliferation (p=1.9X10^-5^) were key functional changes induced by anti-miR17 therapy (Figure 5D).

### Anti-miR-17 therapy inhibits MYC induced transcriptional program

**Figure 6:**
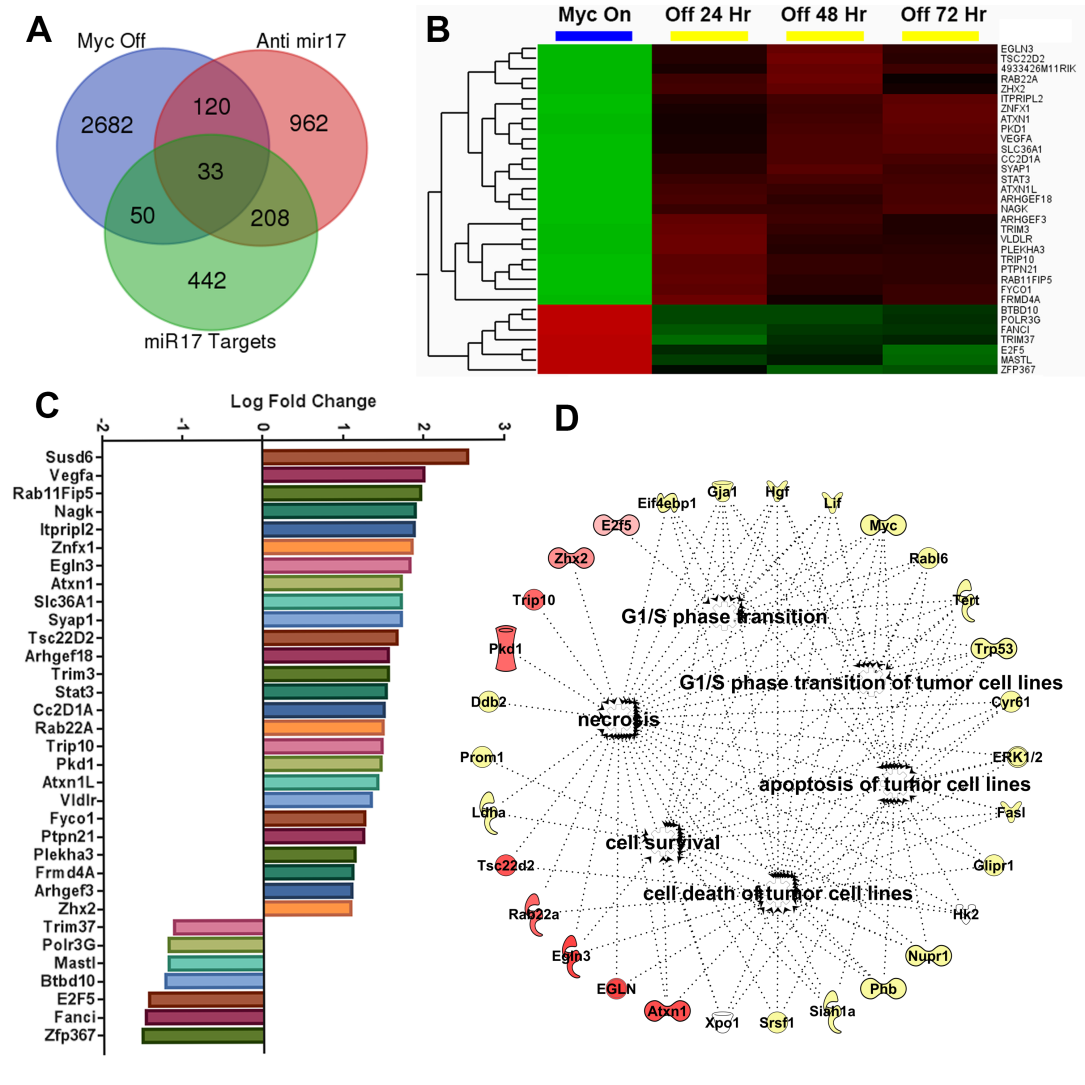
Anti-miR-17 therapy inhibits MYC induced transcriptional program **A**. Venn diagram showing overlap of three separate gene sets i) Genes differentially expressed in conditional cell lines when MYC is inactivated ii) genes differentially expressed in MYC conditional cell lines when treated with anti-miR-17 therapy iii) Genes that are identified to be targets of miR17 on Targetscan analysis. **B**. Heatmap shows expression of the 33 genes identified as overlapping between above 3 gene sets in MYC ON versus OFF states. **C**. Bar graph shows log fold change of expression changes of these 33 genes in cell lines treated with control versus anti-miR-17 therapy. **D**. Network analysis of the 33 genes revealed the most activated pathways were cell death and survival, cell cycle regulation and apoptosis. The molecules in red are the most over expressed in the tumors treated with anti-miR-17 therapy while the other molecules in yellow are indirectly involved in the network.

**Figure 7:**
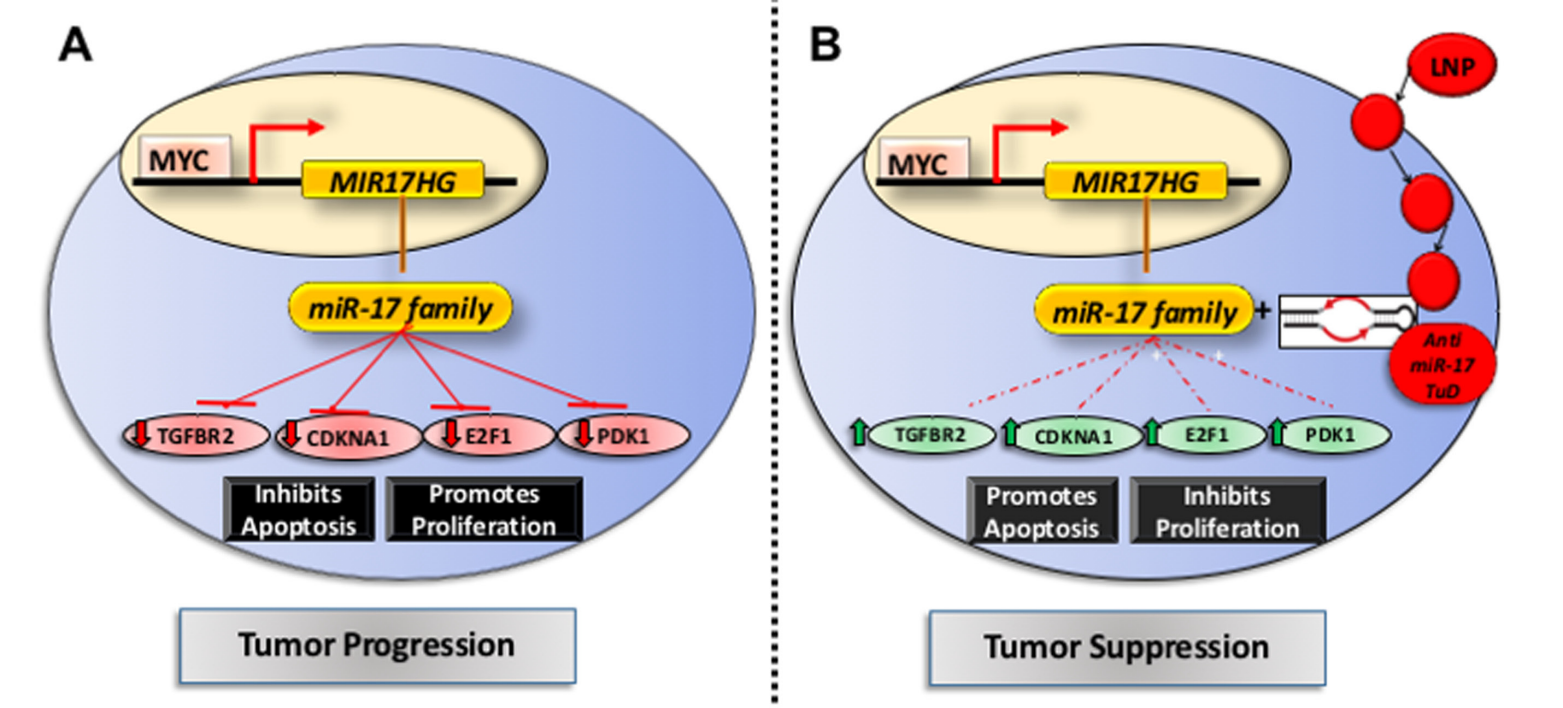
Mechanism of action of anti-miR17 therapy in MYC driven HCCs **A**. In the basal state, MYC transcriptionally activates expression of miR-17 family which leads to repression of its known targets like Tgfbr2, Cdkna1, E2f1 and Wee1 thus promoting tumor progression. **B**. The lipid nano particle (LNP) delivered anti-miR-17 tough decoy anti sense oligonucleotide is delivered into the HCC cell leading to inhibition of anti-miR-17 activity and de-repression of its targets. This results in cell cycle arrest and apoptosis, thus impeding tumor progression.

MYC maintains tumorigenesis via the upregulation of miR-17∼92 family and suppression of its target genes [9]. We compared the transcriptional changes induced by treatment with anti- miR-17 therapy with those induced by inactivating the MYC oncogene. The MYC oncogene was inactivated in the conditional MYC cell line by treating the cells with doxycycline (supplementary Figure 8). Global transcription profiling was performed at 24, 48 or 72 hours after MYC inactivation. A total of 2885 genes (p<0.05) were differentially expressed between the MYC on and off states (Supplementary Table 3); whereas 1323 genes were differentially expressed upon anti-miR-17 therapy, with 153 genes overlapping. Next, we identified genes amongst these that are targets of miR-17 by TargetScan analysis (Figure 6A). We identified 33 direct targets of miR-17 (Supplementary Table 4) which were differentially expressed either upon MYC inactivation (Figure 6B) or upon inhibition of miR-17 (Figure 6C). Our results indicated these genes are part of a common transcriptional program between MYC and miR-17. Network analysis of the 33 genes revealed that the most activated pathways involved cell death (p=5.3X10^-9^), cell cycle regulation (p=5.0X10^-8^) and apoptosis (p=5.2X10^-7^) (Figure 6D). Our results suggest that MYC induces a transcriptional program which, in part, suppresses cell death and apoptosis via miR-17 activation. To summarize, anti-miR-17 therapy induces apoptosis and cell cycle arrest by specifically de-repressing several targets in the MYC pathway and thus delays tumorigenesis in MYC-driven HCCs (Figure 7).

## DISCUSSION

The MYC oncogene is overexpressed in most human HCCs. Here we show that MYC can both induce the expression and is correlated with the overexpression of miR-17. Further, the inhibition of miR-17 using a LNP delivery of an anti-oligonucleotide in an autochthonous transgenic mouse model of MYC-induced HCC impedes tumor growth. We confirmed delivery into tumors, de-repression of miR-17 targets, but did not observe any overt liver-specific or systemic toxicity with this therapy. We conclude that anti-miR-17 therapy is potential novel therapy for MYC-associated HCC.

Previously we [17, 25-28] and others [29] induced reversible tumorigenesis upon experimental MYC inactivation. However, there are no existing therapies that target MYC directly. Targeting MYC regulated microRNAs is an attractive treatment approach since miRNAs affect the expression of a network of target genes and affect a pathway at multiple levels [30-32]. Earlier, we and others have reported that miR-17-92 is causally responsible for at least part of the mechanism by which MYC maintains a neoplastic state [9, 33, 34]. We hypothesized that targeting miR-17 could be effective strategy for MYC driven cancers. Cell-line based studies have shown that targeting miR-17 can suppress tumor proliferation [15].

One of the main challenges in developing miRNA-based therapies is ensuring tissue-specific delivery of the anti-sense oligonucleotide [35]. We used a lipid nanoparticle to deliver the anti-miR-17 oligonucleotide to the liver and we successfully demonstrate that the drug is delivered both to the tumor and to the normal liver. Several barriers are known to impede effective drug delivery to solid tumors including poorly organized vasculature and increased interstitial fluid pressure [36]. This likely explains the observation that the concentration of the drug was lower in the tumor tissue than in the surrounding liver. But we did confirm that LNP delivery of anti-miR-17 therapy, even at this lower concentration, was associated with significant de-repression of known miR17 targets in the tumor. Also, we demonstrated that the anti-miR-17 therapy can effectively delay tumor progression without causing any observed liver toxicity. Further, we have shown that anti-miR-17 therapy promoted apoptosis and proliferative arrest in tumors. Finally, network analysis of gene expression data suggested that the mechanism of action of LNP delivered anti-miR-17 was related to blocking a MYC induced transcriptional program which regulates apoptosis and cell cycle progression.

To date, few studies have suggested efficacy of anti-miR therapy in autochthonous primary tumor models. Targeting miR-221 was reported to be effective in delaying tumor progression but this was performed in a xenograft model of liver cancer [37]. Anti-miR-34a was reported to be an effective therapy but the efficacy was confined to tumors with betacatenin mutation [38]. Recently, anti-miR-17 LNP was shown to be effective in HCC but this study was performed with cell lines and cell-line based xenografts so they were unable to study liver-specific drug delivery or the potential role of the host immune response [15]. Our study is the first to demonstrate that anti-miR-17 therapy can impede MYC induced tumorigenesis in an autochthonous mouse model.

## MATERIALS AND METHODS

### Transgenic mice

The LAP-*tTA*, and *TetO-MYC* transgenic lines have been described previously [9, 20]. Doxycycline (Sigma) was administered in the drinking water weekly at 0.1 mg/mL during mating and continuing until mice reached approximately 4 weeks of age. Animals were euthanized once treatment is completed or upon disease morbidity as assessed by tumor burden. All procedures were performed in accordance with APLAC protocols and animals were housed in a pathogen-free environment.

### Lipid nanoparticle drug delivery

Cationic lipid RL01 was synthesized by Regulus Therapeutics. LNPs encapsulating anti-miR-17 or control oligonucleotide were prepared by mixing oligonucleotide with lipid mixture. The concentration of anti-miR-17 was 2.6mg/mL and lipid content was 57% and the percentage of encapsulated anti-miR-17 was 98% (13).

### Small animal imaging

MRI scans were performed using a 7T small animal MRI scanner (Bruker Inc., Billerica, MA, Stanford Small Animal Imaging Facility, CA) equipped with a 40 mm Millipede RF coil (ExtendMR LLC, Milpitas, CA). Under anesthesia by inhalation of 1–3% isoflurane mixed in with medical-grade oxygen via nose-cone, the animals were placed supine with the respiratory sensor, head first with the kidneys centered with respect to the center of a RF coil. MRI acquisitions were gated using the respiratory triggering. The bore temperature was kept at 28±1 °C. Two-dimensional (2D) scout images on three orthogonal planes (axial, coronal and sagittal) were acquired to ensure the positioning. For tumor detection, a respiration triggered T2-weighted 3D turbo spin echo sequence was used (TR/TE 3000/205 ms, voxel size (0.22 mm^3^). The isotropic voxel size of 0.22 mm in all directions provides a high in plane and across plane resolution. Thereby, the location of one tumor could be defined in all three orientations using specific landmarks, such as major vessels or other tumors. For respiration monitoring, a pressure-sensitive pad was placed on the animal bed directly underneath the animal. The compression and decompression of the pad were measured and the generated signal (SAI Instruments, Stony Brook, NY) was finally fed to the MRI scanner. The average acquisition time was approximately 5 min, depending on the respiration rate of the animal. T2-weighted anatomical imaging was performed approximately once weekly. Anatomical and parametric images were analyzed and tumor volumes were measured using Osirix image processing software (Osirix, UCLA, and Los Angeles, CA).

### Drug concentration assessment

Mass spectrometry (MS) based methods were used to measure concentrations of anti-miR oligonucleotides in mouse liver and implanted tumor after systemic delivery of LNPs. Dissected tissues were extracted by a combination of liquid-liquid (LLE) and solid-phase extraction (SPE), followed by HPLC separation and Time of Flight (TOF) detection. In the HPLC-TOF method, specificity was achieved through high-resolution MS signals that allowed accurate molecular weight determination. The obtained MS signals were integrated and normalized to that of an internal standard (IS), which allowed analyte concentrations to be determined.

### Immunohistochemistry and immunofluorescence

Paraffin embedded tumor sections were deparaffinized by successive incubations in xylene, graded washes in ethanol, and PBS. Epitope unmasking was performed by steaming in 0.01 mol/L citrate buffer (pH 6.0) for 45 minutes. Paraffin embedded sections were immunostained with MYC (1:150, Epitomics), or cleaved capsase 3 (1:100, Cell Signaling technology), phospho histone 3 (1:200, Cell Signaling Technology), CD4 (1:1000, Abcam) and F4/80 ( 1: 150, Life technologies) overnight at 4oC. The tissue was washed with PBS and incubated with biotinylated anti-rabbit or anti-mouse for 30 minutes at room temperature (1:300 Vectastain ABC kit, Vector Labs). Sections were developed using 3,3’- Diaminobenzidine (DAB), counterstained with hematoxylin, and mounted with permount. Images were obtained on a Nikon microscope.

### TCGA analysis

We used microRNA expression, mRNA expression and somatic copy number variation (CNV) data generated by The Cancer Genome Atlas (TCGA) from HCC specimens (373 tumors; 50 surrounding normal liver tissues). We downloaded the file containing level 3 normalized microRNA data, level 3 normalized RSEM (RNA-Seq by Expectation Maximization) data and the level 3 somatic CNV data from the Firehose run of the Broad Genome Data Analysis Center [39].

### Tumor derived cell lines

Conditional HCC cell lines were derived from LAP-*tTA*, and *TetO-MYC* mice. MYC inactivation was achieved with 20ng/ml doxycycline treatment. Cells were grown in DMEM (Invitrogen), supplemented with 10% FBS (Invitrogen), and cultured at 37°C in a humidified incubator with 5% CO_2_.

### RNA sequencing and miRNA target analysis

Mouse MYC-driven HCC cells were transfected with 25nM of anti–miR-17 compound or control in triplicate. Total RNA was isolated from the cells 24 hours after transfection and processed for RNASeq analysis. mRNA expression profiles were determined using next-generation sequencing (NGS) on the Illumina HiSeq 2000 platform producing 50bp paired-end reads. A mean of 21,556,260 read pairs passed filter and were successfully mapped to the reference per sample, with a standard deviation of 4,906,270. In terms of genes, 77.3% (8831/11427) had measurable expression, in at least one of the six samples; and 67.9% (7758/11427) genes saw measurable expression levels in all six samples. Sylamer analysis was performed through a Sylarray web server [40]. TargetScan 6.2 was used to identify putative conserved targets of miR-17 [41]. De-repression in a cell line was defined as upregulation >1.3 fold (p-value<0.05). An absolute fold-change threshold of >1.2 (p value<0.05) was used to select genes for pathway analysis.

### RNA isolation and quantitative real-time PCR analysis

Total RNA and microRNAs were prepared from cells and tissues using the miRNeasy miRNA isolation kit (Qiagen) according to the manufacturer’s instructions. cDNA and miR cDNA synthesis were performed with high capacity RNA to cDNA kit and TaqMan MicroRNA Reverse Transcription kit respectively (Life Technologies). TaqMan assays were performed on a ViiA7 (Applied Biosystems) or a QuantStudio 12K Flex Real Time PCR System (Applied Biosystems) using the following conditions: 50°C for 2 minutes; 95°C for 10 minutes; 40 cycles of 95°C for 15 seconds; and 60°C for 1 minute. The expression of *Ubiquitin* was used for normalization of mRNA samples, whereas *U*6 was used for normalization of small RNA expression.

### Statistical analysis

The results (Mean±SD) were subjected to statistical analysis by Student’s t-test or one-way analysis of variance (ANOVA) with a significance threshold of *P* <0.05. Chi square test was used to compare categorical variables. Kaplan Meier test was used for survival analysis. The statistical software SPSS (IBM) and Graphpad Prism (Graphpad Software Inc.) was used to perform the analysis.

## CONCLUSIONS

We report the first demonstration of an effective use of an anti microRNA based therapy for MYC-driven liver tumors. We confirmed delivery of anti-miR-17 lipid nanoparticles and de-repression of miR-17 targets. Our results suggest that a subgroup of human patients with MYC and miR17 associated HCC may be good candidates for anti-miR-17 therapy.

## SUPPLEMENTARY MATERIALS FIGURES AND TABLES










