# Supplementary material for: Anti-miR-17 therapy delays tumorigenesis in MYC-driven hepatocellular carcinoma (HCC)
Source: Oncotarget. 2017 Nov 9;9(5):5517–28. doi: 10.18632/oncotarget.22342 (PMC5814155; doi:10.18632/oncotarget.22342)
Supplement: Supplementary file 1 [file oncotarget-09-5517-s001.pdf]

Anti-miR-17 Therapy Delays Tumorigenesis in MYC-driven Hepatocellular Carcinoma (HCC)

SUPPLEMENTARY MATERIALS

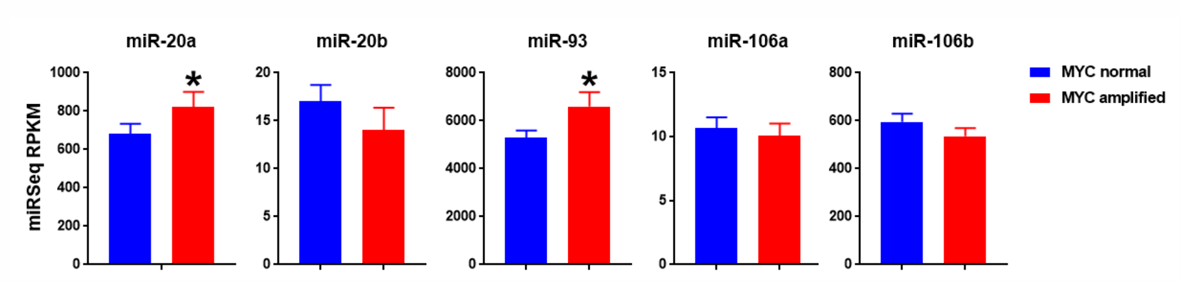

Supplementary Figure 1: TCGA microRNA expression data from human HCCs reveals that miR20a and miR93 are significantly overexpressed in tumors with MYC amplification while miR 20b, miR 106a and miR 106b are not significantly differentially expressed.

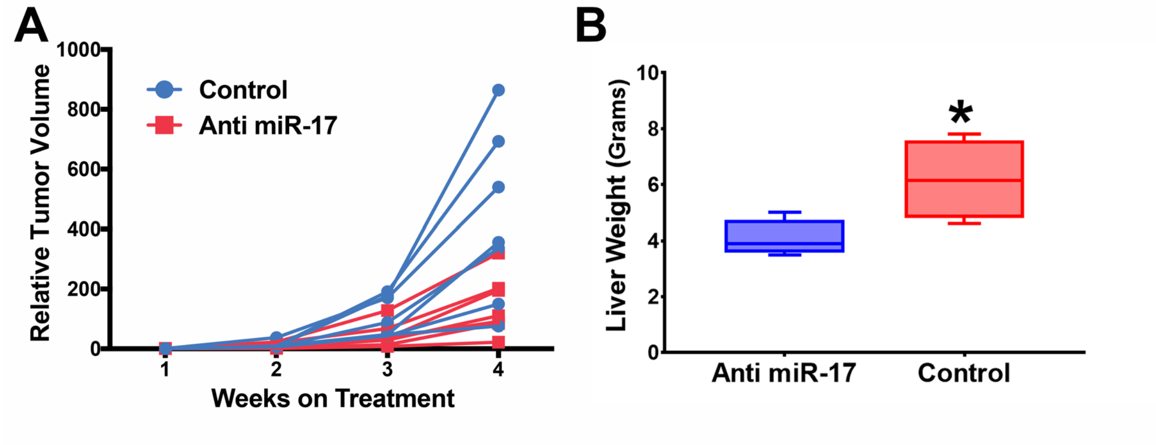

Supplementary Figure 2: A. Tumor volumes of individual mice in the control oligonucleotide treated and anti-miR-17 treated arms as measured by MRI based volumetric analysis is shown. B. The control oligonucleotide treated mice had significantly higher liver weight after 4 weeks of treatment when compared to anti-miR-17 treated mice.

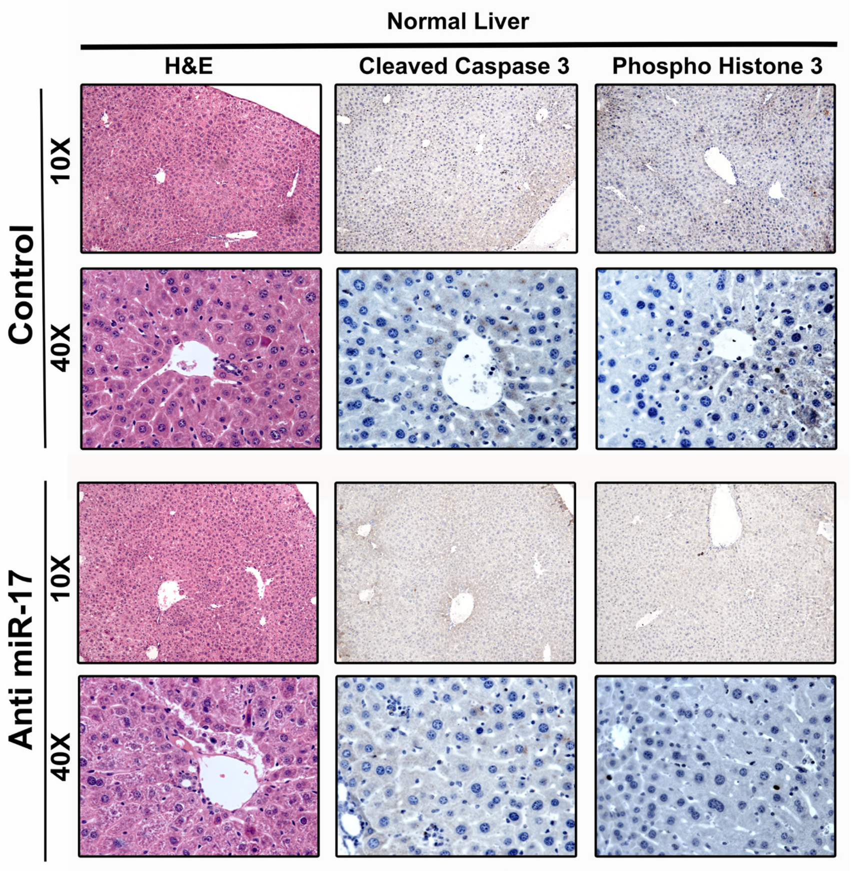

Supplementary Figure 3: H&E, IHC staining for cleaved caspase 3 (CC3) and phospho histone 3 (PH3) at 10X and 40X from representative normal liver samples from mice treated with control or anti-miR-17 therapy did not show any significant changes in apoptosis or proliferation.

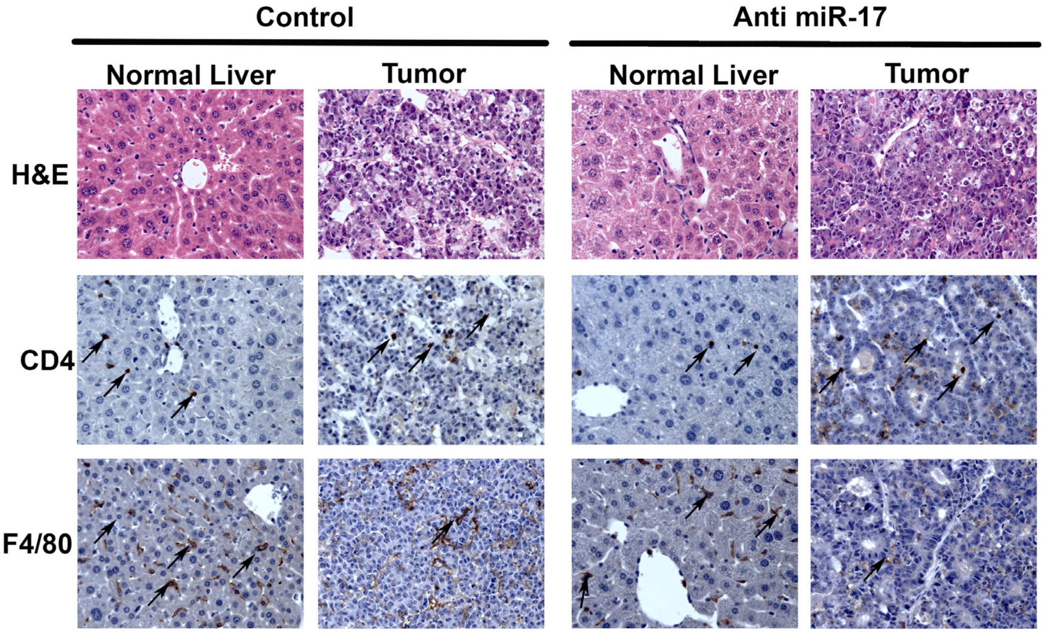

Supplementary Figure 4: H&E, IHC staining for CD4 T cells and macrophage marker (F4/80) at 10X and 40X from representative tumor and normal liver samples from mice treated with control or anti-miR-17 therapy did not show any significant changes in immune infiltration. The little black arrows point to the infiltrating immune cells.

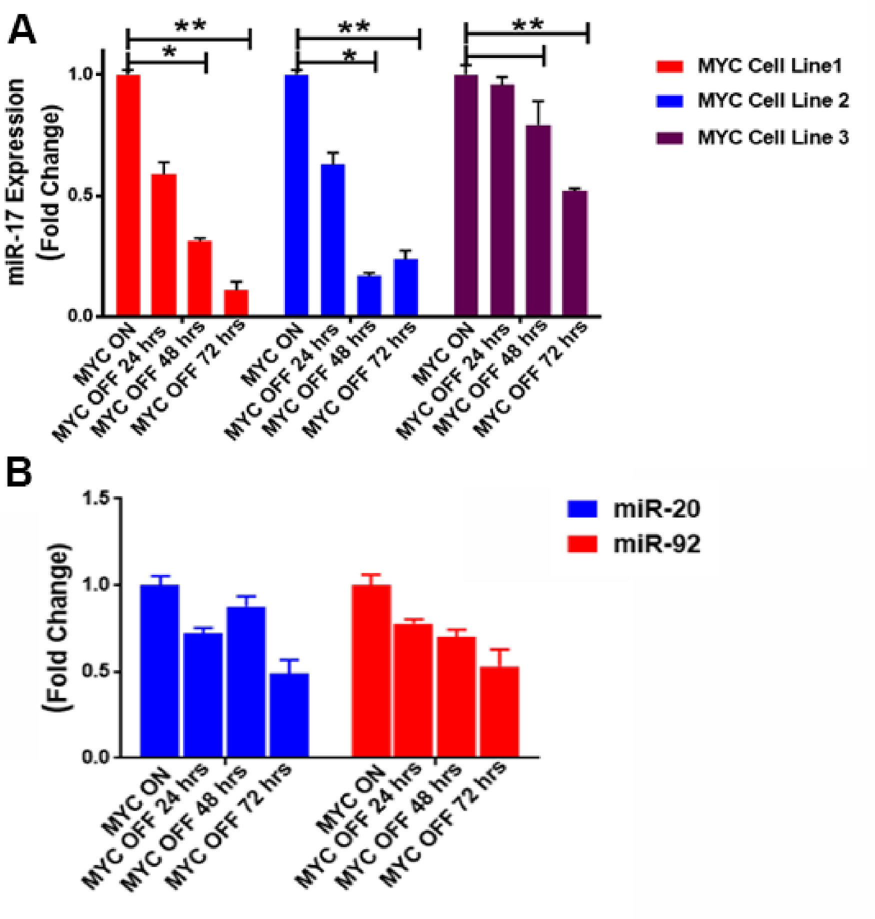

Supplementary Figure 5: A. Quantitative PCR of mRNA derived from 3 different times points after MYC inactivation in 3 different cell lines shows that miR-17 is progressively lower once MYC is turned off. B. miR 20 and miR92 are not significantly correlated with MYC expression.

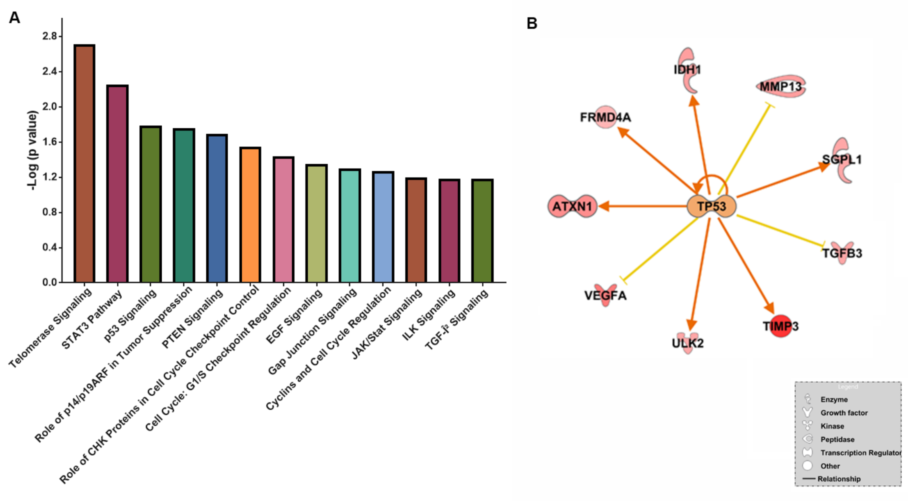

Supplementary Figure 6: A. Top results of pathway analysis of genes differentially expressed between MYC conditional cell lines treated with control oligonucleotide or anti-miR-17 therapy. B. The TP53 pathway is activation is depicted as a network of molecular interactions amongst the genes differentially expressed between MYC conditional cell lines treated with control oligonucleotide or anti-miR-17 therapy.

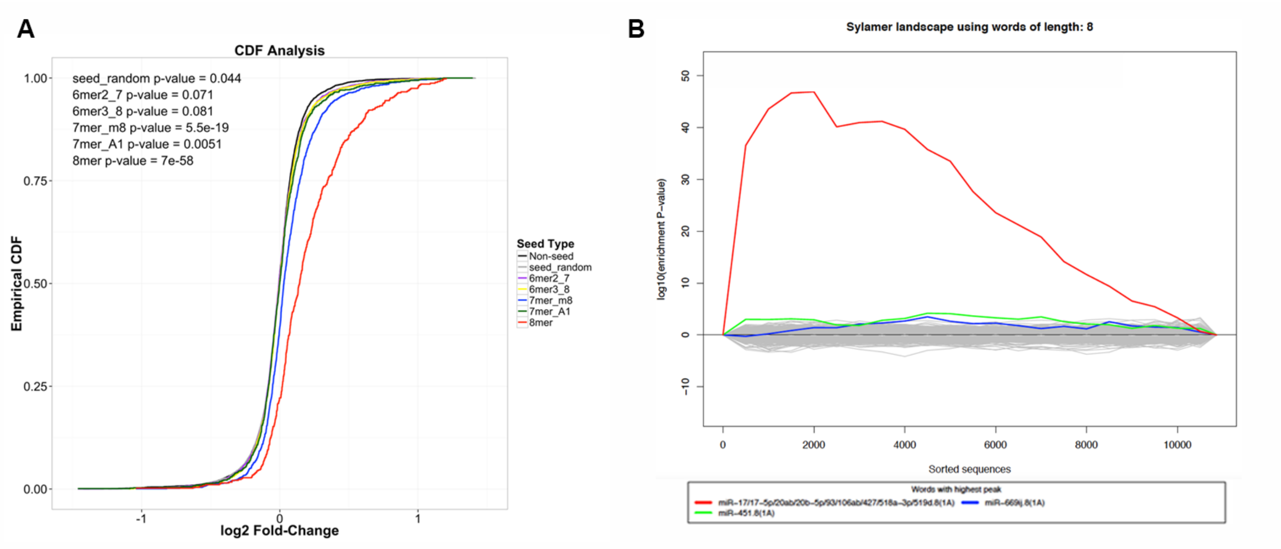

Supplementary Figure 7: A. TargetScan analysis of genes differentially expressed between MYC conditional cell lines treated with control oligonucleotide or anti-miR-17 therapy shows global de-repression of transcripts that contain putative miR-17 binding sites in their 3'-UTRs. Cumulative distribution function (CDF) plots are shown for Log2 fold-changes of mRNAs differently expressed between anti-miR-17 therapy and control. Statistical significance of the differences between fold-changes of mRNAs with and without the miR-17 family seed sequence was determined using one-sided Kolmogorov-Smirnov tests. B. Sylamer plot analysis for the Seed Complementary Region (SCR) words corresponding to the seed of miR-17 (red). Log10- transformed and sign-adjusted enrichment P values for each SCR word, relative to P values of all other words, are plotted on the Y-axis, against the ranked gene list on the X-axis.

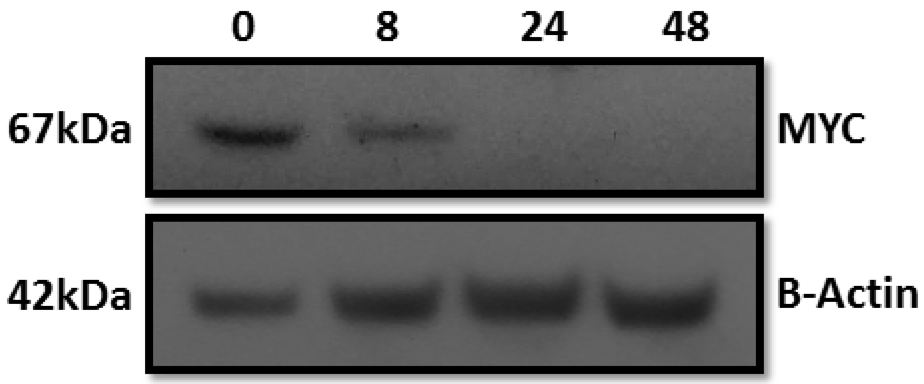

Supplementary Figure 8: Western blotting analysis of MYC expression in MYC conditional cell lines derived from primary HCC shows decreased MYC expression when cells are treated with Doxycycline. Beta actin serves as loading control.

For Supplementary Tables see in Supplementary Files.
