# Supplementary material for: Anti-miR-17 therapy delays tumorigenesis in MYC-driven hepatocellular carcinoma (HCC)
Source: Oncotarget. 2017 Nov 9;9(5):5517–28. doi: 10.18632/oncotarget.22342 (PMC5814155; doi:10.18632/oncotarget.22342)
Supplement: Supplementary file 2 [file oncotarget-09-5517-s002.docx]

**SupplementaryTable 1: Differentially expressed genes in MYC conditional cell lines treated with Anti miR-17 versus Control TuD**

| **Gene symbol** | **Entrez ID** | **Gene description** | **Anti miR-17 vs. Control Log Fold Change** | **p value** |
| --- | --- | --- | --- | --- |
| Tgfbr2 | 21813 | transforming growth factor, beta receptor II | 1.91 | 1.22E-06 |
| D030056L22Rik | 225995 | RIKEN cDNA D030056L22 gene | 2.21 | 1.44E-06 |
| Znfx1 | 98999 | zinc finger, NFX1-type containing 1 | 2.27 | 2.42E-06 |
| Slc36a1 | 215335 | solute carrier family 36 (proton/amino acid symporter), member 1 | 2.02 | 5.73E-06 |
| Polq | 77782 | polymerase (DNA directed), theta | 2.25 | 7.10E-06 |
| Polr3g | 67486 | polymerase (RNA) III (DNA directed) polypeptide G | 2.26 | 7.40E-06 |
| Kctd12 | 239217 | potassium channel tetramerisation domain containing 12 | 0.45 | 7.50E-06 |
| Dnajb5 | 56323 | DnaJ (Hsp40) homolog, subfamily B, member 5 | 1.87 | 8.12E-06 |
| Irak2 | 108960 | interleukin-1 receptor-associated kinase 2 | 1.64 | 8.32E-06 |
| Snord19 | 1E+08 | small nucleolar RNA, C/D box 19 | 0.47 | 8.40E-06 |
| F3 | 14066 | coagulation factor III | 1.81 | 1.01E-05 |
| Atg16l1 | 77040 | autophagy related 16-like 1 (S. cerevisiae) | 1.73 | 1.05E-05 |
| Steap4 | 117167 | STEAP family member 4 | 0.6 | 1.44E-05 |
| Zfp367 | 238673 | zinc finger protein 367 | 1.71 | 2.00E-05 |
| Nagk | 56174 | N-acetylglucosamine kinase | 1.69 | 2.02E-05 |
| Orm1 | 18405 | orosomucoid 1 | 2.21 | 2.03E-05 |
| Mcam | 84004 | melanoma cell adhesion molecule | 0.51 | 2.04E-05 |
| Egf | 13645 | epidermal growth factor | 1.58 | 2.46E-05 |
| Fbxo25 | 66822 | F-box protein 25 | 1.61 | 2.55E-05 |
| Clip4 | 78785 | CAP-GLY domain containing linker protein family, member 4 | 2.23 | 2.55E-05 |
| Gpr137b | 83924 | G protein-coupled receptor 137B | 1.57 | 2.81E-05 |
| Slc22a23 | 73102 | solute carrier family 22, member 23 | 1.54 | 3.30E-05 |
| Fyco1 | 17281 | FYVE and coiled-coil domain containing 1 | 1.93 | 3.57E-05 |
| Slc29a2 | 13340 | solute carrier family 29 (nucleoside transporters), member 2 | 1.69 | 3.92E-05 |
| Adam12 | 11489 | a disintegrin and metallopeptidase domain 12 (meltrin alpha) | 1.61 | 4.14E-05 |
| Mtf1 | 17764 | metal response element binding transcription factor 1 | 1.53 | 4.16E-05 |
| Rcan3 | 53902 | regulator of calcineurin 3 | 1.49 | 4.30E-05 |
| Itgb8 | 320910 | integrin beta 8 | 1.84 | 4.32E-05 |
| Agfg2 | 231801 | ArfGAP with FG repeats 2 | 1.89 | 4.45E-05 |
| P2rx4 | 18438 | purinergic receptor P2X, ligand-gated ion channel 4 | 1.54 | 4.82E-05 |
| Bhlhe41 | 79362 | basic helix-loop-helix family, member e41 | 1.65 | 5.02E-05 |
| Mink1 | 50932 | misshapen-like kinase 1 (zebrafish) | 2 | 5.51E-05 |
| Cdc37l1 | 67072 | cell division cycle 37-like 1 | 1.54 | 5.66E-05 |
| Klhl30 | 70788 | kelch-like 30 (Drosophila) | 0.51 | 5.68E-05 |
| Plekha3 | 83435 | pleckstrin homology domain-containing, family A (phosphoinositide binding specific) member 3 | 1.92 | 5.78E-05 |
| Gdpd2 | 71584 | glycerophosphodiester phosphodiesterase domain containing 2 | 0.56 | 5.80E-05 |
| Hif1an | 319594 | hypoxia-inducible factor 1, alpha subunit inhibitor | 1.47 | 5.86E-05 |
| Mafb | 16658 | v-maf musculoaponeurotic fibrosarcoma oncogene family, protein B (avian) | 0.51 | 6.05E-05 |
| Cdkn1a | 12575 | cyclin-dependent kinase inhibitor 1A (P21) | 1.57 | 6.18E-05 |
| Mospd2 | 76763 | motile sperm domain containing 2 | 1.74 | 6.55E-05 |
| Pdk4 | 27273 | pyruvate dehydrogenase kinase, isoenzyme 4 | 2.13 | 7.16E-05 |
| Orm2 | 18406 | orosomucoid 2 | 1.88 | 7.67E-05 |
| Gpatch11 | 53951 | NA | 1.5 | 7.80E-05 |
| Ginm1 | 215751 | glycoprotein integral membrane 1 | 2.34 | 7.95E-05 |
| Rufy2 | 70432 | RUN and FYVE domain-containing 2 | 1.51 | 8.68E-05 |
| A930004D18Rik | 77940 | RIKEN cDNA A930004D18 gene | 1.52 | 8.93E-05 |
| Mtus1 | 102103 | mitochondrial tumor suppressor 1 | 1.54 | 9.61E-05 |
| Pde4b | 18578 | phosphodiesterase 4B, cAMP specific | 0.64 | 0.000104 |
| Slc25a40 | 319653 | solute carrier family 25, member 40 | 2 | 0.000114 |
| Sema7a | 20361 | sema domain, immunoglobulin domain (Ig), and GPI membrane anchor, (semaphorin) 7A | 2.3 | 0.000114 |
| Smoc2 | 64074 | SPARC related modular calcium binding 2 | 1.54 | 0.000117 |
| Pkd2 | 18764 | polycystic kidney disease 2 | 1.41 | 0.000124 |
| BC037034 | 231807 | cDNA sequence BC037034 | 1.76 | 0.000132 |
| Zfyve9 | 230597 | zinc finger, FYVE domain containing 9 | 1.46 | 0.000141 |
| Cc2d1a | 212139 | coiled-coil and C2 domain containing 1A | 1.78 | 0.000143 |
| Lace1 | 215951 | lactation elevated 1 | 1.53 | 0.000144 |
| Ankrd13c | 433667 | ankyrin repeat domain 13c | 1.4 | 0.000144 |
| Rab38 | 72433 | RAB38, member of RAS oncogene family | 1.92 | 0.000147 |
| Slc40a1 | 53945 | solute carrier family 40 (iron-regulated transporter), member 1 | 1.73 | 0.000156 |
| Ezh1 | 14055 | enhancer of zeste homolog 1 (Drosophila) | 1.78 | 0.00016 |
| Syt13 | 80976 | synaptotagmin XIII | 1.48 | 0.000161 |
| Ddhd1 | 114874 | DDHD domain containing 1 | 1.52 | 0.000163 |
| Itpripl2 | 319622 | inositol 1,4,5-triphosphate receptor interacting protein-like 2 | 1.37 | 0.000163 |
| Reck | 53614 | reversion-inducing-cysteine-rich protein with kazal motifs | 0.55 | 0.000165 |
| Zcchc5 | 213436 | zinc finger, CCHC domain containing 5 | 0.63 | 0.000178 |
| Sema4b | 20352 | sema domain, immunoglobulin domain (Ig), transmembrane domain (TM) and short cytoplasmic domain, (semaphorin) 4B | 1.56 | 0.000185 |
| Pkia | 18767 | protein kinase inhibitor, alpha | 1.96 | 0.000188 |
| Acsbg1 | 94180 | acyl-CoA synthetase bubblegum family member 1 | 1.55 | 0.00019 |
| Tmppe | 1.01E+08 | transmembrane protein with metallophosphoesterase domain | 1.37 | 0.000208 |
| Pcdh7 | 54216 | protocadherin 7 | 1.34 | 0.000227 |
| Myo19 | 66196 | myosin XIX | 1.57 | 0.000228 |
| Gpr137b_ps | 664862 | NA | 1.37 | 0.000228 |
| Cxcl17 | 232983 | chemokine (C-X-C motif) ligand 17 | 0.54 | 0.000242 |
| Il11 | 16156 | interleukin 11 | 0.37 | 0.000257 |
| Sh3bp2 | 24055 | SH3-domain binding protein 2 | 1.74 | 0.000261 |
| Maats1 | 320214 | NA | 1.71 | 0.000269 |
| Col2a1 | 12824 | collagen, type II, alpha 1 | 0.61 | 0.000271 |
| Txnip | 56338 | thioredoxin interacting protein | 1.48 | 0.000271 |
| Podnl1 | 244550 | podocan-like 1 | 0.47 | 0.000275 |
| Rasa2 | 114713 | RAS p21 protein activator 2 | 1.63 | 0.00028 |
| Ficd | 231630 | FIC domain containing | 2.18 | 0.000281 |
| Krt16 | 16666 | keratin 16 | 2.05 | 0.000306 |
| Scara5 | 71145 | scavenger receptor class A, member 5 (putative) | 0.7 | 0.000306 |
| Mfn2 | 170731 | mitofusin 2 | 1.41 | 0.000309 |
| Cxxc4 | 319478 | CXXC finger 4 | 0.74 | 0.000326 |
| Pxk | 218699 | PX domain containing serine/threonine kinase | 1.34 | 0.000333 |
| Ptpn4 | 19258 | protein tyrosine phosphatase, non-receptor type 4 | 1.67 | 0.000338 |
| Pcdhga6 | 93714 | protocadherin gamma subfamily A, 6 | 0.65 | 0.000345 |
| Rhoc | 11853 | ras homolog gene family, member C | 1.54 | 0.000345 |
| Slc19a2 | 116914 | solute carrier family 19 (thiamine transporter), member 2 | 1.39 | 0.000349 |
| Tcp11l2 | 216198 | t-complex 11 (mouse) like 2 | 0.59 | 0.000354 |
| Glt8d2 | 74782 | glycosyltransferase 8 domain containing 2 | 0.69 | 0.000361 |
| Adcy7 | 11513 | adenylate cyclase 7 | 1.35 | 0.000399 |
| Gm19589 | 1.01E+08 | predicted gene, 19589 | 1.59 | 0.000401 |
| E2f1 | 13555 | E2F transcription factor 1 | 1.49 | 0.000402 |
| 3110021N24Rik | 73133 | RIKEN cDNA 3110021N24 gene | 1.57 | 0.000407 |
| Tnfrsf9 | 21942 | tumor necrosis factor receptor superfamily, member 9 | 2.27 | 0.000421 |
| Mrgprf | 211577 | MAS-related GPR, member F | 0.45 | 0.000427 |
| Plekha4 | 69217 | pleckstrin homology domain containing, family A (phosphoinositide binding specific) member 4 | 0.41 | 0.000428 |
| Fgfr3 | 14184 | fibroblast growth factor receptor 3 | 0.57 | 0.000432 |
| Ccbl2 | 229905 | cysteine conjugate-beta lyase 2 | 0.7 | 0.000436 |
| Dab2 | 13132 | disabled 2, mitogen-responsive phosphoprotein | 0.69 | 0.00045 |
| Slc30a1 | 22782 | solute carrier family 30 (zinc transporter), member 1 | 1.44 | 0.000474 |
| Wee1 | 22390 | WEE 1 homolog 1 (S. pombe) | 1.35 | 0.000479 |
| Gdf10 | 14560 | growth differentiation factor 10 | 0.41 | 0.00049 |
| Ssh2 | 237860 | slingshot homolog 2 (Drosophila) | 1.37 | 0.000496 |
| Snx8 | 231834 | sorting nexin 8 | 1.59 | 0.0005 |
| P2ry14 | 140795 | purinergic receptor P2Y, G-protein coupled, 14 | 1.63 | 0.00051 |
| Ereg | 13874 | epiregulin | 1.83 | 0.000539 |
| Slc35b4 | 58246 | solute carrier family 35, member B4 | 1.45 | 0.000545 |
| Kdm5b | 75605 | lysine (K)-specific demethylase 5B | 0.77 | 0.000573 |
| Sfrp2 | 20319 | secreted frizzled-related protein 2 | 0.61 | 0.000583 |
| Ube2j1 | 56228 | ubiquitin-conjugating enzyme E2J 1 | 1.35 | 0.000616 |
| Fjx1 | 14221 | four jointed box 1 (Drosophila) | 2 | 0.000622 |
| Gm5105 | 329763 | predicted gene 5105 | 1.78 | 0.000629 |
| Arhgef18 | 102098 | rho/rac guanine nucleotide exchange factor (GEF) 18 | 1.51 | 0.000634 |
| Mrps6 | 121022 | mitochondrial ribosomal protein S6 | 0.76 | 0.000657 |
| Atxn1 | 20238 | ataxin 1 | 1.33 | 0.000657 |
| Pde8a | 18584 | phosphodiesterase 8A | 1.26 | 0.000661 |
| Map3k5 | 26408 | mitogen-activated protein kinase kinase kinase 5 | 1.32 | 0.000668 |
| Ptger4 | 19219 | prostaglandin E receptor 4 (subtype EP4) | 0.76 | 0.00067 |
| Il18rap | 16174 | interleukin 18 receptor accessory protein | 1.73 | 0.000673 |
| Arl4c | 320982 | ADP-ribosylation factor-like 4C | 1.41 | 0.000677 |
| Fgfbp1 | 14181 | fibroblast growth factor binding protein 1 | 0.75 | 0.000698 |
| Rbp2 | 19660 | retinol binding protein 2, cellular | 0.62 | 0.000701 |
| Pde4a | 18577 | phosphodiesterase 4A, cAMP specific | 0.67 | 0.000743 |
| Fam189a2 | 381217 | family with sequence similarity 189, member A2 | 1.36 | 0.00075 |
| Lmo7 | 380928 | LIM domain only 7 | 0.75 | 0.000764 |
| Fbxo38 | 107035 | F-box protein 38 | 0.74 | 0.000795 |
| Stk40 | 74178 | serine/threonine kinase 40 | 0.75 | 0.000798 |
| Gm19705 | 1.01E+08 | predicted gene, 19705 | 0.59 | 0.000821 |
| Sybu | 319613 | syntabulin (syntaxin-interacting) | 1.73 | 0.000824 |
| Prelp | 116847 | proline arginine-rich end leucine-rich repeat | 0.54 | 0.000831 |
| Agt | 11606 | angiotensinogen (serpin peptidase inhibitor, clade A, member 8) | 0.52 | 0.000831 |
| Gramd3 | 107022 | GRAM domain containing 3 | 0.75 | 0.000833 |
| Crot | 74114 | carnitine O-octanoyltransferase | 1.38 | 0.000841 |
| Mast2 | 17776 | microtubule associated serine/threonine kinase 2 | 1.32 | 0.000861 |
| Wfs1 | 22393 | Wolfram syndrome 1 homolog (human) | 1.35 | 0.000891 |
| Ern1 | 78943 | endoplasmic reticulum (ER) to nucleus signalling 1 | 1.28 | 0.000953 |
| 1500011K16Rik | 67885 | RIKEN cDNA 1500011K16 gene | 1.56 | 0.000953 |
| Smad9 | 55994 | SMAD family member 9 | 0.69 | 0.000974 |
| Ces2g | 72361 | carboxylesterase 2G | 1.4 | 0.000997 |
| Plekhh2 | 213556 | pleckstrin homology domain containing, family H (with MyTH4 domain) member 2 | 0.81 | 0.001018 |
| Nabp1 | 109019 | nucleic acid binding protein 1 | 1.7 | 0.001029 |
| Atxn1l | 52335 | ataxin 1-like | 1.27 | 0.001037 |
| 6030458C11Rik | 77877 | RIKEN cDNA 6030458C11 gene | 1.3 | 0.00104 |
| Clock | 12753 | circadian locomotor output cycles kaput | 1.5 | 0.00106 |
| Tnnt2 | 21956 | troponin T2, cardiac | 1.31 | 0.001065 |
| Stxbp6 | 217517 | syntaxin binding protein 6 (amisyn) | 0.69 | 0.001078 |
| Arhgef10 | 234094 | Rho guanine nucleotide exchange factor (GEF) 10 | 1.55 | 0.001109 |
| Ncmap | 230822 | noncompact myelin associated protein | 0.42 | 0.001151 |
| Cldn1 | 12737 | claudin 1 | 0.62 | 0.001158 |
| Chdh | 218865 | choline dehydrogenase | 0.6 | 0.001166 |
| Evc2 | 68525 | Ellis van Creveld syndrome 2 | 1.53 | 0.00118 |
| Helb | 117599 | helicase (DNA) B | 1.31 | 0.001184 |
| Col6a3 | 12835 | collagen, type VI, alpha 3 | 1.91 | 0.001197 |
| Sik1 | 17691 | salt inducible kinase 1 | 1.28 | 0.001204 |
| Gdnf | 14573 | glial cell line derived neurotrophic factor | 1.57 | 0.001262 |
| Tor1b | 30934 | torsin family 1, member B | 1.31 | 0.00129 |
| Vash2 | 226841 | vasohibin 2 | 1.41 | 0.001308 |
| Sgms2 | 74442 | sphingomyelin synthase 2 | 0.66 | 0.001355 |
| Zfp36l1 | 12192 | zinc finger protein 36, C3H type-like 1 | 0.79 | 0.001358 |
| Clec11a | 20256 | C-type lectin domain family 11, member a | 0.43 | 0.001387 |
| Pla2g6 | 53357 | phospholipase A2, group VI | 1.52 | 0.001393 |
| Ticam1 | 106759 | toll-like receptor adaptor molecule 1 | 1.26 | 0.001396 |
| Nkiras1 | 69721 | NFKB inhibitor interacting Ras-like protein 1 | 1.92 | 0.001396 |
| Tnks1bp1 | 228140 | tankyrase 1 binding protein 1 | 1.4 | 0.001406 |
| Pfkp | 56421 | phosphofructokinase, platelet | 1.27 | 0.001408 |
| Clcc1 | 229725 | chloride channel CLIC-like 1 | 1.29 | 0.001426 |
| Mt2 | 17750 | metallothionein 2 | 1.38 | 0.001427 |
| Rab33b | 19338 | RAB33B, member of RAS oncogene family | 1.35 | 0.001467 |
| Zfyve26 | 211978 | zinc finger, FYVE domain containing 26 | 1.39 | 0.001484 |
| Ank3 | 11735 | ankyrin 3, epithelial | 0.78 | 0.001501 |
| Tbc1d20 | 67231 | TBC1 domain family, member 20 | 1.45 | 0.001511 |
| Daglb | 231871 | diacylglycerol lipase, beta | 1.42 | 0.001561 |
| Dcaf8 | 98193 | DDB1 and CUL4 associated factor 8 | 1.22 | 0.001565 |
| Rhou | 69581 | ras homolog gene family, member U | 0.81 | 0.001572 |
| Jarid2 | 16468 | jumonji, AT rich interactive domain 2 | 0.75 | 0.001575 |
| Sumf1 | 58911 | sulfatase modifying factor 1 | 1.25 | 0.001577 |
| Tnfaip1 | 21927 | tumor necrosis factor, alpha-induced protein 1 (endothelial) | 1.24 | 0.001604 |
| Hn1 | 15374 | hematological and neurological expressed sequence 1 | 1.32 | 0.001614 |
| Gnb5 | 14697 | guanine nucleotide binding protein (G protein), beta 5 | 1.39 | 0.001627 |
| 5430437J10Rik | 71432 | RIKEN cDNA 5430437J10 gene | 0.45 | 0.001638 |
| Plcxd1 | 403178 | phosphatidylinositol-specific phospholipase C, X domain containing 1 | 1.45 | 0.001711 |
| Trib2 | 217410 | tribbles homolog 2 (Drosophila) | 0.75 | 0.001736 |
| Camk2n2 | 73047 | calcium/calmodulin-dependent protein kinase II inhibitor 2 | 1.48 | 0.001743 |
| Rcn1 | 19672 | reticulocalbin 1 | 0.82 | 0.001744 |
| Map3k3 | 26406 | mitogen-activated protein kinase kinase kinase 3 | 1.22 | 0.001768 |
| Fam167a | 219148 | family with sequence similarity 167, member A | 0.81 | 0.001782 |
| Btrc | 12234 | beta-transducin repeat containing protein | 0.76 | 0.001809 |
| H2_T23 | 15040 | NA | 1.36 | 0.001816 |
| Ccl7 | 20306 | chemokine (C-C motif) ligand 7 | 1.49 | 0.001817 |
| Il1rl1 | 17082 | interleukin 1 receptor-like 1 | 1.27 | 0.001853 |
| Dync2li1 | 213575 | dynein cytoplasmic 2 light intermediate chain 1 | 1.34 | 0.001858 |
| Plin2 | 11520 | perilipin 2 | 1.21 | 0.001859 |
| 9030617O03Rik | 217830 | RIKEN cDNA 9030617O03 gene | 1.3 | 0.001871 |
| Csf1 | 12977 | colony stimulating factor 1 (macrophage) | 1.24 | 0.001872 |
| Blk | 12143 | B lymphoid kinase | 0.7 | 0.001887 |
| Col6a1 | 12833 | collagen, type VI, alpha 1 | 0.74 | 0.001898 |
| Fgf13 | 14168 | fibroblast growth factor 13 | 0.79 | 0.00191 |
| Ly6c1 | 17067 | lymphocyte antigen 6 complex, locus C1 | 0.77 | 0.001918 |
| Wdr31 | 71354 | WD repeat domain 31 | 1.41 | 0.001935 |
| Pea15a | 18611 | phosphoprotein enriched in astrocytes 15A | 1.23 | 0.00194 |
| Zfp568 | 243905 | zinc finger protein 568 | 0.81 | 0.00197 |
| Macrod2 | 72899 | MACRO domain containing 2 | 1.73 | 0.001981 |
| Calcoco1 | 67488 | calcium binding and coiled coil domain 1 | 0.74 | 0.001995 |
| Atg2b | 76559 | autophagy related 2B | 1.24 | 0.001997 |
| Prokr1 | 58182 | prokineticin receptor 1 | 1.44 | 0.002017 |
| Parvb | 170736 | parvin, beta | 0.79 | 0.002019 |
| Adck3 | 67426 | aarF domain containing kinase 3 | 1.61 | 0.002023 |
| Gm5886 | 545886 | predicted gene 5886 | 0.6 | 0.002033 |
| Sord | 20322 | sorbitol dehydrogenase | 0.79 | 0.002037 |
| Cyb561a3 | 225912 | NA | 1.36 | 0.002038 |
| Ankib1 | 70797 | ankyrin repeat and IBR domain containing 1 | 1.28 | 0.002056 |
| Hp | 15439 | haptoglobin | 0.72 | 0.002063 |
| Chka | 12660 | choline kinase alpha | 0.79 | 0.002091 |
| Fbxl5 | 242960 | F-box and leucine-rich repeat protein 5 | 1.42 | 0.002094 |
| Zfp704 | 170753 | zinc finger protein 704 | 0.74 | 0.002109 |
| Mapre3 | 100732 | microtubule-associated protein, RP/EB family, member 3 | 1.48 | 0.00213 |
| Tnfrsf22 | 79202 | tumor necrosis factor receptor superfamily, member 22 | 1.3 | 0.002166 |
| Rab11fip5 | 52055 | RAB11 family interacting protein 5 (class I) | 1.35 | 0.002167 |
| Sec14l2 | 67815 | SEC14-like 2 (S. cerevisiae) | 0.69 | 0.002185 |
| Slc25a44 | 229517 | solute carrier family 25, member 44 | 1.23 | 0.002189 |
| Serpine1 | 18787 | serine (or cysteine) peptidase inhibitor, clade E, member 1 | 1.22 | 0.002194 |
| Kcnip3 | 56461 | Kv channel interacting protein 3, calsenilin | 0.73 | 0.002225 |
| Gramd2 | 546134 | GRAM domain containing 2 | 1.34 | 0.002235 |
| Tmem253 | 619301 | NA | 2.26 | 0.002254 |
| Slc16a6 | 104681 | solute carrier family 16 (monocarboxylic acid transporters), member 6 | 1.3 | 0.00228 |
| Tsr1 | 104662 | TSR1, 20S rRNA accumulation, homolog (yeast) | 1.3 | 0.002297 |
| Bach1 | 12013 | BTB and CNC homology 1 | 0.81 | 0.002321 |
| Dcbld1 | 66686 | discoidin, CUB and LCCL domain containing 1 | 0.77 | 0.002365 |
| Apcdd1 | 494504 | adenomatosis polyposis coli down-regulated 1 | 1.29 | 0.002399 |
| Kif23 | 71819 | kinesin family member 23 | 1.24 | 0.002408 |
| Acta2 | 11475 | actin, alpha 2, smooth muscle, aorta | 0.76 | 0.00242 |
| Tusc2 | 80385 | tumor suppressor candidate 2 | 1.45 | 0.002443 |
| Trip10 | 106628 | thyroid hormone receptor interactor 10 | 1.36 | 0.002446 |
| Rab22a | 19334 | RAB22A, member RAS oncogene family | 1.23 | 0.002465 |
| Pmepa1 | 65112 | prostate transmembrane protein, androgen induced 1 | 1.34 | 0.00249 |
| Fam57a | 116972 | family with sequence similarity 57, member A | 1.28 | 0.002512 |
| Kif7 | 16576 | kinesin family member 7 | 0.76 | 0.002518 |
| Scn1b | 20266 | sodium channel, voltage-gated, type I, beta | 0.44 | 0.002533 |
| Bet1l | 54399 | blocked early in transport 1 homolog (S. cerevisiae)-like | 1.31 | 0.002536 |
| Insig1 | 231070 | insulin induced gene 1 | 0.8 | 0.002556 |
| Fgfr2 | 14183 | fibroblast growth factor receptor 2 | 0.82 | 0.002566 |
| Ppm1k | 243382 | protein phosphatase 1K (PP2C domain containing) | 0.62 | 0.002568 |
| Hmga2_ps1 | 15365 | NA | 1.25 | 0.00257 |
| Cep55 | 74107 | centrosomal protein 55 | 1.33 | 0.002577 |
| Ankrd23 | 78321 | ankyrin repeat domain 23 | 1.41 | 0.00262 |
| Cyp39a1 | 56050 | cytochrome P450, family 39, subfamily a, polypeptide 1 | 1.42 | 0.002621 |
| Tmem64 | 100201 | transmembrane protein 64 | 1.33 | 0.002629 |
| Angptl4 | 57875 | angiopoietin-like 4 | 1.33 | 0.002671 |
| Mbp | 17196 | myelin basic protein | 1.3 | 0.002686 |
| Tbc1d8b | 245638 | TBC1 domain family, member 8B | 1.3 | 0.002692 |
| Sim2 | 20465 | single-minded homolog 2 (Drosophila) | 1.34 | 0.002709 |
| Rps6ka4 | 56613 | ribosomal protein S6 kinase, polypeptide 4 | 1.42 | 0.002721 |
| Slc41a2 | 338365 | solute carrier family 41, member 2 | 1.33 | 0.002733 |
| Aknad1 | 329738 | AKNA domain containing 1 | 0.46 | 0.002754 |
| Mfap3l | 71306 | microfibrillar-associated protein 3-like | 3.31 | 0.0028 |
| Limd2 | 67803 | LIM domain containing 2 | 0.79 | 0.002822 |
| Sema3b | 20347 | sema domain, immunoglobulin domain (Ig), short basic domain, secreted, (semaphorin) 3B | 0.69 | 0.002827 |
| Arhgap44 | 216831 | Rho GTPase activating protein 44 | 1.33 | 0.002884 |
| D430020J02Rik | 319545 | RIKEN cDNA D430020J02 gene | 0.7 | 0.002888 |
| Zfp78 | 330463 | zinc finger protein 78 | 0.63 | 0.002892 |
| Tmem8 | 60455 | transmembrane protein 8 (five membrane-spanning domains) | 1.49 | 0.002896 |
| Ldlrap1 | 100017 | low density lipoprotein receptor adaptor protein 1 | 1.44 | 0.002909 |
| Adamtsl2 | 77794 | ADAMTS-like 2 | 1.27 | 0.002931 |
| Btg2 | 12227 | B cell translocation gene 2, anti-proliferative | 0.78 | 0.002935 |
| Nfatc2 | 18019 | nuclear factor of activated T cells, cytoplasmic, calcineurin dependent 2 | 1.37 | 0.00296 |
| Kif21b | 16565 | kinesin family member 21B | 0.82 | 0.002966 |
| Jhdm1d | NA | jumonji C domain-containing histone demethylase 1 homolog D (S. cerevisiae) | 0.68 | 0.002989 |
| Tada2b | 231151 | transcriptional adaptor 2B | 1.26 | 0.003056 |
| Nckap5l | 380969 | NCK-associated protein 5-like | 0.65 | 0.003058 |
| Cast | 12380 | calpastatin | 1.33 | 0.003061 |
| Map3k12 | 26404 | mitogen-activated protein kinase kinase kinase 12 | 1.29 | 0.003094 |
| Mfsd2b | 432628 | major facilitator superfamily domain containing 2B | 0.47 | 0.003161 |
| Fbxl3 | 50789 | F-box and leucine-rich repeat protein 3 | 1.35 | 0.003163 |
| Gm3776 | 1E+08 | predicted gene 3776 | 1.78 | 0.003164 |
| Rasl11a | 68895 | RAS-like, family 11, member A | 0.8 | 0.003169 |
| E2f5 | 13559 | E2F transcription factor 5 | 1.24 | 0.00318 |
| Slc25a25 | 227731 | solute carrier family 25 (mitochondrial carrier, phosphate carrier), member 25 | 1.21 | 0.003218 |
| Cldn2 | 12738 | claudin 2 | 0.79 | 0.003227 |
| Pmm2 | 54128 | phosphomannomutase 2 | 1.2 | 0.003228 |
| Itga11 | 319480 | integrin alpha 11 | 1.79 | 0.00323 |
| Bach2 | 12014 | BTB and CNC homology 2 | 0.75 | 0.003238 |
| Agbl3 | 76223 | ATP/GTP binding protein-like 3 | 1.23 | 0.00329 |
| Tead3 | 21678 | TEA domain family member 3 | 0.66 | 0.003294 |
| Gosr1 | 53334 | golgi SNAP receptor complex member 1 | 1.19 | 0.003296 |
| Dock8 | 76088 | dedicator of cytokinesis 8 | 1.29 | 0.003344 |
| Clmn | 94040 | calmin | 0.82 | 0.003361 |
| Fam211b | 192734 | NA | 0.82 | 0.003367 |
| Fstl1 | 14314 | follistatin-like 1 | 0.82 | 0.003368 |
| Ednra | 13617 | endothelin receptor type A | 0.71 | 0.003388 |
| Atxn7l3b | 382423 | ataxin 7-like 3B | 0.81 | 0.003431 |
| Wdr78 | 242584 | WD repeat domain 78 | 1.5 | 0.003463 |
| Ankrd9 | 74251 | ankyrin repeat domain 9 | 1.8 | 0.003508 |
| Pex19 | 19298 | peroxisomal biogenesis factor 19 | 0.85 | 0.003531 |
| Tnnc1 | 21924 | troponin C, cardiac/slow skeletal | 1.84 | 0.003562 |
| Prss23 | 76453 | protease, serine, 23 | 0.83 | 0.003571 |
| Slc25a23 | 66972 | solute carrier family 25 (mitochondrial carrier; phosphate carrier), member 23 | 0.74 | 0.003573 |
| Lbh | 77889 | limb-bud and heart | 0.8 | 0.003599 |
| Cyp1a1 | 13076 | cytochrome P450, family 1, subfamily a, polypeptide 1 | 0.75 | 0.003605 |
| Cd163 | 93671 | CD163 antigen | 0.57 | 0.003616 |
| Fanci | 208836 | Fanconi anemia, complementation group I | 1.23 | 0.00368 |
| Zfp651 | 270210 | zinc finger protein 651 | 1.68 | 0.003691 |
| Dusp8 | 18218 | dual specificity phosphatase 8 | 1.34 | 0.003693 |
| Fbxl2 | 72179 | F-box and leucine-rich repeat protein 2 | 1.27 | 0.00373 |
| D5Ertd605e | 1E+08 | DNA segment, Chr 5, ERATO Doi 605, expressed | 0.66 | 0.003736 |
| Lamb3 | 16780 | laminin, beta 3 | 1.3 | 0.003739 |
| Nr4a1 | 15370 | nuclear receptor subfamily 4, group A, member 1 | 0.77 | 0.003765 |
| Serpina3h | 546546 | serine (or cysteine) peptidase inhibitor, clade A, member 3H | 0.71 | 0.003788 |
| Meis2 | 17536 | Meis homeobox 2 | 0.81 | 0.003798 |
| Runx1 | 12394 | runt related transcription factor 1 | 1.23 | 0.003832 |
| Zfp846 | 244721 | zinc finger protein 846 | 0.77 | 0.003874 |
| Ckb | 12709 | creatine kinase, brain | 0.73 | 0.003926 |
| Lrrc14b | 432779 | leucine rich repeat containing 14B | 0.73 | 0.003953 |
| Zfp354c | 30944 | zinc finger protein 354C | 0.68 | 0.003957 |
| Hid1 | 217310 | NA | 0.74 | 0.003976 |
| Uchl1 | 22223 | ubiquitin carboxy-terminal hydrolase L1 | 1.75 | 0.00399 |
| Tmub1 | 64295 | transmembrane and ubiquitin-like domain containing 1 | 1.46 | 0.004042 |
| Cep120 | 225523 | centrosomal protein 120 | 1.28 | 0.004044 |
| Fam160b2 | 239170 | family with sequence similarity 160, member B2 | 1.25 | 0.004046 |
| Tspan13 | 66109 | tetraspanin 13 | 0.79 | 0.00405 |
| Phldb2 | 208177 | pleckstrin homology-like domain, family B, member 2 | 0.83 | 0.004067 |
| Otogl | 628870 | otogelin-like | 0.46 | 0.004105 |
| Dusp2 | 13537 | dual specificity phosphatase 2 | 1.41 | 0.00413 |
| Wnt7b | 22422 | wingless-related MMTV integration site 7B | 1.78 | 0.004212 |
| Vps4a | 116733 | vacuolar protein sorting 4a (yeast) | 0.85 | 0.004247 |
| Zc2hc1c | 72350 | NA | 0.77 | 0.004308 |
| Akna | 100182 | AT-hook transcription factor | 1.38 | 0.004333 |
| Thbs1 | 21825 | thrombospondin 1 | 0.84 | 0.004367 |
| Sgpl1 | 20397 | sphingosine phosphate lyase 1 | 1.18 | 0.004382 |
| Hmgcs1 | 208715 | 3-hydroxy-3-methylglutaryl-Coenzyme A synthase 1 | 0.76 | 0.004388 |
| St6galnac6 | 50935 | ST6 (alpha-N-acetyl-neuraminyl-2,3-beta-galactosyl-1,3)-N-acetylgalactosaminide alpha-2,6-sialyltransferase 6 | 1.32 | 0.004396 |
| Nsdhl | 18194 | NAD(P) dependent steroid dehydrogenase-like | 0.83 | 0.004461 |
| Ttc28 | 209683 | tetratricopeptide repeat domain 28 | 0.8 | 0.004565 |
| Fam109a | 231717 | family with sequence similarity 109, member A | 0.6 | 0.004583 |
| Hist1h4k | 319160 | histone cluster 1, H4k | 0.59 | 0.004611 |
| Rmnd5a | 68477 | required for meiotic nuclear division 5 homolog A (S. cerevisiae) | 0.8 | 0.004623 |
| Snx16 | 74718 | sorting nexin 16 | 1.47 | 0.004628 |
| Elovl5 | 68801 | ELOVL family member 5, elongation of long chain fatty acids (yeast) | 0.79 | 0.004641 |
| Pacs1 | 107975 | phosphofurin acidic cluster sorting protein 1 | 1.28 | 0.004648 |
| Pvr | 52118 | poliovirus receptor | 1.29 | 0.004688 |
| Tmem140 | 68487 | transmembrane protein 140 | 1.34 | 0.004688 |
| Tgoln1 | 22134 | trans-golgi network protein | 1.26 | 0.0047 |
| Sgms1 | 208449 | sphingomyelin synthase 1 | 1.22 | 0.00475 |
| Bgn | 12111 | biglycan | 0.78 | 0.004762 |
| Tsc22d2 | 72033 | TSC22 domain family, member 2 | 0.83 | 0.00479 |
| Mdp1 | 67881 | magnesium-dependent phosphatase 1 | 0.75 | 0.0048 |
| Zfp362 | 230761 | zinc finger protein 362 | 0.85 | 0.004825 |
| Spsb1 | 74646 | splA/ryanodine receptor domain and SOCS box containing 1 | 1.3 | 0.00483 |
| Atp6v0a1 | 11975 | ATPase, H+ transporting, lysosomal V0 subunit A1 | 0.81 | 0.004923 |
| Slc12a6 | 107723 | solute carrier family 12, member 6 | 1.22 | 0.004954 |
| Pnrc1 | 108767 | proline-rich nuclear receptor coactivator 1 | 0.78 | 0.004963 |
| Klf7 | 93691 | Kruppel-like factor 7 (ubiquitous) | 1.3 | 0.005063 |
| Htra1 | 56213 | HtrA serine peptidase 1 | 1.34 | 0.00514 |
| Cxcl5 | 20311 | chemokine (C-X-C motif) ligand 5 | 0.56 | 0.005154 |
| Klhl28 | 66689 | kelch-like 28 (Drosophila) | 1.36 | 0.005167 |
| Epcam | 17075 | epithelial cell adhesion molecule | 1.46 | 0.005175 |
| Jak1 | 16451 | Janus kinase 1 | 1.18 | 0.005197 |
| Il6st | 16195 | interleukin 6 signal transducer | 1.18 | 0.005213 |
| Vldlr | 22359 | very low density lipoprotein receptor | 1.35 | 0.005251 |
| Drp2 | 13497 | dystrophin related protein 2 | 0.48 | 0.005254 |
| Pak6 | 214230 | p21 protein (Cdc42/Rac)-activated kinase 6 | 1.28 | 0.005301 |
| Ndel1 | 83431 | nuclear distribution gene E-like homolog 1 (A. nidulans) | 1.18 | 0.005311 |
| Tnip2 | 231130 | TNFAIP3 interacting protein 2 | 1.24 | 0.005341 |
| Slc25a12 | 78830 | solute carrier family 25 (mitochondrial carrier, Aralar), member 12 | 1.2 | 0.005343 |
| Hspbap1 | 66667 | Hspb associated protein 1 | 1.2 | 0.005375 |
| Rgmb | 68799 | RGM domain family, member B | 1.56 | 0.005377 |
| Zbtb7a | 16969 | zinc finger and BTB domain containing 7a | 1.4 | 0.005439 |
| Stradb | 227154 | STE20-related kinase adaptor beta | 0.86 | 0.00547 |
| Bicd2 | 76895 | bicaudal D homolog 2 (Drosophila) | 1.19 | 0.005475 |
| Cecr2 | 330409 | cat eye syndrome chromosome region, candidate 2 | 0.77 | 0.005484 |
| Hsd11b2 | 15484 | hydroxysteroid 11-beta dehydrogenase 2 | 2.67 | 0.005569 |
| Dlc1 | 50768 | deleted in liver cancer 1 | 0.82 | 0.005583 |
| Tagln | 21345 | transgelin | 0.66 | 0.005634 |
| Glipr1 | 73690 | GLI pathogenesis-related 1 (glioma) | 1.25 | 0.005646 |
| Fblim1 | 74202 | filamin binding LIM protein 1 | 1.21 | 0.005652 |
| Jag1 | 16449 | jagged 1 | 1.24 | 0.005679 |
| AU021092 | 239691 | expressed sequence AU021092 | 0.64 | 0.005681 |
| Tlr5 | 53791 | toll-like receptor 5 | 1.31 | 0.005721 |
| Kcnq3 | 110862 | potassium voltage-gated channel, subfamily Q, member 3 | 0.71 | 0.005727 |
| Slc39a10 | 227059 | solute carrier family 39 (zinc transporter), member 10 | 0.78 | 0.005824 |
| Cpd | 12874 | carboxypeptidase D | 0.82 | 0.005844 |
| Trp53bp2 | 209456 | transformation related protein 53 binding protein 2 | 0.82 | 0.005861 |
| Foxc1 | 17300 | forkhead box C1 | 0.78 | 0.005904 |
| Laptm4a | 17775 | lysosomal-associated protein transmembrane 4A | 1.21 | 0.005932 |
| Atp1a2 | 98660 | ATPase, Na+/K+ transporting, alpha 2 polypeptide | 1.56 | 0.005942 |
| Cers6 | 241447 | NA | 1.16 | 0.006025 |
| Mgmt | 17314 | O-6-methylguanine-DNA methyltransferase | 0.79 | 0.00611 |
| Rab3gap2 | 98732 | RAB3 GTPase activating protein subunit 2 | 1.16 | 0.006178 |
| Qrfp | 227717 | pyroglutamylated RFamide peptide | 0.62 | 0.006202 |
| Pigb | 55981 | phosphatidylinositol glycan anchor biosynthesis, class B | 1.26 | 0.006231 |
| Otub2 | 68149 | OTU domain, ubiquitin aldehyde binding 2 | 0.8 | 0.00627 |
| Epha4 | 13838 | Eph receptor A4 | 1.79 | 0.006293 |
| Twf1 | 19230 | twinfilin, actin-binding protein, homolog 1 (Drosophila) | 1.23 | 0.006353 |
| Mier2 | 70427 | mesoderm induction early response 1, family member 2 | 1.43 | 0.006393 |
| Ubap1 | 67123 | ubiquitin-associated protein 1 | 1.18 | 0.006393 |
| Fam134a | 227298 | family with sequence similarity 134, member A | 1.36 | 0.006414 |
| Zfp964 | 636741 | zinc finger protein 964 | 0.68 | 0.006425 |
| Slc12a7 | 20499 | solute carrier family 12, member 7 | 1.23 | 0.006432 |
| Tbc1d9 | 71310 | TBC1 domain family, member 9 | 1.39 | 0.006502 |
| Serpina3g | 20715 | serine (or cysteine) peptidase inhibitor, clade A, member 3G | 0.56 | 0.006505 |
| Taok2 | 381921 | TAO kinase 2 | 1.29 | 0.006507 |
| Maml2 | 270118 | mastermind like 2 (Drosophila) | 0.81 | 0.00652 |
| Pspc1 | 66645 | paraspeckle protein 1 | 1.18 | 0.006678 |
| 3010026O09Rik | 68067 | RIKEN cDNA 3010026O09 gene | 1.39 | 0.006697 |
| Tfcp2l1 | 81879 | transcription factor CP2-like 1 | 0.77 | 0.006715 |
| AI414108 | 102623 | expressed sequence AI414108 | 1.51 | 0.006715 |
| Taf9b | 407786 | TAF9B RNA polymerase II, TATA box binding protein (TBP)-associated factor | 1.58 | 0.006718 |
| Hist1h2be | 319179 | histone cluster 1, H2be | 1.34 | 0.00673 |
| Fmod | 14264 | fibromodulin | 0.75 | 0.006754 |
| Pigv | 230801 | phosphatidylinositol glycan anchor biosynthesis, class V | 1.41 | 0.006757 |
| Ptbp2 | 56195 | polypyrimidine tract binding protein 2 | 0.76 | 0.006789 |
| Efna3 | 13638 | ephrin A3 | 1.54 | 0.006836 |
| Ccl25 | 20300 | chemokine (C-C motif) ligand 25 | 0.75 | 0.00684 |
| Tmem38b | 52076 | transmembrane protein 38B | 1.2 | 0.006858 |
| Frmd4a | 209630 | FERM domain containing 4A | 1.17 | 0.006878 |
| Inpp4b | 234515 | inositol polyphosphate-4-phosphatase, type II | 1.39 | 0.006896 |
| Hipk3 | 15259 | homeodomain interacting protein kinase 3 | 0.84 | 0.006944 |
| Ulk3 | 71742 | unc-51-like kinase 3 | 1.19 | 0.006958 |
| Ifitm1 | 68713 | interferon induced transmembrane protein 1 | 0.86 | 0.007013 |
| Edn2 | 13615 | endothelin 2 | 0.54 | 0.00702 |
| Lipe | 16890 | lipase, hormone sensitive | 0.66 | 0.007026 |
| Mkx | 210719 | mohawk homeobox | 1.22 | 0.007058 |
| Arhgap1 | 228359 | Rho GTPase activating protein 1 | 1.2 | 0.007058 |
| Radil | 231858 | Ras association and DIL domains | 1.81 | 0.007079 |
| Arv1 | 68865 | ARV1 homolog (yeast) | 1.33 | 0.007101 |
| Esx1 | 13984 | extraembryonic, spermatogenesis, homeobox 1 | 1.38 | 0.007146 |
| Coq10b | 67876 | coenzyme Q10 homolog B (S. cerevisiae) | 1.29 | 0.007195 |
| D4Wsu53e | 27981 | DNA segment, Chr 4, Wayne State University 53, expressed | 1.27 | 0.007211 |
| Gpr39 | 71111 | G protein-coupled receptor 39 | 1.4 | 0.007215 |
| Pdxk | 216134 | pyridoxal (pyridoxine, vitamin B6) kinase | 0.85 | 0.007235 |
| Tmem127 | 69470 | transmembrane protein 127 | 1.23 | 0.007249 |
| Trappc2 | 66226 | trafficking protein particle complex 2 | 1.35 | 0.007268 |
| 9130008F23Rik | 71583 | RIKEN cDNA 9130008F23 gene | 0.76 | 0.007287 |
| Sdhc | 66052 | succinate dehydrogenase complex, subunit C, integral membrane protein | 0.86 | 0.007364 |
| Plagl1 | 22634 | pleiomorphic adenoma gene-like 1 | 0.72 | 0.007374 |
| Stk11 | 20869 | serine/threonine kinase 11 | 1.31 | 0.007392 |
| Tuba4a | 22145 | tubulin, alpha 4A | 1.19 | 0.00744 |
| A830010M20Rik | 231570 | RIKEN cDNA A830010M20 gene | 1.39 | 0.007441 |
| M6pr | 17113 | mannose-6-phosphate receptor, cation dependent | 1.21 | 0.007443 |
| Tmem150a | 232086 | transmembrane protein 150A | 0.74 | 0.007444 |
| Fem1c | 240263 | fem-1 homolog c (C.elegans) | 1.18 | 0.007488 |
| Rnf113a2 | 66381 | ring finger protein 113A2 | 0.85 | 0.007576 |
| Saysd1 | 67509 | NA | 0.84 | 0.007632 |
| Fam136a | 66488 | family with sequence similarity 136, member A | 0.86 | 0.007652 |
| Elf3 | 13710 | E74-like factor 3 | 0.68 | 0.007853 |
| Sqle | 20775 | squalene epoxidase | 0.86 | 0.007869 |
| Kctd7 | 212919 | potassium channel tetramerisation domain containing 7 | 1.26 | 0.007892 |
| Gk5 | 235533 | glycerol kinase 5 (putative) | 0.77 | 0.007919 |
| Fcrlb | 435653 | Fc receptor-like B | 2.49 | 0.007975 |
| Prg4 | 96875 | proteoglycan 4 (megakaryocyte stimulating factor, articular superficial zone protein) | 0.72 | 0.008003 |
| Dpp3 | 75221 | dipeptidylpeptidase 3 | 1.29 | 0.008034 |
| Palm3 | 74337 | paralemmin 3 | 0.43 | 0.008097 |
| Rnf6 | 74132 | ring finger protein (C3H2C3 type) 6 | 1.19 | 0.008118 |
| Lctl | 235435 | lactase-like | 1.3 | 0.008124 |
| Pigp | 56176 | phosphatidylinositol glycan anchor biosynthesis, class P | 0.79 | 0.008201 |
| Itgb7 | 16421 | integrin beta 7 | 1.31 | 0.008249 |
| 2010012O05Rik | 66439 | RIKEN cDNA 2010012O05 gene | 0.77 | 0.008286 |
| Kat2b | 18519 | K(lysine) acetyltransferase 2B | 1.36 | 0.00837 |
| Hopx | 74318 | HOP homeobox | 1.44 | 0.008387 |
| Ifngr1 | 15979 | interferon gamma receptor 1 | 1.18 | 0.008413 |
| Ppargc1a | 19017 | peroxisome proliferative activated receptor, gamma, coactivator 1 alpha | 0.38 | 0.008488 |
| Krt13 | 16663 | keratin 13 | 0.81 | 0.008501 |
| Cgnl1 | 68178 | cingulin-like 1 | 0.87 | 0.008509 |
| Ccnd1 | 12443 | cyclin D1 | 1.16 | 0.008644 |
| Col8a2 | 329941 | collagen, type VIII, alpha 2 | 0.65 | 0.008649 |
| Cmtr2 | 234728 | NA | 1.23 | 0.008723 |
| Slc16a2 | 20502 | solute carrier family 16 (monocarboxylic acid transporters), member 2 | 0.79 | 0.00873 |
| Ube2l6 | 56791 | ubiquitin-conjugating enzyme E2L 6 | 1.36 | 0.008752 |
| Chek2 | 50883 | checkpoint kinase 2 | 1.32 | 0.00885 |
| Zfp169 | 67911 | zinc finger protein 169 | 1.28 | 0.008927 |
| Prdm11 | 1E+08 | PR domain containing 11 | 1.28 | 0.008965 |
| Vti1a | 53611 | vesicle transport through interaction with t-SNAREs homolog 1A (yeast) | 0.82 | 0.009044 |
| Atm | 11920 | ataxia telangiectasia mutated homolog (human) | 1.26 | 0.009048 |
| AI450353 | 103729 | expressed sequence AI450353 | 1.45 | 0.009079 |
| 3110043O21Rik | 73205 | RIKEN cDNA 3110043O21 gene | 0.86 | 0.009084 |
| Dnajc22 | 72778 | DnaJ (Hsp40) homolog, subfamily C, member 22 | 0.84 | 0.009166 |
| Swsap1 | 66962 | NA | 0.77 | 0.009245 |
| Pik3r1 | 18708 | phosphatidylinositol 3-kinase, regulatory subunit, polypeptide 1 (p85 alpha) | 0.83 | 0.009259 |
| Kcnj2 | 16518 | potassium inwardly-rectifying channel, subfamily J, member 2 | 0.71 | 0.009268 |
| Map3k2 | 26405 | mitogen-activated protein kinase kinase kinase 2 | 1.27 | 0.009281 |
| Pdgfrl | 68797 | platelet-derived growth factor receptor-like | 0.49 | 0.009293 |
| C130046K22Rik | 399609 | RIKEN cDNA C130046K22 gene | 0.74 | 0.009297 |
| Renbp | 19703 | renin binding protein | 0.84 | 0.009299 |
| Zfp661 | 72180 | zinc finger protein 661 | 0.81 | 0.009329 |
| Mettl25 | 216292 | NA | 1.15 | 0.009369 |
| Smad7 | 17131 | SMAD family member 7 | 1.27 | 0.009467 |
| Rab30 | 75985 | RAB30, member RAS oncogene family | 1.32 | 0.009567 |
| Hmox2 | 15369 | heme oxygenase (decycling) 2 | 1.16 | 0.009636 |
| Hey1 | 15213 | hairy/enhancer-of-split related with YRPW motif 1 | 0.75 | 0.009643 |
| Phlpp2 | 244650 | PH domain and leucine rich repeat protein phosphatase 2 | 1.22 | 0.009671 |
| Arhgap19 | 71085 | Rho GTPase activating protein 19 | 0.85 | 0.009719 |
| Zdhhc4 | 72881 | zinc finger, DHHC domain containing 4 | 0.8 | 0.009819 |
| Gbf1 | 107338 | golgi-specific brefeldin A-resistance factor 1 | 1.17 | 0.009822 |
| Trp63 | 22061 | transformation related protein 63 | 0.13 | 0.009836 |
| Cwf19l1 | 72502 | CWF19-like 1, cell cycle control (S. pombe) | 1.16 | 0.009842 |
| Rnf19b | 75234 | ring finger protein 19B | 1.22 | 0.009845 |
| Dpp7 | 83768 | dipeptidylpeptidase 7 | 1.15 | 0.009869 |
| Kcnab3 | 16499 | potassium voltage-gated channel, shaker-related subfamily, beta member 3 | 0.71 | 0.009872 |
| Kng1 | 16644 | kininogen 1 | 0.77 | 0.009944 |
| Bckdhb | 12040 | branched chain ketoacid dehydrogenase E1, beta polypeptide | 0.83 | 0.010013 |
| Myh10 | 77579 | myosin, heavy polypeptide 10, non-muscle | 0.86 | 0.010038 |
| Mfsd6 | 98682 | major facilitator superfamily domain containing 6 | 1.54 | 0.010057 |
| Ampd3 | 11717 | adenosine monophosphate deaminase 3 | 1.43 | 0.010201 |
| Mex3a | 72640 | mex3 homolog A (C. elegans) | 0.86 | 0.01026 |
| Tlcd1 | 68385 | TLC domain containing 1 | 0.86 | 0.010278 |
| Slc5a6 | 330064 | solute carrier family 5 (sodium-dependent vitamin transporter), member 6 | 0.82 | 0.010294 |
| Chrm1 | 12669 | cholinergic receptor, muscarinic 1, CNS | 0.5 | 0.010296 |
| Rnf43 | 207742 | ring finger protein 43 | 0.73 | 0.010403 |
| Akr1c18 | 105349 | aldo-keto reductase family 1, member C18 | 0.68 | 0.010452 |
| Zswim3 | 67538 | zinc finger, SWIM domain containing 3 | 1.2 | 0.010474 |
| Zfp954 | 232853 | zinc finger protein 954 | 1.18 | 0.010541 |
| Lhx1os | 78365 | NA | 0.76 | 0.010544 |
| Oxsr1 | 108737 | oxidative-stress responsive 1 | 0.87 | 0.010553 |
| Has1 | 15116 | hyaluronan synthase1 | 1.3 | 0.010639 |
| Mtrr | 210009 | 5-methyltetrahydrofolate-homocysteine methyltransferase reductase | 1.14 | 0.01064 |
| Adcy6 | 11512 | adenylate cyclase 6 | 0.84 | 0.010691 |
| Otud1 | 71198 | OTU domain containing 1 | 1.31 | 0.010774 |
| 4933439C10Rik | 74476 | RIKEN cDNA 4933439C10 gene | 1.55 | 0.010778 |
| Ppp1r3e | 105651 | protein phosphatase 1, regulatory (inhibitor) subunit 3E | 1.7 | 0.010781 |
| Plcg2 | 234779 | phospholipase C, gamma 2 | 1.17 | 0.010855 |
| Serinc5 | 218442 | serine incorporator 5 | 0.8 | 0.010857 |
| Pla2g16 | 225845 | phospholipase A2, group XVI | 1.4 | 0.010902 |
| Fgd6 | 13998 | FYVE, RhoGEF and PH domain containing 6 | 1.19 | 0.010905 |
| Cldn15 | 60363 | claudin 15 | 0.45 | 0.010907 |
| 3-Sep | 24050 | septin 3 | 1.34 | 0.010911 |
| Arf3 | 11842 | ADP-ribosylation factor 3 | 0.86 | 0.010924 |
| Rhobtb2 | 246710 | Rho-related BTB domain containing 2 | 1.23 | 0.010976 |
| Irf1 | 16362 | interferon regulatory factor 1 | 1.17 | 0.010984 |
| Ccdc134 | 76457 | coiled-coil domain containing 134 | 1.15 | 0.011035 |
| Col3a1 | 12825 | collagen, type III, alpha 1 | 1.16 | 0.011065 |
| Cd274 | 60533 | CD274 antigen | 1.39 | 0.011181 |
| Prkaca | 18747 | protein kinase, cAMP dependent, catalytic, alpha | 0.87 | 0.011184 |
| Enpep | 13809 | glutamyl aminopeptidase | 0.69 | 0.011201 |
| 1700088E04Rik | 27660 | RIKEN cDNA 1700088E04 gene | 2.07 | 0.011283 |
| Klf11 | 194655 | Kruppel-like factor 11 | 1.37 | 0.01129 |
| Tgfa | 21802 | transforming growth factor alpha | 1.2 | 0.011293 |
| Chac1 | 69065 | ChaC, cation transport regulator 1 | 0.59 | 0.011295 |
| Dcun1d4 | 100737 | DCN1, defective in cullin neddylation 1, domain containing 4 (S. cerevisiae) | 0.84 | 0.011325 |
| Zfp719 | 210105 | zinc finger protein 719 | 0.81 | 0.011361 |
| Lifr | 16880 | leukemia inhibitory factor receptor | 0.79 | 0.011378 |
| Tnfrsf23 | 79201 | tumor necrosis factor receptor superfamily, member 23 | 1.16 | 0.011406 |
| Mras | 17532 | muscle and microspikes RAS | 0.8 | 0.011452 |
| Cdc25c | 12532 | cell division cycle 25C | 0.85 | 0.01147 |
| Gmip | 78816 | Gem-interacting protein | 1.2 | 0.011476 |
| Speg | 11790 | SPEG complex locus | 0.7 | 0.011514 |
| Prrt2 | 69017 | proline-rich transmembrane protein 2 | 1.47 | 0.01158 |
| Hnrnpdl | 50926 | NA | 0.83 | 0.011607 |
| Bcl9 | 77578 | B cell CLL/lymphoma 9 | 0.83 | 0.011632 |
| Thbs3 | 21827 | thrombospondin 3 | 0.68 | 0.011634 |
| Serpina3i | 628900 | serine (or cysteine) peptidase inhibitor, clade A, member 3I | 0.8 | 0.011714 |
| Aktip | 14339 | thymoma viral proto-oncogene 1 interacting protein | 1.18 | 0.011721 |
| Mmd | 67468 | monocyte to macrophage differentiation-associated | 0.8 | 0.011722 |
| Txnrd2 | 26462 | thioredoxin reductase 2 | 1.27 | 0.011731 |
| Limk1 | 16885 | LIM-domain containing, protein kinase | 1.35 | 0.011784 |
| Cdc16 | 69957 | CDC16 cell division cycle 16 | 0.85 | 0.011798 |
| Tubb3 | 22152 | tubulin, beta 3 class III | 1.33 | 0.011822 |
| Rab11fip4 | 268451 | RAB11 family interacting protein 4 (class II) | 1.26 | 0.011823 |
| Rgs17 | 56533 | regulator of G-protein signaling 17 | 1.62 | 0.011848 |
| Vcam1 | 22329 | vascular cell adhesion molecule 1 | 0.75 | 0.01188 |
| Syap1 | 67043 | synapse associated protein 1 | 1.26 | 0.011932 |
| Pfkfb3 | 170768 | 6-phosphofructo-2-kinase/fructose-2,6-biphosphatase 3 | 1.17 | 0.012178 |
| Tcaim | 382117 | NA | 1.18 | 0.012181 |
| Ddah1 | 69219 | dimethylarginine dimethylaminohydrolase 1 | 1.16 | 0.012196 |
| Blmh | 104184 | bleomycin hydrolase | 0.87 | 0.012266 |
| Raph1 | 77300 | Ras association (RalGDS/AF-6) and pleckstrin homology domains 1 | 0.81 | 0.012304 |
| Ptpru | 19273 | protein tyrosine phosphatase, receptor type, U | 1.56 | 0.012319 |
| Susd5 | 382111 | sushi domain containing 5 | 1.2 | 0.012354 |
| Ica1 | 15893 | islet cell autoantigen 1 | 1.17 | 0.012383 |
| Eml1 | 68519 | echinoderm microtubule associated protein like 1 | 1.2 | 0.012434 |
| Ikbkg | 16151 | inhibitor of kappaB kinase gamma | 1.22 | 0.012471 |
| Nek6 | 59126 | NIMA (never in mitosis gene a)-related expressed kinase 6 | 0.87 | 0.012513 |
| Reep5 | 13476 | receptor accessory protein 5 | 0.88 | 0.01256 |
| Dusp28 | 67446 | dual specificity phosphatase 28 | 1.32 | 0.012568 |
| Gm9958 | 791294 | predicted gene 9958 | 0.59 | 0.012609 |
| Setd1b | 208043 | SET domain containing 1B | 0.81 | 0.012613 |
| Tfb2m | 15278 | transcription factor B2, mitochondrial | 1.21 | 0.012652 |
| Gpsm2 | 76123 | G-protein signalling modulator 2 (AGS3-like, C. elegans) | 0.87 | 0.012715 |
| Slc41a1 | 98396 | solute carrier family 41, member 1 | 1.2 | 0.012744 |
| Dusp18 | 75219 | dual specificity phosphatase 18 | 1.19 | 0.012785 |
| Lpgat1 | 226856 | lysophosphatidylglycerol acyltransferase 1 | 1.21 | 0.012822 |
| Mmp13 | 17386 | matrix metallopeptidase 13 | 0.71 | 0.012846 |
| Rasip1 | 69903 | Ras interacting protein 1 | 1.24 | 0.012894 |
| Tubb2a | 22151 | tubulin, beta 2A class IIA | 1.26 | 0.012908 |
| Id2 | 15902 | inhibitor of DNA binding 2 | 0.74 | 0.012945 |
| Lif | 16878 | leukemia inhibitory factor | 1.37 | 0.012946 |
| Uba7 | 74153 | ubiquitin-like modifier activating enzyme 7 | 1.35 | 0.013019 |
| Mdfic | 16543 | MyoD family inhibitor domain containing | 1.28 | 0.013036 |
| Tram2 | 170829 | translocating chain-associating membrane protein 2 | 0.76 | 0.013064 |
| Gtpbp2 | 56055 | GTP binding protein 2 | 1.15 | 0.013083 |
| Tcam1 | 75870 | testicular cell adhesion molecule 1 | 1.42 | 0.013109 |
| Lrba | 80877 | LPS-responsive beige-like anchor | 0.86 | 0.013134 |
| Rps6ka5 | 73086 | ribosomal protein S6 kinase, polypeptide 5 | 1.27 | 0.013162 |
| Cd300lb | 217304 | CD300 antigen like family member B | 0.78 | 0.01317 |
| Btc | 12223 | betacellulin, epidermal growth factor family member | 0.71 | 0.01317 |
| Iqgap2 | 544963 | IQ motif containing GTPase activating protein 2 | 1.26 | 0.013211 |
| Dip2a | 64451 | DIP2 disco-interacting protein 2 homolog A (Drosophila) | 1.22 | 0.01323 |
| Stk32b | 64293 | serine/threonine kinase 32B | 0.55 | 0.013335 |
| Wnt6 | 22420 | wingless-related MMTV integration site 6 | 0.48 | 0.013581 |
| Eogt | 101351 | EGF domain-specific O-linked N-acetylglucosamine (GlcNAc) transferase | 1.17 | 0.013608 |
| Ufsp1 | 70240 | UFM1-specific peptidase 1 | 0.82 | 0.013619 |
| Samd9l | 209086 | sterile alpha motif domain containing 9-like | 1.26 | 0.013636 |
| Dusp6 | 67603 | dual specificity phosphatase 6 | 0.88 | 0.013639 |
| Padi2 | 18600 | peptidyl arginine deiminase, type II | 1.23 | 0.013645 |
| Dlgap4 | 228836 | discs, large homolog-associated protein 4 (Drosophila) | 0.81 | 0.013647 |
| Bpgm | 12183 | 2,3-bisphosphoglycerate mutase | 1.16 | 0.013654 |
| Afap1l2 | 226250 | actin filament associated protein 1-like 2 | 1.2 | 0.013672 |
| Adamts4 | 240913 | a disintegrin-like and metallopeptidase (reprolysin type) with thrombospondin type 1 motif, 4 | 0.79 | 0.013743 |
| Gm765 | 330390 | predicted gene 765 | 0.51 | 0.013746 |
| Zkscan14 | 67235 | zinc finger with KRAB and SCAN domains 14 | 0.79 | 0.013775 |
| Psmd10 | 53380 | proteasome (prosome, macropain) 26S subunit, non-ATPase, 10 | 0.86 | 0.013809 |
| Dnajc16 | 214063 | DnaJ (Hsp40) homolog, subfamily C, member 16 | 1.24 | 0.013885 |
| Dpt | 56429 | dermatopontin | 1.7 | 0.013885 |
| Prlr | 19116 | prolactin receptor | 0.61 | 0.0139 |
| Tmcc1 | 330401 | transmembrane and coiled coil domains 1 | 1.22 | 0.01394 |
| Pisd_ps1 | 236604 | NA | 1.16 | 0.013947 |
| Cep97 | 74201 | centrosomal protein 97 | 1.28 | 0.01404 |
| Klf10 | 21847 | Kruppel-like factor 10 | 1.22 | 0.014101 |
| Prr14l | 215476 | NA | 1.21 | 0.014112 |
| Zfp39 | 22698 | zinc finger protein 39 | 0.79 | 0.014142 |
| Slc37a2 | 56857 | solute carrier family 37 (glycerol-3-phosphate transporter), member 2 | 0.61 | 0.01416 |
| Masp1 | 17174 | mannan-binding lectin serine peptidase 1 | 0.87 | 0.014225 |
| Egln3 | 112407 | EGL nine homolog 3 (C. elegans) | 1.22 | 0.014318 |
| Zc3hav1l | 209032 | zinc finger CCCH-type, antiviral 1-like | 0.75 | 0.014349 |
| Fabp7 | 12140 | fatty acid binding protein 7, brain | 0.82 | 0.014415 |
| Tmem106c | 380967 | transmembrane protein 106C | 1.15 | 0.014442 |
| Gm17762 | 1E+08 | predicted gene, 17762 | 1.75 | 0.014449 |
| Triobp | 110253 | TRIO and F-actin binding protein | 1.26 | 0.014474 |
| Slc26a6 | 171429 | solute carrier family 26, member 6 | 0.85 | 0.014475 |
| Vangl1 | 229658 | vang-like 1 (van gogh, Drosophila) | 1.23 | 0.014479 |
| Mkrn1 | 54484 | makorin, ring finger protein, 1 | 1.14 | 0.01452 |
| Heg1 | 77446 | HEG homolog 1 (zebrafish) | 1.18 | 0.014643 |
| Il17ra | 16172 | interleukin 17 receptor A | 1.17 | 0.014652 |
| Aifm1 | 26926 | apoptosis-inducing factor, mitochondrion-associated 1 | 1.2 | 0.014701 |
| Ccl2 | 20296 | chemokine (C-C motif) ligand 2 | 1.23 | 0.014774 |
| Ugp2 | 216558 | UDP-glucose pyrophosphorylase 2 | 0.77 | 0.014788 |
| Tnfrsf21 | 94185 | tumor necrosis factor receptor superfamily, member 21 | 1.19 | 0.014808 |
| Vcpkmt | 207965 | NA | 0.85 | 0.014839 |
| Emb | 13723 | embigin | 0.73 | 0.014848 |
| Tbc1d24 | 224617 | TBC1 domain family, member 24 | 0.81 | 0.014852 |
| Tceanc2 | 66526 | NA | 0.83 | 0.014888 |
| 2700054A10Rik | 72578 | RIKEN cDNA 2700054A10 gene | 0.47 | 0.015002 |
| Fam120aos | 68128 | family with sequence similarity 120A opposite strand | 0.75 | 0.015004 |
| Tmem62 | 96957 | transmembrane protein 62 | 0.88 | 0.015043 |
| Mroh6 | 223645 | NA | 0.67 | 0.015046 |
| Mapkapk3 | 102626 | mitogen-activated protein kinase-activated protein kinase 3 | 0.76 | 0.015076 |
| 9530082P21Rik | 638247 | RIKEN cDNA 9530082P21 gene | 0.85 | 0.015111 |
| Ptch1 | 19206 | patched homolog 1 | 0.84 | 0.015169 |
| Dhcr7 | 13360 | 7-dehydrocholesterol reductase | 0.87 | 0.015209 |
| Cntn1 | 12805 | contactin 1 | 0.72 | 0.01529 |
| B3gntl1 | 210004 | UDP-GlcNAc:betaGal beta-1,3-N-acetylglucosaminyltransferase-like 1 | 1.26 | 0.015371 |
| Fat4 | 329628 | FAT tumor suppressor homolog 4 (Drosophila) | 0.79 | 0.015409 |
| Wrb | 71446 | tryptophan rich basic protein | 1.14 | 0.015455 |
| Pan2 | 103135 | PAN2 polyA specific ribonuclease subunit homolog (S. cerevisiae) | 0.84 | 0.015465 |
| Ctnnbip1 | 67087 | catenin beta interacting protein 1 | 0.84 | 0.015468 |
| Emc9 | 85308 | ER membrane protein complex subunit 9 | 1.59 | 0.015484 |
| Sin3b | 20467 | transcriptional regulator, SIN3B (yeast) | 1.17 | 0.015682 |
| Mob1a | 232157 | MOB kinase activator 1A | 1.17 | 0.015696 |
| 8430427H17Rik | 329540 | RIKEN cDNA 8430427H17 gene | 0.87 | 0.015703 |
| Tmc4 | 353499 | transmembrane channel-like gene family 4 | 0.69 | 0.015756 |
| Glb1 | 12091 | galactosidase, beta 1 | 0.84 | 0.015784 |
| Nipal3 | 74552 | NIPA-like domain containing 3 | 0.83 | 0.015842 |
| Tmem53 | 68777 | transmembrane protein 53 | 0.69 | 0.01587 |
| Gsta4 | 14860 | glutathione S-transferase, alpha 4 | 1.18 | 0.015943 |
| Pard6a | 56513 | par-6 (partitioning defective 6,) homolog alpha (C. elegans) | 1.22 | 0.015999 |
| Ankrd49 | 56503 | ankyrin repeat domain 49 | 0.84 | 0.016033 |
| Tvp23a | 383103 | NA | 1.31 | 0.016065 |
| Sc4mol | 66234 | sterol-C4-methyl oxidase-like | 0.8 | 0.016207 |
| Arhgap26 | 71302 | Rho GTPase activating protein 26 | 0.67 | 0.016274 |
| Lypd6b | 71897 | LY6/PLAUR domain containing 6B | 1.92 | 0.016321 |
| Tcf7l1 | 21415 | transcription factor 7 like 1 (T cell specific, HMG box) | 0.74 | 0.016333 |
| Rassf2 | 215653 | Ras association (RalGDS/AF-6) domain family member 2 | 0.78 | 0.016402 |
| Rab4a | 19341 | RAB4A, member RAS oncogene family | 0.74 | 0.01648 |
| Zfp97 | 22759 | zinc finger protein 97 | 1.46 | 0.016567 |
| Zfp518b | 100515 | zinc finger protein 518B | 0.82 | 0.01664 |
| Slc22a15 | 242126 | solute carrier family 22 (organic anion/cation transporter), member 15 | 1.15 | 0.016641 |
| Prl2c2 | 18811 | prolactin family 2, subfamily c, member 2 | 1.52 | 0.016725 |
| Adamts1 | 11504 | a disintegrin-like and metallopeptidase (reprolysin type) with thrombospondin type 1 motif, 1 | 0.77 | 0.016775 |
| Ppip5k1 | 327655 | diphosphoinositol pentakisphosphate kinase 1 | 1.18 | 0.016886 |
| Gm19710 | 1.01E+08 | predicted gene, 19710 | 0.56 | 0.016895 |
| Ergic1 | 67458 | endoplasmic reticulum-golgi intermediate compartment (ERGIC) 1 | 0.86 | 0.016962 |
| Fbxl16 | 214931 | F-box and leucine-rich repeat protein 16 | 0.76 | 0.016967 |
| Armc8 | 74125 | armadillo repeat containing 8 | 1.16 | 0.01702 |
| Ift27 | 67042 | intraflagellar transport 27 | 0.84 | 0.017038 |
| Snrnp35 | 76167 | small nuclear ribonucleoprotein 35 (U11/U12) | 0.82 | 0.017068 |
| Pdss2 | 71365 | prenyl (solanesyl) diphosphate synthase, subunit 2 | 0.87 | 0.017163 |
| Odf3l1 | 382075 | outer dense fiber of sperm tails 3-like 1 | 1.69 | 0.01723 |
| Lgals3 | 16854 | lectin, galactose binding, soluble 3 | 1.18 | 0.017306 |
| Derl2 | 116891 | Der1-like domain family, member 2 | 1.19 | 0.017358 |
| Sfxn3 | 94280 | sideroflexin 3 | 0.88 | 0.017435 |
| Ormdl3 | 66612 | ORM1-like 3 (S. cerevisiae) | 1.19 | 0.017528 |
| Klf13 | 50794 | Kruppel-like factor 13 | 0.85 | 0.0176 |
| Dnajb14 | 70604 | DnaJ (Hsp40) homolog, subfamily B, member 14 | 0.85 | 0.017803 |
| Sigirr | 24058 | single immunoglobulin and toll-interleukin 1 receptor (TIR) domain | 1.18 | 0.01791 |
| Arid5b | 71371 | AT rich interactive domain 5B (MRF1-like) | 0.86 | 0.017916 |
| Plekhm1 | 353047 | pleckstrin homology domain containing, family M (with RUN domain) member 1 | 1.26 | 0.01794 |
| Slc35g1 | 240660 | NA | 1.17 | 0.017972 |
| Snhg7 | 72091 | small nucleolar RNA host gene (non-protein coding) 7 | 0.87 | 0.017987 |
| Ptprn | 19275 | protein tyrosine phosphatase, receptor type, N | 0.62 | 0.017998 |
| Aebp1 | 11568 | AE binding protein 1 | 0.82 | 0.018 |
| Cntrob | 216846 | centrobin, centrosomal BRCA2 interacting protein | 1.18 | 0.018002 |
| Serpina11 | 380780 | serine (or cysteine) peptidase inhibitor, clade A (alpha-1 antiproteinase, antitrypsin), member 11 | 2.06 | 0.018006 |
| Depdc5 | 277854 | DEP domain containing 5 | 0.85 | 0.018133 |
| Angpt2 | 11601 | angiopoietin 2 | 1.25 | 0.018179 |
| Zbtb39 | 320080 | zinc finger and BTB domain containing 39 | 0.84 | 0.01818 |
| Nup35 | 69482 | nucleoporin 35 | 1.2 | 0.01824 |
| Slc39a1 | 30791 | solute carrier family 39 (zinc transporter), member 1 | 0.88 | 0.018355 |
| Tmem171 | 380863 | transmembrane protein 171 | 1.65 | 0.018476 |
| Camk1d | 227541 | calcium/calmodulin-dependent protein kinase ID | 1.17 | 0.018577 |
| Pag1 | 94212 | phosphoprotein associated with glycosphingolipid microdomains 1 | 0.83 | 0.018625 |
| Tdrd7 | 100121 | tudor domain containing 7 | 1.25 | 0.018629 |
| Alg12 | 223774 | asparagine-linked glycosylation 12 (alpha-1,6-mannosyltransferase) | 1.23 | 0.018667 |
| Adamtsl4 | 229595 | ADAMTS-like 4 | 1.42 | 0.018691 |
| Zfp395 | 380912 | zinc finger protein 395 | 0.83 | 0.018693 |
| Hmgcr | 15357 | 3-hydroxy-3-methylglutaryl-Coenzyme A reductase | 0.87 | 0.018698 |
| Prcp | 72461 | prolylcarboxypeptidase (angiotensinase C) | 1.18 | 0.018735 |
| Krt8 | 16691 | keratin 8 | 1.22 | 0.018774 |
| Rbmx2 | 209003 | RNA binding motif protein, X-linked 2 | 0.87 | 0.018799 |
| Fam45a | 67894 | family with sequence similarity 45, member A | 1.23 | 0.0188 |
| Acat1 | 110446 | acetyl-Coenzyme A acetyltransferase 1 | 1.14 | 0.018836 |
| Mccc1 | 72039 | methylcrotonoyl-Coenzyme A carboxylase 1 (alpha) | 0.75 | 0.0189 |
| Eif4ebp2 | 13688 | eukaryotic translation initiation factor 4E binding protein 2 | 0.86 | 0.018986 |
| Gm6251 | 621697 | predicted gene 6251 | 1.42 | 0.019049 |
| Mastl | 67121 | microtubule associated serine/threonine kinase-like | 1.28 | 0.019091 |
| Zfp12 | 231866 | zinc finger protein 12 | 0.88 | 0.019096 |
| Casz1 | 69743 | castor zinc finger 1 | 0.78 | 0.019101 |
| Sqstm1 | 18412 | sequestosome 1 | 1.22 | 0.019191 |
| Fdft1 | 14137 | farnesyl diphosphate farnesyl transferase 1 | 0.88 | 0.019211 |
| Ptcd1 | 71799 | pentatricopeptide repeat domain 1 | 1.15 | 0.019263 |
| Apon | 28194 | apolipoprotein N | 1.33 | 0.019277 |
| Cyp2s1 | 74134 | cytochrome P450, family 2, subfamily s, polypeptide 1 | 0.71 | 0.019322 |
| Gadd45g | 23882 | growth arrest and DNA-damage-inducible 45 gamma | 1.31 | 0.01934 |
| Erlin1 | 226144 | ER lipid raft associated 1 | 1.13 | 0.019345 |
| Rell1 | 100532 | RELT-like 1 | 1.15 | 0.019346 |
| Ift57 | 73916 | intraflagellar transport 57 | 1.13 | 0.01936 |
| Oxnad1 | 218885 | oxidoreductase NAD-binding domain containing 1 | 1.15 | 0.019363 |
| Pmvk | 68603 | phosphomevalonate kinase | 0.85 | 0.019439 |
| Tacc1 | 320165 | transforming, acidic coiled-coil containing protein 1 | 0.87 | 0.01944 |
| Synpo | 104027 | synaptopodin | 0.74 | 0.019458 |
| Rbpms | 19663 | RNA binding protein gene with multiple splicing | 0.84 | 0.019483 |
| Pnma1 | 70481 | paraneoplastic antigen MA1 | 0.7 | 0.019572 |
| Prkar2b | 19088 | protein kinase, cAMP dependent regulatory, type II beta | 0.87 | 0.019593 |
| F13a1 | 74145 | coagulation factor XIII, A1 subunit | 1.27 | 0.019607 |
| Osmr | 18414 | oncostatin M receptor | 0.82 | 0.019646 |
| Hsd17b7 | 15490 | hydroxysteroid (17-beta) dehydrogenase 7 | 0.81 | 0.019719 |
| Akirin2 | 433693 | akirin 2 | 0.86 | 0.019758 |
| Mia2 | 338320 | melanoma inhibitory activity 2 | 1.36 | 0.01979 |
| Scube3 | 268935 | signal peptide, CUB domain, EGF-like 3 | 0.47 | 0.019819 |
| Nkx2_2 | 18088 | NA | 0.63 | 0.019827 |
| Dlg3 | 53310 | discs, large homolog 3 (Drosophila) | 0.69 | 0.019886 |
| Ephb1 | 270190 | Eph receptor B1 | 1.48 | 0.019903 |
| Map6d1 | 208158 | MAP6 domain containing 1 | 2.03 | 0.019943 |
| Wisp1 | 22402 | WNT1 inducible signaling pathway protein 1 | 1.14 | 0.019951 |
| Cnn1 | 12797 | calponin 1 | 0.16 | 0.019992 |
| Zscan22 | 232878 | zinc finger and SCAN domain containing 22 | 0.85 | 0.019998 |
| Zscan26 | 432731 | NA | 0.84 | 0.020082 |
| Rasl11b | 68939 | RAS-like, family 11, member B | 1.32 | 0.020096 |
| Cdkn1b | 12576 | cyclin-dependent kinase inhibitor 1B | 0.83 | 0.020108 |
| Zbtb34 | 241311 | zinc finger and BTB domain containing 34 | 0.81 | 0.020155 |
| Homez | 239099 | homeodomain leucine zipper-encoding gene | 0.78 | 0.020195 |
| Map2k6 | 26399 | mitogen-activated protein kinase kinase 6 | 0.8 | 0.020254 |
| Napepld | 242864 | N-acyl phosphatidylethanolamine phospholipase D | 1.26 | 0.020258 |
| Tbc1d32 | 544696 | NA | 1.26 | 0.020259 |
| Tcf7 | 21414 | transcription factor 7, T cell specific | 0.77 | 0.020275 |
| Nutf2 | 68051 | nuclear transport factor 2 | 0.82 | 0.020288 |
| Wnk4 | 69847 | WNK lysine deficient protein kinase 4 | 0.76 | 0.020322 |
| Traf1 | 22029 | TNF receptor-associated factor 1 | 1.47 | 0.020346 |
| Rpia | 19895 | ribose 5-phosphate isomerase A | 1.17 | 0.020411 |
| Sema4a | 20351 | sema domain, immunoglobulin domain (Ig), transmembrane domain (TM) and short cytoplasmic domain, (semaphorin) 4A | 1.73 | 0.020483 |
| Gng7 | 14708 | guanine nucleotide binding protein (G protein), gamma 7 | 1.22 | 0.020489 |
| Ggcx | 56316 | gamma-glutamyl carboxylase | 0.82 | 0.020506 |
| Klhl22 | 224023 | kelch-like 22 (Drosophila) | 1.27 | 0.020521 |
| Tmem65 | 74868 | transmembrane protein 65 | 1.15 | 0.020522 |
| St3gal3 | 20441 | ST3 beta-galactoside alpha-2,3-sialyltransferase 3 | 0.82 | 0.020564 |
| Epb4.1l4b | 54357 | erythrocyte protein band 4.1-like 4b | 1.15 | 0.02058 |
| BC031181 | 407819 | cDNA sequence BC031181 | 0.87 | 0.020622 |
| Glrb | 14658 | glycine receptor, beta subunit | 1.53 | 0.020736 |
| Hsd3b7 | 101502 | hydroxy-delta-5-steroid dehydrogenase, 3 beta- and steroid delta-isomerase 7 | 1.3 | 0.020754 |
| 9330151L19Rik | 414085 | RIKEN cDNA 9330151L19 gene | 1.21 | 0.020789 |
| Pdia5 | 72599 | protein disulfide isomerase associated 5 | 1.26 | 0.020871 |
| Faah | 14073 | fatty acid amide hydrolase | 0.8 | 0.021007 |
| Tgfb3 | 21809 | transforming growth factor, beta 3 | 1.14 | 0.021087 |
| Adamts12 | 239337 | a disintegrin-like and metallopeptidase (reprolysin type) with thrombospondin type 1 motif, 12 | 1.2 | 0.021187 |
| Elp3 | 74195 | elongator acetyltransferase complex subunit 3 | 0.89 | 0.021214 |
| Zfp119a | 104349 | zinc finger protein 119a | 0.79 | 0.021261 |
| Tmem57 | 66146 | transmembrane protein 57 | 0.88 | 0.021262 |
| 2500004C02Rik | 72326 | RIKEN cDNA 2500004C02 gene | 0.7 | 0.021299 |
| Cep72 | 74470 | centrosomal protein 72 | 0.71 | 0.021344 |
| Mvb12b | 72543 | NA | 0.86 | 0.021351 |
| Cd28 | 12487 | CD28 antigen | 0.36 | 0.02137 |
| Cdkl2 | 53886 | cyclin-dependent kinase-like 2 (CDC2-related kinase) | 1.23 | 0.021397 |
| Cdk7 | 12572 | cyclin-dependent kinase 7 | 0.87 | 0.021675 |
| Zfp109 | 56869 | zinc finger protein 109 | 0.82 | 0.021851 |
| Ablim3 | 319713 | actin binding LIM protein family, member 3 | 1.74 | 0.021887 |
| Zfp174 | 385674 | zinc finger protein 174 | 1.27 | 0.021925 |
| Hrct1 | 1E+08 | histidine rich carboxyl terminus 1 | 1.43 | 0.021934 |
| Znrf2 | 387524 | zinc and ring finger 2 | 0.81 | 0.022138 |
| Tmod2 | 50876 | tropomodulin 2 | 1.24 | 0.022151 |
| Pigh | 110417 | phosphatidylinositol glycan anchor biosynthesis, class H | 0.86 | 0.022182 |
| Slc6a15 | 103098 | solute carrier family 6 (neurotransmitter transporter), member 15 | 2.09 | 0.022184 |
| Mre11a | 17535 | meiotic recombination 11 homolog A (S. cerevisiae) | 1.15 | 0.022185 |
| Mpp2 | 50997 | membrane protein, palmitoylated 2 (MAGUK p55 subfamily member 2) | 0.67 | 0.022219 |
| Btbd10 | 68815 | BTB (POZ) domain containing 10 | 1.17 | 0.022273 |
| Cnn2 | 12798 | calponin 2 | 0.87 | 0.022372 |
| Pde2a | 207728 | phosphodiesterase 2A, cGMP-stimulated | 1.2 | 0.022459 |
| Ccdc116 | 76872 | coiled-coil domain containing 116 | 1.14 | 0.022462 |
| Tom1l2 | 216810 | target of myb1-like 2 (chicken) | 0.85 | 0.022464 |
| Pdgfc | 54635 | platelet-derived growth factor, C polypeptide | 1.16 | 0.022488 |
| Mir22hg | 1E+08 | Mir22 host gene (non-protein coding) | 0.83 | 0.022489 |
| Bbs12 | 241950 | Bardet-Biedl syndrome 12 (human) | 1.38 | 0.022492 |
| Kcnj14 | 211480 | potassium inwardly-rectifying channel, subfamily J, member 14 | 1.46 | 0.022583 |
| Bcl2l11 | 12125 | BCL2-like 11 (apoptosis facilitator) | 0.87 | 0.022672 |
| Stk30 | 26448 | serine/threonine kinase 30 | 0.72 | 0.022718 |
| Recql5 | 170472 | RecQ protein-like 5 | 0.84 | 0.022737 |
| Hirip3 | 233876 | HIRA interacting protein 3 | 1.12 | 0.022737 |
| Dpm3 | 68563 | dolichyl-phosphate mannosyltransferase polypeptide 3 | 0.81 | 0.022755 |
| Rp9 | 55934 | retinitis pigmentosa 9 (human) | 0.8 | 0.022783 |
| Fads1 | 76267 | fatty acid desaturase 1 | 0.89 | 0.022796 |
| Ccdc64 | 75665 | coiled-coil domain containing 64 | 0.72 | 0.022799 |
| Zfp408 | 381410 | zinc finger protein 408 | 1.17 | 0.022813 |
| Mcl1 | 17210 | myeloid cell leukemia sequence 1 | 1.14 | 0.022827 |
| Fbxo48 | 319701 | F-box protein 48 | 1.27 | 0.022858 |
| Kif5c | 16574 | kinesin family member 5C | 1.16 | 0.023015 |
| Nthl1 | 18207 | nth (endonuclease III)-like 1 (E.coli) | 1.24 | 0.023018 |
| Pnpla7 | 241274 | patatin-like phospholipase domain containing 7 | 1.15 | 0.023173 |
| Xk | 22439 | Kell blood group precursor (McLeod phenotype) homolog | 1.23 | 0.023258 |
| Arhgdig | 14570 | Rho GDP dissociation inhibitor (GDI) gamma | 1.23 | 0.023323 |
| Fbxw2 | 30050 | F-box and WD-40 domain protein 2 | 1.12 | 0.023352 |
| Slpi | 20568 | secretory leukocyte peptidase inhibitor | 0.89 | 0.023374 |
| Ppp1r1b | 19049 | protein phosphatase 1, regulatory (inhibitor) subunit 1B | 0.8 | 0.023397 |
| Sla2 | 77799 | Src-like-adaptor 2 | 0.65 | 0.023463 |
| Nudt17 | 78373 | nudix (nucleoside diphosphate linked moiety X)-type motif 17 | 1.47 | 0.023471 |
| Atp11a | 50770 | ATPase, class VI, type 11A | 1.12 | 0.023547 |
| Gnb4 | 14696 | guanine nucleotide binding protein (G protein), beta 4 | 1.73 | 0.023641 |
| Cnpy3 | 72029 | canopy 3 homolog (zebrafish) | 1.15 | 0.023692 |
| Lsm10 | 116748 | U7 snRNP-specific Sm-like protein LSM10 | 0.84 | 0.023768 |
| Creg1 | 433375 | cellular repressor of E1A-stimulated genes 1 | 1.15 | 0.023819 |
| Chd9 | 109151 | chromodomain helicase DNA binding protein 9 | 1.2 | 0.023849 |
| Palb2 | 233826 | partner and localizer of BRCA2 | 1.18 | 0.023849 |
| Zfp296 | 63872 | zinc finger protein 296 | 1.34 | 0.023876 |
| Arhgef19 | 213649 | Rho guanine nucleotide exchange factor (GEF) 19 | 1.17 | 0.023916 |
| Marcks | 17118 | myristoylated alanine rich protein kinase C substrate | 0.83 | 0.024017 |
| Gla | 11605 | galactosidase, alpha | 0.86 | 0.024027 |
| Capn15 | 50817 | NA | 1.37 | 0.024077 |
| Glb1l | 74577 | galactosidase, beta 1-like | 1.22 | 0.024171 |
| Usp46 | 69727 | ubiquitin specific peptidase 46 | 1.22 | 0.024229 |
| Ahcyl2 | 74340 | S-adenosylhomocysteine hydrolase-like 2 | 0.86 | 0.024253 |
| Cxcl15 | 20309 | chemokine (C-X-C motif) ligand 15 | 0.29 | 0.024272 |
| Ulk2 | 29869 | unc-51 like kinase 2 | 0.82 | 0.024352 |
| Rbpj | 19664 | recombination signal binding protein for immunoglobulin kappa J region | 0.82 | 0.02437 |
| Zkscan7 | 382118 | NA | 1.34 | 0.024431 |
| Nid1 | 18073 | nidogen 1 | 0.61 | 0.024466 |
| Trim3 | 55992 | tripartite motif-containing 3 | 1.19 | 0.024466 |
| Foxa1 | 15375 | forkhead box A1 | 0.88 | 0.024496 |
| Msh5 | 17687 | mutS homolog 5 (E. coli) | 1.23 | 0.024617 |
| Degs2 | 70059 | degenerative spermatocyte homolog 2 (Drosophila), lipid desaturase | 1.23 | 0.024647 |
| Rnf44 | 105239 | ring finger protein 44 | 0.84 | 0.02468 |
| Gldc | 104174 | glycine decarboxylase | 1.26 | 0.024685 |
| Grhl2 | 252973 | grainyhead-like 2 (Drosophila) | 1.49 | 0.024706 |
| Sprr1a | 20753 | small proline-rich protein 1A | 1.41 | 0.024731 |
| Rnf25 | 57751 | ring finger protein 25 | 0.85 | 0.024739 |
| BC018242 | 235044 | cDNA sequence BC018242 | 0.68 | 0.024757 |
| Timp3 | 21859 | tissue inhibitor of metalloproteinase 3 | 1.17 | 0.024804 |
| Dnph1 | 381101 | NA | 1.14 | 0.024844 |
| Gm8909 | 667977 | predicted gene 8909 | 1.29 | 0.024943 |
| Ncam1 | 17967 | neural cell adhesion molecule 1 | 1.13 | 0.024948 |
| Plekha7 | 233765 | pleckstrin homology domain containing, family A member 7 | 0.65 | 0.024971 |
| Prrg2 | 65116 | proline-rich Gla (G-carboxyglutamic acid) polypeptide 2 | 1.19 | 0.024988 |
| Hs3st3b1 | 54710 | heparan sulfate (glucosamine) 3-O-sulfotransferase 3B1 | 1.17 | 0.024999 |
| Stk38 | 106504 | serine/threonine kinase 38 | 1.16 | 0.025092 |
| Dguok | 27369 | deoxyguanosine kinase | 1.14 | 0.025103 |
| Stx17 | 67727 | syntaxin 17 | 1.21 | 0.025149 |
| Vegfa | 22339 | vascular endothelial growth factor A | 1.19 | 0.025185 |
| Map3k14 | 53859 | mitogen-activated protein kinase kinase kinase 14 | 1.13 | 0.025204 |
| Cry2 | 12953 | cryptochrome 2 (photolyase-like) | 1.37 | 0.025239 |
| Cdk14 | 18647 | cyclin-dependent kinase 14 | 0.86 | 0.025292 |
| Rilp | 280408 | Rab interacting lysosomal protein | 1.71 | 0.025408 |
| Cdr2 | 12585 | cerebellar degeneration-related 2 | 1.15 | 0.025432 |
| Tm7sf2 | 73166 | transmembrane 7 superfamily member 2 | 0.56 | 0.025478 |
| Acox2 | 93732 | acyl-Coenzyme A oxidase 2, branched chain | 1.35 | 0.025523 |
| Ahctf1 | 226747 | AT hook containing transcription factor 1 | 1.16 | 0.025567 |
| Ppm1e | 320472 | protein phosphatase 1E (PP2C domain containing) | 0.86 | 0.025585 |
| Epb4.1l5 | 226352 | erythrocyte protein band 4.1-like 5 | 0.8 | 0.025726 |
| Fam132a | 67389 | family with sequence similarity 132, member A | 1.19 | 0.025753 |
| E2f3 | 13557 | E2F transcription factor 3 | 1.12 | 0.025781 |
| Gem | 14579 | GTP binding protein (gene overexpressed in skeletal muscle) | 0.78 | 0.025793 |
| Zfp658 | 210104 | zinc finger protein 658 | 0.86 | 0.025906 |
| Pak1 | 18479 | p21 protein (Cdc42/Rac)-activated kinase 1 | 1.16 | 0.026007 |
| Eif2d | 16865 | eukaryotic translation initiation factor 2D | 1.2 | 0.026011 |
| Slco2a1 | 24059 | solute carrier organic anion transporter family, member 2a1 | 0.7 | 0.026141 |
| Copg1 | 54161 | NA | 1.14 | 0.026146 |
| Slc48a1 | 67739 | solute carrier family 48 (heme transporter), member 1 | 1.18 | 0.026207 |
| Fkbp14 | 231997 | FK506 binding protein 14 | 0.79 | 0.026283 |
| Tead2 | 21677 | TEA domain family member 2 | 0.88 | 0.026343 |
| Ifi47 | 15953 | interferon gamma inducible protein 47 | 0.65 | 0.026442 |
| 2010315B03Rik | 630836 | RIKEN cDNA 2010315B03 gene | 0.75 | 0.026452 |
| Brms1l | 52592 | breast cancer metastasis-suppressor 1-like | 1.18 | 0.026454 |
| Zdhhc24 | 70605 | zinc finger, DHHC domain containing 24 | 1.16 | 0.026458 |
| Ptp4a1 | 19243 | protein tyrosine phosphatase 4a1 | 0.82 | 0.026496 |
| Rassf3 | 192678 | Ras association (RalGDS/AF-6) domain family member 3 | 0.84 | 0.02663 |
| Psenen | 66340 | presenilin enhancer 2 homolog (C. elegans) | 0.88 | 0.026698 |
| Nrm | 106582 | nurim (nuclear envelope membrane protein) | 1.14 | 0.026724 |
| Ago1 | 236511 | NA | 1.14 | 0.026737 |
| Wipf2 | 68524 | WAS/WASL interacting protein family, member 2 | 1.12 | 0.026789 |
| Naif1 | 71254 | nuclear apoptosis inducing factor 1 | 0.77 | 0.02688 |
| Rps29 | 20090 | ribosomal protein S29 | 0.82 | 0.026957 |
| Gm608 | 207806 | predicted gene 608 | 0.89 | 0.026985 |
| Rfc3 | 69263 | replication factor C (activator 1) 3 | 1.13 | 0.027026 |
| 1810043G02Rik | 67884 | RIKEN cDNA 1810043G02 gene | 1.23 | 0.027028 |
| Brat1 | 231841 | NA | 1.23 | 0.027083 |
| Cgn | 70737 | cingulin | 0.72 | 0.027103 |
| Npc1 | 18145 | Niemann Pick type C1 | 1.15 | 0.027136 |
| Syt7 | 54525 | synaptotagmin VII | 0.86 | 0.027217 |
| Acox3 | 80911 | acyl-Coenzyme A oxidase 3, pristanoyl | 0.88 | 0.027307 |
| Cyp4f16 | 70101 | cytochrome P450, family 4, subfamily f, polypeptide 16 | 1.2 | 0.027334 |
| Pias1 | 56469 | protein inhibitor of activated STAT 1 | 0.87 | 0.027335 |
| Ptpn21 | 24000 | protein tyrosine phosphatase, non-receptor type 21 | 1.13 | 0.02738 |
| Fancb | 237211 | Fanconi anemia, complementation group B | 1.31 | 0.027381 |
| Lipg | 16891 | lipase, endothelial | 0.8 | 0.027387 |
| Otud7b | 229603 | OTU domain containing 7B | 1.11 | 0.0274 |
| Cmtm6 | 67213 | CKLF-like MARVEL transmembrane domain containing 6 | 0.84 | 0.02759 |
| Cnn3 | 71994 | calponin 3, acidic | 0.9 | 0.027661 |
| 2610002J02Rik | 67513 | RIKEN cDNA 2610002J02 gene | 1.16 | 0.027927 |
| Neto2 | 74513 | neuropilin (NRP) and tolloid (TLL)-like 2 | 1.32 | 0.028061 |
| Trip11 | 109181 | thyroid hormone receptor interactor 11 | 1.19 | 0.02809 |
| Spata1 | 70951 | spermatogenesis associated 1 | 0.67 | 0.028171 |
| Zfp637 | 232337 | zinc finger protein 637 | 0.89 | 0.028226 |
| Bmp2k | 140780 | BMP2 inducible kinase | 1.16 | 0.028255 |
| Efna5 | 13640 | ephrin A5 | 0.87 | 0.028342 |
| Acsl3 | 74205 | acyl-CoA synthetase long-chain family member 3 | 0.85 | 0.028374 |
| Igf2bp1 | 140486 | insulin-like growth factor 2 mRNA binding protein 1 | 0.87 | 0.028439 |
| Coro1b | 23789 | coronin, actin binding protein 1B | 0.83 | 0.028454 |
| Cmklr1 | 14747 | chemokine-like receptor 1 | 1.6 | 0.028506 |
| Nrbp1 | 192292 | nuclear receptor binding protein 1 | 1.15 | 0.02859 |
| Ccl27a | 20301 | chemokine (C-C motif) ligand 27A | 0.67 | 0.028678 |
| Rassf8 | 71323 | Ras association (RalGDS/AF-6) domain family (N-terminal) member 8 | 1.13 | 0.028694 |
| Rragb | 245670 | Ras-related GTP binding B | 0.87 | 0.028825 |
| Ugt1a6a | 94284 | UDP glucuronosyltransferase 1 family, polypeptide A6A | 1.14 | 0.02886 |
| Atg10 | 66795 | autophagy related 10 | 1.26 | 0.02886 |
| Tbc1d25 | 209815 | TBC1 domain family, member 25 | 1.17 | 0.028944 |
| Sec23ip | 207352 | Sec23 interacting protein | 1.14 | 0.029143 |
| Snord52 | 1E+08 | small nucleolar RNA, C/D box 52 | 1.16 | 0.029159 |
| Pvrl2 | 19294 | poliovirus receptor-related 2 | 0.72 | 0.029202 |
| Mxd4 | 17122 | Max dimerization protein 4 | 0.86 | 0.029226 |
| Rbmxl1 | 19656 | RNA binding motif protein, X linked-like-1 | 0.89 | 0.029283 |
| Ctsw | 13041 | cathepsin W | 1.4 | 0.029289 |
| Kctd11 | 216858 | potassium channel tetramerisation domain containing 11 | 1.21 | 0.029346 |
| Mgp | 17313 | matrix Gla protein | 0.49 | 0.02935 |
| Mpi | 110119 | mannose phosphate isomerase | 1.13 | 0.029352 |
| Zfp346 | 26919 | zinc finger protein 346 | 1.12 | 0.029374 |
| Sash1 | 70097 | SAM and SH3 domain containing 1 | 1.16 | 0.029391 |
| Clpx | 270166 | caseinolytic peptidase X (E.coli) | 0.85 | 0.029434 |
| Ccdc174 | 232236 | coiled-coil domain containing 174 | 0.86 | 0.029454 |
| Spc24 | 67629 | SPC24, NDC80 kinetochore complex component, homolog (S. cerevisiae) | 1.16 | 0.029505 |
| Zcchc24 | 71918 | zinc finger, CCHC domain containing 24 | 0.9 | 0.029541 |
| Gpr116 | 224792 | G protein-coupled receptor 116 | 1.28 | 0.029569 |
| Nfatc1 | 18018 | nuclear factor of activated T cells, cytoplasmic, calcineurin dependent 1 | 1.13 | 0.029689 |
| Zfp65 | 235907 | NA | 0.79 | 0.029711 |
| Slc9a8 | 77031 | solute carrier family 9 (sodium/hydrogen exchanger), member 8 | 0.89 | 0.029758 |
| D930015E06Rik | 229473 | RIKEN cDNA D930015E06 gene | 1.14 | 0.029768 |
| Ephb3 | 13845 | Eph receptor B3 | 0.79 | 0.029811 |
| Camk4 | 12326 | calcium/calmodulin-dependent protein kinase IV | 0.49 | 0.029828 |
| Areg | 11839 | amphiregulin | 1.15 | 0.029908 |
| Fbxo36 | 66153 | F-box protein 36 | 0.69 | 0.029923 |
| Fam180a | 208164 | family with sequence similarity 180, member A | 1.16 | 0.030052 |
| Isoc1 | 66307 | isochorismatase domain containing 1 | 1.13 | 0.030132 |
| Il17rc | 171095 | interleukin 17 receptor C | 1.3 | 0.0303 |
| AI429214 | 621080 | expressed sequence AI429214 | 0.81 | 0.030352 |
| Vmac | 106639 | vimentin-type intermediate filament associated coiled-coil protein | 0.85 | 0.030357 |
| Suv420h1 | 225888 | suppressor of variegation 4-20 homolog 1 (Drosophila) | 0.88 | 0.030388 |
| Rbm47 | 245945 | RNA binding motif protein 47 | 0.78 | 0.030637 |
| Ngf | 18049 | nerve growth factor | 1.52 | 0.03068 |
| Wif1 | 24117 | Wnt inhibitory factor 1 | 0.47 | 0.030705 |
| Usp28 | 235323 | ubiquitin specific peptidase 28 | 0.88 | 0.030706 |
| Ttc39c | 72747 | tetratricopeptide repeat domain 39C | 1.24 | 0.030761 |
| 8-Mar | 71779 | membrane-associated ring finger (C3HC4) 8 | 1.15 | 0.030813 |
| Lars2 | 102436 | leucyl-tRNA synthetase, mitochondrial | 0.83 | 0.030913 |
| Megf11 | 214058 | multiple EGF-like-domains 11 | 1.48 | 0.030957 |
| Vezt | 215008 | vezatin, adherens junctions transmembrane protein | 1.15 | 0.031019 |
| Commd10 | 69456 | COMM domain containing 10 | 0.85 | 0.031071 |
| Ckap4 | 216197 | cytoskeleton-associated protein 4 | 0.88 | 0.031094 |
| Parva | 57342 | parvin, alpha | 0.89 | 0.031099 |
| Rere | 68703 | arginine glutamic acid dipeptide (RE) repeats | 0.88 | 0.031141 |
| Tmem106a | 217203 | transmembrane protein 106A | 1.22 | 0.031215 |
| Sft2d3 | 67158 | SFT2 domain containing 3 | 0.88 | 0.031264 |
| Tcf7l2 | 21416 | transcription factor 7 like 2, T cell specific, HMG box | 1.15 | 0.031339 |
| Dclre1c | 227525 | DNA cross-link repair 1C, PSO2 homolog (S. cerevisiae) | 0.83 | 0.031364 |
| Tmem79 | 71913 | transmembrane protein 79 | 1.21 | 0.031373 |
| Mrs2 | 380836 | MRS2 magnesium homeostasis factor homolog (S. cerevisiae) | 1.14 | 0.031382 |
| Pcdhga7 | 93715 | protocadherin gamma subfamily A, 7 | 0.86 | 0.031392 |
| Aldh4a1 | 212647 | aldehyde dehydrogenase 4 family, member A1 | 0.82 | 0.031402 |
| Mipep | 70478 | mitochondrial intermediate peptidase | 1.16 | 0.031422 |
| Zfp882 | 382019 | zinc finger protein 882 | 0.75 | 0.031436 |
| Leprel1 | 210530 | leprecan-like 1 | 0.87 | 0.031618 |
| Snora65 | 104367 | small nucleolar RNA, H/ACA box 65 | 1.2 | 0.031801 |
| Mrpl24 | 67707 | mitochondrial ribosomal protein L24 | 1.11 | 0.031828 |
| Abhd2 | 54608 | abhydrolase domain containing 2 | 1.21 | 0.031889 |
| 2610034B18Rik | 70420 | RIKEN cDNA 2610034B18 gene | 1.13 | 0.032059 |
| Aim1 | 11630 | absent in melanoma 1 | 0.9 | 0.03219 |
| Tnc | 21923 | tenascin C | 1.18 | 0.032192 |
| Xrcc4 | 108138 | X-ray repair complementing defective repair in Chinese hamster cells 4 | 1.3 | 0.032206 |
| Ogn | 18295 | osteoglycin | 0.82 | 0.03223 |
| Kank3 | 80880 | KN motif and ankyrin repeat domains 3 | 1.26 | 0.032246 |
| Klhl5 | 71778 | kelch-like 5 (Drosophila) | 1.12 | 0.032285 |
| Rprd1a | 225283 | regulation of nuclear pre-mRNA domain containing 1A | 1.17 | 0.032338 |
| Foxj2 | 60611 | forkhead box J2 | 1.15 | 0.032345 |
| Camkmt | 73582 | calmodulin-lysine N-methyltransferase | 0.87 | 0.032359 |
| Tatdn2 | 381801 | TatD DNase domain containing 2 | 1.12 | 0.032414 |
| Epb4.1 | 269587 | erythrocyte protein band 4.1 | 0.9 | 0.032429 |
| Zbtb12 | 193736 | zinc finger and BTB domain containing 12 | 0.81 | 0.032479 |
| Nipsnap1 | 18082 | 4-nitrophenylphosphatase domain and non-neuronal SNAP25-like protein homolog 1 (C. elegans) | 1.14 | 0.032501 |
| Hsdl1 | 72552 | hydroxysteroid dehydrogenase like 1 | 1.15 | 0.032504 |
| Fam169a | 320557 | family with sequence similarity 169, member A | 0.82 | 0.032509 |
| Ppap2a | 19012 | phosphatidic acid phosphatase type 2A | 0.89 | 0.032515 |
| Acpl2 | 235534 | acid phosphatase-like 2 | 0.86 | 0.032521 |
| Vps13c | 320528 | vacuolar protein sorting 13C (yeast) | 0.88 | 0.032611 |
| Rilpl1 | 75695 | Rab interacting lysosomal protein-like 1 | 0.89 | 0.032689 |
| Uxs1 | 67883 | UDP-glucuronate decarboxylase 1 | 1.12 | 0.032716 |
| Mmab | 77697 | methylmalonic aciduria (cobalamin deficiency) type B homolog (human) | 0.84 | 0.032819 |
| Ephb2 | 13844 | Eph receptor B2 | 0.82 | 0.032846 |
| Klhl42 | 232539 | NA | 1.15 | 0.032858 |
| Paox | 212503 | polyamine oxidase (exo-N4-amino) | 1.26 | 0.032859 |
| Rsg1 | 76166 | REM2 and RAB-like small GTPase 1 | 1.57 | 0.032897 |
| Itga9 | 104099 | integrin alpha 9 | 0.85 | 0.032947 |
| Plxna3 | 18846 | plexin A3 | 0.8 | 0.032957 |
| Trim37 | 68729 | tripartite motif-containing 37 | 1.14 | 0.032984 |
| Efemp1 | 216616 | epidermal growth factor-containing fibulin-like extracellular matrix protein 1 | 0.83 | 0.033017 |
| Col5a2 | 12832 | collagen, type V, alpha 2 | 1.11 | 0.033067 |
| Neat1 | 66961 | nuclear paraspeckle assembly transcript 1 (non-protein coding) | 0.84 | 0.033178 |
| Mcm3 | 17215 | minichromosome maintenance deficient 3 (S. cerevisiae) | 1.12 | 0.033261 |
| Abca3 | 27410 | ATP-binding cassette, sub-family A (ABC1), member 3 | 1.12 | 0.033294 |
| Ptplb | 70757 | protein tyrosine phosphatase-like (proline instead of catalytic arginine), member b | 0.87 | 0.033318 |
| Arhgap18 | 73910 | Rho GTPase activating protein 18 | 0.74 | 0.033391 |
| Ugt1a6b | 394435 | UDP glucuronosyltransferase 1 family, polypeptide A6B | 1.2 | 0.033448 |
| Snora31 | 1E+08 | small nucleolar RNA, H/ACA box 31 | 0.66 | 0.033449 |
| Peli2 | 93834 | pellino 2 | 0.85 | 0.033521 |
| Glud1 | 14661 | glutamate dehydrogenase 1 | 1.11 | 0.033545 |
| Rhob | 11852 | ras homolog gene family, member B | 1.21 | 0.033589 |
| Chd5 | 269610 | chromodomain helicase DNA binding protein 5 | 1.48 | 0.033617 |
| Eno3 | 13808 | enolase 3, beta muscle | 1.13 | 0.033771 |
| Ngef | 53972 | neuronal guanine nucleotide exchange factor | 0.56 | 0.033793 |
| Mfap1b | 1E+08 | microfibrillar-associated protein 1B | 0.88 | 0.033826 |
| Sec14l1 | 74136 | SEC14-like 1 (S. cerevisiae) | 0.87 | 0.033982 |
| Lbp | 16803 | lipopolysaccharide binding protein | 0.79 | 0.033991 |
| Foxd1 | 15229 | forkhead box D1 | 0.76 | 0.034034 |
| Inpp5a | 212111 | inositol polyphosphate-5-phosphatase A | 0.87 | 0.034082 |
| Glrx2 | 69367 | glutaredoxin 2 (thioltransferase) | 0.89 | 0.0341 |
| Arhgef3 | 71704 | Rho guanine nucleotide exchange factor (GEF) 3 | 1.34 | 0.034104 |
| Sh2d4a | 72281 | SH2 domain containing 4A | 0.75 | 0.034144 |
| Ecsit | 26940 | ECSIT homolog (Drosophila) | 1.12 | 0.034208 |
| Dhrs7 | 66375 | dehydrogenase/reductase (SDR family) member 7 | 1.18 | 0.034282 |
| Lrpap1 | 16976 | low density lipoprotein receptor-related protein associated protein 1 | 0.84 | 0.034348 |
| Cpeb3 | 208922 | cytoplasmic polyadenylation element binding protein 3 | 0.86 | 0.034398 |
| Pde4d | 238871 | phosphodiesterase 4D, cAMP specific | 0.89 | 0.034408 |
| Tfr2 | 50765 | transferrin receptor 2 | 1.29 | 0.034499 |
| Pcgf6 | 71041 | polycomb group ring finger 6 | 0.89 | 0.034631 |
| Tomt | 791260 | transmembrane O-methyltransferase | 0.84 | 0.034652 |
| Bend4 | 666938 | BEN domain containing 4 | 0.79 | 0.034707 |
| Ttc13 | 234875 | tetratricopeptide repeat domain 13 | 1.11 | 0.034707 |
| Fam193a | 231128 | family with sequence similarity 193, member A | 0.89 | 0.034759 |
| D630045J12Rik | 330286 | RIKEN cDNA D630045J12 gene | 0.86 | 0.034765 |
| Lyn | 17096 | Yamaguchi sarcoma viral (v-yes-1) oncogene homolog | 0.85 | 0.034852 |
| Anpep | 16790 | alanyl (membrane) aminopeptidase | 0.89 | 0.034855 |
| Smyd4 | 319822 | SET and MYND domain containing 4 | 1.19 | 0.034866 |
| Idh1 | 15926 | isocitrate dehydrogenase 1 (NADP+), soluble | 0.86 | 0.034867 |
| Klhdc1 | 271005 | kelch domain containing 1 | 1.28 | 0.034962 |
| Sap18 | 20220 | Sin3-associated polypeptide 18 | 0.88 | 0.035019 |
| Maff | 17133 | v-maf musculoaponeurotic fibrosarcoma oncogene family, protein F (avian) | 0.83 | 0.035135 |
| Catsper2 | 212670 | cation channel, sperm associated 2 | 1.54 | 0.035143 |
| Stat3 | 20848 | signal transducer and activator of transcription 3 | 0.89 | 0.035197 |
| Htatip2 | 53415 | HIV-1 tat interactive protein 2, homolog (human) | 1.11 | 0.035366 |
| Fam189b | 68521 | family with sequence similarity 189, member B | 0.76 | 0.035402 |
| Efnb2 | 13642 | ephrin B2 | 1.13 | 0.035525 |
| Camta1 | 100072 | calmodulin binding transcription activator 1 | 0.83 | 0.035545 |
| Ptpre | 19267 | protein tyrosine phosphatase, receptor type, E | 1.18 | 0.035579 |
| Pcsk7 | 18554 | proprotein convertase subtilisin/kexin type 7 | 1.19 | 0.03561 |
| Chfr | 231600 | checkpoint with forkhead and ring finger domains | 0.9 | 0.035661 |
| Fdps | 110196 | farnesyl diphosphate synthetase | 0.89 | 0.035682 |
| Zbtb7b | 22724 | zinc finger and BTB domain containing 7B | 0.78 | 0.035849 |
| Map3k6 | 53608 | mitogen-activated protein kinase kinase kinase 6 | 1.25 | 0.035912 |
| Pigg | 433931 | phosphatidylinositol glycan anchor biosynthesis, class G | 1.18 | 0.03595 |
| Fbxo9 | 71538 | f-box protein 9 | 1.16 | 0.036041 |
| Styk1 | 243659 | serine/threonine/tyrosine kinase 1 | 1.37 | 0.036042 |
| Bmper | 73230 | BMP-binding endothelial regulator | 1.18 | 0.036053 |
| Timp1 | 21857 | tissue inhibitor of metalloproteinase 1 | 0.9 | 0.036107 |
| Creb3l1 | 26427 | cAMP responsive element binding protein 3-like 1 | 0.83 | 0.036118 |
| Sesn2 | 230784 | sestrin 2 | 1.21 | 0.036228 |
| Mex3b | 108797 | mex3 homolog B (C. elegans) | 0.89 | 0.036255 |
| Kidins220 | 77480 | kinase D-interacting substrate 220 | 1.13 | 0.036307 |
| Pyhin1 | 236312 | pyrin and HIN domain family, member 1 | 1.49 | 0.036345 |
| Cenpa | 12615 | centromere protein A | 0.86 | 0.03636 |
| Trappc10 | 216131 | trafficking protein particle complex 10 | 1.11 | 0.036449 |
| Urod | 22275 | uroporphyrinogen decarboxylase | 0.86 | 0.036515 |
| Pnpla6 | 50767 | patatin-like phospholipase domain containing 6 | 1.14 | 0.036527 |
| Fam13c | 71721 | family with sequence similarity 13, member C | 1.34 | 0.036547 |
| Ptgfrn | 19221 | prostaglandin F2 receptor negative regulator | 1.12 | 0.036577 |
| Adipor1 | 72674 | adiponectin receptor 1 | 1.14 | 0.036608 |
| Prtg | 235472 | protogenin homolog (Gallus gallus) | 0.84 | 0.03661 |
| Ndufc1 | 66377 | NADH dehydrogenase (ubiquinone) 1, subcomplex unknown, 1 | 0.84 | 0.036632 |
| Il17rd | 171463 | interleukin 17 receptor D | 1.43 | 0.036685 |
| Hspa1l | 15482 | heat shock protein 1-like | 1.32 | 0.036744 |
| Lrrc56 | 70552 | leucine rich repeat containing 56 | 0.58 | 0.036759 |
| 5730508B09Rik | 70617 | RIKEN cDNA 5730508B09 gene | 0.77 | 0.03682 |
| Mipol1 | 73490 | mirror-image polydactyly gene 1 homolog (human) | 0.86 | 0.036852 |
| Car12 | 76459 | carbonic anyhydrase 12 | 1.12 | 0.036857 |
| Map1lc3b | 67443 | microtubule-associated protein 1 light chain 3 beta | 0.84 | 0.0369 |
| 2610318N02Rik | 70458 | RIKEN cDNA 2610318N02 gene | 0.81 | 0.036943 |
| Gm20300 | 1.01E+08 | predicted gene, 20300 | 0.84 | 0.036994 |
| Snap23 | 20619 | synaptosomal-associated protein 23 | 0.84 | 0.03712 |
| Mast4 | 328329 | microtubule associated serine/threonine kinase family member 4 | 1.13 | 0.037137 |
| Myadm | 50918 | myeloid-associated differentiation marker | 0.9 | 0.037155 |
| Tmem101 | 76547 | transmembrane protein 101 | 0.81 | 0.037166 |
| Sytl2 | 83671 | synaptotagmin-like 2 | 0.66 | 0.037194 |
| Rbpms2 | 71973 | RNA binding protein with multiple splicing 2 | 0.74 | 0.037298 |
| Ptma | 19231 | prothymosin alpha | 0.87 | 0.03731 |
| Ube2w | 66799 | ubiquitin-conjugating enzyme E2W (putative) | 1.25 | 0.037351 |
| Slc9a2 | 226999 | solute carrier family 9 (sodium/hydrogen exchanger), member 2 | 1.42 | 0.037381 |
| Napb | 17957 | N-ethylmaleimide sensitive fusion protein attachment protein beta | 0.7 | 0.037426 |
| Gpr157 | 269604 | G protein-coupled receptor 157 | 1.68 | 0.037438 |
| Tmsb4x | 19241 | thymosin, beta 4, X chromosome | 0.81 | 0.03747 |
| Tnfsf13 | 69583 | tumor necrosis factor (ligand) superfamily, member 13 | 0.74 | 0.037546 |
| Loxl4 | 67573 | lysyl oxidase-like 4 | 0.89 | 0.037559 |
| Fam131b | 76156 | family with sequence similarity 131, member B | 0.8 | 0.037594 |
| Zfp334 | 228876 | zinc finger protein 334 | 0.87 | 0.037751 |
| Casp8 | 12370 | caspase 8 | 1.16 | 0.037758 |
| Efna1 | 13636 | ephrin A1 | 1.15 | 0.037781 |
| Capn2 | 12334 | calpain 2 | 1.11 | 0.037832 |
| Slc7a8 | 50934 | solute carrier family 7 (cationic amino acid transporter, y+ system), member 8 | 0.87 | 0.03784 |
| Psmf1 | 228769 | proteasome (prosome, macropain) inhibitor subunit 1 | 1.17 | 0.037931 |
| Tcte2 | 21646 | t-complex-associated testis expressed 2 | 1.28 | 0.038004 |
| Dpf1 | 29861 | D4, zinc and double PHD fingers family 1 | 0.79 | 0.038068 |
| Tle4 | 21888 | transducin-like enhancer of split 4, homolog of Drosophila E(spl) | 0.83 | 0.038153 |
| Eid1 | 58521 | EP300 interacting inhibitor of differentiation 1 | 0.86 | 0.038159 |
| Crim1 | 50766 | cysteine rich transmembrane BMP regulator 1 (chordin like) | 1.13 | 0.038257 |
| Fdx1 | 14148 | ferredoxin 1 | 1.21 | 0.038297 |
| Rbms2 | 56516 | RNA binding motif, single stranded interacting protein 2 | 1.11 | 0.038328 |
| Slc9a5 | 277973 | solute carrier family 9 (sodium/hydrogen exchanger), member 5 | 1.2 | 0.038338 |
| Lamc3 | 23928 | laminin gamma 3 | 0.77 | 0.038388 |
| Sephs1 | 109079 | selenophosphate synthetase 1 | 0.89 | 0.038454 |
| Txlna | 109658 | taxilin alpha | 1.13 | 0.038483 |
| Mthfd2l | 665563 | methylenetetrahydrofolate dehydrogenase (NADP+ dependent) 2-like | 0.84 | 0.038521 |
| Pkd1 | 18763 | polycystic kidney disease 1 homolog | 1.33 | 0.038553 |
| Ypel2 | 77864 | yippee-like 2 (Drosophila) | 0.85 | 0.038556 |
| Sfmbt1 | 54650 | Scm-like with four mbt domains 1 | 1.16 | 0.03863 |
| Ift43 | 76411 | intraflagellar transport 43 homolog (Chlamydomonas) | 0.87 | 0.038688 |
| E2f6 | 50496 | E2F transcription factor 6 | 0.85 | 0.03878 |
| Fgfr1 | 14182 | fibroblast growth factor receptor 1 | 0.83 | 0.038821 |
| Plod2 | 26432 | procollagen lysine, 2-oxoglutarate 5-dioxygenase 2 | 1.16 | 0.038893 |
| Sepw1 | 20364 | selenoprotein W, muscle 1 | 1.15 | 0.038942 |
| Katnal2 | 71206 | katanin p60 subunit A-like 2 | 1.27 | 0.038951 |
| Cep85l | 1E+08 | NA | 1.48 | 0.038984 |
| Tnpo2 | 212999 | transportin 2 (importin 3, karyopherin beta 2b) | 1.11 | 0.038991 |
| Pex1 | 71382 | peroxisomal biogenesis factor 1 | 0.87 | 0.039008 |
| Gnl1 | 14670 | guanine nucleotide binding protein-like 1 | 1.18 | 0.039127 |
| Poc1b | 382406 | POC1 centriolar protein homolog B (Chlamydomonas) | 0.84 | 0.03916 |
| Nhsl2 | 1E+08 | NHS-like 2 | 0.77 | 0.039179 |
| Tpi1 | 21991 | triosephosphate isomerase 1 | 1.12 | 0.039226 |
| Traf6 | 22034 | TNF receptor-associated factor 6 | 0.88 | 0.039241 |
| Rundc1 | 217201 | RUN domain containing 1 | 1.18 | 0.039264 |
| Cltb | 74325 | clathrin, light polypeptide (Lcb) | 1.18 | 0.039377 |
| Gnpda2 | 67980 | glucosamine-6-phosphate deaminase 2 | 1.24 | 0.039535 |
| Kcnh2 | 16511 | potassium voltage-gated channel, subfamily H (eag-related), member 2 | 0.64 | 0.039668 |
| Emp2 | 13731 | epithelial membrane protein 2 | 1.14 | 0.039674 |
| Hrh1 | 15465 | histamine receptor H1 | 1.27 | 0.039702 |
| Oaf | 102644 | OAF homolog (Drosophila) | 0.83 | 0.039871 |
| Cxxc5 | 67393 | CXXC finger 5 | 0.89 | 0.039881 |
| Vimp | 109815 | NA | 0.9 | 0.039956 |
| Spata13 | 219140 | spermatogenesis associated 13 | 0.81 | 0.040029 |
| Ube2ql1 | 76980 | ubiquitin-conjugating enzyme E2Q family-like 1 | 1.92 | 0.040095 |
| Mapkapk5 | 17165 | MAP kinase-activated protein kinase 5 | 1.12 | 0.040126 |
| Ube2l3 | 22195 | ubiquitin-conjugating enzyme E2L 3 | 0.91 | 0.04017 |
| Nrep | 27528 | NA | 0.86 | 0.040224 |
| Tapbpl | 213233 | TAP binding protein-like | 1.18 | 0.040228 |
| Iba57 | 216792 | IBA57, iron-sulfur cluster assembly homolog (S. cerevisiae) | 0.87 | 0.040288 |
| Oser1 | 66680 | NA | 1.18 | 0.040312 |
| Fbxo4 | 106052 | F-box protein 4 | 1.13 | 0.040334 |
| Ak5 | 229949 | adenylate kinase 5 | 0.83 | 0.040374 |
| St7 | 64213 | suppression of tumorigenicity 7 | 1.12 | 0.040386 |
| Sod3 | 20657 | superoxide dismutase 3, extracellular | 1.15 | 0.040504 |
| Tnfaip6 | 21930 | tumor necrosis factor alpha induced protein 6 | 1.17 | 0.04054 |
| Ppl | 19041 | periplakin | 1.17 | 0.040549 |
| 4930503L19Rik | 269033 | RIKEN cDNA 4930503L19 gene | 1.23 | 0.040554 |
| Eif1b | 68969 | eukaryotic translation initiation factor 1B | 0.9 | 0.040678 |
| Lpin1 | 14245 | lipin 1 | 0.83 | 0.040728 |
| Chp1 | 56398 | NA | 0.9 | 0.040835 |
| Ptpn3 | 545622 | protein tyrosine phosphatase, non-receptor type 3 | 1.17 | 0.040866 |
| Hist1h4i | 319158 | histone cluster 1, H4i | 0.84 | 0.041016 |
| 2900097C17Rik | 347740 | RIKEN cDNA 2900097C17 gene | 0.9 | 0.041112 |
| Enthd2 | 78777 | ENTH domain containing 2 | 1.27 | 0.041123 |
| Lrig2 | 269473 | leucine-rich repeats and immunoglobulin-like domains 2 | 0.88 | 0.041202 |
| 1600012H06Rik | 67912 | RIKEN cDNA 1600012H06 gene | 1.11 | 0.041224 |
| Shq1 | 72171 | SHQ1 homolog (S. cerevisiae) | 1.12 | 0.041238 |
| Tex2 | 21763 | testis expressed gene 2 | 1.11 | 0.04148 |
| Ppapdc1b | 71910 | phosphatidic acid phosphatase type 2 domain containing 1B | 1.18 | 0.041513 |
| Ilk | 16202 | integrin linked kinase | 0.9 | 0.041525 |
| Ndufv2 | 72900 | NADH dehydrogenase (ubiquinone) flavoprotein 2 | 0.89 | 0.041605 |
| Adam8 | 11501 | a disintegrin and metallopeptidase domain 8 | 1.39 | 0.041714 |
| Pigl | 327942 | phosphatidylinositol glycan anchor biosynthesis, class L | 1.13 | 0.041771 |
| Fam129c | 1E+08 | family with sequence similarity 129, member C | 1.18 | 0.041812 |
| 9430016H08Rik | 68115 | RIKEN cDNA 9430016H08 gene | 0.9 | 0.04189 |
| Nav1 | 215690 | neuron navigator 1 | 1.13 | 0.041893 |
| Ripply1 | 622473 | ripply1 homolog (zebrafish) | 0.88 | 0.041983 |
| Slco4a1 | 108115 | solute carrier organic anion transporter family, member 4a1 | 0.71 | 0.042036 |
| Plaur | 18793 | plasminogen activator, urokinase receptor | 1.16 | 0.04204 |
| Il34 | 76527 | interleukin 34 | 1.32 | 0.042182 |
| Hddc2 | 69692 | HD domain containing 2 | 1.13 | 0.04244 |
| Arid5a | 214855 | AT rich interactive domain 5A (MRF1-like) | 1.18 | 0.042535 |
| Slc2a8 | 56017 | solute carrier family 2, (facilitated glucose transporter), member 8 | 0.72 | 0.042572 |
| Hcfc1r1 | 353502 | host cell factor C1 regulator 1 (XPO1-dependent) | 0.84 | 0.042589 |
| Zfp28 | 22690 | zinc finger protein 28 | 0.81 | 0.042608 |
| Tiam1 | 21844 | T cell lymphoma invasion and metastasis 1 | 1.12 | 0.042805 |
| Golga1 | 76899 | golgi autoantigen, golgin subfamily a, 1 | 1.17 | 0.042807 |
| 9430015G10Rik | 230996 | RIKEN cDNA 9430015G10 gene | 1.13 | 0.042865 |
| Nr1d1 | 217166 | nuclear receptor subfamily 1, group D, member 1 | 1.21 | 0.042875 |
| Slc24a3 | 94249 | solute carrier family 24 (sodium/potassium/calcium exchanger), member 3 | 1.12 | 0.042921 |
| Dhx32 | 101437 | DEAH (Asp-Glu-Ala-His) box polypeptide 32 | 1.11 | 0.042942 |
| 4933426M11Rik | 217684 | RIKEN cDNA 4933426M11 gene | 1.12 | 0.043103 |
| Zhx2 | 387609 | zinc fingers and homeoboxes 2 | 1.19 | 0.04312 |
| Mcc | 328949 | mutated in colorectal cancers | 1.13 | 0.043198 |
| Ubtd1 | 226122 | ubiquitin domain containing 1 | 1.21 | 0.043207 |
| Prpsap2 | 212627 | phosphoribosyl pyrophosphate synthetase-associated protein 2 | 1.15 | 0.043258 |
| Tex36 | 73808 | testis expressed 36 | 1.58 | 0.04326 |
| Foxo4 | 54601 | forkhead box O4 | 0.9 | 0.043278 |
| Ift20 | 55978 | intraflagellar transport 20 | 0.89 | 0.043292 |
| Gm16062 | 1.01E+08 | predicted gene 16062 | 1.28 | 0.043312 |
| Creb3 | 12913 | cAMP responsive element binding protein 3 | 1.14 | 0.043439 |
| Mrm1 | 217038 | mitochondrial rRNA methyltransferase 1 homolog (S. cerevisiae) | 1.15 | 0.043542 |
| Sema6b | 20359 | sema domain, transmembrane domain (TM), and cytoplasmic domain, (semaphorin) 6B | 1.39 | 0.0437 |
| 2010320M18Rik | 72093 | RIKEN cDNA 2010320M18 gene | 0.74 | 0.043721 |
| Bai2 | 230775 | brain-specific angiogenesis inhibitor 2 | 0.63 | 0.043757 |
| Atg9b | 213948 | autophagy related 9B | 1.52 | 0.043781 |
| Tctex1d2 | 66061 | Tctex1 domain containing 2 | 1.2 | 0.043785 |
| Purg | 75029 | purine-rich element binding protein G | 0.78 | 0.043848 |
| Tanc1 | 66860 | tetratricopeptide repeat, ankyrin repeat and coiled-coil containing 1 | 1.11 | 0.043932 |
| Mrgpre | 244238 | MAS-related GPR, member E | 1.28 | 0.044008 |
| Aplf | 72103 | aprataxin and PNKP like factor | 0.89 | 0.044079 |
| Hbp1 | 73389 | high mobility group box transcription factor 1 | 1.18 | 0.04408 |
| Mtrf1 | 211253 | mitochondrial translational release factor 1 | 1.19 | 0.044083 |
| Wdyhv1 | 76773 | WDYHV motif containing 1 | 0.86 | 0.044152 |
| Evi5 | 14020 | ecotropic viral integration site 5 | 0.86 | 0.04423 |
| Pcgf1 | 69837 | polycomb group ring finger 1 | 1.16 | 0.044242 |
| Arhgef5 | 54324 | Rho guanine nucleotide exchange factor (GEF) 5 | 1.1 | 0.044417 |
| Plagl2 | 54711 | pleiomorphic adenoma gene-like 2 | 1.12 | 0.044441 |
| Ankrd1 | 107765 | ankyrin repeat domain 1 (cardiac muscle) | 1.18 | 0.044457 |
| Onecut2 | 225631 | one cut domain, family member 2 | 0.82 | 0.04453 |
| Nin | 18080 | ninein | 1.1 | 0.044576 |
| Orm3 | 18407 | orosomucoid 3 | 1.56 | 0.044666 |
| Cep128 | 75216 | centrosomal protein 128 | 1.18 | 0.044692 |
| Add2 | 11519 | adducin 2 (beta) | 1.49 | 0.044704 |
| Slc27a3 | 26568 | solute carrier family 27 (fatty acid transporter), member 3 | 0.74 | 0.044784 |
| Zmat5 | 67178 | zinc finger, matrin type 5 | 1.13 | 0.044885 |
| Mxd3 | 17121 | Max dimerization protein 3 | 1.23 | 0.045 |
| Cenpi | 102920 | centromere protein I | 1.14 | 0.045005 |
| Tubb6 | 67951 | tubulin, beta 6 class V | 1.21 | 0.045012 |
| Fam107b | 66540 | family with sequence similarity 107, member B | 0.84 | 0.045151 |
| Tmem194b | 227094 | transmembrane protein 194B | 0.85 | 0.045173 |
| Baz2a | 116848 | bromodomain adjacent to zinc finger domain, 2A | 0.9 | 0.0452 |
| Anapc11 | 66156 | anaphase promoting complex subunit 11 | 1.19 | 0.045365 |
| Dicer1 | 192119 | dicer 1, ribonuclease type III | 0.89 | 0.045367 |
| Scarb2 | 12492 | scavenger receptor class B, member 2 | 0.9 | 0.045419 |
| Tmem241 | 338363 | transmembrane protein 241 | 0.79 | 0.045484 |
| Ttyh3 | 78339 | tweety homolog 3 (Drosophila) | 0.74 | 0.04554 |
| Psme2 | 19188 | proteasome (prosome, macropain) 28 subunit, beta | 1.12 | 0.045603 |
| Rufy4 | 435626 | RUN and FYVE domain containing 4 | 0.81 | 0.045671 |
| Ube2r2 | 67615 | ubiquitin-conjugating enzyme E2R 2 | 0.87 | 0.04573 |
| Klf2 | 16598 | Kruppel-like factor 2 (lung) | 1.62 | 0.045736 |
| A530046M15Rik | 328190 | NA | 1.48 | 0.045793 |
| Irak1 | 16179 | interleukin-1 receptor-associated kinase 1 | 0.9 | 0.046064 |
| Comt | 12846 | catechol-O-methyltransferase | 1.13 | 0.046321 |
| Nrip2 | 60345 | nuclear receptor interacting protein 2 | 0.74 | 0.046358 |
| Snx18 | 170625 | sorting nexin 18 | 0.91 | 0.046505 |
| Alg3 | 208624 | asparagine-linked glycosylation 3 (alpha-1,3-mannosyltransferase) | 0.87 | 0.046588 |
| Tmem45a | 56277 | transmembrane protein 45a | 1.41 | 0.046693 |
| Cbx2 | 12416 | chromobox 2 | 0.83 | 0.046702 |
| Pir | 69656 | pirin | 1.15 | 0.046852 |
| Ccdc137 | 67291 | coiled-coil domain containing 137 | 1.1 | 0.046926 |
| Rbl2 | 19651 | retinoblastoma-like 2 | 1.23 | 0.047049 |
| 2010111I01Rik | 72061 | RIKEN cDNA 2010111I01 gene | 0.85 | 0.047078 |
| Ptpn13 | 19249 | protein tyrosine phosphatase, non-receptor type 13 | 0.87 | 0.047092 |
| Plcb4 | 18798 | phospholipase C, beta 4 | 0.86 | 0.047211 |
| Nr3c2 | 110784 | nuclear receptor subfamily 3, group C, member 2 | 1.63 | 0.04722 |
| Cib2 | 56506 | calcium and integrin binding family member 2 | 0.79 | 0.047244 |
| Gm16039 | 1E+08 | predicted gene 16039 | 0.85 | 0.047246 |
| Romo1 | 67067 | reactive oxygen species modulator 1 | 0.87 | 0.047561 |
| Dapp1 | 26377 | dual adaptor for phosphotyrosine and 3-phosphoinositides 1 | 1.18 | 0.047701 |
| Srgap1 | 117600 | SLIT-ROBO Rho GTPase activating protein 1 | 1.13 | 0.047739 |
| Coro2b | 235431 | coronin, actin binding protein, 2B | 1.27 | 0.047832 |
| Dancr | 70036 | NA | 0.88 | 0.047964 |
| Rbl1 | 19650 | retinoblastoma-like 1 (p107) | 1.19 | 0.047976 |
| Sumf2 | 67902 | sulfatase modifying factor 2 | 1.11 | 0.047977 |
| BC018507 | 218333 | cDNA sequence BC018507 | 1.11 | 0.048011 |
| Palm2 | 242481 | paralemmin 2 | 0.84 | 0.04804 |
| Car5b | 56078 | carbonic anhydrase 5b, mitochondrial | 1.13 | 0.048043 |
| Dennd1a | 227801 | DENN/MADD domain containing 1A | 0.86 | 0.048104 |
| Cacna1a | 12286 | calcium channel, voltage-dependent, P/Q type, alpha 1A subunit | 0.86 | 0.048178 |
| Gm11110 | 1E+08 | predicted gene 11110 | 0.7 | 0.048238 |
| Lrwd1 | 71735 | leucine-rich repeats and WD repeat domain containing 1 | 1.16 | 0.048266 |
| 1810034E14Rik | 66503 | RIKEN cDNA 1810034E14 gene | 0.73 | 0.048305 |
| Fam161a | 73873 | family with sequence similarity 161, member A | 1.22 | 0.048383 |
| Slc35f2 | 72022 | solute carrier family 35, member F2 | 1.13 | 0.048505 |
| Spaca6 | 75202 | NA | 0.76 | 0.048546 |
| Dcp2 | 70640 | DCP2 decapping enzyme homolog (S. cerevisiae) | 0.86 | 0.048626 |
| Pemt | 18618 | phosphatidylethanolamine N-methyltransferase | 0.88 | 0.048654 |
| Arrdc3 | 105171 | arrestin domain containing 3 | 0.81 | 0.048661 |
| Tnfaip8 | 106869 | tumor necrosis factor, alpha-induced protein 8 | 0.86 | 0.048731 |
| 9130011E15Rik | 71617 | RIKEN cDNA 9130011E15 gene | 0.86 | 0.048912 |
| Tmem2 | 83921 | transmembrane protein 2 | 0.91 | 0.048928 |
| Map2k3os | 24082 | NA | 1.17 | 0.048933 |
| Ebf3 | 13593 | early B cell factor 3 | 0.85 | 0.049069 |
| Vamp3 | 22319 | vesicle-associated membrane protein 3 | 0.88 | 0.049131 |
| Smarca2 | 67155 | SWI/SNF related, matrix associated, actin dependent regulator of chromatin, subfamily a, member 2 | 0.89 | 0.049133 |
| Ing4 | 28019 | inhibitor of growth family, member 4 | 0.89 | 0.049134 |
| Gpatch2 | 67769 | G patch domain containing 2 | 0.89 | 0.049227 |
| Tead1 | 21676 | TEA domain family member 1 | 0.88 | 0.049324 |
| Pdcd4 | 18569 | programmed cell death 4 | 0.86 | 0.049422 |
| Glcci1 | 170772 | glucocorticoid induced transcript 1 | 1.12 | 0.049471 |
| 2610203C20Rik | 1E+08 | RIKEN cDNA 2610203C20 gene | 0.74 | 0.049534 |
| Shisa4 | 77552 | shisa homolog 4 (Xenopus laevis) | 0.73 | 0.04962 |
| Il6ra | 16194 | interleukin 6 receptor, alpha | 1.14 | 0.049645 |
| Zmynd8 | 228880 | zinc finger, MYND-type containing 8 | 1.11 | 0.049796 |
| 4933436C20Rik | NA | RIKEN cDNA 4933436C20 gene | 1.23 | 0.049796 |
| Rgcc | 66214 | NA | 1.25 | 0.049902 |
