# Supplementary material for: Anti-miR-17 therapy delays tumorigenesis in MYC-driven hepatocellular carcinoma (HCC)
Source: Oncotarget. 2017 Nov 9;9(5):5517–28. doi: 10.18632/oncotarget.22342 (PMC5814155; doi:10.18632/oncotarget.22342)
Supplement: Supplementary file 3 [file oncotarget-09-5517-s003.docx]

**Supplementary Table 2: Genes Identified as Potential miR-17 targets in MYC conditional cell lines**

|  |  |  | **TargetScan Conserved Targets miR-17-5p** | | | | | | | **miR-17-5p 3'UTR Seed Matches** | | | | |
| --- | --- | --- | --- | --- | --- | --- | --- | --- | --- | --- | --- | --- | --- | --- |
| **Gene_symbol** | **EntrezID** | **Gene_description** | **TargetScan_mir.17.5p** | **total_TSCAN_mir.17.5p** | **X8mer_TSCAN_mir.17.5p** | **X7mer.m8_TSCAN_mir.17.5p** | **X7mer.1A_TSCAN_mir.17.5p** | **Total.context..score_TSCAN_mir.17.5p** | **Aggregate.PCT_TSCAN_mir.17.5p** | **X6mer2_7_UTR_mir.17.5p** | **X6mer3_8_UTR_mir.17.5p** | **X7mer_m8_UTR_mir.17.5p** | **X7mer_A1_UTR_mir.17.5p** | **X8mer_UTR_mir.17.5p** |
| Mfap3l | 71306 | microfibrillar-associated protein 3-like | Y | 2 | 1 | 1 | 0 | -0.255 | 0.996 | Y | Y | Y | N | Y |
| Sema7a | 20361 | sema domain, immunoglobulin domain (Ig), and GPI membrane anchor, (semaphorin) 7A | Y | 1 | 1 | 0 | 0 | -0.36 | 0.93 | N | N | N | N | Y |
| Znfx1 | 98999 | zinc finger, NFX1-type containing 1 | Y | 2 | 2 | 0 | 0 | -0.631 | 0.988 | Y | Y | N | Y | Y |
| Polr3g | 67486 | polymerase (RNA) III (DNA directed) polypeptide G | Y | 1 | 1 | 0 | 0 | -0.267 | 0.961 | Y | N | N | Y | Y |
| Polq | 77782 | polymerase (DNA directed), theta | Y | 1 | 0 | 1 | 0 | -0.173 | 0.47 | Y | Y | Y | N | N |
| Clip4 | 78785 | CAP-GLY domain containing linker protein family, member 4 | Y | 2 | 0 | 2 | 0 | -0.52 | 0.862 | Y | Y | Y | Y | Y |
| D030056L22Rik | 225995 | RIKEN cDNA D030056L22 gene | Y | 1 | 1 | 0 | 0 | -0.275 | 0 | N | Y | N | N | Y |
| Ficd | 231630 | FIC domain containing | Y | 1 | 1 | 0 | 0 | -0.396 | 0.96 | Y | Y | Y | N | Y |
| Slc36a1 | 215335 | solute carrier family 36 (proton/amino acid symporter), member 1 | Y | 1 | 0 | 0 | 1 | -0.133 | 0 | Y | Y | N | Y | Y |
| Fjx1 | 14221 | four jointed box 1 (Drosophila) | Y | 1 | 1 | 0 | 0 | -0.328 | 0.931 | N | N | N | N | Y |
| Slc25a40 | 319653 | solute carrier family 25, member 40 | Y | 1 | 1 | 0 | 0 | -0.368 | 0.922 | Y | N | N | Y | Y |
| Mink1 | 50932 | misshapen-like kinase 1 (zebrafish) | Y | 1 | 1 | 0 | 0 | -0.259 | 0.87 | N | N | N | N | Y |
| Pkia | 18767 | protein kinase inhibitor, alpha | Y | 1 | 0 | 1 | 0 | -0.12 | 0.889 | Y | Y | Y | N | N |
| Fyco1 | 17281 | FYVE and coiled-coil domain containing 1 | Y | 1 | 1 | 0 | 0 | -0.435 | 0.924 | Y | Y | Y | N | Y |
| Plekha3 | 83435 | pleckstrin homology domain-containing, family A (phosphoinositide binding specific) member 3 | Y | 2 | 2 | 0 | 0 | -0.446 | 0.985 | Y | N | N | N | Y |
| Tgfbr2 | 21813 | transforming growth factor, beta receptor II | Y | 1 | 1 | 0 | 0 | -0.305 | 0.967 | Y | Y | N | N | Y |
| Agfg2 | 231801 | ArfGAP with FG repeats 2 | Y | 1 | 1 | 0 | 0 | -0.254 | 0.963 | N | Y | N | N | Y |
| Itgb8 | 320910 | integrin beta 8 | Y | 1 | 1 | 0 | 0 | -0.406 | 0.78 | Y | Y | Y | Y | Y |
| Ereg | 13874 | epiregulin | Y | 1 | 0 | 1 | 0 | -0.293 | 0.876 | Y | Y | Y | N | N |
| F3 | 14066 | coagulation factor III | Y | 1 | 1 | 0 | 0 | -0.293 | 0.921 | N | N | N | N | Y |
| Ankrd9 | 74251 | ankyrin repeat domain 9 | Y | 1 | 1 | 0 | 0 | -0.287 | 0.88 | N | N | N | N | Y |
| Epha4 | 13838 | Eph receptor A4 | Y | 2 | 1 | 1 | 0 | -0.554 | 0.977 | Y | Y | Y | N | Y |
| Cc2d1a | 212139 | coiled-coil and C2 domain containing 1A | Y | 1 | 1 | 0 | 0 | -0.211 | 0.844 | N | N | N | N | Y |
| Ezh1 | 14055 | enhancer of zeste homolog 1 (Drosophila) | Y | 2 | 2 | 0 | 0 | -0.45 | 0.998 | Y | N | N | Y | Y |
| BC037034 | 231807 | cDNA sequence BC037034 | Y | 1 | 1 | 0 | 0 | -0.298 | 0.947 | N | Y | N | N | Y |
| Atg16l1 | 77040 | autophagy related 16-like 1 (S. cerevisiae) | Y | 1 | 1 | 0 | 0 | -0.331 | 0.961 | N | N | N | N | Y |
| Slc40a1 | 53945 | solute carrier family 40 (iron-regulated transporter), member 1 | Y | 2 | 0 | 2 | 0 | -0.297 | 0.954 | Y | Y | Y | N | N |
| Zfp367 | 238673 | zinc finger protein 367 | Y | 2 | 0 | 2 | 0 | -0.366 | 0.695 | Y | Y | Y | N | N |
| Ppp1r3e | 105651 | protein phosphatase 1, regulatory (inhibitor) subunit 3E | Y | 1 | 1 | 0 | 0 | -0.306 | 0.777 | N | N | N | N | Y |
| Slc29a2 | 13340 | solute carrier family 29 (nucleoside transporters), member 2 | Y | 1 | 1 | 0 | 0 | -0.245 | 0.018 | N | Y | N | N | Y |
| Nagk | 56174 | N-acetylglucosamine kinase | Y | 1 | 1 | 0 | 0 | -0.343 | 0.57 | N | N | N | N | Y |
| Zfp651 | 270210 | zinc finger protein 651 | Y | 1 | 1 | 0 | 0 | -0.043 | 0.967 | N | N | N | N | Y |
| Ptpn4 | 19258 | protein tyrosine phosphatase, non-receptor type 4 | Y | 1 | 1 | 0 | 0 | -0.361 | 0.744 | Y | N | N | N | Y |
| Rasa2 | 114713 | RAS p21 protein activator 2 | Y | 1 | 1 | 0 | 0 | -0.06 | 0.964 | N | Y | N | N | Y |
| Rgs17 | 56533 | regulator of G-protein signaling 17 | Y | 3 | 1 | 2 | 0 | -0.08 | 0.992 | Y | Y | Y | Y | Y |
| Snx8 | 231834 | sorting nexin 8 | Y | 1 | 1 | 0 | 0 | -0.27 | 0.966 | N | N | N | N | Y |
| Gpr137b | 83924 | G protein-coupled receptor 137B | Y | 1 | 0 | 0 | 1 | -0.077 | 0.512 | Y | N | N | Y | N |
| Cdkn1a | 12575 | cyclin-dependent kinase inhibitor 1A (P21) | Y | 1 | 0 | 1 | 0 | -0.112 | 0.813 | Y | Y | Y | N | N |
| Rgmb | 68799 | RGM domain family, member B | Y | 1 | 1 | 0 | 0 | -0.306 | 0.967 | N | Y | N | N | Y |
| Atp1a2 | 98660 | ATPase, Na+/K+ transporting, alpha 2 polypeptide | Y | 1 | 1 | 0 | 0 | -0.048 | 0.623 | Y | N | N | Y | Y |
| Sema4b | 20352 | sema domain, immunoglobulin domain (Ig), transmembrane domain (TM) and short cytoplasmic domain, (semaphorin) 4B | Y | 1 | 1 | 0 | 0 | -0.33 | 0.943 | Y | N | N | N | Y |
| Arhgef10 | 234094 | Rho guanine nucleotide exchange factor (GEF) 10 | Y | 1 | 1 | 0 | 0 | -0.298 | 0.943 | N | N | N | N | Y |
| Cdc37l1 | 67072 | cell division cycle 37-like 1 | Y | 1 | 1 | 0 | 0 | -0.288 | 0.967 | Y | N | N | N | Y |
| P2rx4 | 18438 | purinergic receptor P2X, ligand-gated ion channel 4 | Y | 1 | 1 | 0 | 0 | -0.271 | 0.85 | N | N | N | N | Y |
| Rhoc | 11853 | ras homolog gene family, member C | Y | 1 | 1 | 0 | 0 | -0.302 | 0.508 | N | N | N | N | Y |
| Slc22a23 | 73102 | solute carrier family 22, member 23 | Y | 1 | 1 | 0 | 0 | -0.257 | 0.967 | Y | Y | N | N | Y |
| Smoc2 | 64074 | SPARC related modular calcium binding 2 | Y | 1 | 0 | 1 | 0 | -0.231 | 0.904 | Y | Y | Y | N | N |
| Lace1 | 215951 | lactation elevated 1 | Y | 1 | 0 | 1 | 0 | -0.281 | 0.905 | Y | Y | Y | N | Y |
| Mtf1 | 17764 | metal response element binding transcription factor 1 | Y | 1 | 1 | 0 | 0 | -0.03 | 0.945 | Y | Y | N | N | Y |
| Ddhd1 | 114874 | DDHD domain containing 1 | Y | 1 | 1 | 0 | 0 | -0.388 | 0.897 | Y | Y | N | N | Y |
| Rufy2 | 70432 | RUN and FYVE domain-containing 2 | Y | 1 | 1 | 0 | 0 | -0.302 | 0.965 | Y | Y | N | Y | Y |
| Arhgef18 | 102098 | rho/rac guanine nucleotide exchange factor (GEF) 18 | Y | 1 | 1 | 0 | 0 | -0.213 | 0.959 | N | Y | N | N | Y |
| Clock | 12753 | circadian locomotor output cycles kaput | Y | 2 | 1 | 1 | 0 | -0.268 | 0.993 | Y | Y | Y | N | Y |
| Grhl2 | 252973 | grainyhead-like 2 (Drosophila) | Y | 1 | 0 | 1 | 0 | -0.02 | 0.64 | Y | Y | Y | N | N |
| E2f1 | 13555 | E2F transcription factor 1 | Y | 2 | 0 | 2 | 0 | -0.181 | 0.889 | Y | Y | Y | N | N |
| Rcan3 | 53902 | regulator of calcineurin 3 | Y | 1 | 1 | 0 | 0 | -0.231 | 0.966 | Y | N | N | N | Y |
| Txnip | 56338 | thioredoxin interacting protein | Y | 2 | 1 | 1 | 0 | -0.301 | 0.974 | Y | Y | Y | N | Y |
| Mapre3 | 100732 | microtubule-associated protein, RP/EB family, member 3 | Y | 1 | 1 | 0 | 0 | -0.154 | 0.844 | Y | N | N | N | Y |
| Camk2n2 | 73047 | calcium/calmodulin-dependent protein kinase II inhibitor 2 | Y | 1 | 1 | 0 | 0 | -0.216 | 0.473 | N | N | N | N | Y |
| Chd5 | 269610 | chromodomain helicase DNA binding protein 5 | Y | 1 | 0 | 1 | 0 | -0.087 | 0.896 | Y | Y | Y | N | N |
| Snx16 | 74718 | sorting nexin 16 | Y | 1 | 1 | 0 | 0 | -0.299 | 0.769 | N | N | N | N | Y |
| Tusc2 | 80385 | tumor suppressor candidate 2 | Y | 1 | 1 | 0 | 0 | -0.169 | 0.888 | Y | N | N | N | Y |
| Slc30a1 | 22782 | solute carrier family 30 (zinc transporter), member 1 | Y | 1 | 0 | 1 | 0 | -0.04 | 0.84 | Y | Y | Y | N | N |
| Ldlrap1 | 100017 | low density lipoprotein receptor adaptor protein 1 | Y | 1 | 1 | 0 | 0 | -0.182 | 0.963 | Y | Y | N | N | Y |
| Il17rd | 171463 | interleukin 17 receptor D | Y | 1 | 0 | 0 | 1 | -0.01 | 0.516 | Y | Y | N | Y | N |
| Mier2 | 70427 | mesoderm induction early response 1, family member 2 | Y | 1 | 1 | 0 | 0 | -0.092 | 0.938 | N | N | N | N | Y |
| Fbxl5 | 242960 | F-box and leucine-rich repeat protein 5 | Y | 1 | 1 | 0 | 0 | -0.306 | 0.876 | N | Y | N | N | Y |
| Rps6ka4 | 56613 | ribosomal protein S6 kinase, polypeptide 4 | Y | 1 | 0 | 0 | 1 | -0.01 | 0.476 | Y | N | N | Y | N |
| Vash2 | 226841 | vasohibin 2 | Y | 1 | 1 | 0 | 0 | -0.221 | 0.957 | Y | N | N | N | Y |
| Rgma | 244058 | RGM domain family, member A | Y | 1 | 1 | 0 | 0 | -0.309 | 0.967 | N | Y | N | N | Y |
| Mfn2 | 170731 | mitofusin 2 | Y | 1 | 1 | 0 | 0 | -0.373 | 0.964 | Y | Y | Y | N | Y |
| Pkd2 | 18764 | polycystic kidney disease 2 | Y | 1 | 1 | 0 | 0 | -0.326 | 0.965 | N | N | N | N | Y |
| Arl4c | 320982 | ADP-ribosylation factor-like 4C | Y | 1 | 0 | 1 | 0 | -0.137 | 0.846 | Y | Y | Y | N | N |
| Dusp2 | 13537 | dual specificity phosphatase 2 | Y | 1 | 1 | 0 | 0 | -0.312 | 0.938 | N | N | N | N | Y |
| Zbtb7a | 16969 | zinc finger and BTB domain containing 7a | Y | 2 | 0 | 2 | 0 | -0.111 | 0.975 | Y | Y | Y | N | N |
| Tnks1bp1 | 228140 | tankyrase 1 binding protein 1 | Y | 1 | 0 | 1 | 0 | -0.179 | 0.826 | Y | Y | Y | N | N |
| Ankrd13c | 433667 | ankyrin repeat domain 13c | Y | 1 | 1 | 0 | 0 | -0.309 | 0.707 | N | N | N | N | Y |
| Gnb5 | 14697 | guanine nucleotide binding protein (G protein), beta 5 | Y | 1 | 1 | 0 | 0 | -0.223 | 0.556 |  |  |  |  |  |
| Tbc1d9 | 71310 | TBC1 domain family, member 9 | Y | 2 | 0 | 1 | 1 | -0.133 | 0.95 | Y | Y | Y | Y | N |
| Zfyve26 | 211978 | zinc finger, FYVE domain containing 26 | Y | 1 | 1 | 0 | 0 | -0.296 | 0.95 | N | Y | N | N | Y |
| Cd274 | 60533 | CD274 antigen | Y | 1 | 1 | 0 | 0 | -0.26 | 0.863 | Y | N | N | N | Y |
| Crot | 74114 | carnitine O-octanoyltransferase | Y | 1 | 1 | 0 | 0 | -0.269 | 0.928 | N | N | N | N | Y |
| Gpr137b_ps | 664862 | NA | Y | NA | NA | NA | NA | NA | NA |  |  |  |  |  |
| Klf11 | 194655 | Kruppel-like factor 11 | Y | 1 | 1 | 0 | 0 | -0.215 | 0.955 | N | N | N | N | Y |
| Cry2 | 12953 | cryptochrome 2 (photolyase-like) | Y | 1 | 1 | 0 | 0 | -0.273 | 0.907 | Y | Y | N | N | Y |
| Lif | 16878 | leukemia inhibitory factor | Y | 1 | 1 | 0 | 0 | -0.04 | 0.956 | Y | N | N | N | Y |
| Itpripl2 | 319622 | inositol 1,4,5-triphosphate receptor interacting protein-like 2 | Y | 2 | 1 | 1 | 0 | -0.251 | 0.908 | Y | Y | Y | N | Y |
| Klhl28 | 66689 | kelch-like 28 (Drosophila) | Y | 1 | 0 | 1 | 0 | -0.134 | 0.243 | Y | Y | Y | N | N |
| Trip10 | 106628 | thyroid hormone receptor interactor 10 | Y | 1 | 1 | 0 | 0 | -0.226 | 0.964 | N | N | N | N | Y |
| Kat2b | 18519 | K(lysine) acetyltransferase 2B | Y | 1 | 0 | 1 | 0 | -0.211 | 0.572 | Y | Y | Y | N | N |
| Fam134a | 227298 | family with sequence similarity 134, member A | Y | 1 | 1 | 0 | 0 | -0.238 | 0.933 | Y | Y | Y | N | Y |
| Wee1 | 22390 | WEE 1 homolog 1 (S. pombe) | Y | 1 | 1 | 0 | 0 | -0.14 | 0.768 |  |  |  |  |  |
| Rab11fip5 | 52055 | RAB11 family interacting protein 5 (class I) | Y | 1 | 1 | 0 | 0 | -0.329 | 0.966 | N | N | N | N | Y |
| Ube2j1 | 56228 | ubiquitin-conjugating enzyme E2J 1 | Y | 1 | 1 | 0 | 0 | -0.284 | 0.791 | Y | N | N | N | Y |
| Limk1 | 16885 | LIM-domain containing, protein kinase | Y | 1 | 0 | 1 | 0 | -0.138 | 0.881 | Y | Y | Y | Y | N |
| Fbxl3 | 50789 | F-box and leucine-rich repeat protein 3 | Y | 1 | 0 | 0 | 1 | -0.01 | 0.411 | Y | Y | N | Y | N |
| Vldlr | 22359 | very low density lipoprotein receptor | Y | 2 | 0 | 2 | 0 | -0.326 | 0.991 | Y | Y | Y | N | N |
| Wfs1 | 22393 | Wolfram syndrome 1 homolog (human) | Y | 1 | 1 | 0 | 0 | -0.253 | 0.892 | Y | N | N | N | Y |
| Pxk | 218699 | PX domain containing serine/threonine kinase | Y | 1 | 1 | 0 | 0 | -0.361 | 0.959 | N | N | N | N | Y |
| Dusp8 | 18218 | dual specificity phosphatase 8 | Y | 1 | 0 | 1 | 0 | -0.145 | 0.884 | Y | Y | Y | N | N |
| Arhgef3 | 71704 | Rho guanine nucleotide exchange factor (GEF) 3 | Y | 1 | 0 | 1 | 0 | -0.113 | 0.906 | Y | Y | Y | N | N |
| Fam13c | 71721 | family with sequence similarity 13, member C | Y | 1 | 1 | 0 | 0 | -0.31 | 0.492 | N | N | N | N | Y |
| Atxn1 | 20238 | ataxin 1 | Y | 1 | 0 | 1 | 0 | -0.04 | 0.893 | Y | Y | Y | N | N |
| Pkd1 | 18763 | polycystic kidney disease 1 homolog | Y | 1 | 1 | 0 | 0 | -0.233 | 0.928 | N | N | N | N | Y |
| Tmem64 | 100201 | transmembrane protein 64 | Y | 1 | 1 | 0 | 0 | -0.258 | 0.965 | Y | Y | N | N | Y |
| Hn1 | 15374 | hematological and neurological expressed sequence 1 | Y | 1 | 1 | 0 | 0 | -0.279 | 0.962 | N | Y | N | N | Y |
| Map3k5 | 26408 | mitogen-activated protein kinase kinase kinase 5 | Y | 1 | 1 | 0 | 0 | -0.257 | 0.966 | N | N | N | N | Y |
| Rasl11b | 68939 | RAS-like, family 11, member B | Y | 1 | 1 | 0 | 0 | -0.298 | 0.956 | N | N | N | N | Y |
| St6galnac6 | 50935 | ST6 (alpha-N-acetyl-neuraminyl-2,3-beta-galactosyl-1,3)-N-acetylgalactosaminide alpha-2,6-sialyltransferase 6 | Y | 1 | 1 | 0 | 0 | -0.256 | 0.75 | N | N | N | N | Y |
| Neto2 | 74513 | neuropilin (NRP) and tolloid (TLL)-like 2 | Y | 1 | 0 | 1 | 0 | -0.334 | 0.537 | Y | Y | Y | N | Y |
| Tor1b | 30934 | torsin family 1, member B | Y | 1 | 0 | 1 | 0 | -0.117 | 0.846 | Y | Y | Y | Y | N |
| Trim36 | 28105 | tripartite motif-containing 36 | Y | 1 | 1 | 0 | 0 | -0.252 | 0.961 | N | Y | N | N | Y |
| Tbc1d8b | 245638 | TBC1 domain family, member 8B | Y | 1 | 0 | 1 | 0 | -0.311 | 0.366 | Y | Y | Y | Y | N |
| Slc16a6 | 104681 | solute carrier family 16 (monocarboxylic acid transporters), member 6 | Y | 1 | 1 | 0 | 0 | -0.225 | 0.959 | N | N | N | N | Y |
| Midn | 59090 | midnolin | Y | 1 | 1 | 0 | 0 | -0.187 | 0.611 | N | N | N | N | Y |
| Apcdd1 | 494504 | adenomatosis polyposis coli down-regulated 1 | Y | 1 | 1 | 0 | 0 | -0.243 | 0.967 | Y | Y | N | N | Y |
| Map3k12 | 26404 | mitogen-activated protein kinase kinase kinase 12 | Y | 1 | 0 | 1 | 0 | -0.205 | 0.855 | Y | Y | Y | Y | N |
| Taok2 | 381921 | TAO kinase 2 | Y | 1 | 1 | 0 | 0 | -0.333 | 0.679 | N | N | N | N | N |
| Ankib1 | 70797 | ankyrin repeat and IBR domain containing 1 | Y | 2 | 0 | 1 | 1 | -0.182 | 0.856 | Y | Y | Y | Y | N |
| Cep120 | 225523 | centrosomal protein 120 | Y | 1 | 1 | 0 | 0 | -0.247 | 0.796 | N | N | N | N | Y |
| Sik1 | 17691 | salt inducible kinase 1 | Y | 1 | 1 | 0 | 0 | -0.209 | 0.95 | N | N | N | N | Y |
| Cep97 | 74201 | centrosomal protein 97 | Y | 1 | 1 | 0 | 0 | -0.371 | 0.967 | Y | Y | N | Y | Y |
| Mastl | 67121 | microtubule associated serine/threonine kinase-like | Y | 1 | 1 | 0 | 0 | -0.304 | 0.966 | Y | N | N | N | Y |
| Coro2b | 235431 | coronin, actin binding protein, 2B | Y | 1 | 1 | 0 | 0 | -0.198 | 0.654 | Y | N | N | N | Y |
| Smad7 | 17131 | SMAD family member 7 | Y | 1 | 0 | 1 | 0 | -0.177 | 0.9 | Y | Y | Y | N | N |
| D4Wsu53e | 27981 | DNA segment, Chr 4, Wayne State University 53, expressed | Y | 1 | 1 | 0 | 0 | -0.257 | 0.662 | N | N | N | N | Y |
| Rps6ka5 | 73086 | ribosomal protein S6 kinase, polypeptide 5 | Y | 1 | 1 | 0 | 0 | -0.289 | 0.274 | Y | Y | Y | N | Y |
| Map3k2 | 26405 | mitogen-activated protein kinase kinase kinase 2 | Y | 4 | 1 | 2 | 1 | -0.519 | 1 | Y | Y | Y | Y | Y |
| Fastk | 66587 | Fas-activated serine/threonine kinase | Y | 1 | 0 | 1 | 0 | -0.161 | 0.467 | Y | Y | Y | N | N |
| Pfkp | 56421 | phosphofructokinase, platelet | Y | 1 | 1 | 0 | 0 | -0.305 | 0.967 | N | Y | N | N | Y |
| Sorl1 | 20660 | sortilin-related receptor, LDLR class A repeats-containing | Y | 1 | 0 | 1 | 0 | -0.196 | 0.756 | Y | Y | Y | Y | N |
| Atxn1l | 52335 | ataxin 1-like | Y | 2 | 1 | 1 | 0 | -0.225 | 0.981 | Y | Y | Y | N | Y |
| Tgoln1 | 22134 | trans-golgi network protein | Y | 1 | 0 | 1 | 0 | -0.123 | 0.904 | Y | Y | Y | N | N |
| Syap1 | 67043 | synapse associated protein 1 | Y | 1 | 0 | 1 | 0 | -0.39 | 0.364 | Y | Y | Y | N | N |
| Napepld | 242864 | N-acyl phosphatidylethanolamine phospholipase D | Y | 1 | 1 | 0 | 0 | -0.214 | 0 | Y | Y | N | Y | Y |
| Plekhm1 | 353047 | pleckstrin homology domain containing, family M (with RUN domain) member 1 | Y | 1 | 0 | 1 | 0 | -0.075 | 0.876 | Y | Y | Y | N | N |
| Rab11fip4 | 268451 | RAB11 family interacting protein 4 (class II) | Y | 1 | 0 | 1 | 0 | -0.094 | 0.85 | Y | Y | Y | Y | N |
| Tada2b | 231151 | transcriptional adaptor 2B | Y | 1 | 1 | 0 | 0 | -0.046 | 0.871 | N | N | N | N | Y |
| Hmga2_ps1 | 15365 | NA | Y | NA | NA | NA | NA | NA | NA |  |  |  |  |  |
| Fam160b2 | 239170 | family with sequence similarity 160, member B2 | Y | 1 | 0 | 1 | 0 | -0.153 | 0.311 | Y | Y | Y | N | N |
| Celsr2 | 53883 | cadherin, EGF LAG seven-pass G-type receptor 2 (flamingo homolog, Drosophila) | Y | 1 | 0 | 1 | 0 | -0.095 | 0.418 | Y | Y | Y | N | N |
| Ube2w | 66799 | ubiquitin-conjugating enzyme E2W (putative) | Y | 1 | 0 | 1 | 0 | -0.173 | 0.733 | Y | Y | Y | N | N |
| Sumf1 | 58911 | sulfatase modifying factor 1 | Y | 1 | 0 | 1 | 0 | -0.108 | 0.896 | Y | Y | Y | N | N |
| Tnfaip1 | 21927 | tumor necrosis factor, alpha-induced protein 1 (endothelial) | Y | 1 | 0 | 1 | 0 | -0.092 | 0.786 | Y | Y | Y | N | N |
| Atg2b | 76559 | autophagy related 2B | Y | 1 | 1 | 0 | 0 | -0.19 | 0.301 | N | Y | N | N | Y |
| Yes1 | 22612 | Yamaguchi sarcoma viral (v-yes) oncogene homolog 1 | Y | 1 | 0 | 1 | 0 | -0.067 | 0.364 | Y | Y | Y | N | N |
| E2f5 | 13559 | E2F transcription factor 5 | Y | 1 | 1 | 0 | 0 | -0.33 | 0.731 | Y | N | N | N | Y |
| Kif23 | 71819 | kinesin family member 23 | Y | 1 | 1 | 0 | 0 | -0.351 | 0.907 | Y | N | N | N | Y |
| Fam60a | 56306 | family with sequence similarity 60, member A | Y | 1 | 0 | 1 | 0 | -0.196 | 0.853 | Y | Y | Y | N | N |
| Csf1 | 12977 | colony stimulating factor 1 (macrophage) | Y | 1 | 0 | 1 | 0 | -0.035 | 0.594 | Y | Y | Y | N | N |
| Fanci | 208836 | Fanconi anemia, complementation group I | Y | 1 | 0 | 1 | 0 | -0.198 | 0.417 | Y | Y | Y | N | N |
| Fam45a | 67894 | family with sequence similarity 45, member A | Y | 2 | 1 | 1 | 0 | -0.564 | 0.986 | Y | Y | Y | N | Y |
| Rab22a | 19334 | RAB22A, member RAS oncogene family | Y | 2 | 0 | 2 | 0 | -0.189 | 0.951 | Y | Y | Y | N | N |
| Tmem127 | 69470 | transmembrane protein 127 | Y | 1 | 1 | 0 | 0 | -0.13 | 0.95 | Y | N | N | N | Y |
| Vangl1 | 229658 | vang-like 1 (van gogh, Drosophila) | Y | 1 | 1 | 0 | 0 | -0.264 | 0.966 | Y | N | N | Y | Y |
| Bmp2 | 12156 | bone morphogenetic protein 2 | Y | 1 | 0 | 0 | 1 | -0.043 | 0.276 | Y | N | N | Y | N |
| Slc12a7 | 20499 | solute carrier family 12, member 7 | Y | 1 | 1 | 0 | 0 | -0.164 | 0.345 | Y | Y | Y | Y | Y |
| Runx1 | 12394 | runt related transcription factor 1 | Y | 1 | 0 | 1 | 0 | -0.02 | 0.903 | Y | Y | Y | N | N |
| Rbl2 | 19651 | retinoblastoma-like 2 | Y | 2 | 1 | 1 | 0 | -0.132 | 0.995 | Y | Y | Y | N | Y |
| Twf1 | 19230 | twinfilin, actin-binding protein, homolog 1 (Drosophila) | Y | 1 | 0 | 1 | 0 | -0.21 | 0.626 | Y | Y | Y | N | N |
| Dcaf8 | 98193 | DDB1 and CUL4 associated factor 8 | Y | 1 | 0 | 1 | 0 | -0.172 | 0.823 | Y | Y | Y | N | N |
| Osr1 | 23967 | odd-skipped related 1 (Drosophila) | Y | 1 | 1 | 0 | 0 | -0.218 | 0.966 | N | N | N | N | Y |
| Tmcc1 | 330401 | transmembrane and coiled coil domains 1 | Y | 2 | 0 | 2 | 0 | -0.167 | 0.873 | Y | Y | Y | Y | N |
| Dip2a | 64451 | DIP2 disco-interacting protein 2 homolog A (Drosophila) | Y | 1 | 0 | 1 | 0 | -0.208 | 0.327 | Y | Y | Y | N | N |
| Sqstm1 | 18412 | sequestosome 1 | Y | 1 | 1 | 0 | 0 | -0.352 | 0.647 | N | N | N | N | Y |
| Egln3 | 112407 | EGL nine homolog 3 (C. elegans) | Y | 1 | 1 | 0 | 0 | -0.206 | 0.964 | N | N | N | N | Y |
| Klf10 | 21847 | Kruppel-like factor 10 | Y | 1 | 0 | 1 | 0 | -0.12 | 0.851 | Y | Y | Y | N | N |
| Usp46 | 69727 | ubiquitin specific peptidase 46 | Y | 1 | 1 | 0 | 0 | -0.267 | 0.827 | Y | Y | Y | Y | Y |
| Phlpp2 | 244650 | PH domain and leucine rich repeat protein phosphatase 2 | Y | 1 | 0 | 1 | 0 | -0.131 | 0.899 | Y | Y | Y | N | N |
| M6pr | 17113 | mannose-6-phosphate receptor, cation dependent | Y | 2 | 1 | 1 | 0 | -0.117 | 0.3 | Y | Y | Y | N | Y |
| Lpgat1 | 226856 | lysophosphatidylglycerol acyltransferase 1 | Y | 1 | 0 | 1 | 0 | -0.164 | 0.893 | Y | Y | Y | N | Y |
| Uevld | 54122 | UEV and lactate/malate dehyrogenase domains | Y | 2 | 1 | 1 | 0 | -0.237 | 0.876 | Y | Y | Y | N | Y |
| Laptm4a | 17775 | lysosomal-associated protein transmembrane 4A | Y | 1 | 0 | 1 | 0 | -0.217 | 0.463 | Y | Y | Y | N | N |
| Fbxo31 | 76454 | F-box protein 31 | Y | 1 | 1 | 0 | 0 | -0.241 | 0.81 | Y | N | N | N | Y |
| Camta2 | 216874 | calmodulin binding transcription activator 2 | Y | 1 | 0 | 1 | 0 | -0.129 | 0.569 | Y | Y | Y | N | N |
| Abhd2 | 54608 | abhydrolase domain containing 2 | Y | 1 | 0 | 1 | 0 | -0.161 | 0.107 | Y | Y | Y | N | Y |
| Rab5b | 19344 | RAB5B, member RAS oncogene family | Y | 2 | 1 | 1 | 0 | -0.237 | 0.964 | Y | Y | Y | N | Y |
| Chd9 | 109151 | chromodomain helicase DNA binding protein 9 | Y | 2 | 0 | 2 | 0 | -0.195 | 0.748 | Y | Y | Y | N | N |
| Arhgap1 | 228359 | Rho GTPase activating protein 1 | Y | 1 | 0 | 1 | 0 | -0.299 | 0.891 | Y | Y | Y | N | N |
| Nup35 | 69482 | nucleoporin 35 | Y | 1 | 1 | 0 | 0 | -0.296 | 0.966 | N | N | N | N | Y |
| Cntnap1 | 53321 | contactin associated protein-like 1 | Y | 1 | 1 | 0 | 0 | -0.191 | 0 | N | N | N | N | Y |
| Iqsec2 | 245666 | IQ motif and Sec7 domain 2 | Y | 1 | 1 | 0 | 0 | -0.182 | 0.657 | N | Y | N | N | Y |
| Slc41a1 | 98396 | solute carrier family 41, member 1 | Y | 1 | 0 | 1 | 0 | -0.263 | 0.7 | Y | Y | Y | N | N |
| Scamp5 | 56807 | secretory carrier membrane protein 5 | Y | 1 | 0 | 1 | 0 | -0.156 | 0.543 | Y | Y | Y | N | N |
| Tnfrsf21 | 94185 | tumor necrosis factor receptor superfamily, member 21 | Y | 1 | 1 | 0 | 0 | -0.463 | 0.394 | Y | N | N | N | Y |
| Rnf6 | 74132 | ring finger protein (C3H2C3 type) 6 | Y | 1 | 0 | 1 | 0 | -0.172 | 0.812 | Y | Y | Y | N | N |
| Bicd2 | 76895 | bicaudal D homolog 2 (Drosophila) | Y | 1 | 0 | 1 | 0 | -0.162 | 0.754 | Y | Y | Y | N | N |
| Rbl1 | 19650 | retinoblastoma-like 1 (p107) | Y | 1 | 0 | 1 | 0 | -0.161 | 0.65 | Y | Y | Y | N | N |
| Trip11 | 109181 | thyroid hormone receptor interactor 11 | Y | 1 | 1 | 0 | 0 | -0.352 | 0.857 | Y | Y | N | Y | Y |
| Zhx2 | 387609 | zinc fingers and homeoboxes 2 | Y | 1 | 0 | 1 | 0 | -0.204 | 0.844 | Y | Y | Y | Y | N |
| Irf9 | 16391 | interferon regulatory factor 9 | Y | 1 | 1 | 0 | 0 | -0.291 | 0.956 | N | Y | N | N | Y |
| Trim3 | 55992 | tripartite motif-containing 3 | Y | 1 | 1 | 0 | 0 | -0.298 | 0.908 | N | N | N | N | Y |
| Vegfa | 22339 | vascular endothelial growth factor A | Y | 1 | 0 | 1 | 0 | -0.047 | 0.575 | Y | Y | Y | N | N |
| Gosr1 | 53334 | golgi SNAP receptor complex member 1 | Y | 1 | 0 | 1 | 0 | -0.213 | 0.891 | Y | Y | Y | N | N |
| Derl2 | 116891 | Der1-like domain family, member 2 | Y | 1 | 1 | 0 | 0 | -0.266 | 0.967 | N | N | N | N | Y |
| Ormdl3 | 66612 | ORM1-like 3 (S. cerevisiae) | Y | 1 | 1 | 0 | 0 | -0.158 | 0.935 | N | N | N | N | Y |
| Il6st | 16195 | interleukin 6 signal transducer | Y | 1 | 1 | 0 | 0 | -0.099 | 0.934 | N | Y | N | N | Y |
| Aktip | 14339 | thymoma viral proto-oncogene 1 interacting protein | Y | 1 | 0 | 1 | 0 | -0.176 | 0.049 | Y | Y | Y | N | N |
| C330007P06Rik | 77644 | RIKEN cDNA C330007P06 gene | Y | 1 | 1 | 0 | 0 | -0.145 | 0 | Y | Y | Y | Y | Y |
| Mex3d | 237400 | mex3 homolog D (C. elegans) | Y | 1 | 0 | 1 | 0 | -0.197 | 0.856 | Y | Y | Y | N | N |
| Mcf2l | 17207 | mcf.2 transforming sequence-like | Y | 1 | 0 | 0 | 1 | -0.01 | 0.496 | Y | N | N | Y | N |
| Nfic | 18029 | nuclear factor I/C | Y | 2 | 0 | 2 | 0 | -0.04 | 0.747 | Y | Y | Y | N | N |
| Gnl1 | 14670 | guanine nucleotide binding protein-like 1 | Y | 1 | 1 | 0 | 0 | -0.23 | 0 | N | N | N | N | Y |
| Hbp1 | 73389 | high mobility group box transcription factor 1 | Y | 1 | 1 | 0 | 0 | -0.242 | 0.886 | Y | Y | N | N | Y |
| Heg1 | 77446 | HEG homolog 1 (zebrafish) | Y | 1 | 1 | 0 | 0 | -0.258 | 0.945 | Y | N | N | Y | Y |
| Jak1 | 16451 | Janus kinase 1 | Y | 1 | 0 | 1 | 0 | -0.192 | 0.892 | Y | Y | Y | Y | N |
| Brms1l | 52592 | breast cancer metastasis-suppressor 1-like | Y | 2 | 1 | 1 | 0 | -0.334 | 0.754 | Y | Y | Y | Y | Y |
| Lrig1 | 16206 | leucine-rich repeats and immunoglobulin-like domains 1 | Y | 1 | 0 | 1 | 0 | -0.068 | 0.745 | Y | Y | Y | N | N |
| Btbd10 | 68815 | BTB (POZ) domain containing 10 | Y | 1 | 1 | 0 | 0 | -0.255 | 0.7 | Y | N | N | N | Y |
| Frmd4a | 209630 | FERM domain containing 4A | Y | 1 | 0 | 1 | 0 | -0.098 | 0.882 | Y | Y | Y | N | N |
| Irf1 | 16362 | interferon regulatory factor 1 | Y | 1 | 0 | 1 | 0 | -0.079 | 0.651 | Y | Y | Y | N | N |
| Ptpn3 | 545622 | protein tyrosine phosphatase, non-receptor type 3 | Y | 1 | 1 | 0 | 0 | -0.251 | 0.966 | N | Y | N | N | Y |
| Ero1lb | 67475 | ERO1-like beta (S. cerevisiae) | Y | 1 | 0 | 1 | 0 | -0.07 | 0.062 | Y | Y | Y | N | N |
| Zdhhc1 | 70796 | zinc finger, DHHC domain containing 1 | Y | 1 | 1 | 0 | 0 | -0.242 | 0.896 | N | N | N | N | Y |
| Golga1 | 76899 | golgi autoantigen, golgin subfamily a, 1 | Y | 1 | 1 | 0 | 0 | -0.27 | 0.695 | Y | Y | N | N | Y |
| Gbf1 | 107338 | golgi-specific brefeldin A-resistance factor 1 | Y | 1 | 0 | 1 | 0 | -0.123 | 0.846 | Y | Y | Y | N | N |
| Ube2q2 | 109161 | ubiquitin-conjugating enzyme E2Q (putative) 2 | Y | 1 | 0 | 1 | 0 | -0.188 | 0.197 | Y | Y | Y | Y | N |
| Pfkfb3 | 170768 | 6-phosphofructo-2-kinase/fructose-2,6-biphosphatase 3 | Y | 1 | 0 | 1 | 0 | -0.09 | 0.484 | Y | Y | Y | Y | N |
| Ccnd1 | 12443 | cyclin D1 | Y | 1 | 0 | 1 | 0 | -0.033 | 0.89 | Y | Y | Y | N | N |
| Zbtb9 | 474156 | zinc finger and BTB domain containing 9 | Y | 1 | 1 | 0 | 0 | -0.232 | 0.932 | N | N | N | N | Y |
| Gigyf1 | 57330 | GRB10 interacting GYF protein 1 | Y | 1 | 1 | 0 | 0 | -0.192 | 0.95 | Y | Y | N | N | Y |
| Sash1 | 70097 | SAM and SH3 domain containing 1 | Y | 1 | 0 | 1 | 0 | -0.178 | 0.905 | Y | Y | Y | N | N |
| Armc8 | 74125 | armadillo repeat containing 8 | Y | 1 | 1 | 0 | 0 | -0.362 | 0.922 | Y | N | N | N | Y |
| Sfmbt1 | 54650 | Scm-like with four mbt domains 1 | Y | 2 | 0 | 2 | 0 | -0.323 | 0.991 | Y | Y | Y | Y | N |
| 6430548M08Rik | 234797 | RIKEN cDNA 6430548M08 gene | Y | 1 | 1 | 0 | 0 | -0.146 | 0.047 | Y | N | N | N | Y |
| Wasf1 | 83767 | WAS protein family, member 1 | Y | 1 | 0 | 1 | 0 | -0.169 | 0.704 | Y | Y | Y | N | N |
| Epha7 | 13841 | Eph receptor A7 | Y | 2 | 1 | 1 | 0 | -0.183 | 0.816 | Y | Y | Y | Y | Y |
| Stk38 | 106504 | serine/threonine kinase 38 | Y | 1 | 1 | 0 | 0 | -0.176 | 0.96 | N | N | N | N | Y |
| Lhx6 | 16874 | LIM homeobox protein 6 | Y | 1 | 0 | 1 | 0 | -0.253 | 0.904 | Y | Y | Y | N | N |
| Trp53inp1 | 60599 | transformation related protein 53 inducible nuclear protein 1 | Y | 1 | 0 | 1 | 0 | -0.128 | 0.893 | Y | Y | Y | N | N |
| Cep57 | 74360 | centrosomal protein 57 | Y | 1 | 0 | 1 | 0 | -0.172 | 0.758 | Y | Y | Y | N | N |
| March8 | 71779 | membrane-associated ring finger (C3HC4) 8 | Y | 1 | 1 | 0 | 0 | -0.202 | 0.955 | Y | Y | N | N | Y |
| Ppp2r3a | 235542 | protein phosphatase 2, regulatory subunit B'', alpha | Y | 1 | 0 | 0 | 1 | -0.01 | 0.516 | Y | Y | N | Y | N |
| Slc4a7 | 218756 | solute carrier family 4, sodium bicarbonate cotransporter, member 7 | Y | 1 | 0 | 1 | 0 | -0.02 | 0.763 | Y | Y | Y | N | N |
| Foxj2 | 60611 | forkhead box J2 | Y | 1 | 1 | 0 | 0 | -0.17 | 0.872 | N | N | N | N | Y |
| Rell1 | 100532 | RELT-like 1 | Y | 1 | 0 | 1 | 0 | -0.142 | 0.889 | Y | Y | Y | N | N |
| Arid4a | 238247 | AT rich interactive domain 4A (RBP1-like) | Y | 1 | 0 | 1 | 0 | -0.108 | 0.412 | Y | Y | Y | N | N |
| Xiap | 11798 | X-linked inhibitor of apoptosis | Y | 1 | 0 | 0 | 1 | -0.02 | 0.48 | Y | Y | N | Y | N |
| Pthlh | 19227 | parathyroid hormone-like peptide | Y | 1 | 1 | 0 | 0 | -0.299 | 0.858 | N | N | N | N | Y |
| Mcl1 | 17210 | myeloid cell leukemia sequence 1 | Y | 1 | 1 | 0 | 0 | -0.244 | 0.938 | Y | N | N | Y | Y |
| Mkrn1 | 54484 | makorin, ring finger protein, 1 | Y | 2 | 0 | 2 | 0 | -0.273 | 0.918 | Y | Y | Y | N | N |
| Klhl20 | 226541 | kelch-like 20 (Drosophila) | Y | 1 | 0 | 1 | 0 | -0.157 | 0.842 | Y | Y | Y | Y | N |
| Zfp597 | 71063 | zinc finger protein 597 | Y | 1 | 1 | 0 | 0 | -0.422 | 0.489 | Y | Y | Y | Y | Y |
| Tbc1d12 | 209478 | TBC1D12: TBC1 domain family, member 12 | Y | 1 | 1 | 0 | 0 | -0.134 | 0.779 | N | Y | N | N | Y |
| Zfp9 | 22750 | zinc finger protein 9 | Y | 1 | 1 | 0 | 0 | -0.33 | 0 | Y | Y | Y | N | Y |
| Map3k8 | 26410 | mitogen-activated protein kinase kinase kinase 8 | Y | 1 | 0 | 1 | 0 | -0.19 | 0.503 | Y | Y | Y | N | N |
| Trim37 | 68729 | tripartite motif-containing 37 | Y | 1 | 0 | 1 | 0 | -0.039 | 0.906 | Y | Y | Y | N | N |
| Ulk1 | 22241 | unc-51 like kinase 1 | Y | 1 | 1 | 0 | 0 | -0.168 | 0.962 | Y | N | N | Y | Y |
| Map3k14 | 53859 | mitogen-activated protein kinase kinase kinase 14 | Y | 2 | 0 | 2 | 0 | -0.074 | 0.82 | Y | Y | Y | N | N |
| Srgap1 | 117600 | SLIT-ROBO Rho GTPase activating protein 1 | Y | 1 | 0 | 1 | 0 | -0.196 | 0.907 | Y | Y | Y | N | N |
| Tmem123 | 71929 | transmembrane protein 123 | Y | 1 | 0 | 1 | 0 | -0.174 | 0.38 | Y | Y | Y | Y | N |
| Ncoa3 | 17979 | nuclear receptor coactivator 3 | Y | 2 | 1 | 1 | 0 | -0.097 | 0.997 | Y | Y | Y | N | Y |
| Zc3h12c | 244871 | zinc finger CCCH type containing 12C | Y | 2 | 0 | 2 | 0 | -0.264 | 0.888 | Y | Y | Y | N | N |
| Mylip | 218203 | myosin regulatory light chain interacting protein | Y | 1 | 0 | 0 | 1 | -0.034 | 0.515 | Y | N | N | Y | N |
| Atl3 | 109168 | atlastin GTPase 3 | Y | 1 | 1 | 0 | 0 | -0.264 | 0.966 | Y | Y | N | Y | Y |
| Cyb561d1 | 72023 | cytochrome b-561 domain containing 1 | Y | 1 | 0 | 0 | 1 | -0.01 | 0.13 | Y | Y | N | Y | N |
| Txlna | 109658 | taxilin alpha | Y | 1 | 0 | 1 | 0 | -0.071 | 0.758 | Y | Y | Y | N | N |
| Isoc1 | 66307 | isochorismatase domain containing 1 | Y | 1 | 1 | 0 | 0 | -0.264 | 0.178 | N | N | N | N | Y |
| Crim1 | 50766 | cysteine rich transmembrane BMP regulator 1 (chordin like) | Y | 1 | 0 | 1 | 0 | -0.126 | 0.797 | Y | Y | Y | N | N |
| Unkl | 74154 | unkempt-like (Drosophila) | Y | 1 | 0 | 1 | 0 | -0.105 | 0.904 | Y | Y | Y | N | N |
| Phtf2 | 68770 | putative homeodomain transcription factor 2 | Y | 1 | 0 | 1 | 0 | -0.113 | 0.477 | Y | Y | Y | N | N |
| Ptpn21 | 24000 | protein tyrosine phosphatase, non-receptor type 21 | Y | 1 | 0 | 1 | 0 | -0.121 | 0.887 | Y | Y | Y | N | N |
| Mcm3 | 17215 | minichromosome maintenance deficient 3 (S. cerevisiae) | Y | 1 | 1 | 0 | 0 | -0.172 | 0.122 | N | N | N | N | Y |
| Ccng1 | 12450 | cyclin G1 | Y | 1 | 0 | 1 | 0 | -0.108 | 0.86 | Y | Y | Y | N | N |
| Ptgfrn | 19221 | prostaglandin F2 receptor negative regulator | Y | 1 | 0 | 1 | 0 | -0.081 | 0.884 | Y | Y | Y | N | N |
| E2f3 | 13557 | E2F transcription factor 3 | Y | 1 | 1 | 0 | 0 | -0.03 | 0.772 | N | N | N | N | Y |
| Dedd | 21945 | death effector domain-containing | Y | 1 | 0 | 1 | 0 | -0.087 | 0.237 | Y | Y | Y | N | N |
| Reep3 | 28193 | receptor accessory protein 3 | Y | 1 | 0 | 1 | 0 | -0.16 | 0.906 | Y | Y | Y | Y | N |
| Cep170 | 545389 | centrosomal protein 170 | Y | 1 | 0 | 1 | 0 | -0.095 | 0.116 | Y | Y | Y | N | N |
| Wdr37 | 207615 | WD repeat domain 37 | Y | 1 | 1 | 0 | 0 | -0.224 | 0.966 | Y | Y | Y | Y | Y |
| Morf4l1 | 21761 | mortality factor 4 like 1 | Y | 1 | 0 | 1 | 0 | -0.185 | 0.455 | Y | Y | Y | N | N |
| Plxna1 | 18844 | plexin A1 | Y | 1 | 1 | 0 | 0 | -0.037 | 0.922 | Y | N | N | N | Y |
| C2cd2 | 207781 | C2 calcium-dependent domain containing 2 | Y | 1 | 1 | 0 | 0 | -0.189 | 0.919 | N | Y | N | N | Y |
| Tmcc3 | 319880 | transmembrane and coiled coil domains 3 | Y | 1 | 1 | 0 | 0 | -0.253 | 0.346 | Y | Y | N | Y | Y |
| 4933426M11Rik | 217684 | RIKEN cDNA 4933426M11 gene | Y | 1 | 1 | 0 | 0 | -0.03 | 0.89 | Y | Y | N | N | Y |
| Mmp24 | 17391 | matrix metallopeptidase 24 | Y | 1 | 1 | 0 | 0 | -0.194 | 0.924 | N | N | N | N | Y |
| Znrf1 | 170737 | zinc and ring finger 1 | Y | 1 | 0 | 1 | 0 | -0.126 | 0.81 | Y | Y | Y | N | Y |
| Rab11fip1 | 75767 | RAB11 family interacting protein 1 (class I) | Y | 1 | 1 | 0 | 0 | -0.03 | 0.966 | Y | Y | N | N | Y |
| Hmga2 | 15364 | high mobility group AT-hook 2 | Y | 1 | 0 | 1 | 0 | -0.02 | 0.609 | Y | Y | Y | N | N |
| Hs3st3a1 | 15478 | heparan sulfate (glucosamine) 3-O-sulfotransferase 3A1 | Y | 1 | 0 | 0 | 1 | -0.01 | 0 | Y | Y | N | Y | N |
| Dnajc27 | 217378 | DnaJ (Hsp40) homolog, subfamily C, member 27 | Y | 1 | 1 | 0 | 0 | -0.03 | 0.749 | Y | Y | N | N | Y |
| Wipf2 | 68524 | WAS/WASL interacting protein family, member 2 | Y | 1 | 0 | 1 | 0 | -0.04 | 0.56 | Y | Y | Y | N | N |
| Plagl2 | 54711 | pleiomorphic adenoma gene-like 2 | Y | 1 | 0 | 1 | 0 | -0.174 | 0.831 | Y | Y | Y | N | N |
| Sipa1l3 | 74206 | signal-induced proliferation-associated 1 like 3 | Y | 1 | 0 | 1 | 0 | -0.088 | 0.656 | Y | Y | Y | N | N |
| Tiam1 | 21844 | T cell lymphoma invasion and metastasis 1 | Y | 1 | 0 | 1 | 0 | -0.112 | 0.302 | Y | Y | Y | N | N |
| Tnks2 | 74493 | tankyrase, TRF1-interacting ankyrin-related ADP-ribose polymerase 2 | Y | 1 | 1 | 0 | 0 | -0.267 | 0.967 | Y | N | N | N | Y |
| Plekho2 | 102595 | pleckstrin homology domain containing, family O member 2 | Y | 1 | 0 | 1 | 0 | -0.044 | 0.559 | Y | Y | Y | N | N |
| Akt3 | 23797 | thymoma viral proto-oncogene 3 | Y | 1 | 0 | 1 | 0 | -0.048 | 0.436 | Y | Y | Y | N | N |
| Bnip2 | 12175 | BCL2/adenovirus E1B interacting protein 2 | Y | 1 | 1 | 0 | 0 | -0.153 | 0.877 | Y | N | N | Y | Y |
| Cenpo | 52504 | centromere protein O | Y | 1 | 0 | 1 | 0 | -0.02 | 0.851 | Y | Y | Y | N | N |
| Ankrd52 | 237615 | ankyrin repeat domain 52 | Y | 3 | 2 | 1 | 0 | -0.425 | 0.999 | Y | Y | Y | N | Y |
| 2210018M11Rik | 233545 | RIKEN cDNA 2210018M11 gene | Y | 1 | 1 | 0 | 0 | -0.224 | 0.931 | N | N | N | N | Y |
| Rragd | 52187 | Ras-related GTP binding D | Y | 1 | 0 | 1 | 0 | -0.183 | 0.901 | Y | Y | Y | N | N |
| 1600012H06Rik | 67912 | RIKEN cDNA 1600012H06 gene | Y | 1 | 1 | 0 | 0 | -0.282 | 0.667 | Y | Y | N | Y | Y |
| Styx | 56291 | serine/threonine/tyrosine interaction protein | Y | 1 | 0 | 1 | 0 | -0.256 | 0.901 |  |  |  |  |  |
| Tanc1 | 66860 | tetratricopeptide repeat, ankyrin repeat and coiled-coil containing 1 | Y | 1 | 1 | 0 | 0 | -0.174 | 0.966 | Y | Y | Y | N | Y |
| Hlf | 217082 | hepatic leukemia factor | Y | 1 | 1 | 0 | 0 | -0.242 | 0.967 | Y | Y | N | N | Y |
| E2f2 | 242705 | E2F transcription factor 2 | Y | 1 | 0 | 1 | 0 | -0.02 | 0.718 | Y | Y | Y | N | N |
| Tmed8 | 382620 | transmembrane emp24 domain containing 8 | Y | 1 | 1 | 0 | 0 | -0.05 | 0.592 | Y | Y | Y | N | Y |
| Frs2 | 327826 | fibroblast growth factor receptor substrate 2 | Y | 2 | 2 | 0 | 0 | -0.07 | 0.987 | Y | N | N | Y | Y |
| App | 11820 | amyloid beta (A4) precursor protein | Y | 1 | 0 | 1 | 0 | -0.151 | 0.682 | Y | Y | Y | N | N |
| St3gal1 | 20442 | ST3 beta-galactoside alpha-2,3-sialyltransferase 1 | Y | 1 | 0 | 1 | 0 | -0.12 | 0.88 | Y | Y | Y | Y | N |
| Znf512b | 269401 | zinc finger protein 512B | Y | 2 | 2 | 0 | 0 | -0.302 | 0.922 | Y | Y | Y | N | Y |
| Gab1 | 14388 | growth factor receptor bound protein 2-associated protein 1 | Y | 1 | 0 | 1 | 0 | -0.197 | 0.74 | Y | Y | Y | N | N |
| Tmem168 | 101118 | transmembrane protein 168 | Y | 1 | 0 | 1 | 0 | -0.165 | 0.459 | Y | Y | Y | N | N |
| Ppp6c | 67857 | protein phosphatase 6, catalytic subunit | Y | 1 | 0 | 1 | 0 | -0.143 | 0.767 | Y | Y | Y | N | Y |
| Fam126b | 213056 | family with sequence similarity 126, member B | Y | 1 | 0 | 1 | 0 | -0.114 | 0.902 | Y | Y | Y | Y | N |
| Zbtb44 | 235132 | zinc finger and BTB domain containing 44 | Y | 1 | 0 | 1 | 0 | -0.15 | 0.903 | Y | Y | Y | N | N |
| Cnot7 | 18983 | CCR4-NOT transcription complex, subunit 7 | Y | 1 | 1 | 0 | 0 | -0.225 | 0.705 | Y | N | N | N | Y |
| Sirt7 | 209011 | sirtuin 7 (silent mating type information regulation 2, homolog) 7 (S. cerevisiae) | Y | 1 | 0 | 1 | 0 | -0.134 | 0.788 | Y | Y | Y | Y | N |
| 1700021K19Rik | 100502698 | RIKEN cDNA 1700021K19 gene | Y | 1 | 0 | 1 | 0 | -0.098 | 0.839 | Y | Y | Y | N | N |
| Kdm2a | 225876 | lysine (K)-specific demethylase 2A | Y | 1 | 1 | 0 | 0 | -0.035 | 0.895 | N | Y | N | N | Y |
| Foxq1 | 15220 | forkhead box Q1 | Y | 1 | 0 | 1 | 0 | -0.11 | 0.066 | Y | Y | Y | N | N |
| Fam134c | 67998 | family with sequence similarity 134, member C | Y | 1 | 1 | 0 | 0 | -0.18 | 0.862 | Y | Y | Y | Y | Y |
| Pbx3 | 18516 | pre B cell leukemia homeobox 3 | Y | 1 | 1 | 0 | 0 | -0.306 | 0.735 | N | N | N | N | Y |
| Tbcel | 272589 | tubulin folding cofactor E-like | Y | 1 | 0 | 1 | 0 | -0.27 | 0.877 | Y | Y | Y | N | N |
| Bbx | 70508 | bobby sox homolog (Drosophila) | Y | 1 | 0 | 1 | 0 | -0.141 | 0.586 | Y | Y | Y | N | Y |
| Dcun1d3 | 233805 | DCN1, defective in cullin neddylation 1, domain containing 3 (S. cerevisiae) | Y | 1 | 0 | 1 | 0 | -0.159 | 0.903 | Y | Y | Y | N | N |
| Jrkl | 77532 | jerky homolog-like (mouse) | Y | 1 | 0 | 1 | 0 | -0.124 | 0.356 | Y | Y | Y | N | N |
| Usp3 | 235441 | ubiquitin specific peptidase 3 | Y | 1 | 0 | 1 | 0 | -0.196 | 0.797 | Y | Y | Y | N | N |
| Fosl2 | 14284 | fos-like antigen 2 | Y | 1 | 0 | 1 | 0 | -0.02 | 0.247 | Y | Y | Y | N | N |
| Mtmr3 | 74302 | myotubularin related protein 3 | Y | 1 | 0 | 1 | 0 | -0.224 | 0.883 | Y | Y | Y | N | N |
| Sorbs2 | 234214 | sorbin and SH3 domain containing 2 | Y | 1 | 0 | 1 | 0 | -0.096 | 0.381 | Y | Y | Y | Y | N |
| Socs7 | 192157 | suppressor of cytokine signaling 7 | Y | 1 | 0 | 1 | 0 | -0.135 | 0.85 | Y | Y | Y | N | N |
| Tbx3 | 21386 | T-box 3 | Y | 1 | 0 | 1 | 0 | -0.166 | 0.817 | Y | Y | Y | N | N |
| Ddhd2 | 72108 | DDHD domain containing 2 | Y | 1 | 0 | 1 | 0 | -0.221 | 0.896 | Y | Y | Y | N | N |
| Siah2 | 20439 | seven in absentia 2 | Y | 1 | 0 | 0 | 1 | -0.083 | 0 | Y | N | N | Y | N |
| Usp24 | 329908 | ubiquitin specific peptidase 24 | Y | 1 | 1 | 0 | 0 | -0.222 | 0.921 | N | N | N | N | Y |
| Gtdc1 | 227835 | glycosyltransferase-like domain containing 1 | Y | 1 | 0 | 1 | 0 | -0.143 | 0.644 | Y | Y | Y | Y | N |
| Arhgef11 | 213498 | Rho guanine nucleotide exchange factor (GEF) 11 | Y | 1 | 0 | 1 | 0 | -0.155 | 0.804 | Y | Y | Y | N | N |
| Pgm2l1 | 70974 | phosphoglucomutase 2-like 1 | Y | 1 | 1 | 0 | 0 | -0.339 | 0.598 | N | N | N | N | Y |
| Get4 | 67604 | golgi to ER traffic protein 4 homolog (S. cerevisiae) | Y | 1 | 0 | 0 | 1 | -0.052 | 0.501 | Y | N | N | Y | N |
| Atg2a | 329015 | autophagy related 2A | Y | 1 | 0 | 1 | 0 | -0.107 | 0.182 | Y | Y | Y | N | N |
| Fbxo21 | 231670 | F-box protein 21 | Y | 1 | 1 | 0 | 0 | -0.255 | 0.828 | Y | N | N | Y | Y |
| Hmbox1 | 219150 | homeobox containing 1 | Y | 1 | 0 | 1 | 0 | -0.239 | 0.317 | Y | N | N | N | N |
| Csnk1g1 | 214897 | casein kinase 1, gamma 1 | Y | 1 | 0 | 1 | 0 | -0.154 | 0.889 | Y | Y | Y | N | N |
| Dennd5b | 320560 | DENN/MADD domain containing 5B | Y | 1 | 1 | 0 | 0 | -0.314 | 0.967 | N | N | N | N | Y |
| Tmub2 | 72053 | transmembrane and ubiquitin-like domain containing 2 | Y | 1 | 0 | 1 | 0 | -0.139 | 0.4 | Y | Y | Y | N | N |
| Pip4k2a | 18718 | phosphatidylinositol-5-phosphate 4-kinase, type II, alpha | Y | 1 | 0 | 1 | 0 | -0.23 | 0.082 | Y | Y | Y | N | N |
| Zfpm2 | 22762 | zinc finger protein, multitype 2 | Y | 1 | 1 | 0 | 0 | -0.248 | 0.733 | N | Y | N | N | Y |
| Eif5a2 | 208691 | eukaryotic translation initiation factor 5A2 | Y | 1 | 0 | 1 | 0 | -0.138 | 0.732 | Y | Y | Y | N | N |
| Tnrc6a | 233833 | trinucleotide repeat containing 6a | Y | 1 | 0 | 1 | 0 | -0.02 | 0.764 | Y | Y | Y | N | N |
| Dgkq | 110524 | diacylglycerol kinase, theta | Y | 1 | 1 | 0 | 0 | -0.175 | 0 | N | Y | N | N | Y |
| Zfp827 | 622675 | zinc finger protein 827 | Y | 3 | 1 | 2 | 0 | -0.285 | 0.928 | Y | Y | Y | N | Y |
| Ate1 | 11907 | arginyltransferase 1 | Y | 1 | 0 | 1 | 0 | -0.176 | 0.759 | Y | Y | Y | N | N |
| Stx6 | 58244 | syntaxin 6 | Y | 1 | 0 | 1 | 0 | -0.263 | 0.739 | Y | Y | Y | N | N |
| Dcbld2 | 73379 | discoidin, CUB and LCCL domain containing 2 | Y | 1 | 0 | 1 | 0 | -0.043 | 0.903 | Y | Y | Y | N | N |
| Zbtb41 | 226470 | zinc finger and BTB domain containing 41 homolog | Y | 2 | 0 | 2 | 0 | -0.05 | 0.873 | Y | Y | Y | Y | N |
| Rock2 | 19878 | Rho-associated coiled-coil containing protein kinase 2 | Y | 1 | 0 | 1 | 0 | -0.081 | 0.818 | Y | Y | Y | N | N |
| Lin52 | 217708 | lin-52 homolog (C. elegans) | Y | 1 | 0 | 1 | 0 | -0.084 | 0.283 | Y | Y | Y | N | N |
| Iqsec1 | 232227 | IQ motif and Sec7 domain 1 | Y | 1 | 0 | 1 | 0 | -0.161 | 0.892 | Y | Y | Y | Y | N |
| Kpna2 | 16647 | karyopherin (importin) alpha 2 | Y | 1 | 0 | 1 | 0 | -0.238 | 0.281 | Y | Y | Y | N | N |
| Acbd5 | 74159 | acyl-Coenzyme A binding domain containing 5 | Y | 1 | 0 | 1 | 0 | -0.168 | 0.742 | Y | Y | Y | N | N |
| Zfp800 | 627049 | zinc finger protein 800 | Y | 1 | 1 | 0 | 0 | -0.33 | 0.732 | Y | Y | Y | N | Y |
| Ankfy1 | 11736 | ankyrin repeat and FYVE domain containing 1 | Y | 2 | 0 | 2 | 0 | -0.11 | 0.978 | Y | Y | Y | N | N |
| Cmpk1 | 66588 | cytidine monophosphate (UMP-CMP) kinase 1 | Y | 1 | 0 | 1 | 0 | -0.193 | 0.754 | Y | Y | Y | N | N |
| Fam102a | 98952 | family with sequence similarity 102, member A | Y | 2 | 1 | 1 | 0 | -0.077 | 0.841 | Y | Y | Y | N | Y |
| Furin | 18550 | furin (paired basic amino acid cleaving enzyme) | Y | 1 | 0 | 1 | 0 | -0.134 | 0.727 | Y | Y | Y | Y | N |
| Adam9 | 11502 | a disintegrin and metallopeptidase domain 9 (meltrin gamma) | Y | 1 | 1 | 0 | 0 | -0.226 | 0.905 | N | Y | N | N | Y |
| Aff4 | 93736 | AF4/FMR2 family, member 4 | Y | 1 | 0 | 0 | 1 | -0.04 | 0.233 | Y | Y | Y | Y | N |
| Ocrl | 320634 | oculocerebrorenal syndrome of Lowe | Y | 1 | 0 | 1 | 0 | -0.095 | 0.561 | Y | Y | Y | N | N |
| Ip6k1 | 27399 | inositol hexaphosphate kinase 1 | Y | 1 | 0 | 1 | 0 | -0.073 | 0.412 | Y | Y | Y | N | N |
| Neurl1b | 240055 | neuralized homolog 1b (Drosophila) | Y | 1 | 0 | 1 | 0 | -0.14 | 0.858 | Y | Y | Y | Y | N |
| Kirrel | 170643 | kin of IRRE like (Drosophila) | Y | 1 | 0 | 1 | 0 | -0.07 | 0.874 | Y | Y | Y | N | N |
| Zfp217 | 228913 | zinc finger protein 217 | Y | 1 | 0 | 1 | 0 | -0.125 | 0.876 | Y | Y | Y | N | N |
| Ano6 | 105722 | anoctamin 6 | Y | 1 | 1 | 0 | 0 | -0.31 | 0.946 | N | N | N | N | Y |
| Eea1 | 216238 | early endosome antigen 1 | Y | 1 | 0 | 1 | 0 | -0.114 | 0.701 | Y | Y | Y | Y | N |
| Ube3c | 100763 | ubiquitin protein ligase E3C | Y | 1 | 1 | 0 | 0 | -0.13 | 0.794 | Y | N | N | N | Y |
| Ski | 20481 | ski sarcoma viral oncogene homolog (avian) | Y | 1 | 0 | 1 | 0 | -0.038 | 0.877 | Y | Y | Y | N | N |
| Ccnd2 | 12444 | cyclin D2 | Y | 1 | 0 | 1 | 0 | -0.069 | 0.9 | Y | Y | Y | N | N |
| Tor1aip2 | 240832 | torsin A interacting protein 2 | Y | 1 | 0 | 1 | 0 | -0.126 | 0.256 | Y | Y | Y | Y | N |
| 1810013L24Rik | 69053 | RIKEN cDNA 1810013L24 gene | Y | 1 | 0 | 1 | 0 | -0.02 | 0.479 | Y | Y | Y | N | N |
| Mgea5 | 76055 | meningioma expressed antigen 5 (hyaluronidase) | Y | 1 | 0 | 1 | 0 | -0.056 | 0.227 | Y | Y | Y | N | N |
| Rasa1 | 218397 | RAS p21 protein activator 1 | Y | 1 | 0 | 1 | 0 | -0.108 | 0.872 | Y | Y | Y | N | N |
| Foxj3 | 230700 | forkhead box J3 | Y | 1 | 1 | 0 | 0 | -0.098 | 0.868 | N | Y | N | N | Y |
| Rnf4 | 19822 | ring finger protein 4 | Y | 1 | 0 | 0 | 1 | -0.01 | 0.248 | Y | N | N | Y | N |
| Mllt6 | 246198 | myeloid/lymphoid or mixed-lineage leukemia (trithorax homolog, Drosophila); translocated to, 6 | Y | 1 | 0 | 0 | 1 | -0.01 | 0 | Y | Y | N | Y | N |
| Ssx2ip | 99167 | synovial sarcoma, X breakpoint 2 interacting protein | Y | 1 | 0 | 1 | 0 | -0.12 | 0.857 | Y | Y | Y | N | N |
| Egfr | 13649 | epidermal growth factor receptor | Y | 1 | 0 | 1 | 0 | -0.107 | 0.097 | Y | Y | Y | N | N |
| Elk4 | 13714 | ELK4, member of ETS oncogene family | Y | 1 | 0 | 1 | 0 | -0.127 | 0.699 | Y | Y | Y | N | N |
| Fnbp1l | 214459 | formin binding protein 1-like | Y | 1 | 0 | 1 | 0 | -0.111 | 0.497 | Y | Y | Y | Y | N |
| Pdgfra | 18595 | platelet derived growth factor receptor, alpha polypeptide | Y | 1 | 0 | 1 | 0 | -0.213 | 0.899 | Y | Y | Y | N | N |
| Fmnl3 | 22379 | formin-like 3 | Y | 1 | 0 | 1 | 0 | -0.161 | 0.864 | Y | Y | Y | N | N |
| Kif3b | 16569 | kinesin family member 3B | Y | 1 | 0 | 1 | 0 | -0.094 | 0.598 | Y | Y | Y | Y | N |
| Fbxo10 | 269529 | F-box protein 10 | Y | 1 | 0 | 1 | 0 | -0.067 | 0.213 | Y | Y | Y | N | N |
| Zfp148 | 22661 | zinc finger protein 148 | Y | 1 | 1 | 0 | 0 | -0.3 | 0.926 | Y | Y | N | N | Y |
| Xrn1 | 24127 | 5'-3' exoribonuclease 1 | Y | 1 | 0 | 1 | 0 | -0.511 | 0.106 | Y | Y | Y | N | Y |
| Habp4 | 56541 | hyaluronic acid binding protein 4 | Y | 1 | 0 | 1 | 0 | -0.166 | 0.816 | Y | Y | Y | N | N |
| Lysmd3 | 80289 | LysM, putative peptidoglycan-binding, domain containing 3 | Y | 1 | 0 | 1 | 0 | -0.061 | 0.15 | Y | Y | Y | N | N |
| Irf2bp2 | 270110 | interferon regulatory factor 2 binding protein 2 | Y | 1 | 1 | 0 | 0 | -0.174 | 0.862 | N | Y | N | N | Y |
| Btg1 | 12226 | B cell translocation gene 1, anti-proliferative | Y | 1 | 0 | 0 | 1 | -0.026 | 0 | Y | Y | N | Y | N |
| Mapre1 | 13589 | microtubule-associated protein, RP/EB family, member 1 | Y | 1 | 0 | 1 | 0 | -0.126 | 0.905 | Y | Y | Y | N | N |
| Kif26b | 269152 | kinesin family member 26B | Y | 1 | 0 | 1 | 0 | -0.141 | 0.797 | Y | Y | Y | N | N |
| Crk | 12928 | v-crk sarcoma virus CT10 oncogene homolog (avian) | Y | 1 | 1 | 0 | 0 | -0.14 | 0.915 | Y | Y | N | N | Y |
| Fbxo28 | 67948 | F-box protein 28 | Y | 2 | 1 | 0 | 1 | -0.068 | 0.955 | Y | Y | N | Y | Y |
| Cyld | 74256 | cylindromatosis (turban tumor syndrome) | Y | 1 | 1 | 0 | 0 | -0.107 | 0.365 | Y | Y | Y | Y | Y |
| Mbd5 | 109241 | methyl-CpG binding domain protein 5 | Y | 1 | 0 | 0 | 1 | -0.056 | 0 | Y | Y | N | Y | N |
| Snrk | 20623 | SNF related kinase | Y | 1 | 0 | 0 | 1 | -0.01 | 0.199 | Y | Y | N | Y | N |
| Crybg3 | 224273 | beta-gamma crystallin domain containing 3 | Y | 1 | 1 | 0 | 0 | -0.284 | 0.966 | Y | Y | Y | N | Y |
| Ntn1 | 18208 | netrin 1 | Y | 1 | 1 | 0 | 0 | -0.03 | 0.892 | N | N | N | N | Y |
| BC030336 | 233812 | cDNA sequence BC030336 | Y | 1 | 0 | 1 | 0 | -0.242 | 0.764 | Y | Y | Y | Y | N |
| Zfyve20 | 78287 | zinc finger, FYVE domain containing 20 | Y | 1 | 0 | 1 | 0 | -0.138 | 0.893 | Y | Y | Y | N | N |
| Eri1 | 67276 | exoribonuclease 1 | Y | 1 | 0 | 1 | 0 | -0.14 | 0.906 | Y | Y | Y | N | N |
| Has2 | 15117 | hyaluronan synthase 2 | Y | 1 | 0 | 1 | 0 | -0.118 | 0.788 | Y | Y | Y | N | N |
| Pkn2 | 109333 | protein kinase N2 | Y | 1 | 0 | 1 | 0 | -0.119 | 0.897 | Y | Y | Y | Y | N |
| Tbc1d2b | 67016 | TBC1 domain family, member 2B | Y | 1 | 0 | 1 | 0 | -0.102 | 0.866 | Y | Y | Y | N | N |
| Zdhhc9 | 208884 | zinc finger, DHHC domain containing 9 | Y | 1 | 0 | 1 | 0 | -0.025 | 0.354 | Y | Y | Y | N | N |
| Skil | 20482 | SKI-like | Y | 1 | 0 | 1 | 0 | -0.047 | 0.804 | Y | Y | Y | N | N |
| Wdfy3 | 72145 | WD repeat and FYVE domain containing 3 | Y | 1 | 0 | 1 | 0 | -0.167 | 0.631 | Y | Y | Y | N | N |
| Cfl2 | 12632 | cofilin 2, muscle | Y | 1 | 0 | 1 | 0 | -0.231 | 0.209 | Y | Y | Y | N | N |
| Rest | 19712 | RE1-silencing transcription factor | Y | 1 | 1 | 0 | 0 | -0.278 | 0.662 | N | N | N | N | Y |
| Sall1 | 58198 | sal-like 1 (Drosophila) | Y | 1 | 0 | 1 | 0 | -0.206 | 0.578 | Y | Y | Y | N | N |
| Fam117a | 215512 | family with sequence similarity 117, member A | Y | 1 | 0 | 1 | 0 | -0.127 | 0.183 | Y | Y | Y | Y | N |
| Pank3 | 211347 | pantothenate kinase 3 | Y | 1 | 1 | 0 | 0 | -0.04 | 0.677 | Y | Y | N | Y | Y |
| Cxadr | 13052 | coxsackie virus and adenovirus receptor | Y | 1 | 0 | 1 | 0 | -0.179 | 0.851 | Y | Y | Y | N | N |
| Dpysl2 | 12934 | dihydropyrimidinase-like 2 | Y | 1 | 0 | 1 | 0 | -0.169 | 0.904 | Y | Y | Y | N | N |
| Mpdz | 17475 | multiple PDZ domain protein | Y | 1 | 0 | 0 | 1 | -0.075 | 0 | Y | N | N | Y | N |
| Smad6 | 17130 | SMAD family member 6 | Y | 1 | 0 | 1 | 0 | -0.09 | 0.746 | Y | Y | Y | N | N |
| Mknk2 | 17347 | MAP kinase-interacting serine/threonine kinase 2 | Y | 1 | 0 | 1 | 0 | -0.15 | 0.865 | Y | Y | Y | N | N |
| Ptbp1 | 19205 | polypyrimidine tract binding protein 1 | Y | 1 | 0 | 0 | 1 | -0.05 | 0.516 | Y | Y | N | Y | N |
| Itfg1 | 71927 | integrin alpha FG-GAP repeat containing 1 | Y | 1 | 0 | 1 | 0 | -0.237 | 0.839 | Y | Y | Y | N | N |
| Fnbp4 | 55935 | formin binding protein 4 | Y | 1 | 0 | 0 | 1 | -0.01 | 0.305 | Y | N | N | Y | N |
| Zfp236 | 329002 | zinc finger protein 236 | Y | 1 | 0 | 1 | 0 | -0.045 | 0.894 | Y | Y | Y | N | N |
| Nploc4 | 217365 | nuclear protein localization 4 homolog (S. cerevisiae) | Y | 1 | 1 | 0 | 0 | -0.136 | 0.304 | N | N | N | N | Y |
| Ubxn2b | 68053 | UBX domain protein 2B | Y | 1 | 1 | 0 | 0 | -0.185 | 0.754 | Y | Y | N | N | Y |
| Ctdspl2 | 329506 | CTD (carboxy-terminal domain, RNA polymerase II, polypeptide A) small phosphatase like 2 | Y | 1 | 0 | 1 | 0 | -0.111 | 0.765 | Y | Y | Y | N | Y |
| Frmd6 | 319710 | FERM domain containing 6 | Y | 1 | 1 | 0 | 0 | -0.285 | 0.962 | Y | Y | N | N | Y |
| Dnm2 | 13430 | dynamin 2 | Y | 1 | 0 | 1 | 0 | -0.069 | 0.654 | Y | Y | Y | N | N |
| Abhd5 | 67469 | abhydrolase domain containing 5 | Y | 1 | 1 | 0 | 0 | -0.2 | 0.706 | N | Y | N | N | Y |
| Plekhg5 | 269608 | pleckstrin homology domain containing, family G (with RhoGef domain) member 5 | Y | 1 | 0 | 1 | 0 | -0.088 | 0.507 | Y | Y | Y | N | N |
| Zbtb6 | 241322 | zinc finger and BTB domain containing 6 | Y | 1 | 0 | 1 | 0 | -0.12 | 0.746 | Y | Y | Y | Y | N |
| Nufip2 | 68564 | nuclear fragile X mental retardation protein interacting protein 2 | Y | 1 | 0 | 1 | 0 | -0.02 | 0.865 | Y | Y | N | N | N |
| Msl1 | 74026 | male-specific lethal 1 homolog (Drosophila) | Y | 1 | 0 | 1 | 0 | -0.032 | 0.641 | Y | Y | Y | N | N |
| Lrch1 | 380916 | leucine-rich repeats and calponin homology (CH) domain containing 1 | Y | 1 | 1 | 0 | 0 | -0.285 | 0.675 | Y | Y | Y | N | Y |
| Atad2 | 70472 | ATPase family, AAA domain containing 2 | Y | 1 | 0 | 1 | 0 | -0.198 | 0.84 | Y | Y | Y | N | N |
| Lsm6 | 78651 | LSM6 homolog, U6 small nuclear RNA associated (S. cerevisiae) | Y | 1 | 0 | 0 | 1 | -0.01 | 0 | Y | N | N | Y | N |
| Kpna3 | 16648 | karyopherin (importin) alpha 3 | Y | 2 | 0 | 1 | 1 | -0.189 | 0.808 | Y | Y | Y | Y | N |
| Rybp | 56353 | RING1 and YY1 binding protein | Y | 1 | 0 | 0 | 1 | -0.081 | 0.388 | Y | Y | Y | Y | N |
| Fndc3b | 72007 | fibronectin type III domain containing 3B | Y | 1 | 1 | 0 | 0 | -0.117 | 0 | N | N | N | N | Y |
| Ostm1 | 14628 | osteopetrosis associated transmembrane protein 1 | Y | 1 | 1 | 0 | 0 | -0.229 | 0.928 | N | N | N | N | Y |
| Ldlr | 16835 | low density lipoprotein receptor | Y | 1 | 0 | 1 | 0 | -0.02 | 0.341 | Y | Y | Y | N | N |
| Zbtb43 | 71834 | zinc finger and BTB domain containing 43 | Y | 1 | 0 | 1 | 0 | -0.043 | 0.906 | Y | Y | Y | N | N |
| Rab10 | 19325 | RAB10, member RAS oncogene family | Y | 1 | 0 | 1 | 0 | -0.161 | 0.123 | Y | Y | Y | N | N |
| Slc30a7 | 66500 | solute carrier family 30 (zinc transporter), member 7 | Y | 1 | 1 | 0 | 0 | -0.05 | 0.364 | Y | Y | Y | N | Y |
| Dgke | 56077 | diacylglycerol kinase, epsilon | Y | 1 | 1 | 0 | 0 | -0.245 | 0.938 | Y | Y | N | Y | Y |
| Slc25a36 | 192287 | solute carrier family 25, member 36 | Y | 1 | 0 | 1 | 0 | -0.061 | 0.855 | Y | Y | Y | Y | N |
| Oxr1 | 170719 | oxidation resistance 1 | Y | 1 | 1 | 0 | 0 | -0.275 | 0.857 | N | N | N | N | Y |
| Fmn1 | 14260 | formin 1 | Y | 1 | 1 | 0 | 0 | -0.17 | 0.514 | Y | Y | N | Y | Y |
| Usp31 | 76179 | ubiquitin specific peptidase 31 | Y | 2 | 0 | 2 | 0 | -0.223 | 0.962 | Y | Y | Y | N | N |
| Metap1 | 75624 | methionyl aminopeptidase 1 | Y | 1 | 0 | 1 | 0 | -0.099 | 0.674 | Y | Y | Y | N | N |
| Prune2 | 353211 | prune homolog 2 (Drosophila) | Y | 1 | 0 | 1 | 0 | -0.101 | 0.85 | Y | Y | Y | N | N |
| Socs6 | 54607 | suppressor of cytokine signaling 6 | Y | 1 | 0 | 1 | 0 | -0.172 | 0.897 | Y | Y | Y | Y | N |
| Grlf1 | 232906 | glucocorticoid receptor DNA binding factor 1 | Y | 1 | 1 | 0 | 0 | -0.04 | 0.572 | Y | N | N | Y | Y |
| Rapgef2 | 76089 | Rap guanine nucleotide exchange factor (GEF) 2 | Y | 1 | 0 | 1 | 0 | -0.053 | 0.781 | Y | Y | Y | N | N |
| Rb1 | 19645 | retinoblastoma 1 | Y | 1 | 0 | 1 | 0 | -0.07 | 0.449 | Y | Y | Y | N | N |
| Rsf1 | 233532 | remodeling and spacing factor 1 | Y | 1 | 0 | 0 | 1 | -0.06 | 0.367 | Y | Y | N | Y | Y |
| Fcho2 | 218503 | FCH domain only 2 | Y | 2 | 0 | 2 | 0 | -0.309 | 0.845 | Y | Y | Y | N | N |
| B630005N14Rik | 101148 | RIKEN cDNA B630005N14 gene | Y | 1 | 0 | 1 | 0 | -0.166 | 0.874 | Y | Y | Y | Y | N |
| Vps26a | 30930 | vacuolar protein sorting 26 homolog A (yeast) | Y | 1 | 0 | 1 | 0 | -0.109 | 0.635 | Y | Y | Y | N | N |
| Naa30 | 70646 | N(alpha)-acetyltransferase 30, NatC catalytic subunit | Y | 1 | 0 | 1 | 0 | -0.213 | 0.765 | Y | Y | Y | N | N |
| Tet3 | 194388 | tet methylcytosine dioxygenase 3 | Y | 2 | 0 | 2 | 0 | -0.109 | 0.977 | Y | Y | Y | N | N |
| Luzp1 | 269593 | leucine zipper protein 1 | Y | 1 | 1 | 0 | 0 | -0.03 | 0.899 | Y | Y | N | N | Y |
| Slain2 | 75991 | SLAIN motif family, member 2 | Y | 1 | 1 | 0 | 0 | -0.254 | 0.963 | Y | Y | N | Y | Y |
| D230025D16Rik | 234678 | RIKEN cDNA D230025D16 gene | Y | 1 | 0 | 0 | 1 | -0.013 | 0.505 | Y | N | N | Y | N |
| Sike1 | 66641 | suppressor of IKBKE 1 | Y | 1 | 0 | 1 | 0 | -0.04 | 0.601 | Y | Y | Y | N | N |
| Papola | 18789 | poly (A) polymerase alpha | Y | 1 | 0 | 1 | 0 | -0.186 | 0.73 | Y | Y | Y | N | N |
| Dip2c | 208440 | DIP2 disco-interacting protein 2 homolog C (Drosophila) | Y | 1 | 0 | 0 | 1 | -0.01 | 0.259 | Y | N | N | Y | N |
| Arid4b | 94246 | AT rich interactive domain 4B (RBP1-like) | Y | 1 | 0 | 1 | 0 | -0.38 | 0.643 | Y | Y | Y | Y | N |
| Klf9 | 16601 | Kruppel-like factor 9 | Y | 1 | 0 | 1 | 0 | -0.124 | 0.751 | Y | Y | Y | N | N |
| Tsg101 | 22088 | tumor susceptibility gene 101 | Y | 1 | 0 | 1 | 0 | -0.163 | 0.9 | Y | Y | Y | N | N |
| Sos1 | 20662 | son of sevenless homolog 1 (Drosophila) | Y | 1 | 0 | 1 | 0 | -0.222 | 0.867 | Y | Y | Y | N | N |
| Lrp8 | 16975 | low density lipoprotein receptor-related protein 8, apolipoprotein e receptor | Y | 1 | 1 | 0 | 0 | -0.086 | 0.762 | Y | Y | N | N | Y |
| Dnajb6 | 23950 | DnaJ (Hsp40) homolog, subfamily B, member 6 | Y | 1 | 0 | 1 | 0 | -0.179 | 0.626 | N | Y | N | N | N |
| Npat | 244879 | nuclear protein in the AT region | Y | 2 | 0 | 1 | 1 | -0.18 | 0.955 | Y | Y | Y | Y | N |
| Ubfd1 | 28018 | ubiquitin family domain containing 1 | Y | 1 | 0 | 1 | 0 | -0.03 | 0.827 | Y | Y | Y | Y | N |
| Hif1a | 15251 | hypoxia inducible factor 1, alpha subunit | Y | 1 | 0 | 1 | 0 | -0.085 | 0.502 | Y | Y | Y | N | N |
| Ankrd17 | 81702 | ankyrin repeat domain 17 | Y | 1 | 1 | 0 | 0 | -0.251 | 0.939 | Y | N | N | N | Y |
| Tob2 | 57259 | transducer of ERBB2, 2 | Y | 1 | 0 | 1 | 0 | -0.148 | 0.147 | Y | Y | Y | N | N |
| Sertad2 | 58172 | SERTA domain containing 2 | Y | 1 | 1 | 0 | 0 | -0.107 | 0.811 | Y | Y | Y | Y | Y |
| Rab12 | 19328 | RAB12, member RAS oncogene family | Y | 1 | 0 | 1 | 0 | -0.123 | 0.475 | Y | Y | Y | N | N |
| Ss18l1 | 269397 | synovial sarcoma translocation gene on chromosome 18-like 1 | Y | 1 | 0 | 1 | 0 | -0.143 | 0.905 | Y | Y | Y | N | N |
| Erc1 | 111173 | ELKS/RAB6-interacting/CAST family member 1 | Y | 1 | 1 | 0 | 0 | -0.197 | 0.961 | Y | N | N | N | Y |
| Pitpna | 18738 | phosphatidylinositol transfer protein, alpha | Y | 1 | 0 | 1 | 0 | -0.189 | 0.839 | Y | Y | Y | Y | N |
| Pknox1 | 18771 | Pbx/knotted 1 homeobox | Y | 1 | 0 | 1 | 0 | -0.161 | 0.906 | Y | Y | Y | N | N |
| Elk3 | 13713 | ELK3, member of ETS oncogene family | Y | 1 | 0 | 1 | 0 | -0.322 | 0.906 | Y | Y | Y | N | Y |
| Nav2 | 78286 | neuron navigator 2 | Y | 1 | 0 | 1 | 0 | -0.02 | 0.727 | Y | Y | Y | N | N |
| Mbtps1 | 56453 | membrane-bound transcription factor peptidase, site 1 | Y | 1 | 0 | 0 | 1 | -0.017 | 0.41 | Y | N | N | Y | N |
| Arhgef7 | 54126 | Rho guanine nucleotide exchange factor (GEF7) | Y | 1 | 0 | 1 | 0 | -0.097 | 0.842 | Y | Y | Y | N | N |
| Ctsa | 19025 | cathepsin A | Y | 1 | 0 | 1 | 0 | -0.072 | 0.843 | Y | Y | Y | N | N |
| Tnrc6c | 217351 | trinucleotide repeat containing 6C | Y | 1 | 0 | 1 | 0 | -0.076 | 0.714 | Y | Y | Y | N | N |
| Pfn2 | 18645 | profilin 2 | Y | 2 | 0 | 2 | 0 | -0.209 | 0.883 | Y | Y | Y | N | N |
| Nr2c1 | 22025 | nuclear receptor subfamily 2, group C, member 1 | Y | 1 | 0 | 1 | 0 | -0.181 | 0.881 | Y | Y | Y | N | N |
| Atg7 | 74244 | autophagy related 7 | Y | 1 | 1 | 0 | 0 | -0.322 | 0.967 | N | N | N | N | Y |
| Shoc2 | 56392 | soc-2 (suppressor of clear) homolog (C. elegans) | Y | 1 | 0 | 1 | 0 | -0.026 | 0.557 | Y | Y | Y | N | N |
| Rnf128 | 66889 | ring finger protein 128 | Y | 1 | 1 | 0 | 0 | -0.239 | 0.701 | Y | N | N | N | Y |
| Strbp | 20744 | spermatid perinuclear RNA binding protein | Y | 1 | 0 | 0 | 1 | -0.152 | 0.516 | Y | Y | Y | Y | N |
| Abl2 | 11352 | v-abl Abelson murine leukemia viral oncogene 2 (arg, Abelson-related gene) | Y | 1 | 0 | 1 | 0 | -0.02 | 0.208 | Y | Y | Y | N | N |
| Phf1 | 21652 | PHD finger protein 1 | Y | 1 | 0 | 1 | 0 | -0.106 | 0.663 | Y | Y | Y | N | N |
| Map3k1 | 26401 | mitogen-activated protein kinase kinase kinase 1 | Y | 1 | 0 | 1 | 0 | -0.137 | 0.405 | Y | Y | Y | N | N |
| Zfp664 | 269704 | zinc finger protein 664 | Y | 1 | 0 | 0 | 1 | -0.109 | 0 | Y | N | N | Y | N |
| Zdhhc8 | 27801 | zinc finger, DHHC domain containing 8 | Y | 1 | 1 | 0 | 0 | -0.151 | 0.596 | N | N | N | N | Y |
| Zfp91 | 109910 | zinc finger protein 91 | Y | 2 | 1 | 1 | 0 | -0.207 | 0.994 | Y | Y | Y | Y | Y |
| Acsl4 | 50790 | acyl-CoA synthetase long-chain family member 4 | Y | 1 | 0 | 1 | 0 | -0.08 | 0.897 | Y | Y | Y | N | N |
| Mapk9 | 26420 | mitogen-activated protein kinase 9 | Y | 1 | 0 | 1 | 0 | -0.117 | 0.416 | Y | Y | Y | Y | N |
| S1pr1 | 13609 | sphingosine-1-phosphate receptor 1 | Y | 1 | 0 | 1 | 0 | -0.128 | 0.252 | Y | Y | Y | N | N |
| Rab8b | 235442 | RAB8B, member RAS oncogene family | Y | 1 | 1 | 0 | 0 | -0.272 | 0.665 | Y | Y | N | N | Y |
| Fndc3a | 319448 | fibronectin type III domain containing 3A | Y | 1 | 0 | 1 | 0 | -0.036 | 0.649 | Y | Y | Y | N | N |
| Phc3 | 241915 | polyhomeotic-like 3 (Drosophila) | Y | 1 | 0 | 1 | 0 | -0.17 | 0.905 | Y | Y | Y | N | N |
| Arhgap12 | 75415 | Rho GTPase activating protein 12 | Y | 2 | 0 | 2 | 0 | -0.254 | 0.886 | Y | Y | Y | N | N |
| Psd | 73728 | pleckstrin and Sec7 domain containing | Y | 1 | 0 | 1 | 0 | -0.168 | 0.899 | Y | Y | Y | N | N |
| Mat2b | 108645 | methionine adenosyltransferase II, beta | Y | 1 | 0 | 1 | 0 | -0.192 | 0.412 | Y | Y | Y | N | N |
| Ythdf3 | 229096 | YTH domain family 3 | Y | 1 | 0 | 1 | 0 | -0.152 | 0.773 | Y | Y | Y | Y | N |
| Atg14 | 100504663 | autophagy related 14 | Y | 1 | 0 | 1 | 0 | -0.045 | 0.844 | Y | Y | Y | N | N |
| Pafah1b2 | 18475 | platelet-activating factor acetylhydrolase, isoform 1b, subunit 2 | Y | 1 | 0 | 1 | 0 | -0.117 | 0.894 | Y | Y | Y | N | N |
| Dync1li2 | 234663 | dynein, cytoplasmic 1 light intermediate chain 2 | Y | 1 | 0 | 1 | 0 | -0.04 | 0.398 | Y | Y | Y | N | N |
| Ash1l | 192195 | ash1 (absent, small, or homeotic)-like (Drosophila) | Y | 1 | 0 | 1 | 0 | -0.02 | 0.32 | Y | Y | Y | N | N |
| Bambi | 68010 | BMP and activin membrane-bound inhibitor, homolog (Xenopus laevis) | Y | 1 | 0 | 1 | 0 | -0.167 | 0.907 | Y | Y | Y | N | N |
| Eif4g2 | 13690 | eukaryotic translation initiation factor 4, gamma 2 | Y | 1 | 0 | 1 | 0 | -0.139 | 0.907 | Y | Y | Y | N | N |
| Eif4h | 22384 | eukaryotic translation initiation factor 4H | Y | 1 | 0 | 1 | 0 | -0.02 | 0.433 | Y | Y | Y | N | N |
| Wac | 225131 | WW domain containing adaptor with coiled-coil | Y | 1 | 0 | 1 | 0 | -0.076 | 0.631 | Y | Y | Y | N | N |
| March6 | 223455 | membrane-associated ring finger (C3HC4) 6 | Y | 1 | 0 | 0 | 1 | -0.041 | 0.516 | Y | Y | N | Y | N |
| Scamp2 | 24044 | secretory carrier membrane protein 2 | Y | 1 | 0 | 1 | 0 | -0.088 | 0.84 | Y | Y | Y | N | N |
| Tspan9 | 109246 | tetraspanin 9 | Y | 1 | 0 | 1 | 0 | -0.156 | 0.882 | Y | Y | Y | N | N |
| F2r | 14062 | coagulation factor II (thrombin) receptor | Y | 1 | 0 | 1 | 0 | -0.11 | 0.206 | Y | Y | Y | N | N |
| Klhl12 | 240756 | kelch-like 12 (Drosophila) | Y | 1 | 0 | 1 | 0 | -0.114 | 0.255 | Y | Y | Y | N | N |
| Ap3d1 | 11776 | adaptor-related protein complex 3, delta 1 subunit | Y | 1 | 0 | 0 | 1 | -0.021 | 0.515 | Y | N | N | Y | N |
| Kif13a | 16553 | kinesin family member 13A | Y | 1 | 0 | 1 | 0 | -0.085 | 0.384 | Y | Y | Y | N | N |
| Dyrk1a | 13548 | dual-specificity tyrosine-(Y)-phosphorylation regulated kinase 1a | Y | 1 | 0 | 1 | 0 | -0.02 | 0.773 | Y | Y | Y | N | N |
| Rps6ka1 | 20111 | ribosomal protein S6 kinase polypeptide 1 | Y | 1 | 0 | 1 | 0 | -0.119 | 0.603 | Y | Y | Y | N | N |
| Ccdc50 | 67501 | coiled-coil domain containing 50 | Y | 1 | 1 | 0 | 0 | -0.04 | 0.843 | Y | Y | N | Y | Y |
| Cald1 | 109624 | caldesmon 1 | Y | 1 | 1 | 0 | 0 | -0.238 | 0.954 | N | N | N | N | Y |
| Med17 | 234959 | mediator complex subunit 17 | Y | 1 | 0 | 0 | 1 | -0.04 | 0.507 | Y | N | N | Y | N |
| Tgfbrap1 | 73122 | transforming growth factor, beta receptor associated protein 1 | Y | 1 | 0 | 1 | 0 | -0.02 | 0.375 | Y | Y | Y | N | N |
| Ino80 | 68142 | INO80 homolog (S. cerevisiae) | Y | 1 | 0 | 1 | 0 | -0.135 | 0.751 | Y | Y | Y | N | N |
| Eif5b | 226982 | eukaryotic translation initiation factor 5B | Y | 2 | 0 | 2 | 0 | -0.073 | 0.988 | Y | Y | Y | Y | N |
| Hs2st1 | 23908 | heparan sulfate 2-O-sulfotransferase 1 | Y | 1 | 1 | 0 | 0 | -0.03 | 0 | Y | Y | N | N | Y |
| Dmtf1 | 23857 | cyclin D binding myb-like transcription factor 1 | Y | 1 | 0 | 1 | 0 | -0.228 | 0.105 | Y | Y | Y | N | N |
| Spopl | 76857 | speckle-type POZ protein-like | Y | 1 | 0 | 1 | 0 | -0.169 | 0.359 | Y | Y | Y | N | N |
| Mast3 | 546071 | microtubule associated serine/threonine kinase 3 | Y | 1 | 0 | 1 | 0 | -0.085 | 0.319 | Y | Y | Y | N | N |
| Mecp2 | 17257 | methyl CpG binding protein 2 | Y | 1 | 0 | 1 | 0 | -0.182 | 0.907 | Y | Y | Y | N | N |
| Ppp2r2a | 71978 | protein phosphatase 2 (formerly 2A), regulatory subunit B (PR 52), alpha isoform | Y | 1 | 0 | 1 | 0 | -0.21 | 0.873 | Y | Y | Y | N | N |
| Rad21 | 19357 | RAD21 homolog (S. pombe) | Y | 1 | 0 | 1 | 0 | -0.117 | 0.345 | Y | Y | Y | Y | N |
| Tmem50b | 77975 | transmembrane protein 50B | Y | 1 | 0 | 1 | 0 | -0.102 | 0.176 | Y | Y | Y | Y | N |
| Pcyt1b | 236899 | phosphate cytidylyltransferase 1, choline, beta isoform | Y | 1 | 0 | 1 | 0 | -0.05 | 0.635 | Y | Y | Y | Y | N |
| Pten | 19211 | phosphatase and tensin homolog | Y | 1 | 0 | 1 | 0 | -0.139 | 0.894 | Y | Y | Y | Y | Y |
| Rnf13 | 24017 | ring finger protein 13 | Y | 1 | 0 | 0 | 1 | -0.057 | 0.508 | Y | N | N | Y | N |
| Taok1 | 216965 | TAO kinase 1 | Y | 1 | 0 | 1 | 0 | -0.03 | 0.823 | Y | Y | Y | Y | N |
| Rnft1 | 76892 | ring finger protein, transmembrane 1 | Y | 1 | 1 | 0 | 0 | -0.03 | 0.149 | Y | N | N | N | Y |
| Ubr5 | 70790 | ubiquitin protein ligase E3 component n-recognin 5 | Y | 1 | 0 | 1 | 0 | -0.157 | 0.135 | Y | Y | Y | N | N |
| Nanos1 | 332397 | nanos homolog 1 (Drosophila) | Y | 1 | 1 | 0 | 0 | -0.297 | 0.963 | Y | Y | Y | N | Y |
| Cybb | 13058 | cytochrome b-245, beta polypeptide | Y | 1 | 0 | 1 | 0 | -0.172 | 0.44 | Y | Y | Y | N | N |
| Phip | 83946 | pleckstrin homology domain interacting protein | Y | 1 | 0 | 1 | 0 | -0.02 | 0.458 | Y | Y | Y | N | N |
| Aak1 | 269774 | AP2 associated kinase 1 | Y | 2 | 2 | 0 | 0 | -0.1 | 0.998 | Y | Y | Y | N | Y |
| Fam84b | 399603 | family with sequence similarity 84, member B | Y | 1 | 1 | 0 | 0 | -0.06 | 0.958 | N | N | N | N | Y |
| Alkbh5 | 268420 | alkB, alkylation repair homolog 5 (E. coli) | Y | 1 | 0 | 1 | 0 | -0.02 | 0.905 | Y | Y | Y | N | N |
| Tceb3 | 27224 | transcription elongation factor B (SIII), polypeptide 3 | Y | 1 | 1 | 0 | 0 | -0.061 | 0.822 | Y | N | N | N | Y |
| Rapgef4 | 56508 | Rap guanine nucleotide exchange factor (GEF) 4 | Y | 1 | 0 | 1 | 0 | -0.192 | 0.54 | Y | Y | Y | N | N |
| Prickle3 | 54630 | prickle homolog 3 (Drosophila) | Y | 1 | 0 | 1 | 0 | -0.154 | 0.273 | Y | Y | Y | N | N |
| Rabep1 | 54189 | rabaptin, RAB GTPase binding effector protein 1 | Y | 1 | 0 | 1 | 0 | -0.031 | 0.441 | Y | Y | Y | N | N |
| Mynn | 80732 | myoneurin | Y | 1 | 0 | 1 | 0 | -0.149 | 0.604 | Y | Y | Y | N | N |
| Sh3pxd2a | 14218 | SH3 and PX domains 2A | Y | 1 | 1 | 0 | 0 | -0.05 | 0.967 | Y | Y | Y | N | Y |
| Dock4 | 238130 | dedicator of cytokinesis 4 | Y | 1 | 1 | 0 | 0 | -0.297 | 0.889 | Y | N | N | N | Y |
| Itch | 16396 | itchy, E3 ubiquitin protein ligase | Y | 1 | 0 | 1 | 0 | -0.031 | 0.3 | Y | Y | Y | N | N |
| Slc39a9 | 328133 | solute carrier family 39 (zinc transporter), member 9 | Y | 1 | 0 | 0 | 1 | -0.111 | 0.429 | Y | N | N | Y | N |
| Ccnt2 | 72949 | cyclin T2 | Y | 1 | 0 | 1 | 0 | -0.124 | 0.15 | Y | Y | Y | N | N |
| Tnrc6b | 213988 | trinucleotide repeat containing 6b | Y | 2 | 0 | 1 | 1 | -0.046 | 0.94 | Y | Y | Y | Y | N |
| Ccng2 | 12452 | cyclin G2 | Y | 1 | 1 | 0 | 0 | -0.277 | 0.068 | Y | N | N | N | Y |
| Creb1 | 12912 | cAMP responsive element binding protein 1 | Y | 2 | 0 | 2 | 0 | -0.098 | 0.919 | Y | Y | Y | N | N |
| Dirc2 | 224132 | disrupted in renal carcinoma 2 (human) | Y | 1 | 0 | 1 | 0 | -0.136 | 0.849 | Y | Y | Y | Y | N |
| Ahnak | 66395 | AHNAK nucleoprotein (desmoyokin) | Y | 1 | 1 | 0 | 0 | -0.285 | 0.92 | N | N | N | N | Y |
| Sh3bp5 | 24056 | SH3-domain binding protein 5 (BTK-associated) | Y | 1 | 0 | 1 | 0 | -0.097 | 0.596 | Y | Y | Y | N | N |
| Hook3 | 320191 | hook homolog 3 (Drosophila) | Y | 1 | 0 | 1 | 0 | -0.254 | 0.851 | Y | Y | Y | N | N |
| Fam3c | 27999 | family with sequence similarity 3, member C | Y | 1 | 0 | 1 | 0 | -0.068 | 0.829 | Y | Y | Y | N | N |
| Bahd1 | 228536 | bromo adjacent homology domain containing 1 | Y | 1 | 0 | 1 | 0 | -0.216 | 0.905 | Y | Y | Y | N | N |
| Egr2 | 13654 | early growth response 2 | Y | 1 | 1 | 0 | 0 | -0.184 | 0.04 | N | N | N | N | Y |
| Arl5a | 75423 | ADP-ribosylation factor-like 5A | Y | 1 | 1 | 0 | 0 | -0.03 | 0.741 | Y | N | N | N | Y |
| Ankrd50 | 99696 | ankyrin repeat domain 50 | Y | 1 | 0 | 1 | 0 | -0.02 | 0.577 | Y | Y | Y | N | N |
| Ankrd10 | 102334 | ankyrin repeat domain 10 | Y | 1 | 0 | 1 | 0 | 0 | 0.167 | Y | Y | Y | N | N |
| Ppp2ca | 19052 | protein phosphatase 2 (formerly 2A), catalytic subunit, alpha isoform | Y | 1 | 0 | 0 | 1 | -0.019 | 0 | Y | Y | N | Y | N |
| Alg13 | 67574 | asparagine-linked glycosylation 13 | Y | 1 | 0 | 0 | 1 | 0 | 0.35 | Y | Y | N | Y | N |
| Rsbn1 | 229675 | rosbin, round spermatid basic protein 1 | Y | 1 | 0 | 1 | 0 | -0.189 | 0.309 | Y | Y | Y | Y | N |
| Dstyk | 213452 | dual serine/threonine and tyrosine protein kinase | Y | 1 | 0 | 0 | 1 | -0.01 | 0.466 | Y | Y | N | Y | N |
| Ptprj | 19271 | protein tyrosine phosphatase, receptor type, J | Y | 2 | 0 | 2 | 0 | -0.04 | 0.947 | Y | Y | Y | N | N |
| Myo1d | 338367 | myosin ID | Y | 1 | 0 | 1 | 0 | -0.09 | 0.775 | Y | Y | Y | N | N |
| Nr2c2 | 22026 | nuclear receptor subfamily 2, group C, member 2 | Y | 1 | 0 | 1 | 0 | -0.321 | 0.907 | Y | Y | Y | Y | N |
| Cand1 | 71902 | cullin associated and neddylation disassociated 1 | Y | 1 | 0 | 0 | 1 | -0.051 | 0.124 | Y | Y | N | Y | N |
| Ino80d | 227195 | INO80 complex subunit D | Y | 1 | 0 | 1 | 0 | -0.03 | 0.756 | Y | Y | Y | Y | N |
| Ppp3r1 | 19058 | protein phosphatase 3, regulatory subunit B, alpha isoform (calcineurin B, type I) | Y | 1 | 1 | 0 | 0 | -0.293 | 0.886 | Y | N | N | N | Y |
| Stxbp5 | 78808 | syntaxin binding protein 5 (tomosyn) | Y | 1 | 0 | 1 | 0 | -0.104 | 0.849 | Y | Y | Y | N | N |
| Fxr1 | 14359 | fragile X mental retardation gene 1, autosomal homolog | Y | 1 | 0 | 1 | 0 | -0.101 | 0.529 | Y | Y | Y | N | N |
| Ddx5 | 13207 | DEAD (Asp-Glu-Ala-Asp) box polypeptide 5 | Y | 1 | 0 | 1 | 0 | -0.183 | 0.905 | Y | Y | Y | N | N |
| Ephb4 | 13846 | Eph receptor B4 | Y | 1 | 0 | 1 | 0 | -0.128 | 0.856 | Y | Y | Y | N | N |
| Ubn2 | 320538 | ubinuclein 2 | Y | 1 | 0 | 1 | 0 | -0.04 | 0.211 | Y | Y | Y | N | N |
| Tanc2 | 77097 | tetratricopeptide repeat, ankyrin repeat and coiled-coil containing 2 | Y | 1 | 1 | 0 | 0 | -0.05 | 0.947 | Y | Y | Y | N | Y |
| Sf3b1 | 81898 | splicing factor 3b, subunit 1 | Y | 1 | 0 | 1 | 0 | -0.33 | 0.339 | Y | Y | Y | N | N |
| Pard6b | 58220 | par-6 (partitioning defective 6) homolog beta (C. elegans) | Y | 1 | 0 | 1 | 0 | -0.12 | 0.875 | Y | Y | Y | N | N |
| Rarb | 218772 | retinoic acid receptor, beta | Y | 1 | 0 | 0 | 1 | -0.026 | 0.394 | Y | N | N | Y | N |
| Zfp697 | 242109 | zinc finger protein 697 | Y | 1 | 0 | 1 | 0 | -0.075 | 0.662 | Y | Y | Y | Y | N |
| Ubc | 22190 | ubiquitin C | Y | 1 | 0 | 1 | 0 | -0.211 | 0.682 | Y | Y | Y | N | N |
| Pafah1b1 | 18472 | platelet-activating factor acetylhydrolase, isoform 1b, subunit 1 | Y | 1 | 1 | 0 | 0 | -0.056 | 0.829 | N | Y | N | N | Y |
| Unk | 217331 | unkempt homolog (Drosophila) | Y | 1 | 0 | 1 | 0 | -0.196 | 0.881 | Y | Y | Y | N | N |
| Myo9a | 270163 | myosin IXa | Y | 1 | 0 | 1 | 0 | -0.201 | 0.869 | Y | Y | Y | Y | N |
| Spty2d1 | 101685 | SPT2, Suppressor of Ty, domain containing 1 (S. cerevisiae) | Y | 1 | 0 | 1 | 0 | -0.05 | 0.613 | Y | Y | Y | N | N |
| Med13 | 327987 | mediator complex subunit 13 | Y | 1 | 0 | 0 | 1 | -0.074 | 0.31 | Y | N | N | Y | N |
| Rnf150 | 330812 | ring finger protein 150 | Y | 1 | 1 | 0 | 0 | -0.271 | 0.967 | Y | Y | N | N | Y |
| Nek9 | 217718 | NIMA (never in mitosis gene a)-related expressed kinase 9 | Y | 2 | 0 | 2 | 0 | -0.252 | 0.549 | Y | Y | Y | N | N |
| Bmpr2 | 12168 | bone morphogenetic protein receptor, type II (serine/threonine kinase) | Y | 1 | 0 | 1 | 0 | -0.211 | 0.404 | N | N | N | N | N |
| Zc3h7b | 20286 | zinc finger CCCH type containing 7B | Y | 1 | 0 | 0 | 1 | -0.078 | 0.505 | Y | N | N | Y | N |
| Asf1a | 66403 | ASF1 anti-silencing function 1 homolog A (S. cerevisiae) | Y | 1 | 0 | 1 | 0 | -0.166 | 0.134 | Y | Y | Y | N | N |
| Litaf | 56722 | LPS-induced TN factor | Y | 1 | 0 | 0 | 1 | -0.045 | 0.202 | Y | N | N | Y | N |
| Sp1 | 20683 | trans-acting transcription factor 1 | Y | 1 | 0 | 1 | 0 | -0.04 | 0.58 | Y | Y | Y | N | N |
| Fam117b | 72750 | family with sequence similarity 117, member B | Y | 1 | 1 | 0 | 0 | -0.216 | 0.804 | N | Y | N | N | Y |
| Arl4a | 11861 | ADP-ribosylation factor-like 4A | Y | 1 | 0 | 1 | 0 | -0.02 | 0.464 | Y | N | N | N | N |
| Ppp6r3 | 52036 | protein phosphatase 6, regulatory subunit 3 | Y | 1 | 0 | 1 | 0 | -0.051 | 0.058 | Y | Y | Y | N | N |
| Flt1 | 14254 | FMS-like tyrosine kinase 1 | Y | 1 | 1 | 0 | 0 | -0.109 | 0.92 | Y | Y | N | N | Y |
| Phf6 | 70998 | PHD finger protein 6 | Y | 1 | 1 | 0 | 0 | -0.03 | 0.714 | Y | N | N | N | Y |
| Zfp365 | 216049 | zinc finger protein 365 | Y | 1 | 0 | 1 | 0 | -0.061 | 0.867 | Y | Y | Y | N | N |
| Fzd7 | 14369 | frizzled homolog 7 (Drosophila) | Y | 1 | 0 | 0 | 1 | -0.045 | 0.506 | Y | N | N | Y | N |
| Sntb2 | 20650 | syntrophin, basic 2 | Y | 1 | 0 | 1 | 0 | -0.171 | 0.852 | Y | Y | Y | N | N |
| Pip4k2c | 117150 | phosphatidylinositol-5-phosphate 4-kinase, type II, gamma | Y | 1 | 0 | 1 | 0 | -0.129 | 0.315 | Y | Y | Y | N | N |
| Srpk2 | 20817 | serine/arginine-rich protein specific kinase 2 | Y | 1 | 1 | 0 | 0 | -0.114 | 0.966 | Y | Y | N | N | Y |
| Actr1a | 54130 | ARP1 actin-related protein 1A, centractin alpha | Y | 1 | 0 | 0 | 1 | -0.064 | 0.394 | Y | N | N | Y | N |
| 4933411K20Rik | 66756 | RIKEN cDNA 4933411K20 gene | Y | 1 | 0 | 1 | 0 | -0.096 | 0.361 | Y | Y | Y | N | N |
| Mterfd2 | 69821 | MTERF domain containing 2 | Y | 1 | 1 | 0 | 0 | -0.438 | 0.526 | Y | Y | Y | N | Y |
| Abca1 | 11303 | ATP-binding cassette, sub-family A (ABC1), member 1 | Y | 1 | 1 | 0 | 0 | -0.254 | 0.965 | Y | Y | N | N | Y |
| Rpa2 | 19891 | replication protein A2 | Y | 1 | 0 | 1 | 0 | -0.068 | 0.159 | Y | Y | Y | N | N |
| Fbxw11 | 103583 | F-box and WD-40 domain protein 11 | Y | 1 | 0 | 1 | 0 | 0 | 0.787 | Y | Y | Y | N | N |
| Slc6a9 | 14664 | solute carrier family 6 (neurotransmitter transporter, glycine), member 9 | Y | 1 | 0 | 1 | 0 | -0.063 | 0.81 | Y | Y | Y | N | N |
| Gxylt1 | 223827 | glucoside xylosyltransferase 1 | Y | 2 | 0 | 0 | 2 | -0.02 | 0.762 | Y | Y | N | Y | N |
| Smad5 | 17129 | SMAD family member 5 | Y | 1 | 0 | 1 | 0 | -0.108 | 0.905 | Y | Y | Y | N | N |
| Ugdh | 22235 | UDP-glucose dehydrogenase | Y | 1 | 1 | 0 | 0 | -0.256 | 0.936 | N | N | N | N | Y |
| Nrp2 | 18187 | neuropilin 2 | Y | 1 | 0 | 1 | 0 | -0.198 | 0.697 | Y | Y | Y | N | N |
| Plcb1 | 18795 | phospholipase C, beta 1 | Y | 1 | 0 | 1 | 0 | -0.071 | 0.298 | Y | Y | Y | N | N |
| Usp32 | 237898 | ubiquitin specific peptidase 32 | Y | 1 | 1 | 0 | 0 | -0.118 | 0 | Y | N | N | N | N |
| Sar1b | 66397 | SAR1 gene homolog B (S. cerevisiae) | Y | 1 | 0 | 1 | 0 | -0.188 | 0.508 | Y | Y | Y | N | N |
| Ppp2r1b | 73699 | protein phosphatase 2 (formerly 2A), regulatory subunit A (PR 65), beta isoform | Y | 1 | 0 | 1 | 0 | -0.211 | 0.869 | Y | Y | Y | Y | N |
| Kazn | 71529 | kazrin, periplakin interacting protein | Y | 1 | 0 | 1 | 0 | -0.094 | 0.134 | Y | Y | Y | Y | N |
| Cyp26b1 | 232174 | cytochrome P450, family 26, subfamily b, polypeptide 1 | Y | 1 | 0 | 1 | 0 | -0.109 | 0.907 | Y | Y | Y | N | N |
| Pcbp2 | 18521 | poly(rC) binding protein 2 | Y | 1 | 0 | 0 | 1 | -0.06 | 0.477 | Y | N | N | Y | N |
| Fsd1l | 319636 | fibronectin type III and SPRY domain containing 1-like | Y | 1 | 1 | 0 | 0 | -0.03 | 0.967 | Y | Y | N | N | Y |
| Rrm2 | 20135 | ribonucleotide reductase M2 | Y | 1 | 0 | 1 | 0 | -0.053 | 0.893 | Y | Y | Y | N | N |
| Wwtr1 | 97064 | WW domain containing transcription regulator 1 | Y | 1 | 1 | 0 | 0 | -0.03 | 0.878 | N | Y | N | N | Y |
| Hmgb3 | 15354 | high mobility group box 3 | Y | 1 | 0 | 1 | 0 | -0.108 | 0.37 | Y | Y | Y | N | N |
| 2700081O15Rik | 108899 | RIKEN cDNA 2700081O15 gene | Y | 1 | 0 | 1 | 0 | -0.076 | 0.686 | Y | Y | Y | N | N |
| Kpna4 | 16649 | karyopherin (importin) alpha 4 | Y | 1 | 0 | 0 | 1 | -0.01 | 0.411 | Y | Y | N | Y | N |
| Timp2 | 21858 | tissue inhibitor of metalloproteinase 2 | Y | 1 | 0 | 1 | 0 | -0.162 | 0.881 | Y | Y | Y | N | N |
| Rgl1 | 19731 | ral guanine nucleotide dissociation stimulator,-like 1 | Y | 1 | 0 | 1 | 0 | -0.222 | 0.283 | Y | Y | Y | N | N |
| Epas1 | 13819 | endothelial PAS domain protein 1 | Y | 1 | 0 | 1 | 0 | -0.107 | 0.084 | Y | Y | Y | Y | N |
| Serp1 | 28146 | stress-associated endoplasmic reticulum protein 1 | Y | 1 | 0 | 1 | 0 | -0.132 | 0.447 | Y | Y | Y | N | N |
| Rlim | 19820 | ring finger protein, LIM domain interacting | Y | 1 | 1 | 0 | 0 | -0.05 | 0.4 | Y | Y | Y | N | Y |
| Qk | 19317 | quaking | Y | 1 | 0 | 1 | 0 | -0.205 | 0.738 | Y | Y | Y | N | N |
| Senp1 | 223870 | SUMO1/sentrin specific peptidase 1 | Y | 1 | 0 | 1 | 0 | -0.02 | 0.659 | Y | Y | Y | N | N |
| Smoc1 | 64075 | SPARC related modular calcium binding 1 | Y | 1 | 0 | 1 | 0 | -0.202 | 0.906 | Y | Y | Y | N | N |
| Rnf38 | 73469 | ring finger protein 38 | Y | 1 | 0 | 1 | 0 | -0.02 | 0.756 | Y | Y | Y | N | N |
| Adipor2 | 68465 | adiponectin receptor 2 | Y | 1 | 0 | 1 | 0 | -0.02 | 0.748 | Y | Y | Y | N | N |
| Sema3c | 20348 | sema domain, immunoglobulin domain (Ig), short basic domain, secreted, (semaphorin) 3C | Y | 1 | 0 | 1 | 0 | -0.184 | 0.668 | Y | Y | Y | N | N |
| Lima1 | 65970 | LIM domain and actin binding 1 | Y | 1 | 1 | 0 | 0 | -0.121 | 0.965 | Y | Y | Y | N | Y |
| Tcf4 | 21413 | transcription factor 4 | Y | 1 | 0 | 1 | 0 | -0.04 | 0.442 | Y | Y | Y | N | N |
| Fam168a | 319604 | family with sequence similarity 168, member A | Y | 1 | 0 | 0 | 1 | -0.033 | 0.516 | Y | Y | N | Y | N |
| Nedd4l | 83814 | neural precursor cell expressed, developmentally down-regulated gene 4-like | Y | 2 | 1 | 1 | 0 | -0.1 | 0.99 | Y | Y | Y | Y | Y |
| Mier3 | 218613 | mesoderm induction early response 1, family member 3 | Y | 1 | 0 | 0 | 1 | -0.01 | 0 | Y | Y | N | Y | N |
| Rb1cc1 | 12421 | RB1-inducible coiled-coil 1 | Y | 1 | 0 | 1 | 0 | -0.307 | 0.656 | Y | Y | Y | N | N |
| Fam199x | 245622 | family with sequence similarity 199, X-linked | Y | 1 | 0 | 1 | 0 | -0.062 | 0.905 | Y | Y | Y | Y | N |
| Pde3b | 18576 | phosphodiesterase 3B, cGMP-inhibited | Y | 1 | 0 | 1 | 0 | -0.285 | 0.902 | Y | Y | Y | Y | N |
| Akap11 | 219181 | A kinase (PRKA) anchor protein 11 | Y | 1 | 0 | 1 | 0 | -0.02 | 0.511 | Y | Y | Y | N | N |
| Slc25a27 | 74011 | solute carrier family 25, member 27 | Y | 1 | 1 | 0 | 0 | -0.243 | 0.803 | Y | N | N | N | Y |
| Elovl6 | 170439 | ELOVL family member 6, elongation of long chain fatty acids (yeast) | Y | 1 | 1 | 0 | 0 | -0.03 | 0.874 | N | Y | N | N | Y |
| Nacc2 | 67991 | nucleus accumbens associated 2, BEN and BTB (POZ) domain containing | Y | 1 | 1 | 0 | 0 | -0.03 | 0.741 | N | Y | N | N | Y |
| Rap2c | 72065 | RAP2C, member of RAS oncogene family | Y | 1 | 1 | 0 | 0 | -0.078 | 0.295 | Y | N | N | N | Y |
| Maml1 | 103806 | mastermind like 1 (Drosophila) | Y | 1 | 0 | 1 | 0 | -0.076 | 0.799 | Y | Y | Y | Y | N |
| Vasp | 22323 | vasodilator-stimulated phosphoprotein | Y | 1 | 0 | 0 | 1 | -0.022 | 0.31 | Y | N | N | Y | N |
| Tns1 | 21961 | tensin 1 | Y | 1 | 0 | 1 | 0 | -0.02 | 0.891 | Y | Y | Y | N | N |
| Nucks1 | 98415 | nuclear casein kinase and cyclin-dependent kinase substrate 1 | Y | 1 | 0 | 0 | 1 | -0.068 | 0.216 | Y | Y | N | Y | N |
| Rnf2 | 19821 | ring finger protein 2 | Y | 1 | 1 | 0 | 0 | -0.095 | 0.848 | N | N | N | N | Y |
| Pdlim5 | 56376 | PDZ and LIM domain 5 | Y | 1 | 1 | 0 | 0 | -0.03 | 0.937 | Y | Y | N | N | Y |
| Akap13 | 75547 | A kinase (PRKA) anchor protein 13 | Y | 1 | 1 | 0 | 0 | -0.03 | 0.943 | N | Y | N | N | Y |
| Mtss1l | 244654 | metastasis suppressor 1-like | Y | 1 | 0 | 0 | 1 | -0.01 | 0.381 | Y | N | N | Y | N |
| Fzd3 | 14365 | frizzled homolog 3 (Drosophila) | Y | 1 | 1 | 0 | 0 | -0.347 | 0.966 | Y | N | N | N | N |
| Tet2 | 214133 | tet methylcytosine dioxygenase 2 | Y | 1 | 0 | 1 | 0 | -0.067 | 0.177 | Y | Y | Y | Y | N |
| Synm | 233335 | synemin, intermediate filament protein | Y | 1 | 0 | 1 | 0 | -0.02 | 0.485 | Y | Y | Y | N | N |
| Ccnj | 240665 | cyclin J | Y | 1 | 0 | 1 | 0 | -0.02 | 0.593 | Y | Y | Y | N | N |
| Epb4.1 | 269587 | erythrocyte protein band 4.1 | Y | 1 | 0 | 1 | 0 | -0.028 | 0.537 | Y | Y | Y | N | N |
| Gpr146 | 80290 | G protein-coupled receptor 146 | Y | 1 | 0 | 1 | 0 | -0.19 | 0.297 | Y | Y | Y | N | N |
| Nfat5 | 54446 | nuclear factor of activated T cells 5 | Y | 1 | 1 | 0 | 0 | -0.04 | 0.928 | Y | Y | N | Y | Y |
| Cic | 71722 | capicua homolog (Drosophila) | Y | 1 | 0 | 1 | 0 | -0.027 | 0.793 | Y | Y | Y | N | N |
| Prr16 | 71373 | proline rich 16 | Y | 1 | 0 | 1 | 0 | -0.192 | 0.905 | Y | Y | Y | N | N |
| Stat3 | 20848 | signal transducer and activator of transcription 3 | Y | 2 | 0 | 2 | 0 | -0.216 | 0.834 | Y | Y | Y | N | N |
| Ptp4a2 | 19244 | protein tyrosine phosphatase 4a2 | Y | 1 | 0 | 1 | 0 | -0.053 | 0.186 | Y | Y | Y | N | N |
| Rapgefl1 | 268480 | Rap guanine nucleotide exchange factor (GEF)-like 1 | Y | 1 | 0 | 1 | 0 | -0.12 | 0.898 | Y | Y | Y | N | N |
| Trpv6 | 64177 | transient receptor potential cation channel, subfamily V, member 6 | Y | 1 | 1 | 0 | 0 | -0.28 | 0.966 | N | N | N | N | Y |
| Suv420h1 | 225888 | suppressor of variegation 4-20 homolog 1 (Drosophila) | Y | 2 | 0 | 1 | 1 | -0.05 | 0.843 | Y | Y | Y | Y | N |
| Nfib | 18028 | nuclear factor I/B | Y | 1 | 0 | 1 | 0 | -0.02 | 0.762 | Y | Y | Y | N | N |
| Etnk1 | 75320 | ethanolamine kinase 1 | Y | 1 | 0 | 1 | 0 | -0.05 | 0.584 | Y | Y | Y | N | Y |
| Foxa1 | 15375 | forkhead box A1 | Y | 1 | 0 | 1 | 0 | -0.134 | 0.826 | Y | Y | Y | N | N |
| Thra | 21833 | thyroid hormone receptor alpha | Y | 1 | 0 | 1 | 0 | -0.154 | 0.766 | Y | Y | Y | N | N |
| Bcl2l11 | 12125 | BCL2-like 11 (apoptosis facilitator) | Y | 1 | 1 | 0 | 0 | -0.05 | 0.951 | Y | Y | Y | N | Y |
| Igf2bp1 | 140486 | insulin-like growth factor 2 mRNA binding protein 1 | Y | 2 | 1 | 1 | 0 | -0.05 | 0.896 | Y | Y | Y | N | Y |
| Kdm6b | 216850 | KDM1 lysine (K)-specific demethylase 6B | Y | 1 | 0 | 1 | 0 | -0.043 | 0.792 | Y | Y | Y | N | N |
| Zfp385a | 29813 | zinc finger protein 385A | Y | 1 | 0 | 1 | 0 | -0.05 | 0.53 | Y | Y | Y | N | N |
| Slc4a4 | 54403 | solute carrier family 4 (anion exchanger), member 4 | Y | 1 | 0 | 1 | 0 | -0.089 | 0.9 | Y | Y | Y | N | N |
| Dnajb9 | 27362 | DnaJ (Hsp40) homolog, subfamily B, member 9 | Y | 1 | 0 | 1 | 0 | -0.162 | 0.83 | Y | Y | Y | N | N |
| Syt7 | 54525 | synaptotagmin VII | Y | 1 | 0 | 0 | 1 | -0.013 | 0.51 | Y | N | N | Y | N |
| Acpl2 | 235534 | acid phosphatase-like 2 | Y | 1 | 0 | 1 | 0 | -0.026 | 0.437 | Y | Y | Y | N | N |
| Zfp362 | 230761 | zinc finger protein 362 | Y | 1 | 0 | 1 | 0 | -0.12 | 0.868 | Y | Y | Y | N | N |
| Ypel2 | 77864 | yippee-like 2 (Drosophila) | Y | 1 | 0 | 1 | 0 | -0.023 | 0.846 | Y | Y | Y | N | N |
| Trps1 | 83925 | trichorhinophalangeal syndrome I (human) | Y | 1 | 0 | 1 | 0 | -0.02 | 0.815 | Y | Y | Y | N | N |
| Prex1 | 277360 | phosphatidylinositol-3,4,5-trisphosphate-dependent Rac exchange factor 1 | Y | 1 | 0 | 1 | 0 | -0.041 | 0.825 | Y | Y | Y | N | N |
| Plekha6 | 240753 | pleckstrin homology domain containing, family A member 6 | Y | 1 | 0 | 0 | 1 | -0.01 | 0.514 | Y | Y | N | Y | N |
| Shc4 | 271849 | SHC (Src homology 2 domain containing) family, member 4 | Y | 1 | 0 | 1 | 0 | -0.224 | 0.467 | Y | Y | Y | N | N |
| Nckap5 | 210356 | NCK-associated protein 5 | Y | 1 | 0 | 1 | 0 | -0.224 | 0.578 | Y | Y | Y | N | N |
| Tsc22d2 | 72033 | TSC22 domain family, member 2 | Y | 1 | 0 | 0 | 1 | -0.092 | 0.516 | Y | Y | Y | Y | N |
| Camta1 | 100072 | calmodulin binding transcription activator 1 | Y | 1 | 1 | 0 | 0 | -0.052 | 0.797 | N | N | N | N | Y |
| Pik3r1 | 18708 | phosphatidylinositol 3-kinase, regulatory subunit, polypeptide 1 (p85 alpha) | Y | 1 | 0 | 1 | 0 | -0.02 | 0.592 | Y | Y | Y | N | N |
| Tle4 | 21888 | transducin-like enhancer of split 4, homolog of Drosophila E(spl) | Y | 1 | 0 | 1 | 0 | -0.119 | 0.767 | Y | Y | Y | N | N |
| Dlc1 | 50768 | deleted in liver cancer 1 | Y | 1 | 0 | 0 | 1 | -0.01 | 0 | Y | N | N | Y | N |
| Csgalnact1 | 234356 | chondroitin sulfate N-acetylgalactosaminyltransferase 1 | Y | 1 | 0 | 1 | 0 | -0.175 | 0.758 | Y | Y | Y | N | N |
| Bnc2 | 242509 | basonuclin 2 | Y | 1 | 0 | 1 | 0 | -0.123 | 0.754 | N | N | N | N | N |
| Zfp661 | 72180 | zinc finger protein 661 | Y | 1 | 1 | 0 | 0 | -0.257 | 0.841 | N | Y | N | N | Y |
| Arap2 | 212285 | ArfGAP with RhoGAP domain, ankyrin repeat and PH domain 2 | Y | 1 | 0 | 1 | 0 | -0.148 | 0.884 | Y | Y | Y | N | N |
| Fat4 | 329628 | FAT tumor suppressor homolog 4 (Drosophila) | Y | 1 | 1 | 0 | 0 | -0.155 | 0.754 | N | Y | N | N | Y |
| Bend4 | 666938 | BEN domain containing 4 | Y | 1 | 1 | 0 | 0 | -0.07 | 0.82 | Y | N | N | Y | Y |
| Btg2 | 12227 | B cell translocation gene 2, anti-proliferative | Y | 1 | 0 | 0 | 1 | -0.01 | 0 | Y | N | N | Y | N |
| Klf12 | 16597 | Kruppel-like factor 12 | Y | 2 | 0 | 2 | 0 | -0.101 | 0.852 | N | N | N | N | N |
| Rassf2 | 215653 | Ras association (RalGDS/AF-6) domain family member 2 | Y | 1 | 0 | 1 | 0 | -0.231 | 0.884 | Y | Y | Y | N | N |
| Zfp704 | 170753 | zinc finger protein 704 | Y | 3 | 0 | 3 | 0 | -0.104 | 0.992 | Y | Y | Y | Y | N |
| Sobp | 109205 | sine oculis-binding protein homolog (Drosophila) | Y | 1 | 0 | 1 | 0 | -0.149 | 0.788 | Y | Y | Y | N | N |
| Dab2 | 13132 | disabled 2, mitogen-responsive phosphoprotein | Y | 1 | 0 | 1 | 0 | -0.178 | 0.895 | Y | Y | Y | N | N |
| Tet1 | 52463 | tet methylcytosine dioxygenase 1 | Y | 1 | 0 | 1 | 0 | -0.148 | 0.907 | Y | Y | Y | N | N |
| Sp8 | 320145 | trans-acting transcription factor 8 | Y | 1 | 0 | 1 | 0 | -0.02 | 0.305 | Y | Y | Y | N | N |
| Pls1 | 102502 | plastin 1 (I-isoform) | Y | 1 | 1 | 0 | 0 | -0.358 | 0 | Y | N | N | Y | Y |
| Arhgap26 | 71302 | Rho GTPase activating protein 26 | Y | 1 | 1 | 0 | 0 | -0.279 | 0.81 | Y | Y | N | N | Y |
| Plekha7 | 233765 | pleckstrin homology domain containing, family A member 7 | Y | 1 | 0 | 1 | 0 | -0.166 | 0.845 | Y | Y | Y | N | N |
| 2510009E07Rik | 72190 | RIKEN cDNA 2510009E07 gene | Y | 1 | 1 | 0 | 0 | -0.05 | 0.958 | Y | N | N | Y | Y |
