# Supplementary material for: Anti-miR-17 therapy delays tumorigenesis in MYC-driven hepatocellular carcinoma (HCC)
Source: Oncotarget. 2017 Nov 9;9(5):5517–28. doi: 10.18632/oncotarget.22342 (PMC5814155; doi:10.18632/oncotarget.22342)
Supplement: Supplementary file 4 [file oncotarget-09-5517-s004.docx]

**Supplementary Table 3: Differentially expressed genes upon MYC inactivation in MYC conditional cell lines**

| **TargetID** | **p-value** | **q-value** | **Fold change** |
| --- | --- | --- | --- |
| RPLP1 | 1.15E-07 | 0.003542 | 1.155086 |
| GLS2 | 4.58E-06 | 0.070707 | 0.755481 |
| RAD18 | 2.75E-05 | 0.227398 | 0.392573 |
| 2310014H01RIK | 2.99E-05 | 0.227398 | 1.670948 |
| LOC100048037 | 4.20E-05 | 0.227398 | 1.922522 |
| PYGB | 4.79E-05 | 0.227398 | 1.272148 |
| E030042C16RIK | 5.16E-05 | 0.227398 | 0.770126 |
| 0910001L09RIK | 8.97E-05 | 0.346109 | 1.755239 |
| PRMT2 | 0.000113 | 0.354677 | 1.416503 |
| CCL7 | 0.000115 | 0.354677 | 2.000647 |
| LGR4 | 0.000156 | 0.421739 | 0.874239 |
| CAP1 | 0.000164 | 0.421739 | 1.516067 |
| VAC14 | 0.000222 | 0.438692 | 1.207202 |
| 6130401J04RIK | 0.000226 | 0.438692 | 0.887037 |
| C920030L09RIK | 0.000243 | 0.438692 | 1.015507 |
| HPBRII4 | 0.000246 | 0.438692 | 0.970523 |
| SYDE1 | 0.000247 | 0.438692 | 1.372684 |
| NACA | 0.000269 | 0.438692 | 0.816014 |
| F730023C13RIK | 0.000284 | 0.438692 | 0.867559 |
| E230013J01RIK | 0.000284 | 0.438692 | 0.815864 |
| COPE | 0.000307 | 0.439926 | 1.149761 |
| SSXB3 | 0.00034 | 0.439926 | 0.901022 |
| 2500002L14RIK | 0.000384 | 0.439926 | 1.547207 |
| BCL2A1D | 0.000445 | 0.439926 | 1.045964 |
| LOC385087 | 0.000462 | 0.439926 | 1.129538 |
| SND1 | 0.000468 | 0.439926 | 0.689202 |
| LOC380765 | 0.000483 | 0.439926 | 1.059781 |
| SCL0001065.1_54 | 0.000495 | 0.439926 | 0.904191 |
| 1810044B20RIK | 0.00051 | 0.439926 | 1.043165 |
| VRK1 | 0.000518 | 0.439926 | 0.770162 |
| LARS | 0.000547 | 0.439926 | 0.449326 |
| KLHL32 | 0.000568 | 0.439926 | 0.965267 |
| SLTM | 0.00057 | 0.439926 | 0.592738 |
| TRB | 0.000572 | 0.439926 | 0.746751 |
| CLEC2G | 0.000579 | 0.439926 | 0.877741 |
| WDR7 | 0.000589 | 0.439926 | 1.644976 |
| OLFR491 | 0.000603 | 0.439926 | 1.20815 |
| 2310016C16RIK | 0.000608 | 0.439926 | 1.627247 |
| 8030402P03RIK | 0.000615 | 0.439926 | 1.434717 |
| EG630499 | 0.00063 | 0.439926 | 1.545064 |
| SLC22A1 | 0.000646 | 0.439926 | 1.112959 |
| LOC100042970 | 0.000655 | 0.439926 | 0.428787 |
| ITPR3 | 0.000661 | 0.439926 | 1.767324 |
| 1110002D22RIK | 0.000667 | 0.439926 | 0.571173 |
| OLFR174 | 0.000674 | 0.439926 | 1.098042 |
| A430005L14RIK | 0.00069 | 0.439926 | 0.610375 |
| L2HGDH | 0.000694 | 0.439926 | 0.470565 |
| FRMPD4 | 0.000705 | 0.439926 | 0.809872 |
| OLFR984 | 0.000715 | 0.439926 | 0.955746 |
| ARD1A | 0.000725 | 0.439926 | 0.416099 |
| AGPAT1 | 0.000742 | 0.439926 | 1.59656 |
| ADIPOR1 | 0.000745 | 0.439926 | 1.200803 |
| RNF26 | 0.000756 | 0.439926 | 0.555169 |
| LOC670044 | 0.000776 | 0.441371 | 1.415423 |
| 1700027F06RIK | 0.000787 | 0.441371 | 0.916093 |
| ANKRD50 | 0.000854 | 0.442028 | 1.780563 |
| C330023J09RIK | 0.000902 | 0.442028 | 1.228814 |
| LOC381129 | 0.000902 | 0.442028 | 1.096673 |
| 2410015N17RIK | 0.000924 | 0.442028 | 0.3822 |
| DAG1 | 0.000933 | 0.442028 | 1.756861 |
| RPPH1 | 0.000933 | 0.442028 | 1.087941 |
| RPL14 | 0.000939 | 0.442028 | 0.563258 |
| FASL | 0.000982 | 0.442028 | 0.962417 |
| MYL6 | 0.000992 | 0.442028 | 2.142557 |
| BC003885 | 0.000997 | 0.442028 | 0.732754 |
| ZFH4 | 0.001 | 0.442028 | 1.133591 |
| CES3 | 0.001044 | 0.442028 | 0.832641 |
| L7RN6 | 0.001048 | 0.442028 | 0.738157 |
| HSPA1A | 0.001048 | 0.442028 | 0.918637 |
| LOC100046578 | 0.001057 | 0.442028 | 1.092904 |
| STRA13 | 0.001068 | 0.442028 | 0.680752 |
| PREI3 | 0.001074 | 0.442028 | 0.727238 |
| OLFR157 | 0.001074 | 0.442028 | 0.982503 |
| MRPL13 | 0.001086 | 0.442028 | 0.623013 |
| GPATCH4 | 0.001102 | 0.442028 | 0.588793 |
| LOC381140 | 0.001111 | 0.442028 | 2.620423 |
| LOC100047009 | 0.001176 | 0.442028 | 0.694349 |
| NDST2 | 0.001198 | 0.442028 | 1.532443 |
| MESDC2 | 0.001202 | 0.442028 | 1.138184 |
| LOC234582 | 0.001208 | 0.442028 | 1.36358 |
| GRINA | 0.001236 | 0.442028 | 1.441929 |
| AU040829 | 0.001254 | 0.442028 | 1.542638 |
| FCGR4 | 0.001258 | 0.442028 | 2.212176 |
| 5830418G11RIK | 0.001305 | 0.442028 | 0.812403 |
| PWP1 | 0.001336 | 0.442028 | 0.552584 |
| ZFP318 | 0.001337 | 0.442028 | 0.915184 |
| LOC671064 | 0.001342 | 0.442028 | 1.086433 |
| OLFR470 | 0.001372 | 0.442028 | 1.123811 |
| THADA | 0.001432 | 0.442028 | 1.111545 |
| LOC385319 | 0.001457 | 0.442028 | 1.062945 |
| A430072D10RIK | 0.001475 | 0.442028 | 1.041431 |
| CPSF1 | 0.001485 | 0.442028 | 0.744288 |
| LMCD1 | 0.001487 | 0.442028 | 3.291702 |
| LOC207685 | 0.001487 | 0.442028 | 0.815675 |
| 1110017D15RIK | 0.001503 | 0.442028 | 0.843738 |
| TAPBP | 0.001505 | 0.442028 | 1.424807 |
| RNF8 | 0.001507 | 0.442028 | 0.926245 |
| GPX8 | 0.00155 | 0.442028 | 1.536875 |
| SMC4 | 0.001558 | 0.442028 | 0.600582 |
| ESR1 | 0.001562 | 0.442028 | 1.059292 |
| TUBGCP5 | 0.001565 | 0.442028 | 0.904944 |
| LOC626259 | 0.001573 | 0.442028 | 0.862781 |
| 8430403M15RIK | 0.001585 | 0.442028 | 0.840858 |
| ATP6V1E1 | 0.001613 | 0.442028 | 1.478021 |
| TEX10 | 0.001618 | 0.442028 | 0.697646 |
| ATP5B | 0.001619 | 0.442028 | 0.887612 |
| ATXN1L | 0.00162 | 0.442028 | 1.433955 |
| B230373P09RIK | 0.001645 | 0.442028 | 0.950615 |
| CD48 | 0.001646 | 0.442028 | 1.042514 |
| STAT3 | 0.001649 | 0.442028 | 1.542924 |
| NFKB1 | 0.001673 | 0.442028 | 1.376623 |
| PRMT7 | 0.001679 | 0.442028 | 0.41823 |
| SCYE1 | 0.001693 | 0.442028 | 0.675331 |
| ENSMUSG00000043661 | 0.00172 | 0.442028 | 0.930256 |
| NGP | 0.001727 | 0.442028 | 0.901042 |
| MMP14 | 0.001744 | 0.442028 | 0.80057 |
| USP19 | 0.00176 | 0.442028 | 1.335302 |
| A930009E05RIK | 0.00177 | 0.442028 | 0.680028 |
| LOC382732 | 0.001773 | 0.442028 | 1.143429 |
| E330022G21RIK | 0.001779 | 0.442028 | 0.8511 |
| PRL7A2 | 0.001782 | 0.442028 | 1.065773 |
| PSMD6 | 0.001786 | 0.442028 | 0.750574 |
| 2010313D22RIK | 0.0018 | 0.442028 | 1.586778 |
| DBN1 | 0.001801 | 0.442028 | 1.767488 |
| 1110033K02RIK | 0.001804 | 0.442028 | 1.585751 |
| LOC207853 | 0.001805 | 0.442028 | 1.161267 |
| 9629514_7_RC | 0.001828 | 0.444008 | 0.89331 |
| AKR1C20 | 0.001846 | 0.445038 | 0.939197 |
| OASL2 | 0.001863 | 0.445643 | 1.910389 |
| GPN3 | 0.001922 | 0.453244 | 0.755673 |
| RNF166 | 0.001936 | 0.453244 | 1.471479 |
| ALG11 | 0.001953 | 0.453244 | 1.184518 |
| 1700095J19RIK | 0.001958 | 0.453244 | 1.21397 |
| CDKL2 | 0.001968 | 0.453244 | 0.870571 |
| UBE1L | 0.002024 | 0.458694 | 3.029963 |
| LOC100042773 | 0.002038 | 0.458694 | 0.82283 |
| PYCR1 | 0.002042 | 0.458694 | 0.392283 |
| D430030F08RIK | 0.002102 | 0.458694 | 1.176608 |
| LOC100046035 | 0.002114 | 0.458694 | 0.812891 |
| SLC27A2 | 0.002126 | 0.458694 | 0.917407 |
| TGFBR1 | 0.002139 | 0.458694 | 1.693177 |
| PIK3R5 | 0.00214 | 0.458694 | 0.90754 |
| LOC100046770 | 0.002143 | 0.458694 | 0.752623 |
| AMDHD2 | 0.002156 | 0.458694 | 1.95265 |
| MKIAA0250 | 0.002161 | 0.458694 | 0.903648 |
| LOC666185 | 0.002186 | 0.458694 | 0.929504 |
| FLRT3 | 0.002225 | 0.458694 | 0.869164 |
| TGIF2 | 0.002232 | 0.458694 | 0.881154 |
| AI836003 | 0.002261 | 0.458694 | 1.092626 |
| F830008E12RIK | 0.002283 | 0.458694 | 1.092096 |
| AA409316 | 0.002284 | 0.458694 | 1.369611 |
| A4GNT | 0.002287 | 0.458694 | 0.902876 |
| OLFR715 | 0.00229 | 0.458694 | 1.106293 |
| PSTK | 0.002298 | 0.458694 | 0.635472 |
| 5031439A09RIK | 0.002304 | 0.458694 | 1.406946 |
| COLQ | 0.002327 | 0.460176 | 1.132151 |
| PABPC4 | 0.002376 | 0.467034 | 0.503478 |
| LOC383579 | 0.002403 | 0.468935 | 0.820761 |
| USP1 | 0.002444 | 0.468935 | 0.411349 |
| AGPAT5 | 0.002447 | 0.468935 | 1.090079 |
| FRAT2 | 0.002453 | 0.468935 | 0.57087 |
| FIBP | 0.002496 | 0.468935 | 1.305256 |
| 1700024P12RIK | 0.002518 | 0.468935 | 0.873633 |
| LOC385877 | 0.002537 | 0.468935 | 1.455351 |
| MGP | 0.002549 | 0.468935 | 2.186566 |
| 6720469N11RIK | 0.002557 | 0.468935 | 1.822603 |
| 2610207P08RIK | 0.002564 | 0.468935 | 0.626665 |
| IAH1 | 0.002569 | 0.468935 | 1.797136 |
| BTBD10 | 0.002575 | 0.468935 | 0.806884 |
| LOC100044103 | 0.002584 | 0.468935 | 0.801014 |
| PPID | 0.002666 | 0.470169 | 0.400072 |
| GCLM | 0.002675 | 0.470169 | 0.786181 |
| NAGK | 0.002694 | 0.470169 | 1.918086 |
| LOC384418 | 0.002704 | 0.470169 | 1.103663 |
| PLEKHG2 | 0.002745 | 0.470169 | 2.287016 |
| 2400001E08RIK | 0.002753 | 0.470169 | 1.563703 |
| 1700066J24RIK | 0.002768 | 0.470169 | 0.914 |
| 5730543M03RIK | 0.00277 | 0.470169 | 0.754591 |
| VAMP8 | 0.002773 | 0.470169 | 1.807297 |
| 2310039E09RIK | 0.002832 | 0.470169 | 1.547672 |
| OLFR419 | 0.002833 | 0.470169 | 0.8895 |
| 5430405G05RIK | 0.002856 | 0.470169 | 1.039747 |
| BC061039 | 0.002921 | 0.470169 | 0.900917 |
| WDR43 | 0.002959 | 0.470169 | 0.401183 |
| TMEM174 | 0.002972 | 0.470169 | 1.070017 |
| FAM168B | 0.003002 | 0.470169 | 1.379266 |
| LOC240669 | 0.003051 | 0.470169 | 1.128338 |
| AVIL | 0.003078 | 0.470169 | 1.120544 |
| TRAP1A | 0.0031 | 0.470169 | 1.109698 |
| LOC674960 | 0.003107 | 0.470169 | 0.795592 |
| E430039I23RIK | 0.003119 | 0.470169 | 0.617567 |
| E330031M19RIK | 0.003121 | 0.470169 | 0.835957 |
| LOC384054 | 0.003133 | 0.470169 | 0.583 |
| APC2 | 0.003136 | 0.470169 | 0.914994 |
| HCN3 | 0.003146 | 0.470169 | 0.891043 |
| AASDHPPT | 0.00315 | 0.470169 | 0.850765 |
| CCNA1 | 0.003153 | 0.470169 | 0.837136 |
| V1RC4 | 0.003159 | 0.470169 | 1.114065 |
| SOX17 | 0.003161 | 0.470169 | 1.124097 |
| LOC100047093 | 0.00318 | 0.470169 | 1.229154 |
| LOC386123 | 0.003193 | 0.470169 | 1.231628 |
| EDG5 | 0.003223 | 0.470169 | 1.667862 |
| 4833425P12RIK | 0.003226 | 0.470169 | 0.78433 |
| 6230416J20RIK | 0.003296 | 0.470169 | 0.541763 |
| LOC384508 | 0.003299 | 0.470169 | 0.864737 |
| ELAC2 | 0.003304 | 0.470169 | 0.636059 |
| ZRANB2 | 0.003314 | 0.470169 | 0.70466 |
| EIF3S8 | 0.003326 | 0.470169 | 0.513464 |
| GFM | 0.003373 | 0.470169 | 0.772336 |
| 9330176C04RIK | 0.003385 | 0.470169 | 0.825935 |
| LOC380927 | 0.0034 | 0.470169 | 0.546806 |
| 4932417H02RIK | 0.003411 | 0.470169 | 1.186792 |
| ZFP316 | 0.003422 | 0.470169 | 1.435944 |
| OLFR704 | 0.003466 | 0.470169 | 1.14907 |
| 1500011J06RIK | 0.00347 | 0.470169 | 1.120648 |
| 2700023E23RIK | 0.003475 | 0.470169 | 0.733736 |
| C77604 | 0.003517 | 0.470169 | 1.52323 |
| ST6GAL1 | 0.003546 | 0.470169 | 1.538296 |
| LOC245475 | 0.003555 | 0.470169 | 1.203387 |
| F630041D06RIK | 0.003559 | 0.470169 | 0.821937 |
| RPS11 | 0.003559 | 0.470169 | 0.883724 |
| MTBP | 0.00357 | 0.470169 | 0.467013 |
| OLFR73 | 0.003669 | 0.470169 | 1.181675 |
| LOC382128 | 0.003678 | 0.470169 | 0.507883 |
| NAP1L1 | 0.003688 | 0.470169 | 0.536134 |
| FGFRL1 | 0.003717 | 0.470169 | 1.908448 |
| CDC7 | 0.003725 | 0.470169 | 0.425678 |
| TMPRSS11B | 0.003732 | 0.470169 | 0.882724 |
| GNL3 | 0.003734 | 0.470169 | 0.406032 |
| UCHL3 | 0.003739 | 0.470169 | 0.727894 |
| B130006D01RIK | 0.003739 | 0.470169 | 1.083501 |
| NFXL1 | 0.003783 | 0.470169 | 0.804464 |
| C80913 | 0.003785 | 0.470169 | 0.626955 |
| CPB2 | 0.003786 | 0.470169 | 1.095154 |
| FSHPRH1 | 0.003791 | 0.470169 | 0.520751 |
| 1810063B05RIK | 0.003791 | 0.470169 | 0.880707 |
| WFIKKN2 | 0.003793 | 0.470169 | 0.937484 |
| LOC675813 | 0.003794 | 0.470169 | 1.12714 |
| IGSF3 | 0.003806 | 0.470169 | 1.53503 |
| PUS10 | 0.003811 | 0.470169 | 0.674629 |
| NCAPD3 | 0.003822 | 0.470169 | 0.325847 |
| LOC381177 | 0.003826 | 0.470169 | 1.125188 |
| PDE4DIP | 0.003869 | 0.470169 | 0.926824 |
| A630059F13RIK | 0.003883 | 0.470169 | 0.783316 |
| DHX32 | 0.003887 | 0.470169 | 0.665879 |
| RETSAT | 0.003891 | 0.470169 | 0.36736 |
| STXBP3A | 0.003921 | 0.470169 | 0.923873 |
| 9530058B02RIK | 0.003929 | 0.470169 | 0.284789 |
| 1700029G01RIK | 0.003939 | 0.470169 | 1.393165 |
| LOC385758 | 0.003954 | 0.470169 | 0.931052 |
| VMN2R42 | 0.003956 | 0.470169 | 1.098575 |
| CARHSP1 | 0.003983 | 0.470169 | 1.277982 |
| LOC382255 | 0.003987 | 0.470169 | 0.888124 |
| LOC383029 | 0.004017 | 0.470169 | 1.130295 |
| A630084D02RIK | 0.004026 | 0.470169 | 0.782068 |
| XRCC1 | 0.004037 | 0.470169 | 0.849116 |
| TCERG1 | 0.004082 | 0.470169 | 1.070264 |
| 6530406A20RIK | 0.004095 | 0.470169 | 1.292084 |
| D17WSU92E | 0.004102 | 0.470169 | 1.246601 |
| ATP6V1D | 0.004115 | 0.470169 | 1.280169 |
| HNF1B | 0.004134 | 0.470169 | 1.138999 |
| LOC671641 | 0.00415 | 0.470169 | 0.692635 |
| A130094L10RIK | 0.004157 | 0.470169 | 1.135714 |
| REEP6 | 0.00416 | 0.470169 | 0.254135 |
| SUDS3 | 0.004161 | 0.470169 | 1.139183 |
| 2010003O18RIK | 0.004164 | 0.470169 | 0.643242 |
| OLFR961 | 0.004208 | 0.470169 | 0.832045 |
| LOC380833 | 0.00421 | 0.470169 | 0.936013 |
| DNAJA3 | 0.004213 | 0.470169 | 0.721298 |
| EG546250 | 0.004234 | 0.470169 | 0.924193 |
| ULK4 | 0.004292 | 0.470169 | 0.802496 |
| MSR1 | 0.004332 | 0.470169 | 0.94261 |
| 1700071K01RIK | 0.004341 | 0.470169 | 0.704253 |
| 6530401P13 | 0.004349 | 0.470169 | 0.876909 |
| LOC386225 | 0.004355 | 0.470169 | 0.900647 |
| C130027E04RIK | 0.004355 | 0.470169 | 1.246457 |
| SUCLG2 | 0.004379 | 0.470169 | 0.45986 |
| PUSL1 | 0.00439 | 0.470169 | 0.713771 |
| 5930403H17RIK | 0.004429 | 0.470169 | 0.818715 |
| ARHGEF5 | 0.004431 | 0.470169 | 1.624767 |
| ENSMUSG00000058057 | 0.004443 | 0.470169 | 0.931095 |
| DRD4 | 0.004448 | 0.470169 | 1.080601 |
| GM1631 | 0.004486 | 0.470169 | 1.181211 |
| 6530415H11RIK | 0.004517 | 0.470169 | 0.911133 |
| HGS | 0.00452 | 0.470169 | 1.47973 |
| HIST1H2AC | 0.004531 | 0.470169 | 1.249543 |
| LOC207879 | 0.004537 | 0.470169 | 1.139131 |
| D14ERTD500E | 0.004547 | 0.470169 | 0.845475 |
| ANAPC13 | 0.004577 | 0.470169 | 1.105475 |
| LOC236069 | 0.004603 | 0.470169 | 1.10614 |
| 9630055N22RIK | 0.004605 | 0.470169 | 0.633156 |
| A130067E09RIK | 0.004609 | 0.470169 | 0.77414 |
| SMU1 | 0.004642 | 0.470169 | 0.745252 |
| SERF2 | 0.004688 | 0.470169 | 1.489333 |
| SERBP1 | 0.004697 | 0.470169 | 0.499077 |
| WDR76 | 0.004706 | 0.470169 | 0.481453 |
| FAM102A | 0.004755 | 0.470169 | 3.006879 |
| E130303B06RIK | 0.004758 | 0.470169 | 0.583552 |
| COG4 | 0.004771 | 0.470169 | 1.216722 |
| MSH6 | 0.004772 | 0.470169 | 0.593368 |
| F630043A04RIK | 0.004777 | 0.470169 | 0.36527 |
| ACY1 | 0.004778 | 0.470169 | 0.454452 |
| PDHB | 0.0048 | 0.470169 | 0.611447 |
| EG226604 | 0.004836 | 0.470169 | 0.860392 |
| LRRC19 | 0.004841 | 0.470169 | 0.934068 |
| TMEM209 | 0.00488 | 0.470169 | 0.72499 |
| 4432406L21RIK | 0.00492 | 0.470169 | 1.086986 |
| MJ-8000-190_4699 | 0.004945 | 0.470169 | 0.918382 |
| PTGES2 | 0.004947 | 0.470169 | 0.779003 |
| CAR12 | 0.004948 | 0.470169 | 0.212088 |
| DLG3 | 0.00497 | 0.470169 | 1.137658 |
| SH3GL1 | 0.004987 | 0.470169 | 1.551539 |
| GOLGA7B | 0.005011 | 0.470169 | 1.135583 |
| ARHGEF18 | 0.005019 | 0.470169 | 1.577966 |
| TXNDC17 | 0.005028 | 0.470169 | 1.120648 |
| POLR3G | 0.005036 | 0.470169 | 0.833892 |
| RAB3IP | 0.005069 | 0.470169 | 0.858506 |
| KLRA15 | 0.005075 | 0.470169 | 0.905906 |
| PPIL3 | 0.005118 | 0.470169 | 0.869063 |
| PRDX5 | 0.005119 | 0.470169 | 1.827241 |
| LOC268602 | 0.005129 | 0.470169 | 1.146312 |
| MTMR13 | 0.005166 | 0.470169 | 1.074327 |
| LOC385461 | 0.005186 | 0.470169 | 0.368831 |
| ACPT | 0.005189 | 0.470169 | 1.109313 |
| OSBPL3 | 0.005195 | 0.470169 | 1.517539 |
| MTDNA_ATP8 | 0.005211 | 0.470169 | 1.683737 |
| FBN1 | 0.005212 | 0.470169 | 1.662783 |
| 4930471I01RIK | 0.005217 | 0.470169 | 0.780263 |
| TOP2A | 0.005221 | 0.470169 | 0.499238 |
| GTF2H3 | 0.005252 | 0.470169 | 0.706633 |
| ILVBL | 0.005265 | 0.470169 | 1.4466 |
| OLFR513 | 0.005265 | 0.470169 | 1.216526 |
| SARS | 0.005266 | 0.470169 | 0.514972 |
| NLRP9C | 0.005272 | 0.470169 | 0.927273 |
| 5730470C09RIK | 0.005288 | 0.470169 | 0.855615 |
| DNAJC2 | 0.005296 | 0.470169 | 0.49339 |
| TUBE1 | 0.005315 | 0.470169 | 0.482489 |
| KRTAP8-2 | 0.005316 | 0.470169 | 1.116023 |
| OLFR91 | 0.005363 | 0.470169 | 0.868622 |
| NOB1 | 0.005376 | 0.470169 | 0.471904 |
| OLFR1213 | 0.005379 | 0.470169 | 0.917852 |
| OLFR801 | 0.005413 | 0.470169 | 0.877639 |
| LOC384359 | 0.00544 | 0.470169 | 0.967522 |
| 9630031D17RIK | 0.005447 | 0.470169 | 0.727171 |
| KBTBD10 | 0.005468 | 0.470169 | 1.111494 |
| 2310047M10RIK | 0.005527 | 0.470169 | 1.602843 |
| CDCP1 | 0.005528 | 0.470169 | 0.930536 |
| 6430553K24RIK | 0.00555 | 0.470169 | 0.898589 |
| CYP7B1 | 0.005553 | 0.470169 | 0.831007 |
| ATG4B | 0.005558 | 0.470169 | 1.27742 |
| TEAD2 | 0.005559 | 0.470169 | 1.381594 |
| COMMD9 | 0.005566 | 0.470169 | 1.367335 |
| TSPAN4 | 0.0056 | 0.470169 | 1.179329 |
| DDX11 | 0.005633 | 0.470169 | 0.514093 |
| HTRA2 | 0.005669 | 0.470169 | 0.609585 |
| BZW2 | 0.005679 | 0.470169 | 0.462759 |
| MTDNA_ND5 | 0.005681 | 0.470169 | 2.344965 |
| ABCA8B | 0.005687 | 0.470169 | 0.876768 |
| B230377N03RIK | 0.005708 | 0.470169 | 0.830528 |
| DSN1 | 0.005714 | 0.470169 | 0.591903 |
| A730049H05RIK | 0.005716 | 0.470169 | 1.120622 |
| MASP1 | 0.005727 | 0.470169 | 1.144116 |
| COX6A2 | 0.005744 | 0.470169 | 0.525162 |
| SCL0001434.1_18 | 0.005773 | 0.470169 | 0.907603 |
| RBMS1 | 0.00578 | 0.470169 | 1.574615 |
| A730010O16RIK | 0.005789 | 0.470169 | 0.879385 |
| LOC100041613 | 0.005794 | 0.470169 | 0.670232 |
| FAM159B | 0.005805 | 0.470169 | 1.107955 |
| MRPS17 | 0.005806 | 0.470169 | 0.802552 |
| 2610315N17RIK | 0.005847 | 0.470169 | 0.787363 |
| LOC235300 | 0.005853 | 0.470169 | 0.833527 |
| 6430537F04 | 0.005854 | 0.470169 | 0.918807 |
| MST1R | 0.005855 | 0.470169 | 0.928152 |
| ARL6IP1 | 0.005862 | 0.470169 | 0.679871 |
| UBA1 | 0.005867 | 0.470169 | 0.545392 |
| PHACTR4 | 0.005871 | 0.470169 | 1.186737 |
| A430060F13RIK | 0.005886 | 0.470169 | 1.09139 |
| NUDCD2 | 0.005894 | 0.470169 | 0.563961 |
| TCRG | 0.005901 | 0.470169 | 0.825286 |
| ABHD11 | 0.005915 | 0.470169 | 0.576703 |
| AMBRA1 | 0.005928 | 0.470169 | 1.3644 |
| 3321401G04RIK | 0.005937 | 0.470169 | 1.554696 |
| APBB1 | 0.005966 | 0.470169 | 1.451824 |
| NAT2 | 0.005979 | 0.470169 | 1.351817 |
| LOC100045044 | 0.005984 | 0.470169 | 0.835185 |
| WDR67 | 0.005987 | 0.470169 | 0.525975 |
| TAX1BP1 | 0.006012 | 0.470169 | 0.727154 |
| SSTR2 | 0.006026 | 0.470169 | 1.128808 |
| MRPS24 | 0.006027 | 0.470169 | 0.732212 |
| TCOF1 | 0.006044 | 0.470169 | 0.466312 |
| EHD2 | 0.006055 | 0.470169 | 1.654123 |
| LOC100047184 | 0.006071 | 0.470169 | 0.57821 |
| PHB2 | 0.006077 | 0.470169 | 0.587503 |
| EPB7.2 | 0.006088 | 0.470169 | 0.62781 |
| ATAD1 | 0.006107 | 0.470169 | 0.721331 |
| 4632407F12RIK | 0.00611 | 0.470169 | 0.713606 |
| LAMB2 | 0.006137 | 0.470169 | 1.808174 |
| 4930534B04RIK | 0.006138 | 0.470169 | 0.666157 |
| TRIP6 | 0.006155 | 0.470169 | 1.410624 |
| SLC7A13 | 0.006171 | 0.470169 | 0.896018 |
| SLC25A3 | 0.006176 | 0.470169 | 0.566573 |
| SCL0003131.1_3 | 0.006179 | 0.470169 | 1.109775 |
| H1FX | 0.006192 | 0.470169 | 0.615018 |
| COX6B1 | 0.006214 | 0.470169 | 1.206922 |
| 2310003F16RIK | 0.006247 | 0.470169 | 0.771818 |
| C1GALT1 | 0.006248 | 0.470169 | 1.378756 |
| B230114H05RIK | 0.006252 | 0.470169 | 1.287584 |
| 4732435K05RIK | 0.006262 | 0.470169 | 0.594356 |
| PCSK5 | 0.006272 | 0.470169 | 0.979217 |
| MAPK1 | 0.00633 | 0.470169 | 0.783932 |
| ARHGAP19 | 0.006352 | 0.470169 | 0.578518 |
| DUS2L | 0.006371 | 0.470169 | 0.896329 |
| ATN1 | 0.006386 | 0.470169 | 1.898465 |
| ULK1 | 0.0064 | 0.470169 | 1.384086 |
| 9830002L24RIK | 0.006411 | 0.470169 | 0.516056 |
| EG625349 | 0.00645 | 0.470169 | 0.83557 |
| AQP6 | 0.006465 | 0.470169 | 0.878938 |
| C030011O14RIK | 0.006472 | 0.470169 | 0.666187 |
| CD63 | 0.006485 | 0.470169 | 1.321033 |
| SASS6 | 0.006507 | 0.470169 | 0.461318 |
| ANXA8 | 0.006509 | 0.470169 | 1.462395 |
| AGXT2L2 | 0.006512 | 0.470169 | 0.481631 |
| FAT2 | 0.006514 | 0.470169 | 1.099032 |
| RNF215 | 0.006517 | 0.470169 | 1.27515 |
| GM216 | 0.006547 | 0.470169 | 1.045505 |
| 4632407J06RIK | 0.006566 | 0.470169 | 0.892547 |
| NR1H2 | 0.00657 | 0.470169 | 1.812105 |
| LOC380719 | 0.006613 | 0.470169 | 1.06809 |
| 3110082I17RIK | 0.006618 | 0.470169 | 0.632469 |
| PA2G4 | 0.006621 | 0.470169 | 0.405395 |
| LOC380706 | 0.006632 | 0.470169 | 2.862084 |
| PRIM1 | 0.006678 | 0.470169 | 0.504095 |
| EG545370 | 0.006697 | 0.470169 | 1.131994 |
| CNN1 | 0.006707 | 0.470169 | 0.847607 |
| SCL00238693.1_37 | 0.006719 | 0.470169 | 1.540003 |
| MLYCD | 0.006767 | 0.470169 | 1.113962 |
| E030024N20RIK | 0.006768 | 0.470169 | 0.875209 |
| FOLR1 | 0.006773 | 0.470169 | 1.066808 |
| ZFP513 | 0.006778 | 0.470169 | 1.489368 |
| TTC9C | 0.006799 | 0.470169 | 0.559328 |
| CTLA4 | 0.006812 | 0.470169 | 1.147478 |
| LOC268828 | 0.006823 | 0.470169 | 1.179302 |
| TSC2 | 0.006837 | 0.470169 | 1.465101 |
| SETD6 | 0.006846 | 0.470169 | 0.522873 |
| MADD | 0.006849 | 0.470169 | 1.171481 |
| F13B | 0.00686 | 0.470169 | 0.935991 |
| EG665685 | 0.006885 | 0.470169 | 0.786799 |
| RNPS1 | 0.006893 | 0.470169 | 0.658399 |
| CYP2C55 | 0.006921 | 0.470169 | 0.57637 |
| TOM1 | 0.006932 | 0.470169 | 1.318319 |
| HSPE1 | 0.006964 | 0.470169 | 0.511699 |
| OLA1 | 0.007017 | 0.470169 | 0.565618 |
| TWISTNB | 0.007043 | 0.470169 | 0.66762 |
| RIN3 | 0.007062 | 0.470169 | 1.440198 |
| 2310045K21RIK | 0.007078 | 0.470169 | 0.439642 |
| E130306L18RIK | 0.007106 | 0.470169 | 0.756826 |
| CC2D1A | 0.007139 | 0.470169 | 1.523405 |
| SLC1A5 | 0.007188 | 0.470169 | 0.845299 |
| RELA | 0.007215 | 0.470169 | 1.525906 |
| MRPL28 | 0.007228 | 0.470169 | 0.583309 |
| 2010005E20RIK | 0.007228 | 0.470169 | 0.744873 |
| SLC16A12 | 0.007245 | 0.470169 | 1.081625 |
| SIPA1L1 | 0.007254 | 0.470169 | 1.55617 |
| TXNRD2 | 0.007258 | 0.470169 | 0.597841 |
| RNU65 | 0.007324 | 0.470169 | 0.459064 |
| A730014O07RIK | 0.007333 | 0.470169 | 1.128156 |
| CCDC80 | 0.007341 | 0.470169 | 1.516067 |
| DTL | 0.007344 | 0.470169 | 0.709611 |
| PQLC3 | 0.007348 | 0.470169 | 0.712914 |
| PRKACA | 0.007351 | 0.470169 | 1.32516 |
| KRT83 | 0.007356 | 0.470169 | 0.863619 |
| 9330155C01RIK | 0.007359 | 0.470169 | 1.398875 |
| OLFR24 | 0.007393 | 0.470169 | 0.95557 |
| IRGQ | 0.007403 | 0.470169 | 1.778713 |
| LOC386126 | 0.007429 | 0.470169 | 0.921039 |
| WDR5 | 0.007437 | 0.470169 | 0.6232 |
| PRKAR1B | 0.007462 | 0.470169 | 0.920039 |
| CACNA1A | 0.007466 | 0.470169 | 1.094496 |
| SELK | 0.007492 | 0.470169 | 1.474815 |
| NCAPG2 | 0.007509 | 0.470169 | 0.542051 |
| 4930428E23RIK | 0.007522 | 0.470169 | 1.044346 |
| CYB5 | 0.007528 | 0.470169 | 1.505247 |
| HDGF | 0.007531 | 0.470169 | 0.710333 |
| ORC4L | 0.007539 | 0.470169 | 0.853108 |
| ZFP775 | 0.007552 | 0.470169 | 1.23308 |
| IFI47 | 0.007552 | 0.470169 | 2.292042 |
| CENPP | 0.007559 | 0.470169 | 0.267282 |
| TPP1 | 0.007559 | 0.470169 | 2.037783 |
| E130016E03RIK | 0.007565 | 0.470169 | 0.216039 |
| C130046N05RIK | 0.007565 | 0.470169 | 0.883703 |
| DNAJB6 | 0.007566 | 0.470169 | 1.53826 |
| LOC331752 | 0.007629 | 0.470169 | 1.144037 |
| 5830418K08RIK | 0.007632 | 0.470169 | 1.12254 |
| LOC333744 | 0.007674 | 0.470169 | 0.724354 |
| OLFR798 | 0.007675 | 0.470169 | 1.057458 |
| QRICH1 | 0.007685 | 0.470169 | 1.230746 |
| PELI3 | 0.007698 | 0.470169 | 1.207341 |
| TARS2 | 0.007699 | 0.470169 | 0.46399 |
| SSBP4 | 0.007717 | 0.470169 | 0.597081 |
| 2210408O09RIK | 0.00772 | 0.470169 | 1.236561 |
| AK5 | 0.007726 | 0.470169 | 1.675161 |
| GSPT1 | 0.007736 | 0.470169 | 0.70907 |
| ZFP438 | 0.00774 | 0.470169 | 1.214335 |
| 4930506M07RIK | 0.007742 | 0.470169 | 0.789239 |
| GTF3C6 | 0.007758 | 0.470169 | 0.860969 |
| STEAP3 | 0.007762 | 0.470169 | 1.173974 |
| LOC100047693 | 0.007793 | 0.470169 | 0.914296 |
| FRMD7 | 0.007795 | 0.470169 | 1.13553 |
| PPFIBP2 | 0.007801 | 0.470169 | 0.96393 |
| EG665509 | 0.007839 | 0.470169 | 2.006805 |
| SLC39A4 | 0.007909 | 0.470169 | 1.107085 |
| PAF1 | 0.007928 | 0.470169 | 1.207341 |
| 1700025O08RIK | 0.007963 | 0.470169 | 1.026713 |
| LOC623121 | 0.007999 | 0.470169 | 0.743068 |
| MGAT3 | 0.008007 | 0.470169 | 1.846466 |
| NRG1 | 0.008048 | 0.470169 | 1.774567 |
| PPP1R9B | 0.008049 | 0.470169 | 1.375574 |
| A230083G16RIK | 0.008053 | 0.470169 | 1.055286 |
| PRR13 | 0.008056 | 0.470169 | 1.745532 |
| MECR | 0.008088 | 0.470169 | 0.500312 |
| MYD88 | 0.008102 | 0.470169 | 1.459357 |
| 6330548G22RIK | 0.008104 | 0.470169 | 0.724706 |
| SLC35F2 | 0.008109 | 0.470169 | 0.414593 |
| GPR113 | 0.008113 | 0.470169 | 0.851887 |
| CASP6 | 0.008115 | 0.470169 | 1.7767 |
| A230055O06RIK | 0.008143 | 0.470169 | 1.097281 |
| S3-12 | 0.008164 | 0.470169 | 2.317764 |
| 1110057P08RIK | 0.008164 | 0.470169 | 0.895563 |
| RRM2B | 0.008165 | 0.470169 | 0.812759 |
| LOC100044215 | 0.008186 | 0.470169 | 1.134534 |
| NOLA3 | 0.008197 | 0.470169 | 0.542866 |
| B230322F03RIK | 0.008263 | 0.470169 | 0.897655 |
| FANCI | 0.008264 | 0.470169 | 0.669026 |
| PCDHGB6 | 0.008282 | 0.470169 | 1.288566 |
| SCL000179.1_19 | 0.008293 | 0.470169 | 0.564913 |
| MEA1 | 0.008308 | 0.470169 | 1.540466 |
| 9530081N05RIK | 0.008309 | 0.470169 | 1.919415 |
| GPHB5 | 0.008314 | 0.470169 | 1.24911 |
| 2310002B06RIK | 0.008318 | 0.470169 | 1.426982 |
| MAPKAPK2 | 0.008328 | 0.470169 | 1.347638 |
| LOC380665 | 0.008355 | 0.470169 | 0.397043 |
| TIEG | 0.008377 | 0.470169 | 0.822013 |
| BC024139 | 0.008388 | 0.470169 | 0.725309 |
| ZNHIT1 | 0.008391 | 0.470169 | 1.417354 |
| NSL1 | 0.008398 | 0.470169 | 0.760033 |
| PDLIM7 | 0.00841 | 0.470169 | 1.425367 |
| OPRM1 | 0.008413 | 0.470169 | 1.141265 |
| ZFP553 | 0.008423 | 0.470169 | 1.174381 |
| GUCY1A2 | 0.008424 | 0.470169 | 0.877355 |
| 9330151E04RIK | 0.008426 | 0.470169 | 1.099438 |
| POMC | 0.00845 | 0.470169 | 1.187669 |
| B630019K06RIK | 0.008453 | 0.470169 | 0.819586 |
| LOC675954 | 0.008461 | 0.470169 | 0.888391 |
| NDUFA9 | 0.00848 | 0.470169 | 0.726482 |
| 4833421E05RIK | 0.008492 | 0.470169 | 1.798383 |
| 6820406G21RIK | 0.008498 | 0.470169 | 0.719583 |
| SOX7 | 0.008565 | 0.470169 | 0.913557 |
| ZFP606 | 0.008572 | 0.470169 | 1.304804 |
| AI847670 | 0.008578 | 0.470169 | 0.217326 |
| LOC676420 | 0.008596 | 0.470169 | 1.901186 |
| YWHAE | 0.008615 | 0.470169 | 0.80532 |
| FBXW5 | 0.008639 | 0.470169 | 1.182085 |
| MANEA | 0.008643 | 0.470169 | 1.370878 |
| MICALL2 | 0.008647 | 0.470169 | 1.711151 |
| SLC32A1 | 0.008661 | 0.470169 | 0.888124 |
| OTTMUSG00000010673 | 0.008662 | 0.470169 | 0.765708 |
| 1110018M03RIK | 0.008666 | 0.470169 | 0.874744 |
| BRD2 | 0.008667 | 0.470169 | 1.271913 |
| TCIRG1 | 0.008671 | 0.470169 | 1.216666 |
| COLEC11 | 0.008686 | 0.470169 | 1.128208 |
| AKAP11 | 0.008723 | 0.470169 | 1.460235 |
| SORT1 | 0.008731 | 0.470169 | 1.445732 |
| 1700013D24RIK | 0.008731 | 0.470169 | 0.91567 |
| H6PD | 0.008764 | 0.470393 | 1.504446 |
| NUDT18 | 0.008779 | 0.470393 | 1.167617 |
| MSH2 | 0.008807 | 0.470393 | 0.620269 |
| KLHL15 | 0.00883 | 0.470393 | 0.623733 |
| LOC383199 | 0.008832 | 0.470393 | 1.055359 |
| NMD3 | 0.008833 | 0.470393 | 0.618395 |
| HIRIP3 | 0.008852 | 0.470393 | 0.268799 |
| MRPL21 | 0.00887 | 0.470393 | 1.110672 |
| RBP2 | 0.008892 | 0.470393 | 2.114036 |
| 2410127L17RIK | 0.008899 | 0.470393 | 0.580647 |
| PRRG3 | 0.00892 | 0.470393 | 0.559638 |
| PRPF38A | 0.00895 | 0.470393 | 0.756826 |
| C8B | 0.00897 | 0.470393 | 1.063485 |
| SCD2 | 0.008975 | 0.470393 | 1.558618 |
| GAA | 0.008977 | 0.470393 | 1.41683 |
| 2-Mar | 0.008979 | 0.470393 | 1.547243 |
| SAMD14 | 0.008996 | 0.470436 | 2.552657 |
| A730089K16RIK | 0.009051 | 0.472527 | 0.925818 |
| YDJC | 0.0091 | 0.474269 | 0.210754 |
| SERPINA9 | 0.009142 | 0.474754 | 2.102589 |
| LOC385314 | 0.009159 | 0.474754 | 1.12057 |
| TTLL6 | 0.009194 | 0.474754 | 1.13771 |
| 1700112C13RIK | 0.009199 | 0.474754 | 1.901889 |
| EXOD1 | 0.009244 | 0.474754 | 0.616398 |
| NUDCD1 | 0.009258 | 0.474754 | 0.840508 |
| ZFYVE1 | 0.009276 | 0.474754 | 1.493606 |
| VKORC1L1 | 0.009316 | 0.474754 | 0.856604 |
| NUP160 | 0.00932 | 0.474754 | 0.584929 |
| B430305P08RIK | 0.009324 | 0.474754 | 1.275504 |
| LOC622602 | 0.009358 | 0.474754 | 1.050711 |
| HOXA1 | 0.009391 | 0.474754 | 1.24203 |
| SERPINB6B | 0.009418 | 0.474754 | 2.052905 |
| SYAP1 | 0.009442 | 0.474754 | 1.733235 |
| ARL2 | 0.009457 | 0.474754 | 1.144963 |
| LOC100047090 | 0.009474 | 0.474754 | 0.793591 |
| HSD11B1 | 0.009474 | 0.474754 | 1.324977 |
| LOC630470 | 0.009479 | 0.474754 | 1.072046 |
| OLFR1248 | 0.009479 | 0.474754 | 0.934867 |
| C79407 | 0.009502 | 0.474754 | 0.453749 |
| HTR4 | 0.009505 | 0.474754 | 0.840236 |
| B930014F01RIK | 0.009553 | 0.474754 | 0.955658 |
| GAD1 | 0.009556 | 0.474754 | 0.845631 |
| WFDC1 | 0.009597 | 0.474754 | 0.788054 |
| LOC195806 | 0.009616 | 0.474754 | 0.862582 |
| 4933439J11RIK | 0.009626 | 0.474754 | 1.405743 |
| SPC25 | 0.009723 | 0.474754 | 0.270938 |
| OLFR507 | 0.009744 | 0.474754 | 0.857852 |
| 2400010C15RIK | 0.009771 | 0.474754 | 0.707434 |
| LOC623466 | 0.009819 | 0.474754 | 0.806269 |
| A930013F09RIK | 0.009824 | 0.474754 | 0.731079 |
| SET | 0.009827 | 0.474754 | 0.499434 |
| LOC100046003 | 0.009835 | 0.474754 | 1.45461 |
| OLFR92 | 0.009846 | 0.474754 | 0.965312 |
| TRAP100-PENDING | 0.009847 | 0.474754 | 0.895273 |
| 1700081D05RIK | 0.00986 | 0.474754 | 0.892691 |
| 2010309J24RIK | 0.009865 | 0.474754 | 0.756773 |
| AI115600 | 0.009908 | 0.474754 | 1.094445 |
| PLA2G4A | 0.009922 | 0.474754 | 1.151914 |
| CCDC120 | 0.009928 | 0.474754 | 1.253707 |
| PTTG1 | 0.009944 | 0.474754 | 0.73562 |
| B930045J24RIK | 0.009963 | 0.474754 | 1.633576 |
| OLFR1507 | 0.009983 | 0.474754 | 1.258001 |
| 4930506F14RIK | 0.009988 | 0.474754 | 0.86798 |
| BLMH | 0.009993 | 0.474754 | 0.605263 |
| FXYD4 | 0.009994 | 0.474754 | 1.147001 |
| GBP2 | 0.009999 | 0.474754 | 2.412005 |
| E330019C05RIK | 0.010003 | 0.474754 | 0.844401 |
| CAMK2D | 0.010007 | 0.474754 | 1.096698 |
| ADAMTS2 | 0.010015 | 0.474754 | 2.194613 |
| BC057079 | 0.010019 | 0.474754 | 0.439418 |
| TNMD | 0.010026 | 0.474754 | 1.236904 |
| PLXND1 | 0.010029 | 0.474754 | 1.539505 |
| 6330525I24RIK | 0.010054 | 0.474754 | 1.09609 |
| IYD | 0.010063 | 0.474754 | 0.849705 |
| OTOP1 | 0.010072 | 0.474754 | 0.800792 |
| PSIP1 | 0.010072 | 0.474754 | 0.664466 |
| RFX7 | 0.010074 | 0.474754 | 0.883662 |
| NDFIP2 | 0.010079 | 0.474754 | 1.240166 |
| CCDC77 | 0.010096 | 0.474754 | 0.40459 |
| A930004K21RIK | 0.010097 | 0.474754 | 0.874704 |
| EG244911 | 0.010171 | 0.474754 | 0.889562 |
| COPZ2 | 0.010214 | 0.474754 | 1.101676 |
| NPHS2 | 0.010216 | 0.474754 | 0.772657 |
| MAK3 | 0.010242 | 0.474754 | 1.065453 |
| HAGHL | 0.010244 | 0.474754 | 1.586228 |
| UBE3C | 0.010248 | 0.474754 | 0.822545 |
| SCL0002993.1_1671 | 0.010266 | 0.474754 | 1.096191 |
| OLFR937 | 0.010315 | 0.474754 | 1.085505 |
| YWHAH | 0.010365 | 0.474754 | 0.848194 |
| RIPK1 | 0.010383 | 0.474754 | 1.595085 |
| KDELR3 | 0.010383 | 0.474754 | 1.921678 |
| SAR1B | 0.010392 | 0.474754 | 1.192564 |
| OLFML2B | 0.0104 | 0.474754 | 2.328177 |
| ZIC4 | 0.010416 | 0.474754 | 1.593574 |
| LOC623312 | 0.010417 | 0.474754 | 0.852694 |
| MYO9B | 0.010433 | 0.474754 | 1.648134 |
| SOCS7 | 0.010443 | 0.474754 | 0.804446 |
| ACOT6 | 0.010479 | 0.474754 | 0.831065 |
| NMRAL1 | 0.01048 | 0.474754 | 0.5078 |
| ENDOD1 | 0.010492 | 0.474754 | 2.048877 |
| 5830468K18RIK | 0.010496 | 0.474754 | 0.442475 |
| RTP1 | 0.010499 | 0.474754 | 0.905237 |
| ZFP820 | 0.010532 | 0.474754 | 0.89265 |
| 4930417O22RIK | 0.010552 | 0.474754 | 0.739455 |
| ADCY3 | 0.010564 | 0.474754 | 0.895687 |
| RNF214 | 0.010584 | 0.474754 | 1.46693 |
| SNX33 | 0.010612 | 0.474754 | 2.036464 |
| LOC383713 | 0.010622 | 0.474754 | 0.85698 |
| PRSS23 | 0.010628 | 0.474754 | 1.936475 |
| MRPL20 | 0.010661 | 0.474754 | 1.068065 |
| SUMO2 | 0.010662 | 0.474754 | 0.883111 |
| PRL3A1 | 0.010662 | 0.474754 | 1.13223 |
| ING3 | 0.010674 | 0.474754 | 1.111982 |
| LOC100046274 | 0.010675 | 0.474754 | 0.847058 |
| URM1 | 0.0107 | 0.474754 | 1.192784 |
| 9130415E20RIK | 0.010704 | 0.474754 | 1.516768 |
| D630036G22RIK | 0.010713 | 0.474754 | 1.05042 |
| 6330403E01RIK | 0.010713 | 0.474754 | 0.781526 |
| NHP2L1 | 0.01075 | 0.474754 | 0.640987 |
| RABGEF1 | 0.010752 | 0.474754 | 1.491055 |
| DHRS7B | 0.010764 | 0.474754 | 0.875209 |
| B130017I01RIK | 0.010782 | 0.474754 | 1.46588 |
| 3010015K02RIK | 0.010811 | 0.474754 | 0.586323 |
| GAS2L3 | 0.010843 | 0.474754 | 0.551042 |
| 1810015A11RIK | 0.010853 | 0.474754 | 0.190994 |
| PCSK4 | 0.010854 | 0.474754 | 0.821766 |
| 4930405A10RIK | 0.010859 | 0.474754 | 0.84446 |
| LOC100047490 | 0.010869 | 0.474754 | 0.737646 |
| WDR73 | 0.01088 | 0.474754 | 0.647267 |
| LOC667769 | 0.010909 | 0.474754 | 1.124798 |
| MAN1C1 | 0.010923 | 0.474754 | 1.27018 |
| MTDNA_CYTB | 0.010957 | 0.474754 | 1.60029 |
| A730081H18RIK | 0.010958 | 0.474754 | 0.922679 |
| LOC669420 | 0.010983 | 0.474754 | 1.090054 |
| NLGN2 | 0.011004 | 0.474754 | 2.325811 |
| DCTN5 | 0.011017 | 0.474754 | 1.262544 |
| D630037D12RIK | 0.011033 | 0.474754 | 0.857634 |
| POU4F3 | 0.011042 | 0.474754 | 1.055408 |
| LOC671624 | 0.011067 | 0.474754 | 0.908988 |
| EXOSC8 | 0.011095 | 0.474754 | 0.322201 |
| PLSCR2 | 0.011107 | 0.474754 | 1.463071 |
| LOC623006 | 0.011115 | 0.474754 | 0.47233 |
| UBE2Q2 | 0.011132 | 0.474754 | 0.65916 |
| GINS4 | 0.011134 | 0.474754 | 0.806828 |
| BC050078 | 0.01114 | 0.474754 | 1.16608 |
| POP5 | 0.01115 | 0.474754 | 0.551347 |
| JMJD2A | 0.011169 | 0.474754 | 0.660684 |
| 1700028I16RIK | 0.011173 | 0.474754 | 0.856742 |
| IL10 | 0.011174 | 0.474754 | 1.143429 |
| HIST2H2AB | 0.011197 | 0.474754 | 0.288132 |
| SCL000860.1_1 | 0.011206 | 0.474754 | 0.820343 |
| MLH1 | 0.011213 | 0.474754 | 0.760173 |
| 0610010I05RIK | 0.011227 | 0.474754 | 2.059556 |
| LOC385130 | 0.011228 | 0.474754 | 1.080775 |
| COX18 | 0.011268 | 0.474754 | 0.521546 |
| BC017643 | 0.011274 | 0.474754 | 1.478739 |
| 2410166I05RIK | 0.011279 | 0.474754 | 1.332344 |
| CAMTA2 | 0.011291 | 0.474754 | 1.70874 |
| LIG3 | 0.011321 | 0.474754 | 0.84113 |
| 1700021K02RIK | 0.011323 | 0.474754 | 1.127713 |
| LBA1 | 0.011342 | 0.474754 | 0.844537 |
| LOC385109 | 0.01135 | 0.474754 | 1.047996 |
| TRAPPC2 | 0.011353 | 0.474754 | 1.373668 |
| HAT1 | 0.011366 | 0.474754 | 0.701412 |
| YIPF7 | 0.011412 | 0.474754 | 1.061472 |
| C230064B05RIK | 0.011419 | 0.474754 | 0.800718 |
| SLC5A11 | 0.011424 | 0.474754 | 0.954621 |
| SPG7 | 0.011428 | 0.474754 | 0.830297 |
| PHC3 | 0.01143 | 0.474754 | 1.167509 |
| REX3 | 0.011471 | 0.474754 | 1.124435 |
| 2010321I05RIK | 0.011482 | 0.474754 | 1.460944 |
| TCP1 | 0.011499 | 0.474754 | 0.525465 |
| 4930560E09RIK | 0.011506 | 0.474754 | 1.130687 |
| DDX60 | 0.011513 | 0.474754 | 1.080201 |
| STX7 | 0.011537 | 0.474754 | 1.675549 |
| AHNAK2 | 0.011562 | 0.474754 | 1.976571 |
| LOC385078 | 0.011579 | 0.474754 | 0.898651 |
| PDGFC | 0.011589 | 0.474754 | 0.940565 |
| NUMA1 | 0.011619 | 0.474754 | 1.177587 |
| A430031C20RIK | 0.011623 | 0.474754 | 0.989611 |
| LOC329698 | 0.011624 | 0.474754 | 0.868983 |
| ZFP819 | 0.01164 | 0.474754 | 1.112419 |
| SHISA4 | 0.011646 | 0.474754 | 1.590228 |
| LOC277744 | 0.011656 | 0.474754 | 1.064518 |
| LOC194492 | 0.011663 | 0.474754 | 0.875229 |
| LONP1 | 0.011718 | 0.475992 | 0.689681 |
| PTTG1IP | 0.011733 | 0.475992 | 1.515017 |
| BC034507 | 0.01174 | 0.475992 | 0.769273 |
| OLFR1277 | 0.011796 | 0.477648 | 0.892877 |
| PLS3 | 0.011813 | 0.477726 | 1.237018 |
| LOC668837 | 0.011887 | 0.479654 | 0.768544 |
| NDRL | 0.0119 | 0.479654 | 1.580813 |
| TRIM17 | 0.011916 | 0.479654 | 0.876261 |
| LOC100042514 | 0.011936 | 0.479654 | 1.656648 |
| LOC100048295 | 0.011939 | 0.479654 | 1.247811 |
| UGT8A | 0.011985 | 0.48009 | 0.90945 |
| LOC271022 | 0.012024 | 0.48009 | 1.029326 |
| RPL21 | 0.012025 | 0.48009 | 0.6598 |
| PCYT2 | 0.012046 | 0.48009 | 1.45488 |
| LOC329750 | 0.012061 | 0.48009 | 0.476869 |
| FYCO1 | 0.012095 | 0.48009 | 1.27253 |
| V1RD13 | 0.012097 | 0.48009 | 1.163523 |
| GABARAP | 0.012106 | 0.48009 | 1.918972 |
| RTN4RL1 | 0.012141 | 0.48009 | 0.767746 |
| ITGB1BP3 | 0.012148 | 0.48009 | 0.908841 |
| ARAF | 0.01215 | 0.48009 | 1.09642 |
| SCL0001546.1_9 | 0.012168 | 0.48009 | 1.402208 |
| PKMYT1 | 0.012177 | 0.48009 | 0.472603 |
| FABP5 | 0.012189 | 0.48009 | 0.887837 |
| MGEA5 | 0.012214 | 0.48009 | 0.745975 |
| 4932413O14RIK | 0.012221 | 0.48009 | 0.96462 |
| FAM107A | 0.012228 | 0.48009 | 1.125111 |
| LCAT | 0.012246 | 0.48009 | 1.111571 |
| 2810029C07RIK | 0.012279 | 0.48009 | 0.95829 |
| AA881470 | 0.012287 | 0.48009 | 1.134063 |
| EG277333 | 0.012306 | 0.48009 | 1.237618 |
| RFC4 | 0.012351 | 0.48009 | 0.366978 |
| WBP7 | 0.012361 | 0.48009 | 1.328778 |
| ISY1 | 0.012372 | 0.48009 | 0.619854 |
| RAB19 | 0.012426 | 0.48009 | 0.855437 |
| 1810007A24RIK | 0.012462 | 0.48009 | 0.827348 |
| SNX7 | 0.012487 | 0.48009 | 1.326784 |
| LOC100048832 | 0.012491 | 0.48009 | 0.633566 |
| TERA-PENDING | 0.01252 | 0.48009 | 0.588902 |
| LOC331507 | 0.012536 | 0.48009 | 0.655348 |
| IL17RC | 0.012537 | 0.48009 | 1.944861 |
| CSTB | 0.012544 | 0.48009 | 1.567719 |
| TCN2 | 0.01258 | 0.48009 | 2.158506 |
| DNAJC13 | 0.012598 | 0.48009 | 1.443463 |
| A630053N20RIK | 0.012607 | 0.48009 | 0.643971 |
| NOC2L | 0.012609 | 0.48009 | 0.480608 |
| ZFAND6 | 0.012622 | 0.48009 | 1.125266 |
| LOC100048050 | 0.012643 | 0.48009 | 1.091945 |
| A530058G07RIK | 0.01265 | 0.48009 | 1.233251 |
| COL7A1 | 0.012658 | 0.48009 | 2.273214 |
| 1700112H15RIK | 0.012679 | 0.48009 | 1.024131 |
| LOC100047193 | 0.012695 | 0.48009 | 1.156234 |
| OLFR853 | 0.01271 | 0.48009 | 1.141634 |
| UBL4 | 0.01272 | 0.48009 | 1.187697 |
| V1RB1 | 0.012721 | 0.48009 | 1.102516 |
| IPAS | 0.012726 | 0.48009 | 0.894777 |
| RNASEH2A | 0.012737 | 0.48009 | 0.470011 |
| IBRDC3 | 0.012745 | 0.48009 | 1.292771 |
| SLC25A5 | 0.012751 | 0.48009 | 0.488129 |
| 6720463M24RIK | 0.012795 | 0.48009 | 0.475813 |
| POLR3B | 0.012805 | 0.48009 | 0.690143 |
| RAB11B | 0.012816 | 0.48009 | 0.847469 |
| CENPF | 0.01284 | 0.48009 | 0.713507 |
| PAIP2 | 0.01285 | 0.48009 | 0.843758 |
| ITGA8 | 0.012856 | 0.48009 | 0.851395 |
| ZFP2 | 0.01286 | 0.48009 | 1.129721 |
| MITF | 0.01288 | 0.48009 | 0.905613 |
| CYP8B1 | 0.012885 | 0.48009 | 1.162797 |
| SPDYB | 0.012906 | 0.48009 | 1.13477 |
| DNAHC11 | 0.012932 | 0.48009 | 0.454242 |
| ITPRIP | 0.012959 | 0.48009 | 1.654888 |
| E430025C17RIK | 0.012989 | 0.48009 | 0.805654 |
| LOC100042351 | 0.013006 | 0.48009 | 1.048069 |
| BTN2A2 | 0.01304 | 0.48009 | 0.81447 |
| TAS2R120 | 0.013048 | 0.48009 | 0.932796 |
| CYP2C37 | 0.013051 | 0.48009 | 0.876748 |
| SNRPD1 | 0.013053 | 0.48009 | 0.602208 |
| LOC381034 | 0.013089 | 0.48009 | 0.891414 |
| 6330414G02RIK | 0.013096 | 0.48009 | 2.591522 |
| LOC632431 | 0.013102 | 0.48009 | 0.836092 |
| MKNK2 | 0.013111 | 0.48009 | 1.491158 |
| SCLT1 | 0.013116 | 0.48009 | 0.734703 |
| CCT4 | 0.01313 | 0.48009 | 0.543744 |
| A930009M04RIK | 0.013135 | 0.48009 | 0.616084 |
| GZMM | 0.01314 | 0.48009 | 0.926139 |
| EXOSC3 | 0.013154 | 0.48009 | 0.871235 |
| DIP2C | 0.013161 | 0.48009 | 1.208039 |
| H2-T22 | 0.013163 | 0.48009 | 0.964687 |
| UBE2S | 0.013254 | 0.481136 | 0.525939 |
| A730006E03RIK | 0.013287 | 0.481136 | 0.754364 |
| DYNC1H1 | 0.013309 | 0.481136 | 1.352285 |
| GTF2H4 | 0.01331 | 0.481136 | 0.683336 |
| OLFR1338 | 0.013327 | 0.481136 | 0.847039 |
| PVRL2 | 0.013331 | 0.481136 | 1.708385 |
| SHCBP1 | 0.013344 | 0.481136 | 0.741867 |
| IFRD2 | 0.013358 | 0.481136 | 0.390664 |
| LCE1M | 0.013375 | 0.481136 | 1.055359 |
| PRMT1 | 0.013391 | 0.481136 | 1.340155 |
| D4ERTD196E | 0.013414 | 0.481136 | 0.888987 |
| ORM2 | 0.013447 | 0.481136 | 0.70125 |
| LOC381607 | 0.013455 | 0.481136 | 0.839693 |
| RAB43 | 0.013459 | 0.481136 | 1.267718 |
| UHMK1 | 0.013475 | 0.481136 | 0.843017 |
| LOC238662 | 0.0135 | 0.481136 | 1.119483 |
| SLC23A2 | 0.013515 | 0.481136 | 1.279281 |
| D12ERTD647E | 0.013519 | 0.481136 | 1.653283 |
| CYBB | 0.013519 | 0.481136 | 1.118424 |
| GNAL | 0.013524 | 0.481136 | 0.905446 |
| PIP5K1B | 0.013543 | 0.481136 | 2.282108 |
| 9530062N20RIK | 0.013598 | 0.481136 | 0.701833 |
| CYBASC3 | 0.013606 | 0.481136 | 1.491227 |
| 4930511M11RIK | 0.013666 | 0.481136 | 1.209883 |
| ELOF1 | 0.013674 | 0.481136 | 1.269565 |
| LSM4 | 0.013692 | 0.481136 | 0.469978 |
| HSPA4 | 0.013692 | 0.481136 | 0.554132 |
| CPLX2 | 0.013694 | 0.481136 | 0.888514 |
| OLFR522 | 0.013723 | 0.481136 | 1.125917 |
| E030043F19RIK | 0.013735 | 0.481136 | 1.181812 |
| BPI | 0.013741 | 0.481136 | 1.194522 |
| PARP4 | 0.013747 | 0.481136 | 1.552472 |
| ATP13A2 | 0.013748 | 0.481136 | 1.220439 |
| TMEM109 | 0.01378 | 0.481136 | 0.631855 |
| SYT15 | 0.013787 | 0.481136 | 0.878268 |
| UTP14A | 0.013824 | 0.481136 | 0.553594 |
| ZFP791 | 0.013826 | 0.481136 | 0.892196 |
| PTPRF | 0.013857 | 0.481136 | 1.882522 |
| SNF1LK | 0.013878 | 0.481136 | 1.76296 |
| FAM20A | 0.013902 | 0.481136 | 1.497096 |
| SPC24 | 0.013904 | 0.481136 | 0.259331 |
| MRPL17 | 0.013905 | 0.481136 | 0.882499 |
| GJE1 | 0.013917 | 0.481136 | 0.847352 |
| MAZ | 0.013942 | 0.481136 | 1.200443 |
| GTF2F1 | 0.013945 | 0.481136 | 1.241255 |
| LOC241593 | 0.013947 | 0.481136 | 0.782159 |
| ITGB5 | 0.013948 | 0.481136 | 1.718321 |
| BC048562 | 0.013967 | 0.481136 | 0.967589 |
| GLT1D1 | 0.013968 | 0.481136 | 0.770037 |
| 6130401L20RIK | 0.013972 | 0.481136 | 0.85364 |
| LOC385644 | 0.013992 | 0.48123 | 2.035476 |
| SRD5A3 | 0.014026 | 0.48123 | 1.290383 |
| GDPD2 | 0.014031 | 0.48123 | 2.620121 |
| 1810006K21RIK | 0.01406 | 0.48123 | 1.810849 |
| RHOG | 0.014066 | 0.48123 | 1.419288 |
| 9430029L20RIK | 0.014068 | 0.48123 | 1.720785 |
| BC042423 | 0.014126 | 0.481655 | 1.448139 |
| CART | 0.014153 | 0.481655 | 1.181129 |
| NEDD4B | 0.014155 | 0.481655 | 1.261873 |
| LOC383865 | 0.014171 | 0.481655 | 1.12714 |
| ZWILCH | 0.014175 | 0.481655 | 0.483638 |
| LOC677375 | 0.01421 | 0.481655 | 1.082625 |
| TRAPPC1 | 0.014223 | 0.481655 | 1.713723 |
| 5730427M17RIK | 0.014278 | 0.481655 | 1.10898 |
| TAS1R3 | 0.014286 | 0.481655 | 1.046689 |
| ADAM5 | 0.014288 | 0.481655 | 0.87194 |
| 2210408I21RIK | 0.0143 | 0.481655 | 0.700036 |
| FRRS1 | 0.014313 | 0.481655 | 1.094547 |
| SCL000408.1_6 | 0.014314 | 0.481655 | 0.522982 |
| SLCO6B1 | 0.014332 | 0.481655 | 0.908778 |
| EIF2B3 | 0.014337 | 0.481655 | 0.510683 |
| HNRPK | 0.014354 | 0.481655 | 0.690653 |
| D4WSU132E | 0.014379 | 0.481655 | 0.456356 |
| AHI1 | 0.014382 | 0.481655 | 0.719617 |
| C130072A16RIK | 0.014397 | 0.481655 | 0.543568 |
| TNFSF9 | 0.014407 | 0.481655 | 1.221737 |
| CPE | 0.014434 | 0.481655 | 1.966549 |
| MAP3K11 | 0.014447 | 0.481655 | 1.363455 |
| RNF148 | 0.014466 | 0.481655 | 1.198918 |
| CTDSPL | 0.014471 | 0.481655 | 1.485244 |
| OLFR466 | 0.014489 | 0.481655 | 1.210721 |
| LOC218617 | 0.014529 | 0.481655 | 1.317467 |
| MORC2B | 0.014553 | 0.481655 | 1.183205 |
| LOC100045680 | 0.014566 | 0.481655 | 1.239622 |
| LOC380716 | 0.014578 | 0.481655 | 0.907876 |
| OLFR654 | 0.014578 | 0.481655 | 1.141766 |
| HSP90AA1 | 0.014584 | 0.481655 | 0.60898 |
| A830023I12RIK | 0.014625 | 0.481655 | 1.248014 |
| ARG1 | 0.014633 | 0.481655 | 1.143218 |
| ADAMTS7 | 0.014634 | 0.481655 | 1.78563 |
| PRCP | 0.014652 | 0.481655 | 1.674812 |
| TWF2 | 0.014657 | 0.481655 | 1.27256 |
| B930007L02RIK | 0.014658 | 0.481655 | 0.74489 |
| AKAP2 | 0.014677 | 0.481669 | 1.556746 |
| YES | 0.014707 | 0.481669 | 0.763641 |
| DLGAP5 | 0.014715 | 0.481669 | 0.232392 |
| DPP7 | 0.014724 | 0.481669 | 1.496578 |
| SCL0001849.1_2273 | 0.014749 | 0.481669 | 1.940372 |
| IVNS1ABP | 0.014763 | 0.481669 | 0.538667 |
| 5930429A15RIK | 0.014777 | 0.481669 | 2.039525 |
| NUP205 | 0.014794 | 0.481669 | 0.368397 |
| RAB28 | 0.014854 | 0.481669 | 0.810004 |
| WDR34 | 0.014904 | 0.481669 | 0.607785 |
| TBN-PENDING | 0.01491 | 0.481669 | 0.895377 |
| DBNDD2 | 0.014912 | 0.481669 | 0.871658 |
| KCNK15 | 0.014923 | 0.481669 | 1.071699 |
| D430006B11RIK | 0.014938 | 0.481669 | 0.845162 |
| AI464131 | 0.01496 | 0.481669 | 1.740055 |
| DDX20 | 0.01497 | 0.481669 | 0.62626 |
| LOC381799 | 0.014974 | 0.481669 | 1.203303 |
| PTPN21 | 0.014977 | 0.481669 | 1.258291 |
| LOC100047905 | 0.014987 | 0.481669 | 0.704042 |
| SPNB2 | 0.015009 | 0.481669 | 1.085806 |
| 4930572J05RIK | 0.01501 | 0.481669 | 0.391676 |
| SLC4A2 | 0.015029 | 0.481669 | 1.966095 |
| B230339H12RIK | 0.01505 | 0.481669 | 1.794398 |
| C330023M02RIK | 0.015123 | 0.481669 | 0.399703 |
| EIF3EIP | 0.015132 | 0.481669 | 0.759085 |
| NEB | 0.015138 | 0.481669 | 1.144143 |
| V1RD18 | 0.015139 | 0.481669 | 1.034691 |
| NBEAL2 | 0.015158 | 0.481669 | 1.126671 |
| BC056474 | 0.015174 | 0.481669 | 0.792381 |
| SLAMF1 | 0.015185 | 0.481669 | 1.065133 |
| LOC100044968 | 0.015187 | 0.481669 | 1.519926 |
| NDUFA3 | 0.015194 | 0.481669 | 1.46544 |
| TK2 | 0.015217 | 0.481669 | 1.324425 |
| SUPT5H | 0.015233 | 0.481669 | 1.176227 |
| OLFR176 | 0.015251 | 0.481669 | 0.958909 |
| BC030440 | 0.01526 | 0.481669 | 0.895294 |
| LOC383668 | 0.015275 | 0.481669 | 0.941609 |
| CXCL3 | 0.015295 | 0.481669 | 0.882173 |
| PABPC1 | 0.015314 | 0.481669 | 0.732415 |
| C230060D12RIK | 0.015318 | 0.481669 | 1.072616 |
| KPNA6 | 0.015332 | 0.481669 | 1.140157 |
| GDF2 | 0.015355 | 0.481669 | 1.047343 |
| RFC5 | 0.015367 | 0.481669 | 0.311982 |
| DDX4 | 0.015371 | 0.481669 | 1.084026 |
| IMPDH2 | 0.015392 | 0.481669 | 0.410561 |
| OIP5 | 0.015402 | 0.481669 | 0.215009 |
| A430061O19RIK | 0.015405 | 0.481669 | 0.769521 |
| TMEM100 | 0.01541 | 0.481669 | 1.042707 |
| 2310024N18RIK | 0.015423 | 0.481669 | 1.396162 |
| 2610028H07RIK | 0.015442 | 0.48176 | 1.439299 |
| 4933402J24RIK | 0.015487 | 0.481915 | 0.961594 |
| ATPAF2 | 0.015499 | 0.481915 | 0.690621 |
| SYT17 | 0.01551 | 0.481915 | 0.933853 |
| TRIP10 | 0.015513 | 0.481915 | 1.50139 |
| OTOG | 0.015525 | 0.481915 | 0.898755 |
| C230029D21RIK | 0.015541 | 0.481915 | 0.915099 |
| 8430410K20RIK | 0.015601 | 0.483217 | 1.413233 |
| RNASEH2B | 0.015615 | 0.483217 | 0.580674 |
| MYCBP2 | 0.01564 | 0.483217 | 1.181265 |
| GLDC | 0.015645 | 0.483217 | 1.780275 |
| LOC100038882 | 0.015664 | 0.483217 | 1.352505 |
| LPCAT3 | 0.015761 | 0.483217 | 1.237647 |
| ISYNA1 | 0.015762 | 0.483217 | 0.394218 |
| OVGP1 | 0.015766 | 0.483217 | 0.853759 |
| 2310043D08RIK | 0.015768 | 0.483217 | 0.762583 |
| 4933414E15RIK | 0.015775 | 0.483217 | 0.901188 |
| STAT6 | 0.015779 | 0.483217 | 0.795077 |
| C920025C15RIK | 0.015806 | 0.483217 | 0.948465 |
| KDELC1 | 0.015826 | 0.483217 | 0.67594 |
| IKBKG | 0.015828 | 0.483217 | 1.455889 |
| D6WSU163E | 0.015862 | 0.483217 | 0.810453 |
| TINAGL | 0.015877 | 0.483217 | 2.001571 |
| LOC330517 | 0.015923 | 0.483217 | 1.041455 |
| 4833409N03RIK | 0.01596 | 0.483217 | 1.382839 |
| LOC100044756 | 0.015961 | 0.483217 | 1.158186 |
| NUP107 | 0.015985 | 0.483217 | 0.506652 |
| 9130230L23RIK | 0.015993 | 0.483217 | 0.848625 |
| LOC641221 | 0.016007 | 0.483217 | 0.852596 |
| TDRD7 | 0.016008 | 0.483217 | 1.558365 |
| NR4A2 | 0.01601 | 0.483217 | 1.620007 |
| EDARADD | 0.016031 | 0.483217 | 1.119535 |
| EPN1 | 0.016036 | 0.483217 | 1.533364 |
| LOC100047930 | 0.016044 | 0.483217 | 1.244558 |
| A730047D20RIK | 0.016049 | 0.483217 | 1.210945 |
| FGL1 | 0.01606 | 0.483217 | 1.392908 |
| DRD2 | 0.016081 | 0.483217 | 1.24292 |
| BC006779 | 0.016095 | 0.483217 | 1.429159 |
| 4930589L23RIK | 0.016104 | 0.483217 | 1.138394 |
| D130023B06RIK | 0.016119 | 0.483217 | 0.930536 |
| DNMT1 | 0.01614 | 0.483217 | 0.459096 |
| SPATA5 | 0.016151 | 0.483217 | 0.872826 |
| RAD1 | 0.016167 | 0.483217 | 0.538021 |
| C030002J06RIK | 0.016177 | 0.483217 | 0.850097 |
| 1110003F05RIK | 0.016178 | 0.483217 | 1.123059 |
| SPSB1 | 0.016194 | 0.483225 | 1.697408 |
| PLXNB1 | 0.016224 | 0.483651 | 0.858923 |
| SEMA3C | 0.016261 | 0.483822 | 1.226602 |
| LOC237877 | 0.016271 | 0.483822 | 0.482266 |
| RSL1D1 | 0.016276 | 0.483822 | 0.459669 |
| LOC385231 | 0.016314 | 0.484486 | 0.944463 |
| RAI1 | 0.016334 | 0.484603 | 0.78196 |
| TIMELESS | 0.016459 | 0.485608 | 0.444093 |
| REV3L | 0.016465 | 0.485608 | 1.339258 |
| GM1821 | 0.016496 | 0.485608 | 1.379361 |
| HTATSF1 | 0.016501 | 0.485608 | 0.703229 |
| BXDC1 | 0.016504 | 0.485608 | 0.713788 |
| TMEM161B | 0.016505 | 0.485608 | 0.84839 |
| CLA3 | 0.016518 | 0.485608 | 0.425953 |
| 2610110G12RIK | 0.016524 | 0.485608 | 1.128521 |
| OLFR1203 | 0.016576 | 0.485608 | 0.911912 |
| RWDD2 | 0.016617 | 0.485608 | 1.276034 |
| 2310002J15RIK | 0.016619 | 0.485608 | 0.805524 |
| COIL | 0.016653 | 0.485608 | 0.766204 |
| PLEKHO2 | 0.016654 | 0.485608 | 1.521049 |
| CCT8 | 0.016658 | 0.485608 | 0.630252 |
| 1110002B05RIK | 0.016671 | 0.485608 | 0.742622 |
| 1110049F12RIK | 0.016731 | 0.485608 | 0.636853 |
| 3010002L02RIK | 0.016735 | 0.485608 | 1.158989 |
| AHCTF1 | 0.016746 | 0.485608 | 0.450074 |
| ITPRIPL2 | 0.01676 | 0.485608 | 1.910257 |
| 1110002E22RIK | 0.016792 | 0.485608 | 1.452428 |
| 3110031I02RIK | 0.016855 | 0.485608 | 0.924856 |
| 6030455H03RIK | 0.016856 | 0.485608 | 0.899233 |
| SLC7A1 | 0.016876 | 0.485608 | 0.936121 |
| MRPS35 | 0.016899 | 0.485608 | 0.536618 |
| RAB12 | 0.016936 | 0.485608 | 1.186655 |
| F830005D05RIK | 0.016948 | 0.485608 | 0.868903 |
| LOC100039829 | 0.016979 | 0.485608 | 0.836672 |
| DEK | 0.017023 | 0.485608 | 0.53216 |
| LOC100047226 | 0.017042 | 0.485608 | 0.790443 |
| PCOLCE | 0.017068 | 0.485608 | 1.313424 |
| 2310011J03RIK | 0.017077 | 0.485608 | 1.574761 |
| ZBTB11 | 0.017078 | 0.485608 | 0.875756 |
| SGCG | 0.01708 | 0.485608 | 0.922785 |
| SREBF2 | 0.017091 | 0.485608 | 0.766398 |
| RBMX | 0.017107 | 0.485608 | 0.445696 |
| DPF1 | 0.017118 | 0.485608 | 1.089173 |
| VAT1 | 0.017132 | 0.485608 | 1.679386 |
| TSPAN7 | 0.017143 | 0.485608 | 1.459527 |
| SEC31A | 0.017161 | 0.485608 | 1.484866 |
| CCDC68 | 0.017181 | 0.485608 | 1.043815 |
| PARP1 | 0.017186 | 0.485608 | 0.425825 |
| LRP1 | 0.017188 | 0.485608 | 1.911582 |
| LOC209281 | 0.017196 | 0.485608 | 0.760349 |
| ESCO2 | 0.017196 | 0.485608 | 0.282026 |
| 1110048D14RIK | 0.017199 | 0.485608 | 1.421092 |
| 9330120H11RIK | 0.017201 | 0.485608 | 1.224053 |
| TSR2 | 0.017227 | 0.485608 | 0.696583 |
| BC037594 | 0.017231 | 0.485608 | 1.055408 |
| LOC383621 | 0.017233 | 0.485608 | 0.792363 |
| ADRB3 | 0.017259 | 0.485608 | 1.007072 |
| RHEB | 0.017266 | 0.485608 | 0.738328 |
| NOL8 | 0.01727 | 0.485608 | 0.700278 |
| 3110003A17RIK | 0.017294 | 0.485608 | 1.088972 |
| DDB1 | 0.017339 | 0.485608 | 1.298338 |
| DHX30 | 0.017381 | 0.485608 | 0.841869 |
| USP21 | 0.017392 | 0.485608 | 1.397421 |
| 2900052P03RIK | 0.017403 | 0.485608 | 0.800755 |
| LOC230622 | 0.017405 | 0.485608 | 0.858327 |
| CEP55 | 0.017437 | 0.485608 | 0.310917 |
| USP14 | 0.017464 | 0.485608 | 0.599972 |
| ZFP191 | 0.017509 | 0.485608 | 0.918594 |
| 1810020D17RIK | 0.017527 | 0.485608 | 1.376718 |
| PPP3R1 | 0.017576 | 0.485608 | 1.337927 |
| LBR | 0.017581 | 0.485608 | 0.588168 |
| DPP8 | 0.017583 | 0.485608 | 1.151701 |
| SLC41A3 | 0.017598 | 0.485608 | 1.292174 |
| LOC100039094 | 0.0176 | 0.485608 | 1.060712 |
| C030026E19RIK | 0.0176 | 0.485608 | 0.816391 |
| AK122498 | 0.01761 | 0.485608 | 0.857971 |
| SCL0003651.1_17 | 0.01762 | 0.485608 | 1.207676 |
| MRPL3 | 0.017624 | 0.485608 | 0.599625 |
| DEFCR-RS1 | 0.017629 | 0.485608 | 0.84998 |
| OTUD7B | 0.01764 | 0.485608 | 1.033043 |
| LOC385238 | 0.017658 | 0.485608 | 0.871054 |
| GSG2 | 0.017706 | 0.485608 | 0.545733 |
| ATF7IP2 | 0.017707 | 0.485608 | 1.141186 |
| 4931403M11RIK | 0.017736 | 0.485608 | 1.078306 |
| LOC624662 | 0.017779 | 0.485608 | 1.496231 |
| QTRT1 | 0.017788 | 0.485608 | 0.471632 |
| DGKQ | 0.017795 | 0.485608 | 0.911849 |
| BRD3 | 0.017801 | 0.485608 | 0.859856 |
| ELTD1 | 0.017811 | 0.485608 | 0.920145 |
| FANCD2 | 0.017836 | 0.485608 | 0.278407 |
| LOC381427 | 0.017855 | 0.485608 | 1.0816 |
| CST6 | 0.017863 | 0.485608 | 1.062527 |
| GPR19 | 0.017886 | 0.485608 | 0.629669 |
| 1700034H14RIK | 0.017896 | 0.485608 | 0.515556 |
| MORC2A | 0.017902 | 0.485608 | 1.589236 |
| CFD | 0.017916 | 0.485608 | 0.858546 |
| A830007N09RIK | 0.017936 | 0.485608 | 1.046811 |
| TAX1BP3 | 0.017959 | 0.485608 | 1.615895 |
| TST | 0.017999 | 0.485608 | 0.412567 |
| A930035G08RIK | 0.018016 | 0.485608 | 1.034739 |
| FCNA | 0.018018 | 0.485608 | 1.212233 |
| LOC385308 | 0.01802 | 0.485608 | 1.1304 |
| 6620401K05RIK | 0.018026 | 0.485608 | 0.741867 |
| AK7 | 0.018073 | 0.485608 | 0.943896 |
| MYADM | 0.018078 | 0.485608 | 1.854033 |
| AW540478 | 0.018116 | 0.485608 | 0.806399 |
| NEK8 | 0.018133 | 0.485608 | 1.220185 |
| CYC1 | 0.018144 | 0.485608 | 0.53812 |
| KBTBD5 | 0.018224 | 0.485608 | 1.181648 |
| UXT | 0.018229 | 0.485608 | 0.653896 |
| TADA2L | 0.018232 | 0.485608 | 0.758174 |
| MDH2 | 0.018242 | 0.485608 | 0.511805 |
| ORC5L | 0.01825 | 0.485608 | 0.764153 |
| MYBBP1A | 0.0183 | 0.485608 | 0.426317 |
| 9930122J16RIK | 0.018304 | 0.485608 | 0.784657 |
| TIMM9 | 0.018311 | 0.485608 | 0.515973 |
| 5330403O16RIK | 0.01832 | 0.485608 | 1.233109 |
| SACS | 0.01834 | 0.485608 | 0.834085 |
| TRIP13 | 0.018377 | 0.485608 | 0.739045 |
| SCL0002785.1_49 | 0.018455 | 0.485608 | 1.375224 |
| SNORA65 | 0.018456 | 0.485608 | 0.436101 |
| PCBP3 | 0.018478 | 0.485608 | 1.151249 |
| 2810012D02RIK | 0.018486 | 0.485608 | 0.534811 |
| DHRS1 | 0.018508 | 0.485608 | 1.421027 |
| 2810441E16RIK | 0.018531 | 0.485608 | 0.771052 |
| LOC554292 | 0.018531 | 0.485608 | 1.046423 |
| LOC100047712 | 0.018536 | 0.485608 | 0.535689 |
| OLFR611 | 0.018542 | 0.485608 | 0.907058 |
| MAGED2 | 0.018554 | 0.485608 | 1.967368 |
| ILF2 | 0.018579 | 0.485608 | 0.644387 |
| BTNL7 | 0.01858 | 0.485608 | 0.94466 |
| FBLIM1 | 0.018587 | 0.485608 | 1.618249 |
| GNPTG | 0.018597 | 0.485608 | 1.379903 |
| A130093I21RIK | 0.018602 | 0.485608 | 1.145782 |
| CCDC23 | 0.018604 | 0.485608 | 2.035524 |
| UBAP2 | 0.018615 | 0.485608 | 0.79996 |
| ATP5L | 0.018617 | 0.485608 | 1.239135 |
| TSEN2 | 0.018619 | 0.485608 | 0.569302 |
| LOC544718 | 0.018622 | 0.485608 | 1.073186 |
| D130054H18RIK | 0.018626 | 0.485608 | 0.773479 |
| 9630025G02RIK | 0.018635 | 0.485608 | 0.84218 |
| RWDD1 | 0.018668 | 0.485608 | 0.743463 |
| ZNF512B | 0.018679 | 0.485608 | 1.354037 |
| OLFR812 | 0.01868 | 0.485608 | 0.914317 |
| GNB1L | 0.018682 | 0.485608 | 0.737101 |
| ZFYVE27 | 0.018686 | 0.485608 | 1.345025 |
| TGFB1 | 0.018687 | 0.485608 | 1.526154 |
| EPHB6 | 0.018713 | 0.485608 | 1.909286 |
| ACTA2 | 0.01875 | 0.485608 | 1.597261 |
| EG632802 | 0.018785 | 0.485608 | 1.594164 |
| 9130215G10RIK | 0.018803 | 0.485608 | 1.053093 |
| LOC633677 | 0.018824 | 0.485608 | 1.072764 |
| IQGAP3 | 0.018841 | 0.485608 | 0.521968 |
| 4933406J08RIK | 0.018856 | 0.485608 | 1.088418 |
| A730080H06RIK | 0.018862 | 0.485608 | 1.147664 |
| NOD1 | 0.018868 | 0.485608 | 1.541677 |
| 4632411B12RIK | 0.018872 | 0.485608 | 1.216217 |
| PSMG2 | 0.018914 | 0.485608 | 0.575492 |
| F830045P16RIK | 0.018929 | 0.485608 | 1.151116 |
| PRKAR2B | 0.018931 | 0.485608 | 0.851946 |
| D730042P09RIK | 0.018932 | 0.485608 | 0.582192 |
| SLC1A4 | 0.018948 | 0.485608 | 0.909072 |
| E530004N13RIK | 0.018973 | 0.485608 | 0.852024 |
| SLC6A8 | 0.018991 | 0.485608 | 1.425894 |
| ARPC3 | 0.019005 | 0.485608 | 1.519223 |
| FBXW17 | 0.019011 | 0.485608 | 1.395163 |
| LOC385361 | 0.019013 | 0.485608 | 1.137079 |
| D230004N23RIK | 0.019015 | 0.485608 | 0.854587 |
| ALAD | 0.019021 | 0.485608 | 0.663576 |
| LOC628101 | 0.019032 | 0.485608 | 0.908841 |
| NLRP2 | 0.019032 | 0.485608 | 0.954886 |
| TUBD1 | 0.019045 | 0.485608 | 0.786308 |
| ATP2B1 | 0.019068 | 0.485608 | 1.45065 |
| GNA-RS1 | 0.01908 | 0.485608 | 1.13971 |
| RNPEP | 0.019084 | 0.485608 | 0.686326 |
| LOC382846 | 0.019089 | 0.485608 | 1.142637 |
| MND1 | 0.019098 | 0.485608 | 0.338853 |
| LOC638794 | 0.019103 | 0.485608 | 0.815524 |
| LOC233466 | 0.019108 | 0.485608 | 1.171752 |
| 5033414K04RIK | 0.019136 | 0.485608 | 1.171589 |
| FARSB | 0.01914 | 0.485608 | 0.409613 |
| OLFR195 | 0.019166 | 0.485608 | 0.868662 |
| BIRC4 | 0.019172 | 0.485608 | 0.968797 |
| CGRRF1 | 0.019203 | 0.485608 | 1.266605 |
| LOC380756 | 0.019224 | 0.485608 | 0.584983 |
| KNTC1 | 0.019251 | 0.485608 | 0.282456 |
| TNPO1 | 0.019251 | 0.485608 | 0.861447 |
| 4930447J18RIK | 0.019253 | 0.485608 | 1.054676 |
| SLC6A9 | 0.019295 | 0.485608 | 0.919465 |
| CST13 | 0.019339 | 0.485608 | 1.104862 |
| 0610008F07RIK | 0.019355 | 0.485608 | 1.139078 |
| NUCB1 | 0.019367 | 0.485608 | 1.440097 |
| EG666002 | 0.019377 | 0.485608 | 1.106779 |
| LOC668408 | 0.019404 | 0.485608 | 0.937116 |
| STARD7 | 0.019417 | 0.485608 | 0.62506 |
| BMP3 | 0.019428 | 0.485608 | 0.948531 |
| RAB11FIP5 | 0.019456 | 0.485608 | 1.982976 |
| CASC4 | 0.019468 | 0.485608 | 0.761668 |
| OOG2 | 0.019472 | 0.485608 | 1.126932 |
| LOC669660 | 0.019493 | 0.485608 | 1.38671 |
| ZFP90 | 0.019494 | 0.485608 | 1.483392 |
| OLFR324 | 0.01954 | 0.485608 | 0.944573 |
| JUND1 | 0.019553 | 0.485608 | 1.352598 |
| KCNQ3 | 0.019565 | 0.485608 | 0.788582 |
| D630036H09RIK | 0.019568 | 0.485608 | 1.50967 |
| GOLT1B | 0.01957 | 0.485608 | 1.12241 |
| POLE | 0.019589 | 0.485608 | 0.453781 |
| LOC219145 | 0.019601 | 0.485608 | 0.447306 |
| LOC385904 | 0.019613 | 0.485608 | 1.087589 |
| D030065J16RIK | 0.01962 | 0.485608 | 1.089526 |
| 4732423C17RIK | 0.01962 | 0.485608 | 0.808639 |
| ZFP101 | 0.019632 | 0.485608 | 0.724354 |
| A830007N20RIK | 0.019638 | 0.485608 | 1.100175 |
| PLA2G2E | 0.019661 | 0.485608 | 1.167023 |
| SNAP23 | 0.019699 | 0.485608 | 0.905341 |
| NUB1 | 0.019719 | 0.485608 | 1.400394 |
| NUP85 | 0.019723 | 0.485608 | 0.477619 |
| MLKL | 0.019724 | 0.485608 | 1.299269 |
| LOC381977 | 0.01974 | 0.485608 | 1.16312 |
| GNAI2 | 0.019742 | 0.485608 | 1.515367 |
| A430088H15RIK | 0.019762 | 0.485608 | 0.73625 |
| CLPP | 0.019762 | 0.485608 | 0.712025 |
| OLFR1212 | 0.019767 | 0.485608 | 1.068485 |
| C330011K17RIK | 0.019786 | 0.485613 | 1.127713 |
| 1200003C05RIK | 0.019799 | 0.485613 | 1.252983 |
| EG331493 | 0.019826 | 0.485783 | 0.944245 |
| PCSK6 | 0.019838 | 0.485783 | 1.062527 |
| LOC100040413 | 0.019894 | 0.486787 | 0.592793 |
| RNF24 | 0.01992 | 0.486884 | 0.889624 |
| GM1568 | 0.019937 | 0.486884 | 1.178131 |
| CHEK2 | 0.019971 | 0.486884 | 0.58795 |
| BRMS1 | 0.019971 | 0.486884 | 1.661823 |
| 4933427G23RIK | 0.019983 | 0.486884 | 0.638755 |
| HTR1D | 0.019993 | 0.486884 | 1.165972 |
| TDP1 | 0.020026 | 0.487308 | 0.878836 |
| V1RE2 | 0.020077 | 0.487905 | 1.091441 |
| DDX55 | 0.020082 | 0.487905 | 1.091819 |
| C730027P07RIK | 0.020104 | 0.487925 | 1.16065 |
| RABAC1 | 0.020115 | 0.487925 | 1.49554 |
| LOC385878 | 0.020139 | 0.487945 | 0.927316 |
| 1810057B09RIK | 0.020147 | 0.487945 | 0.939414 |
| SPEER4F | 0.020219 | 0.489044 | 0.925155 |
| 4930547N16RIK | 0.020236 | 0.489044 | 0.267671 |
| GCH1 | 0.02024 | 0.489044 | 2.167151 |
| KIF20B | 0.020266 | 0.489208 | 0.264102 |
| NELF | 0.020279 | 0.489208 | 1.606587 |
| PORCN | 0.020311 | 0.489602 | 3.422065 |
| STAT1 | 0.020331 | 0.489706 | 1.169723 |
| 2300002D11RIK | 0.020425 | 0.490179 | 1.567973 |
| SMC2 | 0.020436 | 0.490179 | 0.86816 |
| RPS2 | 0.020478 | 0.490179 | 0.756109 |
| E230011J22RIK | 0.020485 | 0.490179 | 1.442829 |
| BC065085 | 0.020496 | 0.490179 | 1.441729 |
| ASNSD1 | 0.020497 | 0.490179 | 0.882927 |
| LOC195356 | 0.020509 | 0.490179 | 1.079602 |
| B3GALNT2 | 0.02051 | 0.490179 | 0.880178 |
| LOC386545 | 0.020544 | 0.490179 | 0.849332 |
| COX8C | 0.020572 | 0.490179 | 0.872564 |
| OMG | 0.020601 | 0.490179 | 0.903962 |
| TFDP2 | 0.020602 | 0.490179 | 0.559742 |
| TMEM178 | 0.020638 | 0.490179 | 1.547708 |
| CAMK2N1 | 0.02064 | 0.490179 | 2.195932 |
| FH1 | 0.020641 | 0.490179 | 0.538991 |
| LOC384717 | 0.020705 | 0.490179 | 1.116075 |
| GFPT1 | 0.020715 | 0.490179 | 1.187889 |
| LRRN3 | 0.02073 | 0.490179 | 1.229354 |
| CENPI | 0.020737 | 0.490179 | 0.35649 |
| SIAT7F | 0.020745 | 0.490179 | 1.300921 |
| BC003324 | 0.020755 | 0.490179 | 0.842043 |
| BRD9 | 0.02076 | 0.490179 | 1.646155 |
| OLFR373 | 0.020775 | 0.490179 | 0.768864 |
| IFRD1 | 0.0208 | 0.490179 | 0.643346 |
| BAHD1 | 0.020806 | 0.490179 | 1.503648 |
| LOC381576 | 0.020817 | 0.490179 | 0.970231 |
| TOR1AIP2 | 0.020825 | 0.490179 | 1.064739 |
| SHE | 0.020827 | 0.490179 | 0.934802 |
| LOC100046796 | 0.02083 | 0.490179 | 1.217425 |
| MTDNA_ND4L | 0.020832 | 0.490179 | 2.210951 |
| 4930483J18RIK | 0.020843 | 0.490179 | 1.143508 |
| IGHV1S21_K02154_IG_HEAVY_VARIABLE_1S21_81 | 0.020869 | 0.490349 | 0.913726 |
| 2410129H14RIK | 0.020884 | 0.490349 | 0.681633 |
| LOC381547 | 0.020898 | 0.490349 | 0.922337 |
| A330079A07RIK | 0.021004 | 0.491918 | 1.200693 |
| SAT2 | 0.021011 | 0.491918 | 1.227622 |
| FAM18A | 0.021018 | 0.491918 | 1.250207 |
| CPSF2 | 0.021037 | 0.491918 | 0.675737 |
| KLRA21 | 0.021063 | 0.491918 | 1.098549 |
| OLFR1508 | 0.021102 | 0.491918 | 1.065034 |
| SIRPA | 0.02114 | 0.491918 | 1.127478 |
| LOC637353 | 0.021147 | 0.491918 | 0.49476 |
| C1QTNF1 | 0.021147 | 0.491918 | 1.462159 |
| GCLC | 0.021148 | 0.491918 | 0.936359 |
| CIT | 0.021151 | 0.491918 | 0.665157 |
| HIGD2A | 0.021172 | 0.491918 | 1.224619 |
| PPP2R5B | 0.021178 | 0.491918 | 1.354506 |
| A430041B07RIK | 0.021188 | 0.491918 | 0.897987 |
| 2900024C23RIK | 0.021209 | 0.492019 | 1.2894 |
| LOC384422 | 0.021228 | 0.492019 | 1.096698 |
| DNAJB9 | 0.021267 | 0.492019 | 1.491848 |
| RPL4 | 0.021267 | 0.492019 | 0.545254 |
| SMC3 | 0.021272 | 0.492019 | 1.189042 |
| RCC2 | 0.021341 | 0.492799 | 0.720865 |
| ITIH5 | 0.021367 | 0.492799 | 1.370244 |
| GM288 | 0.02138 | 0.492799 | 0.653987 |
| APP | 0.021387 | 0.492799 | 1.760925 |
| 4930528H21RIK | 0.021413 | 0.492799 | 1.124825 |
| MRPS6 | 0.021419 | 0.492799 | 1.557969 |
| CST3 | 0.021444 | 0.492799 | 1.204416 |
| LOC333456 | 0.021447 | 0.492799 | 0.989794 |
| PHLDA2 | 0.02145 | 0.492799 | 0.298734 |
| 9030624J02RIK | 0.021476 | 0.493032 | 1.246889 |
| WBP5 | 0.021496 | 0.493135 | 1.520979 |
| PSPC1 | 0.021541 | 0.49343 | 1.051489 |
| SCL0001602.1_506 | 0.021541 | 0.49343 | 0.831123 |
| 2210414B05RIK | 0.021577 | 0.493683 | 0.907876 |
| GM757 | 0.021585 | 0.493683 | 0.928967 |
| B230312A22RIK | 0.021609 | 0.493683 | 2.414905 |
| NUP210 | 0.021643 | 0.493683 | 0.131984 |
| A330086O21RIK | 0.021653 | 0.493683 | 1.119225 |
| D15ERTD682E | 0.021668 | 0.493683 | 0.782123 |
| TMUB2 | 0.021729 | 0.493683 | 1.313789 |
| PIF1 | 0.021742 | 0.493683 | 0.570975 |
| 2810017I02RIK | 0.021773 | 0.493683 | 0.673632 |
| ZFP711 | 0.021776 | 0.493683 | 1.030326 |
| SSPO | 0.021776 | 0.493683 | 1.145015 |
| LOC100047194 | 0.021785 | 0.493683 | 0.932904 |
| LIMS2 | 0.021816 | 0.493683 | 3.866513 |
| BC005624 | 0.021822 | 0.493683 | 1.177886 |
| LOC381829 | 0.021826 | 0.493683 | 0.870068 |
| BC022623 | 0.02184 | 0.493683 | 1.225752 |
| LOC384344 | 0.021841 | 0.493683 | 0.89457 |
| CCDC101 | 0.021845 | 0.493683 | 0.54274 |
| ET-A, GPCR | 0.021856 | 0.493683 | 1.136344 |
| RNASEK | 0.021874 | 0.493727 | 1.610974 |
| IRF9 | 0.021961 | 0.494997 | 1.58458 |
| DNAJC19 | 0.021962 | 0.494997 | 0.573951 |
| VEGFA | 0.021981 | 0.49503 | 2.026327 |
| BC017612 | 0.022041 | 0.49503 | 0.541162 |
| DHRSX | 0.022068 | 0.49503 | 1.660403 |
| THRAP2 | 0.022076 | 0.49503 | 1.351161 |
| HAO3 | 0.022108 | 0.49503 | 0.899337 |
| NSUN4 | 0.022144 | 0.49503 | 1.424313 |
| CCNA2 | 0.022155 | 0.49503 | 0.579628 |
| ZSCAN10 | 0.022172 | 0.49503 | 0.888658 |
| 2610528E23RIK | 0.022238 | 0.49503 | 0.304849 |
| C230002E15RIK | 0.02224 | 0.49503 | 1.082625 |
| FBXO27 | 0.022266 | 0.49503 | 0.919614 |
| NPC2 | 0.022288 | 0.49503 | 1.428664 |
| 4930405H06RIK | 0.022289 | 0.49503 | 1.117881 |
| GM1006 | 0.022297 | 0.49503 | 1.094193 |
| ARPC1A | 0.022304 | 0.49503 | 1.199971 |
| PHAX | 0.022336 | 0.49503 | 0.75419 |
| GTF3A | 0.022344 | 0.49503 | 0.506453 |
| TAAR5 | 0.022354 | 0.49503 | 1.193887 |
| D630002J18RIK | 0.022386 | 0.49503 | 1.238133 |
| TAC1 | 0.022399 | 0.49503 | 1.054286 |
| TTC4 | 0.022406 | 0.49503 | 0.739267 |
| PI16 | 0.022417 | 0.49503 | 0.898672 |
| LOC100045621 | 0.022437 | 0.49503 | 0.938351 |
| RASSF7 | 0.022476 | 0.49503 | 1.312424 |
| LOC669001 | 0.0225 | 0.49503 | 1.17167 |
| LOC384104 | 0.022516 | 0.49503 | 0.75002 |
| ASPM | 0.022527 | 0.49503 | 0.344641 |
| B930059J09RIK | 0.022532 | 0.49503 | 1.339567 |
| RAD54L | 0.022536 | 0.49503 | 1.023043 |
| PTGER3 | 0.022546 | 0.49503 | 0.856703 |
| 4832420L08RIK | 0.022555 | 0.49503 | 1.182522 |
| CCDC43 | 0.022599 | 0.49503 | 0.686993 |
| ERMP1 | 0.022631 | 0.49503 | 0.513843 |
| ARD1 | 0.022652 | 0.49503 | 0.414775 |
| TRIM27 | 0.022675 | 0.49503 | 0.672451 |
| AQP5 | 0.022699 | 0.49503 | 2.247519 |
| BC048355 | 0.022716 | 0.49503 | 0.297446 |
| IFNA14 | 0.022716 | 0.49503 | 1.094521 |
| LYSMD2 | 0.022789 | 0.49503 | 0.693836 |
| ETAA1 | 0.022797 | 0.49503 | 0.681003 |
| EFEMP1 | 0.022802 | 0.49503 | 1.167751 |
| NFU1 | 0.022822 | 0.49503 | 0.583417 |
| A630057J21RIK | 0.02283 | 0.49503 | 1.121891 |
| FRS2 | 0.022859 | 0.49503 | 1.412515 |
| ZFP397OS | 0.022879 | 0.49503 | 0.891455 |
| CKAP5 | 0.022884 | 0.49503 | 0.468645 |
| 2700060E02RIK | 0.022902 | 0.49503 | 0.786399 |
| LOC100043906 | 0.022931 | 0.49503 | 0.410257 |
| EEF1E1 | 0.02294 | 0.49503 | 0.479477 |
| MRPL50 | 0.022977 | 0.49503 | 0.690797 |
| POLD1 | 0.022994 | 0.49503 | 0.331313 |
| 2410091N08RIK | 0.023007 | 0.49503 | 0.871416 |
| ZFP516 | 0.023016 | 0.49503 | 1.57218 |
| ELF2 | 0.023027 | 0.49503 | 0.831161 |
| BTBD11 | 0.023031 | 0.49503 | 1.096749 |
| C130057N11RIK | 0.023038 | 0.49503 | 1.185011 |
| TAL2 | 0.023042 | 0.49503 | 1.138368 |
| KRT75 | 0.023057 | 0.49503 | 1.137973 |
| C730036N12RIK | 0.023062 | 0.49503 | 0.801681 |
| IL23R | 0.023089 | 0.49503 | 1.113602 |
| LOC228107 | 0.023145 | 0.49503 | 1.073434 |
| JMJD1C | 0.023153 | 0.49503 | 0.76121 |
| TMEM132A | 0.023168 | 0.49503 | 1.435513 |
| NEUROD1 | 0.023172 | 0.49503 | 1.110698 |
| SPCS1 | 0.023174 | 0.49503 | 1.629015 |
| LOC100041725 | 0.02318 | 0.49503 | 1.396033 |
| BICC1 | 0.02319 | 0.49503 | 1.874709 |
| ANKS6 | 0.023193 | 0.49503 | 1.099134 |
| 1300018J18RIK | 0.0232 | 0.49503 | 0.84181 |
| LLGL1 | 0.023206 | 0.49503 | 1.525555 |
| SPHK2 | 0.02321 | 0.49503 | 1.751066 |
| CCT6B | 0.023217 | 0.49503 | 0.756529 |
| A230033A17RIK | 0.023243 | 0.49503 | 0.849901 |
| 2310007G05RIK | 0.023267 | 0.49503 | 1.613582 |
| CCDC28A | 0.023267 | 0.49503 | 0.83851 |
| E130301L11RIK | 0.023271 | 0.49503 | 0.63871 |
| GTLF3A | 0.023274 | 0.49503 | 0.715439 |
| MT3 | 0.023299 | 0.49503 | 1.148964 |
| EPN2 | 0.023301 | 0.49503 | 0.906304 |
| LOC100047260 | 0.023368 | 0.49503 | 1.295282 |
| NLRP14 | 0.023418 | 0.49503 | 1.061154 |
| DDX17 | 0.023419 | 0.49503 | 1.14618 |
| NRN1 | 0.023423 | 0.49503 | 2.351531 |
| 2010007L18RIK | 0.02343 | 0.49503 | 0.659008 |
| ARHGEF3 | 0.023443 | 0.49503 | 1.111648 |
| E430024F02RIK | 0.023459 | 0.49503 | 0.535157 |
| ENOPH1 | 0.023462 | 0.49503 | 0.543983 |
| LOC674706 | 0.023476 | 0.49503 | 1.355195 |
| 1110029L17RIK | 0.023483 | 0.49503 | 1.414965 |
| MED30 | 0.023517 | 0.49503 | 0.79132 |
| MRPS10 | 0.023537 | 0.49503 | 0.516092 |
| HIST1H2BH | 0.023593 | 0.49503 | 2.597157 |
| LOC100040755 | 0.023614 | 0.49503 | 0.952594 |
| 5830434K11RIK | 0.023617 | 0.49503 | 1.06238 |
| F730006M22RIK | 0.023641 | 0.49503 | 0.8523 |
| BCS1L | 0.023645 | 0.49503 | 0.55458 |
| SSTR5 | 0.023647 | 0.49503 | 0.949627 |
| ECM1 | 0.023659 | 0.49503 | 1.973741 |
| LOC384343 | 0.023679 | 0.49503 | 0.855555 |
| LOC385672 | 0.023679 | 0.49503 | 1.095787 |
| ASPHD1 | 0.023681 | 0.49503 | 0.924963 |
| CFLAR | 0.023717 | 0.49503 | 1.077384 |
| ZFP503 | 0.023773 | 0.49503 | 1.161106 |
| 4732481D19RIK | 0.023781 | 0.49503 | 1.190994 |
| AATF | 0.023781 | 0.49503 | 0.546603 |
| 9430015G10RIK | 0.023784 | 0.49503 | 0.585876 |
| ALPI | 0.023833 | 0.49503 | 0.874502 |
| D930014E17RIK | 0.02384 | 0.49503 | 1.140316 |
| ZFP330 | 0.023891 | 0.49503 | 0.827654 |
| CHCHD4 | 0.023903 | 0.49503 | 0.374153 |
| CALR3 | 0.023911 | 0.49503 | 0.766186 |
| LRP4 | 0.02393 | 0.49503 | 1.43134 |
| LOC100042270 | 0.02393 | 0.49503 | 1.113627 |
| TM7SF4 | 0.023947 | 0.49503 | 1.083475 |
| PYGO2 | 0.02396 | 0.49503 | 1.45599 |
| CDKL1 | 0.023962 | 0.49503 | 0.828018 |
| LOC386115 | 0.023963 | 0.49503 | 1.090306 |
| SLC36A1 | 0.023973 | 0.49503 | 1.736562 |
| PYCS | 0.023982 | 0.49503 | 0.943264 |
| BXDC2 | 0.023989 | 0.49503 | 0.623013 |
| E330023A07RIK | 0.024002 | 0.49503 | 0.901459 |
| TAF4A | 0.02403 | 0.49503 | 1.038331 |
| LOC381888 | 0.024044 | 0.49503 | 0.930407 |
| D130061E05RIK | 0.024059 | 0.49503 | 0.916051 |
| CRYAB | 0.024061 | 0.49503 | 2.969253 |
| LOC547329 | 0.024107 | 0.49503 | 1.199971 |
| LOC328136 | 0.024109 | 0.49503 | 0.968059 |
| LOC100043675 | 0.024132 | 0.49503 | 0.750418 |
| MMRN2 | 0.024135 | 0.49503 | 1.128 |
| INTS10 | 0.024148 | 0.49503 | 0.548767 |
| 5730412N02RIK | 0.024206 | 0.49503 | 0.913979 |
| PDCD2 | 0.024238 | 0.49503 | 0.6685 |
| OLFR1122 | 0.024252 | 0.49503 | 0.948487 |
| TRAPPC5 | 0.024256 | 0.49503 | 1.170236 |
| FGFR1OP | 0.024256 | 0.49503 | 0.509846 |
| TMSB4X | 0.024266 | 0.49503 | 2.013306 |
| LOC100046616 | 0.024268 | 0.49503 | 2.437947 |
| CALD1 | 0.02427 | 0.49503 | 0.819377 |
| B230304I02RIK | 0.024273 | 0.49503 | 0.804278 |
| DCPS | 0.024336 | 0.49503 | 0.664804 |
| DLST | 0.024337 | 0.49503 | 0.776846 |
| CCBL2 | 0.024353 | 0.49503 | 0.448537 |
| WDR81 | 0.024377 | 0.49503 | 0.907205 |
| DHRS13 | 0.024408 | 0.49503 | 0.711762 |
| CD82 | 0.024472 | 0.49503 | 1.598294 |
| ASNS | 0.024475 | 0.49503 | 0.26384 |
| C630002L24RIK | 0.024477 | 0.49503 | 0.879141 |
| ZFP703 | 0.024489 | 0.49503 | 1.536556 |
| ZFP281 | 0.024496 | 0.49503 | 1.846594 |
| RILPL1 | 0.024506 | 0.49503 | 1.457302 |
| LOC385953 | 0.024511 | 0.49503 | 0.821311 |
| BRRN1 | 0.024513 | 0.49503 | 0.210706 |
| MRPL47 | 0.024551 | 0.49503 | 0.467585 |
| 5330430O04RIK | 0.024579 | 0.49503 | 0.920847 |
| E030002K04RIK | 0.024603 | 0.49503 | 1.171724 |
| BC003498 | 0.024603 | 0.49503 | 1.310091 |
| DTD1 | 0.024618 | 0.49503 | 0.385802 |
| RNF14 | 0.02465 | 0.49503 | 1.215627 |
| BC024814 | 0.024654 | 0.49503 | 0.546389 |
| MAPK12 | 0.024682 | 0.49503 | 0.753528 |
| SCL0004029.1_17 | 0.024699 | 0.49503 | 0.949825 |
| MYT1L | 0.024728 | 0.49503 | 1.045167 |
| 2810410A03RIK | 0.024728 | 0.49503 | 1.393455 |
| PMP22 | 0.024729 | 0.49503 | 1.428367 |
| EG666756 | 0.02473 | 0.49503 | 0.528753 |
| 4632404M16RIK | 0.024773 | 0.49503 | 0.910796 |
| LOC384767 | 0.024784 | 0.49503 | 0.964376 |
| 1700029F09RIK | 0.024785 | 0.49503 | 0.564378 |
| CDCA5 | 0.024788 | 0.49503 | 0.468179 |
| EG545886 | 0.024821 | 0.49503 | 1.275003 |
| SLC19A2 | 0.024828 | 0.49503 | 0.703522 |
| C130020C13RIK | 0.024849 | 0.49503 | 0.64998 |
| CHCHD2 | 0.02486 | 0.49503 | 0.81244 |
| SCL0001489.1_43 | 0.024917 | 0.49503 | 0.968887 |
| PPAPDC1 | 0.024955 | 0.49503 | 1.130635 |
| SMAP2 | 0.024957 | 0.49503 | 1.373319 |
| MRPL18 | 0.024963 | 0.49503 | 0.692891 |
| SLC26A6 | 0.024974 | 0.49503 | 1.475769 |
| GPR64, GPCR | 0.02499 | 0.49503 | 0.905864 |
| VMN2R28 | 0.025006 | 0.49503 | 1.04425 |
| HDHD1A | 0.02502 | 0.49503 | 1.069819 |
| LIN54 | 0.025025 | 0.49503 | 0.547223 |
| D230010H24RIK | 0.025031 | 0.49503 | 0.780173 |
| SEPN1 | 0.025032 | 0.49503 | 1.705821 |
| CLIC1 | 0.025054 | 0.49503 | 1.566994 |
| ZNFX1 | 0.025057 | 0.49503 | 1.876616 |
| AIPL1 | 0.025111 | 0.49503 | 0.774623 |
| COL14A1 | 0.025111 | 0.49503 | 1.492675 |
| SHROOM3 | 0.02513 | 0.49503 | 2.357406 |
| QRSL1 | 0.025181 | 0.49503 | 0.714184 |
| ALDH8A1 | 0.02521 | 0.49503 | 1.187203 |
| NUP88 | 0.025217 | 0.49503 | 0.627926 |
| FBXL6 | 0.025224 | 0.49503 | 0.631651 |
| SCL000981.1_40 | 0.025235 | 0.49503 | 1.648896 |
| KARS-PS1 | 0.025258 | 0.49503 | 0.965981 |
| CX3CL1 | 0.025266 | 0.49503 | 1.785919 |
| 4933427E13RIK | 0.025283 | 0.49503 | 0.863898 |
| LOC213332 | 0.025292 | 0.49503 | 0.893063 |
| 1110059E24RIK | 0.025326 | 0.49503 | 0.845767 |
| RAPGEF3 | 0.025358 | 0.49503 | 1.744403 |
| LOC386068 | 0.025364 | 0.49503 | 1.071452 |
| 4930569O18RIK | 0.025367 | 0.49503 | 0.917216 |
| NUP43 | 0.025373 | 0.49503 | 0.401341 |
| 4933434I06RIK | 0.025386 | 0.49503 | 0.862901 |
| DHX57 | 0.025389 | 0.49503 | 1.277597 |
| CD164L2 | 0.025393 | 0.49503 | 1.159417 |
| 2610019N13RIK | 0.025397 | 0.49503 | 0.905132 |
| A730016J02RIK | 0.025414 | 0.49503 | 0.882846 |
| 9130422G05RIK | 0.02542 | 0.49503 | 1.607962 |
| LOC100044292 | 0.025455 | 0.49503 | 1.21341 |
| CUL7 | 0.025478 | 0.49503 | 1.456999 |
| RAD51AP1 | 0.025486 | 0.49503 | 0.510482 |
| C330049H01RIK | 0.025494 | 0.49503 | 1.455451 |
| ASB15 | 0.025521 | 0.49503 | 0.934068 |
| SCAMP1 | 0.025523 | 0.49503 | 1.380317 |
| CCL9 | 0.025533 | 0.49503 | 2.379569 |
| PMAIP1 | 0.025538 | 0.49503 | 0.905529 |
| MTDNA_COXIII | 0.025541 | 0.49503 | 1.258175 |
| OLFR568 | 0.025564 | 0.49503 | 0.842472 |
| THYN1 | 0.025626 | 0.49503 | 0.479854 |
| SEMA3B | 0.025636 | 0.49503 | 1.145042 |
| NOL14 | 0.025659 | 0.49503 | 0.463626 |
| SUPV3L1 | 0.025681 | 0.49503 | 0.643851 |
| MAP3K3 | 0.025697 | 0.49503 | 1.278129 |
| ZFP64 | 0.025718 | 0.49503 | 0.835609 |
| LOC232887 | 0.025718 | 0.49503 | 0.371697 |
| LOC381226 | 0.025721 | 0.49503 | 1.192426 |
| XYLT2 | 0.025764 | 0.49503 | 1.550607 |
| LOC238316 | 0.025766 | 0.49503 | 1.108724 |
| 1110059G02RIK | 0.025779 | 0.49503 | 0.911133 |
| GEMIN5 | 0.025785 | 0.49503 | 0.626998 |
| SYNCRIP | 0.02579 | 0.49503 | 0.544561 |
| AKR1A4 | 0.025792 | 0.49503 | 1.317984 |
| 4833423E24RIK | 0.025798 | 0.49503 | 0.853325 |
| DENND5A | 0.025801 | 0.49503 | 1.585348 |
| E130319J22RIK | 0.025832 | 0.49503 | 1.048408 |
| ACOT5 | 0.02585 | 0.49503 | 1.087262 |
| HES6 | 0.025944 | 0.49503 | 1.932274 |
| BANF1 | 0.025954 | 0.49503 | 0.596875 |
| A730006N07RIK | 0.025965 | 0.49503 | 0.871275 |
| CACNG8 | 0.025966 | 0.49503 | 1.07731 |
| LOC676974 | 0.025981 | 0.49503 | 1.326477 |
| 2210016L21RIK | 0.025999 | 0.49503 | 1.430844 |
| LOC327803 | 0.026006 | 0.49503 | 1.265406 |
| LRRC23 | 0.026025 | 0.49503 | 1.165783 |
| GHSR | 0.026053 | 0.49503 | 0.834143 |
| E330009F12RIK | 0.026053 | 0.49503 | 1.234135 |
| BATF2 | 0.026058 | 0.49503 | 1.118553 |
| MCPT9 | 0.026082 | 0.49503 | 0.914824 |
| LOC667388 | 0.026095 | 0.49503 | 1.231571 |
| LOC230628 | 0.02611 | 0.49503 | 1.216947 |
| D19ERTD386E | 0.02613 | 0.49503 | 0.900459 |
| JAG2 | 0.026149 | 0.49503 | 0.697823 |
| BCAP29 | 0.026151 | 0.49503 | 0.649424 |
| HIST1H2BF | 0.026193 | 0.49503 | 3.21912 |
| SLITRK4 | 0.026201 | 0.49503 | 0.836691 |
| BCL3 | 0.026205 | 0.49503 | 1.379552 |
| 4933401B08RIK | 0.026214 | 0.49503 | 0.674364 |
| 2010110K16RIK | 0.026246 | 0.49503 | 1.162072 |
| LINGO1 | 0.026255 | 0.49503 | 0.76842 |
| CD207 | 0.026258 | 0.49503 | 0.964442 |
| LOC100044177 | 0.026263 | 0.49503 | 0.814131 |
| C920005C14RIK | 0.026272 | 0.49503 | 1.180092 |
| MBIP | 0.026273 | 0.49503 | 1.389244 |
| CSPG5 | 0.02634 | 0.49503 | 1.066684 |
| KIF11 | 0.026361 | 0.49503 | 0.271332 |
| MRPS28 | 0.026374 | 0.49503 | 0.49642 |
| MFSD7A | 0.026382 | 0.49503 | 1.10962 |
| RNF219 | 0.026383 | 0.49503 | 0.679871 |
| RBM5 | 0.026385 | 0.49503 | 1.668633 |
| LOC100040538 | 0.026393 | 0.49503 | 1.139842 |
| SHMT1 | 0.026407 | 0.49503 | 0.524495 |
| KCTD9 | 0.026415 | 0.49503 | 0.863619 |
| MT-ND5 | 0.026416 | 0.49503 | 2.419596 |
| TRAF4 | 0.026423 | 0.49503 | 1.41526 |
| 9030404E10RIK | 0.02644 | 0.49503 | 0.973464 |
| 2410018M08RIK | 0.02648 | 0.49503 | 0.565579 |
| 2610528B01RIK | 0.026482 | 0.49503 | 0.684205 |
| D130005J21RIK | 0.026494 | 0.49503 | 0.895294 |
| EED | 0.026521 | 0.49503 | 0.73455 |
| MUSTN1 | 0.026551 | 0.49503 | 2.848627 |
| AKR1B3 | 0.026555 | 0.49503 | 0.544587 |
| CENPN | 0.026562 | 0.49503 | 0.363619 |
| LOC385251 | 0.026563 | 0.49503 | 0.744116 |
| SLC25A26 | 0.026588 | 0.49503 | 0.778733 |
| TULP4 | 0.026617 | 0.49503 | 1.882957 |
| PDE6H | 0.026642 | 0.49503 | 1.099413 |
| ZFP608 | 0.026648 | 0.49503 | 1.81018 |
| PLA2G15 | 0.026667 | 0.49503 | 2.026047 |
| B9D1 | 0.026684 | 0.49503 | 0.939957 |
| PLUNC | 0.026686 | 0.49503 | 0.855002 |
| 1110012L19RIK | 0.026707 | 0.49503 | 1.24137 |
| SNUPN | 0.026712 | 0.49503 | 0.927252 |
| 3010026O09RIK | 0.02673 | 0.49503 | 0.483906 |
| 1110003O08RIK | 0.02674 | 0.49503 | 1.42685 |
| EG434181 | 0.026748 | 0.49503 | 1.52986 |
| OLFR1433 | 0.026767 | 0.49503 | 0.86304 |
| A530075A22RIK | 0.026792 | 0.49503 | 0.876302 |
| EGLN3 | 0.026807 | 0.49503 | 1.851593 |
| IMPA1 | 0.026828 | 0.49503 | 0.832179 |
| 4933427L07RIK | 0.026853 | 0.49503 | 1.053848 |
| HIST1H2BK | 0.026862 | 0.49503 | 3.0947 |
| LBP | 0.026866 | 0.49503 | 2.726909 |
| ESPL1 | 0.026877 | 0.49503 | 0.435708 |
| E230026C15RIK | 0.02688 | 0.49503 | 0.901438 |
| PMF1 | 0.026881 | 0.49503 | 0.429164 |
| SERPINA1D | 0.026913 | 0.49503 | 1.13658 |
| 2310005L22RIK | 0.026926 | 0.49503 | 0.519058 |
| TTK | 0.026929 | 0.49503 | 0.693451 |
| PCGF6 | 0.026969 | 0.49503 | 0.667404 |
| ALG6 | 0.02697 | 0.49503 | 0.596489 |
| C130073O12RIK | 0.026985 | 0.49503 | 0.644908 |
| ANP32E | 0.026986 | 0.49503 | 0.483828 |
| PNLIPRP2 | 0.027072 | 0.496324 | 0.905467 |
| LOC100047651 | 0.027099 | 0.496417 | 1.907038 |
| IMPDH1 | 0.02712 | 0.496417 | 0.826126 |
| LOC384943 | 0.027126 | 0.496417 | 1.280079 |
| 4930597O21RIK | 0.027163 | 0.496417 | 0.870088 |
| TMEM46 | 0.027195 | 0.496417 | 0.901605 |
| LOC384864 | 0.027218 | 0.496417 | 1.108058 |
| 4930511J15RIK | 0.027226 | 0.496417 | 1.119664 |
| SLC6A12 | 0.027243 | 0.496417 | 1.321308 |
| C230090G04RIK | 0.027281 | 0.496417 | 0.953827 |
| YBX3 | 0.027294 | 0.496417 | 2.113548 |
| ZBTB24 | 0.027307 | 0.496417 | 1.396549 |
| 2310021P13RIK | 0.027307 | 0.496417 | 2.00996 |
| SOX10 | 0.02733 | 0.496417 | 1.019221 |
| GOT2 | 0.027343 | 0.496417 | 0.710875 |
| UBE2L6 | 0.027351 | 0.496417 | 1.906686 |
| DCPP1 | 0.027358 | 0.496417 | 0.809966 |
| CDKN2AIPNL | 0.027363 | 0.496417 | 1.320728 |
| 2310040C09RIK | 0.02737 | 0.496417 | 1.173621 |
| ZFAND2A | 0.027399 | 0.496417 | 1.343813 |
| PKD1 | 0.027446 | 0.496417 | 1.486308 |
| LOC100046232 | 0.027484 | 0.496417 | 0.850078 |
| PCNA | 0.027495 | 0.496417 | 0.690047 |
| LSM3 | 0.027523 | 0.496417 | 0.557548 |
| 2210408F21RIK | 0.02755 | 0.496417 | 1.469169 |
| LOC236170 | 0.027551 | 0.496417 | 1.062159 |
| LOC384865 | 0.027568 | 0.496417 | 0.821595 |
| DDX1 | 0.027572 | 0.496417 | 0.748289 |
| LOC384196 | 0.02761 | 0.496417 | 1.108109 |
| LOC385679 | 0.027619 | 0.496417 | 0.889603 |
| PEPD | 0.027637 | 0.496417 | 1.358894 |
| B930008A12RIK | 0.027647 | 0.496417 | 0.654183 |
| BSDC1 | 0.027673 | 0.496417 | 1.614403 |
| IIGP2 | 0.027699 | 0.496417 | 1.986689 |
| KNSL5 | 0.02772 | 0.496417 | 2.237157 |
| RHOIP3-PENDING | 0.027733 | 0.496417 | 1.091087 |
| CD300LG | 0.027736 | 0.496417 | 1.19397 |
| ZFP354A | 0.027764 | 0.496417 | 0.842316 |
| LRRC37A | 0.027768 | 0.496417 | 1.153726 |
| UBB | 0.027772 | 0.496417 | 1.151621 |
| TTC17 | 0.027799 | 0.496417 | 1.459054 |
| D330028K02RIK | 0.027804 | 0.496417 | 0.915332 |
| OLFR910 | 0.027841 | 0.496417 | 1.232881 |
| LOC381875 | 0.027844 | 0.496417 | 1.134822 |
| A730071D19RIK | 0.027867 | 0.496417 | 0.829837 |
| PDE3A | 0.02787 | 0.496417 | 1.087589 |
| UQCRC1 | 0.027881 | 0.496417 | 0.6846 |
| WBP1 | 0.027893 | 0.496417 | 1.532089 |
| 4732462B05RIK | 0.027893 | 0.496417 | 0.928238 |
| LOC666403 | 0.027934 | 0.496417 | 0.859756 |
| HMG20B | 0.027936 | 0.496417 | 1.376496 |
| 3632431M01RIK | 0.027943 | 0.496417 | 0.865137 |
| TRIAP1 | 0.027944 | 0.496417 | 0.862941 |
| UPRT | 0.027964 | 0.496417 | 0.753685 |
| ORM1 | 0.027987 | 0.496417 | 0.537809 |
| LOC269515 | 0.027989 | 0.496417 | 0.461979 |
| NUDEL-PENDING | 0.028051 | 0.496417 | 1.27512 |
| 4932442C03RIK | 0.028071 | 0.496417 | 0.809349 |
| CD151 | 0.028116 | 0.496417 | 1.464424 |
| 6330503H08RIK | 0.028168 | 0.496417 | 1.177886 |
| IHH | 0.028175 | 0.496417 | 0.821804 |
| ATP6V1C1 | 0.028182 | 0.496417 | 1.326906 |
| A430088A22RIK | 0.028189 | 0.496417 | 0.875938 |
| MAP3K6 | 0.028197 | 0.496417 | 1.364053 |
| DORZ1 | 0.028291 | 0.496417 | 0.518087 |
| NKX6-3 | 0.028317 | 0.496417 | 1.205195 |
| CHMP4B | 0.028345 | 0.496417 | 1.409321 |
| SLC25A39 | 0.028355 | 0.496417 | 0.596213 |
| PCSK7 | 0.028362 | 0.496417 | 1.50549 |
| LOC100038857 | 0.02837 | 0.496417 | 0.847156 |
| CCDC99 | 0.028375 | 0.496417 | 0.446851 |
| SNRPA | 0.028378 | 0.496417 | 0.688725 |
| SDHA | 0.028399 | 0.496417 | 0.967477 |
| 2900006F19RIK | 0.028411 | 0.496417 | 1.079627 |
| CKAP2L | 0.028431 | 0.496417 | 0.27191 |
| TNIP1 | 0.028447 | 0.496417 | 1.833076 |
| RND3 | 0.02845 | 0.496417 | 1.220242 |
| C130038I05RIK | 0.02847 | 0.496417 | 0.889952 |
| LOC382024 | 0.028496 | 0.496417 | 0.767302 |
| 8430438L13RIK | 0.028498 | 0.496417 | 1.155539 |
| SKI | 0.028507 | 0.496417 | 1.730553 |
| CDC42BPB | 0.028528 | 0.496417 | 2.129035 |
| 4933409L06RIK | 0.028528 | 0.496417 | 1.126385 |
| LOC100043209 | 0.028533 | 0.496417 | 0.954401 |
| CN1A | 0.028551 | 0.496417 | 1.07066 |
| ZDHHC4 | 0.028555 | 0.496417 | 1.443162 |
| NFATC1 | 0.028565 | 0.496417 | 1.711941 |
| LOC624610 | 0.028571 | 0.496417 | 0.78462 |
| 4930451C15RIK | 0.028572 | 0.496417 | 1.204611 |
| SNRPG | 0.028584 | 0.496417 | 0.862343 |
| UBE2E1 | 0.028637 | 0.496417 | 0.420983 |
| DHRS2 | 0.028644 | 0.496417 | 1.128652 |
| ZFP715 | 0.028665 | 0.496417 | 1.212317 |
| PHPT1 | 0.028666 | 0.496417 | 1.181265 |
| LOC100044221 | 0.028671 | 0.496417 | 0.912418 |
| CAP2 | 0.028673 | 0.496417 | 1.196317 |
| AA619741 | 0.028691 | 0.496417 | 1.081275 |
| ZBTB40 | 0.028719 | 0.496417 | 0.845983 |
| ARHGEF19 | 0.028722 | 0.496417 | 2.255426 |
| ANGPT2 | 0.028727 | 0.496417 | 1.837571 |
| E430003D02RIK | 0.028738 | 0.496417 | 0.271238 |
| LOC386054 | 0.02874 | 0.496417 | 0.944878 |
| C730046C01RIK | 0.028745 | 0.496417 | 1.328502 |
| A830026B15RIK | 0.028779 | 0.496417 | 0.717558 |
| DISP1 | 0.028844 | 0.496417 | 1.408767 |
| TCP10A | 0.028846 | 0.496417 | 1.275533 |
| C230098K08RIK | 0.028856 | 0.496417 | 0.82666 |
| PMS2 | 0.028874 | 0.496417 | 0.759032 |
| OLFR1020 | 0.0289 | 0.496417 | 1.153805 |
| PSCD1 | 0.028904 | 0.496417 | 1.339289 |
| TIMM8A1 | 0.028933 | 0.496417 | 0.478393 |
| EIF3D | 0.028944 | 0.496417 | 0.803108 |
| 2310003P10RIK | 0.028947 | 0.496417 | 1.374049 |
| E030011O05RIK | 0.02896 | 0.496417 | 1.146047 |
| LOC330870 | 0.028964 | 0.496417 | 0.887078 |
| SCGB3A2 | 0.028969 | 0.496417 | 1.103 |
| MASTL | 0.028971 | 0.496417 | 0.827138 |
| BC062115 | 0.028976 | 0.496417 | 0.893785 |
| ASAH1 | 0.029009 | 0.496417 | 1.290264 |
| CDKN2C | 0.029022 | 0.496417 | 2.043062 |
| OXSR1 | 0.029023 | 0.496417 | 0.868943 |
| ATXN1 | 0.029024 | 0.496417 | 1.738609 |
| SUV39H1 | 0.029065 | 0.496523 | 0.571239 |
| GRAMD1A | 0.029066 | 0.496523 | 1.034524 |
| NCAN | 0.029079 | 0.496523 | 0.947589 |
| ERBB2 | 0.029147 | 0.497245 | 1.50153 |
| C1QBP | 0.029153 | 0.497245 | 0.403041 |
| CXCL9 | 0.029183 | 0.497312 | 1.0808 |
| LOC100044204 | 0.029193 | 0.497312 | 1.748963 |
| 2010001F03RIK | 0.029205 | 0.497312 | 0.669196 |
| RRAGC | 0.029237 | 0.49749 | 1.220664 |
| CISD1 | 0.029249 | 0.49749 | 0.607673 |
| LOC100046278 | 0.029268 | 0.49749 | 1.044708 |
| 2610021K21RIK | 0.029301 | 0.49749 | 0.419788 |
| SSTR1 | 0.029333 | 0.49749 | 0.911765 |
| 1700022P15RIK | 0.029333 | 0.49749 | 0.937809 |
| CD9 | 0.029366 | 0.49749 | 1.420435 |
| SPATA2 | 0.029384 | 0.49749 | 0.89608 |
| IFNGR1 | 0.029402 | 0.49749 | 1.717726 |
| NDUFV2 | 0.029406 | 0.49749 | 0.570909 |
| D2ERTD391E | 0.029442 | 0.49749 | 1.400751 |
| PDLIM1 | 0.029448 | 0.49749 | 0.655499 |
| LOC230602 | 0.029468 | 0.49749 | 1.039988 |
| OLFR945 | 0.029499 | 0.49749 | 0.849469 |
| TPM3 | 0.029518 | 0.49749 | 0.853108 |
| TAF140 | 0.029518 | 0.49749 | 0.772603 |
| P38IP-PENDING | 0.029547 | 0.49749 | 0.850647 |
| SPRR2K | 0.029552 | 0.49749 | 1.297918 |
| E2F5 | 0.029567 | 0.49749 | 0.688884 |
| SIRT5 | 0.02965 | 0.49749 | 0.723651 |
| ANKRD24 | 0.029677 | 0.49749 | 0.71321 |
| DEPDC7 | 0.029681 | 0.49749 | 0.858605 |
| CYP1B1 | 0.029708 | 0.49749 | 1.831891 |
| GDPD5 | 0.029752 | 0.49749 | 1.743879 |
| A930026I22RIK | 0.029787 | 0.49749 | 2.631709 |
| NUDT5 | 0.029788 | 0.49749 | 0.684663 |
| LOC100043313 | 0.029815 | 0.49749 | 0.650626 |
| NPM1 | 0.029861 | 0.49749 | 0.624021 |
| AIM1L | 0.029896 | 0.49749 | 0.756791 |
| 6330403K07RIK | 0.029898 | 0.49749 | 1.1304 |
| 4931426K16RIK | 0.029901 | 0.49749 | 1.494884 |
| CYP2D10 | 0.029908 | 0.49749 | 1.278838 |
| C330006A16RIK | 0.029912 | 0.49749 | 0.754888 |
| EG668761 | 0.029936 | 0.49749 | 1.053945 |
| PCCA | 0.029949 | 0.49749 | 0.439154 |
| ERGIC3 | 0.029972 | 0.49749 | 1.412255 |
| STRA6 | 0.030001 | 0.49749 | 1.341859 |
| LOC240066 | 0.030011 | 0.49749 | 0.915649 |
| SPCS3 | 0.030013 | 0.49749 | 1.391138 |
| ENSMUSG00000045946 | 0.03005 | 0.49749 | 1.13189 |
| GGT1 | 0.030073 | 0.49749 | 1.366861 |
| NARS2 | 0.030074 | 0.49749 | 0.682594 |
| 9430048B09RIK | 0.030084 | 0.49749 | 1.136686 |
| 2210023G05RIK | 0.030086 | 0.49749 | 0.835358 |
| S100A11 | 0.030087 | 0.49749 | 2.11648 |
| FOLH1 | 0.030108 | 0.49749 | 0.865037 |
| 8430421H08RIK | 0.030137 | 0.49749 | 0.932774 |
| B230365C01RIK | 0.030147 | 0.49749 | 0.797469 |
| WIPI1 | 0.030154 | 0.49749 | 1.718163 |
| ARL8A | 0.030159 | 0.49749 | 1.3463 |
| PLTP | 0.030199 | 0.49749 | 0.751668 |
| LOC383279 | 0.030204 | 0.49749 | 0.857792 |
| D130026O08RIK | 0.030215 | 0.49749 | 0.921741 |
| PPP5C | 0.030235 | 0.49749 | 0.527691 |
| TRIM3 | 0.03024 | 0.49749 | 1.578259 |
| OTTMUSG00000001070 | 0.030262 | 0.49749 | 1.935983 |
| LOC329139 | 0.030273 | 0.49749 | 0.915247 |
| SLC12A6 | 0.030274 | 0.49749 | 1.527035 |
| AYTL1B | 0.030293 | 0.49749 | 1.087714 |
| SORCS3 | 0.030317 | 0.49749 | 0.867879 |
| HOOK3 | 0.030326 | 0.49749 | 1.375669 |
| DIS3L2 | 0.03034 | 0.49749 | 0.679965 |
| MAD2L1 | 0.030375 | 0.49749 | 0.339912 |
| SSH1 | 0.0304 | 0.49749 | 1.343752 |
| PRODH | 0.0304 | 0.49749 | 0.332287 |
| DTX3L | 0.030402 | 0.49749 | 1.325129 |
| 4931422A14RIK | 0.030403 | 0.49749 | 1.132073 |
| D230044L08RIK | 0.030412 | 0.49749 | 0.935667 |
| ODC1 | 0.030437 | 0.49749 | 0.529609 |
| 2410157M17RIK | 0.030438 | 0.49749 | 1.112753 |
| FURIN | 0.030439 | 0.49749 | 1.69729 |
| RIN1 | 0.030461 | 0.49749 | 0.928688 |
| CD99L2 | 0.030463 | 0.49749 | 1.218748 |
| RP1H | 0.030499 | 0.49749 | 0.913346 |
| AK2 | 0.030506 | 0.49749 | 0.591616 |
| ENSMUSG00000044227 | 0.030509 | 0.49749 | 1.157356 |
| LOC100045359 | 0.030516 | 0.49749 | 2.081273 |
| OLFR538 | 0.030521 | 0.49749 | 1.124487 |
| 3930401K13RIK | 0.030566 | 0.49749 | 1.358894 |
| 1110002N22RIK | 0.03057 | 0.49749 | 0.840585 |
| DAZAP1 | 0.030598 | 0.49749 | 0.55711 |
| CCAR1 | 0.030612 | 0.49749 | 0.628681 |
| TTF2 | 0.030618 | 0.49749 | 0.437989 |
| SMARCA5 | 0.030622 | 0.49749 | 0.832872 |
| IMP3 | 0.030667 | 0.49749 | 0.722632 |
| TBC1D5 | 0.030674 | 0.49749 | 1.147558 |
| LOC100044190 | 0.030679 | 0.49749 | 1.28073 |
| LOC381983 | 0.030694 | 0.49749 | 0.942305 |
| 4933426M11RIK | 0.030696 | 0.49749 | 2.56976 |
| TTC39B | 0.0307 | 0.49749 | 1.562367 |
| HIST2H2AA2 | 0.030702 | 0.49749 | 4.75419 |
| LOC234987 | 0.030714 | 0.49749 | 1.583371 |
| HPBRII-4 | 0.030732 | 0.49749 | 1.092475 |
| IGKV8-31_AJ235957_IG_KAPPA_VARIABLE_8-31_3 | 0.030744 | 0.49749 | 1.08225 |
| CD80 | 0.030766 | 0.49749 | 1.498341 |
| CEP63 | 0.030769 | 0.49749 | 0.889151 |
| FER1L3 | 0.03078 | 0.49749 | 1.820499 |
| BRD8 | 0.030839 | 0.497645 | 0.811615 |
| LOC382418 | 0.030875 | 0.497645 | 1.082 |
| LOC330264 | 0.030902 | 0.497645 | 1.150691 |
| MRE11A | 0.030911 | 0.497645 | 0.452179 |
| SLC44A2 | 0.030933 | 0.497645 | 1.653283 |
| MYO18A | 0.03097 | 0.497645 | 1.516662 |
| ZHX2 | 0.030985 | 0.497645 | 1.095964 |
| LOC385254 | 0.030992 | 0.497645 | 1.054774 |
| EG625540 | 0.031011 | 0.497645 | 0.941348 |
| CHI3L1 | 0.031034 | 0.497645 | 1.366608 |
| AL022832 | 0.031039 | 0.497645 | 0.792418 |
| PET112L | 0.031054 | 0.497645 | 0.766292 |
| MPV17 | 0.03107 | 0.497645 | 1.365977 |
| 5730588I11RIK | 0.031071 | 0.497645 | 0.402176 |
| LOC665051 | 0.031081 | 0.497645 | 1.057213 |
| SCL0002702.1_3805 | 0.031082 | 0.497645 | 1.139262 |
| OLFR97 | 0.031083 | 0.497645 | 0.904421 |
| HIST1H2BM | 0.031095 | 0.497645 | 2.881593 |
| LOC381790 | 0.031096 | 0.497645 | 0.816599 |
| SCL000205.1_10 | 0.031125 | 0.497852 | 0.560013 |
| MXD3 | 0.031143 | 0.497881 | 0.538008 |
| LOC665393 | 0.031182 | 0.498185 | 0.909051 |
| EIF4E | 0.031204 | 0.498185 | 0.77066 |
| D16ERTD472E | 0.031213 | 0.498185 | 0.713458 |
| D12ERTD553E | 0.031228 | 0.498185 | 1.069176 |
| PELI1 | 0.031263 | 0.498185 | 1.13821 |
| A030010B06RIK | 0.031269 | 0.498185 | 1.200249 |
| LOC385241 | 0.031304 | 0.498185 | 1.029374 |
| ICA1 | 0.03131 | 0.498185 | 1.089148 |
| 1810033M07RIK | 0.031314 | 0.498185 | 0.910291 |
| A330080J22RIK | 0.031328 | 0.498185 | 0.536692 |
| QSOX1 | 0.031339 | 0.498185 | 2.199639 |
| SCL0004025.1_69 | 0.031364 | 0.498321 | 0.660563 |
| ZSWIM4 | 0.031463 | 0.499643 | 1.432797 |
| LOC100045542 | 0.031499 | 0.49978 | 2.100113 |
| 9230116M18RIK | 0.031505 | 0.49978 | 1.040444 |
| GNA15 | 0.03153 | 0.499849 | 1.183205 |
| TMEM131 | 0.031547 | 0.499849 | 1.289936 |
| PTGER4, FP4, GPCR | 0.031557 | 0.499849 | 1.115301 |
| RENBP | 0.031577 | 0.499894 | 1.539434 |
| LY9 | 0.031638 | 0.500324 | 1.114709 |
| TTYH3 | 0.031644 | 0.500324 | 1.674271 |
| 2310046O06RIK | 0.03167 | 0.500324 | 0.923553 |
| POLD2 | 0.031673 | 0.500324 | 0.442076 |
| ARID3B | 0.031693 | 0.500324 | 1.175195 |
| 1700021E15RIK | 0.031706 | 0.500324 | 0.789001 |
| RPS15A | 0.031717 | 0.500324 | 0.718221 |
| CRYBB1 | 0.031783 | 0.500946 | 0.91603 |
| EBF3 | 0.031789 | 0.500946 | 0.643227 |
| 2610510J17RIK | 0.031827 | 0.501072 | 0.273871 |
| S1PR1 | 0.031849 | 0.501072 | 0.927059 |
| PPP2CA | 0.031867 | 0.501072 | 0.852044 |
| 2010317E24RIK | 0.031882 | 0.501072 | 0.208131 |
| RAPGEFL1 | 0.031883 | 0.501072 | 1.36563 |
| CPLX1 | 0.031895 | 0.501072 | 1.108109 |
| RILP | 0.031926 | 0.501237 | 1.646915 |
| 1700123O12RIK | 0.031945 | 0.501237 | 1.121244 |
| SFXN3 | 0.031964 | 0.501237 | 1.886702 |
| ECSIT | 0.03197 | 0.501237 | 0.514497 |
| 4933433P14RIK | 0.032059 | 0.502374 | 1.19629 |
| ZFAND3 | 0.032138 | 0.503245 | 1.641483 |
| PDSS2 | 0.032147 | 0.503245 | 0.617866 |
| OLFR207 | 0.032172 | 0.50337 | 0.826718 |
| HSP90AB1 | 0.032188 | 0.50337 | 0.678773 |
| SETD1B | 0.032213 | 0.503509 | 1.462632 |
| PRDX3 | 0.032256 | 0.503926 | 0.620456 |
| 4930448N21RIK | 0.03228 | 0.504042 | 0.821614 |
| BC004701 | 0.032323 | 0.504143 | 0.599556 |
| 2810407L07RIK | 0.032324 | 0.504143 | 0.944463 |
| IFITM2 | 0.03238 | 0.504143 | 1.324395 |
| SMOC1 | 0.032418 | 0.504143 | 2.622179 |
| MCM7 | 0.032441 | 0.504143 | 0.360682 |
| SLC7A2 | 0.032463 | 0.504143 | 1.190224 |
| CENPB | 0.032464 | 0.504143 | 1.303448 |
| 3110013H01RIK | 0.032471 | 0.504143 | 0.54274 |
| GREM1 | 0.032471 | 0.504143 | 1.743476 |
| AA407270 | 0.032474 | 0.504143 | 1.49133 |
| NEDD9 | 0.032495 | 0.504143 | 2.311239 |
| PSAT1 | 0.0325 | 0.504143 | 0.209323 |
| NOL9 | 0.032502 | 0.504143 | 0.622322 |
| 9130213B05RIK | 0.032548 | 0.504143 | 2.614134 |
| CDC20 | 0.032558 | 0.504143 | 0.24363 |
| PPM1F | 0.032573 | 0.504143 | 0.713128 |
| RAI16 | 0.032594 | 0.504143 | 1.50049 |
| LOC277856 | 0.032597 | 0.504143 | 0.492092 |
| PIP5K1A | 0.032611 | 0.504143 | 1.254402 |
| ERO1LB | 0.032623 | 0.504143 | 0.869344 |
| GM767 | 0.032632 | 0.504143 | 0.903732 |
| EG435970 | 0.032646 | 0.504143 | 0.969066 |
| RNF181 | 0.032691 | 0.504388 | 1.205808 |
| SLIT1 | 0.032704 | 0.504388 | 0.840081 |
| SCL0001883.1_112 | 0.03271 | 0.504388 | 0.87527 |
| FBS1 | 0.032735 | 0.504512 | 1.394905 |
| TGFB3 | 0.032809 | 0.504888 | 1.354225 |
| LOC233227 | 0.032847 | 0.504888 | 0.81794 |
| DMRTC2 | 0.032872 | 0.504888 | 0.830585 |
| RBM12B | 0.032898 | 0.504888 | 1.169615 |
| MS4A4C | 0.032899 | 0.504888 | 0.856782 |
| D130071N09 | 0.032903 | 0.504888 | 0.835957 |
| MYO18B | 0.032903 | 0.504888 | 1.091087 |
| MSX3 | 0.032922 | 0.504888 | 0.910922 |
| NEUROD2 | 0.032934 | 0.504888 | 1.153486 |
| PEA15A | 0.032945 | 0.504888 | 2.221858 |
| PIP4K2C | 0.032947 | 0.504888 | 1.656342 |
| RG9MTD1 | 0.032985 | 0.504888 | 0.70907 |
| 6430706D22RIK | 0.032993 | 0.504888 | 0.376451 |
| CMYA5 | 0.033 | 0.504888 | 1.123838 |
| SDF2 | 0.033005 | 0.504888 | 1.576254 |
| LOC381571 | 0.033047 | 0.505038 | 1.069004 |
| LOC665425 | 0.033054 | 0.505038 | 1.170994 |
| CXCL15 | 0.033064 | 0.505038 | 2.194714 |
| TMEM48 | 0.033089 | 0.505174 | 0.375278 |
| MPP6 | 0.033126 | 0.505307 | 0.533317 |
| LOC382553 | 0.03314 | 0.505307 | 0.87614 |
| SH3BGR | 0.033156 | 0.505307 | 1.076091 |
| SF3A1 | 0.033167 | 0.505307 | 0.691755 |
| NEURL | 0.033179 | 0.505307 | 2.057653 |
| KHDRBS2 | 0.033206 | 0.505451 | 0.885993 |
| D530007H06RIK | 0.033227 | 0.505451 | 0.765301 |
| LOC385360 | 0.033238 | 0.505451 | 1.059708 |
| TMCO6 | 0.033272 | 0.505633 | 0.698404 |
| VLDLR | 0.033292 | 0.505633 | 1.353724 |
| SPECC1L | 0.03331 | 0.505633 | 1.075942 |
| GJB1 | 0.033348 | 0.505633 | 0.955636 |
| UBE2C | 0.033355 | 0.505633 | 0.433459 |
| LZTS2 | 0.033401 | 0.505633 | 1.583006 |
| MIB1 | 0.033411 | 0.505633 | 0.852635 |
| DPT | 0.033411 | 0.505633 | 0.849351 |
| 2700078E11RIK | 0.033443 | 0.505633 | 0.85605 |
| EG433923 | 0.033447 | 0.505633 | 0.46308 |
| TMEM44 | 0.033454 | 0.505633 | 2.110766 |
| TRIM37 | 0.033479 | 0.505633 | 0.885542 |
| RORC | 0.033485 | 0.505633 | 0.847137 |
| LOC240779 | 0.033501 | 0.505633 | 1.086584 |
| OLFR763 | 0.033554 | 0.505633 | 1.161723 |
| PALLD | 0.033556 | 0.505633 | 1.743919 |
| SNTA1 | 0.033587 | 0.505633 | 1.055505 |
| CHEK1 | 0.033595 | 0.505633 | 0.702141 |
| RPO1-2 | 0.0336 | 0.505633 | 1.109954 |
| 2310022K01RIK | 0.033646 | 0.505633 | 0.532456 |
| LOC385729 | 0.033656 | 0.505633 | 0.984412 |
| OLFR49 | 0.033675 | 0.505633 | 1.125787 |
| ZCWPW1 | 0.0337 | 0.505633 | 0.788801 |
| PGLS | 0.033722 | 0.505633 | 0.513226 |
| 1190007I07RIK | 0.033747 | 0.505633 | 0.581936 |
| MON1B | 0.033771 | 0.505633 | 1.560887 |
| V1RD22 | 0.03378 | 0.505633 | 0.821861 |
| LOC382760 | 0.033793 | 0.505633 | 1.104454 |
| RNUXA | 0.033805 | 0.505633 | 0.958444 |
| E130007O11RIK | 0.033834 | 0.505633 | 0.819207 |
| PPP2R2D | 0.033848 | 0.505633 | 0.620714 |
| ASB1 | 0.033894 | 0.505633 | 0.919933 |
| D030069C22RIK | 0.0339 | 0.505633 | 0.896018 |
| ARHGAP17 | 0.0339 | 0.505633 | 0.960506 |
| CHRNB1 | 0.033908 | 0.505633 | 2.642127 |
| FISH | 0.033916 | 0.505633 | 0.86792 |
| TIMP3 | 0.033943 | 0.505633 | 3.122425 |
| LOC385914 | 0.03395 | 0.505633 | 0.82481 |
| HMGB2 | 0.033962 | 0.505633 | 0.354306 |
| A330045B10RIK | 0.033979 | 0.505633 | 1.139973 |
| 1700123J19RIK | 0.033985 | 0.505633 | 0.909829 |
| SGOL1 | 0.034003 | 0.505633 | 0.725728 |
| MVP | 0.03402 | 0.505633 | 1.608817 |
| LOC633945 | 0.034027 | 0.505633 | 0.401007 |
| SERPINF1 | 0.03405 | 0.505633 | 0.536742 |
| LOC654494 | 0.034052 | 0.505633 | 1.139552 |
| ATP6AP1 | 0.034085 | 0.505633 | 1.455317 |
| 2500002A22RIK | 0.034095 | 0.505633 | 0.338236 |
| USP29 | 0.034095 | 0.505633 | 0.886443 |
| NEK3 | 0.034101 | 0.505633 | 1.613806 |
| 4732471D19RIK | 0.034144 | 0.505633 | 0.726483 |
| PITX3 | 0.034145 | 0.505633 | 1.092374 |
| B130047O12RIK | 0.034146 | 0.505633 | 0.873512 |
| B130064I06RIK | 0.034161 | 0.505633 | 0.915797 |
| E030013D07RIK | 0.034176 | 0.505633 | 0.950395 |
| CENPM | 0.034183 | 0.505633 | 0.354404 |
| RALB | 0.034209 | 0.505633 | 1.666013 |
| 2810021O14RIK | 0.034232 | 0.505633 | 1.54535 |
| 4930432O21RIK | 0.034241 | 0.505633 | 0.898361 |
| FGF21 | 0.034255 | 0.505633 | 0.174762 |
| TRAF7 | 0.034261 | 0.505633 | 1.201775 |
| GSTCD | 0.034281 | 0.505633 | 0.593382 |
| SCL0001651.1_229 | 0.034282 | 0.505633 | 1.164841 |
| 1110001J03RIK | 0.034344 | 0.506278 | 0.406399 |
| 4930438A08RIK | 0.034377 | 0.506278 | 0.910628 |
| SBSN | 0.034426 | 0.506278 | 1.453435 |
| SLITRK5 | 0.034447 | 0.506278 | 0.870128 |
| BC030183 | 0.03446 | 0.506278 | 1.161724 |
| PRKX | 0.034479 | 0.506278 | 1.55725 |
| MICAL2 | 0.034496 | 0.506278 | 1.987792 |
| 9330158N06RIK | 0.034502 | 0.506278 | 0.635384 |
| OLFR109 | 0.034505 | 0.506278 | 0.933917 |
| MMP28 | 0.034518 | 0.506278 | 1.084452 |
| PR | 0.034542 | 0.506278 | 1.114451 |
| LOC381232 | 0.03455 | 0.506278 | 1.124331 |
| NXT2 | 0.034594 | 0.506278 | 0.553786 |
| HES3 | 0.034596 | 0.506278 | 0.838374 |
| TAF1D | 0.034627 | 0.506278 | 0.600679 |
| OLFR1231 | 0.034636 | 0.506278 | 0.935537 |
| SERPINB6A | 0.034642 | 0.506278 | 1.497615 |
| 9530043G02RIK | 0.034643 | 0.506278 | 0.947983 |
| TMEM150 | 0.034663 | 0.506278 | 1.707951 |
| 2310045N01RIK | 0.034663 | 0.506278 | 1.443596 |
| METAP1 | 0.03468 | 0.506278 | 0.742622 |
| OTOS | 0.034687 | 0.506278 | 1.043068 |
| LOC675098 | 0.034729 | 0.506521 | 1.101243 |
| DUS4L | 0.034737 | 0.506521 | 0.418829 |
| 9030227G01RIK | 0.034784 | 0.506698 | 0.922316 |
| PMCH | 0.034861 | 0.506698 | 1.208429 |
| B230306G18RIK | 0.034866 | 0.506698 | 1.046472 |
| RIOK1 | 0.03487 | 0.506698 | 0.852044 |
| SLCO4A1 | 0.034892 | 0.506698 | 0.829722 |
| D630035L11RIK | 0.034894 | 0.506698 | 1.212317 |
| CUL2 | 0.034896 | 0.506698 | 0.652704 |
| RIOK3 | 0.034908 | 0.506698 | 1.057702 |
| H2-K1 | 0.034951 | 0.506698 | 1.039555 |
| LOC384887 | 0.034965 | 0.506698 | 1.190197 |
| LOC218060 | 0.034976 | 0.506698 | 0.661739 |
| CTXN1 | 0.035001 | 0.506698 | 1.702239 |
| IL7 | 0.035011 | 0.506698 | 0.921826 |
| LOC100048020 | 0.035016 | 0.506698 | 1.350163 |
| BC030336 | 0.035026 | 0.506698 | 0.924321 |
| TRAF3IP1 | 0.035031 | 0.506698 | 1.038259 |
| A330074K22RIK | 0.035041 | 0.506698 | 1.145809 |
| LOC629364 | 0.035052 | 0.506698 | 1.598146 |
| SHC1 | 0.035061 | 0.506698 | 1.48521 |
| NDRG1 | 0.035083 | 0.506782 | 1.491055 |
| LOC238447 | 0.035112 | 0.506958 | 0.882907 |
| 1200016E24RIK | 0.035143 | 0.507149 | 1.808257 |
| 2810423A18RIK | 0.035167 | 0.507149 | 1.085882 |
| 2610029G23RIK | 0.035174 | 0.507149 | 0.75487 |
| LOC384184 | 0.035193 | 0.507179 | 1.106216 |
| PRIM2 | 0.035212 | 0.507223 | 0.465913 |
| LOC381234 | 0.035252 | 0.507561 | 1.123786 |
| ERP29 | 0.035271 | 0.507579 | 0.876606 |
| RUVBL2 | 0.035336 | 0.507579 | 0.413053 |
| GPX5 | 0.035342 | 0.507579 | 0.909282 |
| PPFIA3 | 0.035351 | 0.507579 | 1.162798 |
| CD6 | 0.035356 | 0.507579 | 0.785999 |
| 2610301G19RIK | 0.035388 | 0.507579 | 1.304443 |
| V2R12 | 0.03542 | 0.507579 | 0.953122 |
| TYR | 0.03542 | 0.507579 | 0.933788 |
| LOC100044180 | 0.035449 | 0.507579 | 0.8895 |
| 2410005O16RIK | 0.035462 | 0.507579 | 1.07014 |
| HEXIM1 | 0.035466 | 0.507579 | 1.361629 |
| SCOTIN | 0.035468 | 0.507579 | 1.420337 |
| C330029I24RIK | 0.03549 | 0.507579 | 0.902084 |
| 1110008P14RIK | 0.035495 | 0.507579 | 3.085989 |
| TRP53INP2 | 0.035509 | 0.507579 | 3.432676 |
| LOC100041769 | 0.035527 | 0.507579 | 0.895087 |
| LOC633979 | 0.035542 | 0.507579 | 1.071377 |
| D630004A14RIK | 0.035567 | 0.507579 | 0.579561 |
| LOC271048 | 0.035568 | 0.507579 | 0.924108 |
| GPR160 | 0.035582 | 0.507579 | 0.951934 |
| 4933406I18RIK | 0.035636 | 0.507929 | 1.181593 |
| UBE2T | 0.035661 | 0.507929 | 0.349493 |
| MMP13 | 0.035671 | 0.507929 | 1.476895 |
| 5-Sep | 0.035721 | 0.507929 | 1.335457 |
| D030068E18RIK | 0.035724 | 0.507929 | 0.893826 |
| A230091B22RIK | 0.035737 | 0.507929 | 1.13325 |
| 1110037F02RIK | 0.035747 | 0.507929 | 1.278484 |
| D330001F17RIK | 0.035752 | 0.507929 | 2.029794 |
| YAP1 | 0.03577 | 0.507929 | 1.408117 |
| 3110035F07RIK | 0.035789 | 0.507929 | 1.098042 |
| LOC383293 | 0.03579 | 0.507929 | 1.163308 |
| A430090J22 | 0.035829 | 0.507929 | 1.135688 |
| HOXA7 | 0.035858 | 0.507929 | 1.101854 |
| ITGAM | 0.035872 | 0.507929 | 0.939805 |
| 2410002I01RIK | 0.035941 | 0.507929 | 1.338175 |
| LOC434200 | 0.035952 | 0.507929 | 0.743755 |
| GPR126 | 0.035956 | 0.507929 | 1.458784 |
| LOC100047707 | 0.035962 | 0.507929 | 1.258583 |
| 4833426H15RIK | 0.03597 | 0.507929 | 0.930299 |
| BHMT | 0.035981 | 0.507929 | 0.908841 |
| PLEKHA3 | 0.035983 | 0.507929 | 1.153326 |
| C430026P20RIK | 0.036019 | 0.507929 | 0.715835 |
| LOC667609 | 0.036023 | 0.507929 | 0.491274 |
| 4930535E21RIK | 0.036037 | 0.507929 | 0.92445 |
| MGC117608 | 0.036042 | 0.507929 | 0.88146 |
| HNRPH1 | 0.036074 | 0.507929 | 0.661861 |
| FBP2 | 0.036094 | 0.507929 | 0.815996 |
| DBF4 | 0.036113 | 0.507929 | 0.349363 |
| MIS12 | 0.036137 | 0.507929 | 1.13942 |
| COQ5 | 0.036138 | 0.507929 | 0.671131 |
| EG236891 | 0.036171 | 0.507929 | 0.85439 |
| MUP1 | 0.036178 | 0.507929 | 0.858208 |
| LOC382949 | 0.0362 | 0.507929 | 1.050712 |
| ZFPM2 | 0.036214 | 0.507929 | 1.666475 |
| 2700094K13RIK | 0.036224 | 0.507929 | 0.346998 |
| TMEM93 | 0.036234 | 0.507929 | 0.777366 |
| LOC241084 | 0.036242 | 0.507929 | 0.877822 |
| E330017A01RIK | 0.036249 | 0.507929 | 0.824295 |
| ZFP472 | 0.036258 | 0.507929 | 0.630427 |
| LOC278041 | 0.03628 | 0.507929 | 1.153272 |
| TBC1D25 | 0.036282 | 0.507929 | 1.434982 |
| RASSF3 | 0.036307 | 0.508048 | 1.159551 |
| OLFR1424 | 0.036338 | 0.50826 | 0.934068 |
| RNPC2 | 0.036402 | 0.50833 | 0.736233 |
| CCDC85B | 0.036406 | 0.50833 | 1.6449 |
| LOC384206 | 0.036416 | 0.50833 | 0.486777 |
| ULK3 | 0.036435 | 0.50833 | 1.433193 |
| SMCR7 | 0.036438 | 0.50833 | 1.504621 |
| MARCKS | 0.036442 | 0.50833 | 1.572435 |
| ZFH4-PENDING | 0.036465 | 0.508383 | 1.12184 |
| 2810440D03RIK | 0.036486 | 0.508383 | 1.235618 |
| 2810449M09RIK | 0.036496 | 0.508383 | 1.368915 |
| DNAJC9 | 0.036514 | 0.508383 | 0.894922 |
| 2610524A10RIK | 0.036528 | 0.508383 | 1.55556 |
| TSPAN1 | 0.036612 | 0.509312 | 0.962083 |
| CHCHD6 | 0.036685 | 0.509357 | 0.474397 |
| JAK2 | 0.036697 | 0.509357 | 1.94531 |
| 4930563B10RIK | 0.036697 | 0.509357 | 0.316578 |
| FAM168A | 0.036717 | 0.509357 | 1.578076 |
| LOC229879 | 0.036721 | 0.509357 | 0.817845 |
| DACT1 | 0.036726 | 0.509357 | 1.098702 |
| DCI | 0.036731 | 0.509357 | 0.636912 |
| LOC100045567 | 0.036747 | 0.509357 | 1.341023 |
| COQ2 | 0.036811 | 0.510016 | 0.350438 |
| LOC382138 | 0.036828 | 0.510021 | 0.750557 |
| 2410001C21RIK | 0.036846 | 0.510021 | 1.459121 |
| LOC384371 | 0.036881 | 0.510021 | 0.850058 |
| CPT2 | 0.036883 | 0.510021 | 0.74072 |
| GAMT | 0.036894 | 0.510021 | 0.56275 |
| 4933416C03RIK | 0.036942 | 0.510454 | 0.844518 |
| DAPP1 | 0.03697 | 0.510612 | 0.691819 |
| RNMTL1 | 0.037015 | 0.510657 | 0.727793 |
| CENPL | 0.03702 | 0.510657 | 0.388872 |
| HIST1H2BE | 0.037063 | 0.510657 | 3.007714 |
| TRIM59 | 0.037072 | 0.510657 | 0.569842 |
| LOC208177 | 0.037112 | 0.510657 | 1.097155 |
| C130036G20RIK | 0.037116 | 0.510657 | 0.862064 |
| OLFR935 | 0.037123 | 0.510657 | 0.890961 |
| 1190002A17RIK | 0.037131 | 0.510657 | 0.968685 |
| LOC381398 | 0.037161 | 0.510657 | 0.575412 |
| MCM6 | 0.037163 | 0.510657 | 0.275788 |
| ARFIP1 | 0.037166 | 0.510657 | 1.126619 |
| DPY30 | 0.037172 | 0.510657 | 0.485878 |
| NTE | 0.037213 | 0.510995 | 1.412352 |
| 2610002D18RIK | 0.037279 | 0.511414 | 0.287919 |
| UTP11L | 0.037307 | 0.511414 | 0.621718 |
| B130034E13RIK | 0.037308 | 0.511414 | 0.903732 |
| TRERF1 | 0.03732 | 0.511414 | 0.596365 |
| LOC385830 | 0.037339 | 0.511414 | 1.093864 |
| GOLGB1 | 0.037343 | 0.511414 | 1.101854 |
| NDUFV1 | 0.037392 | 0.511861 | 0.666803 |
| CHML | 0.037416 | 0.511962 | 0.846608 |
| LOC239192 | 0.037517 | 0.512324 | 1.122229 |
| MMP1B | 0.037533 | 0.512324 | 1.149867 |
| TNFRSF9 | 0.037592 | 0.512324 | 1.447972 |
| KCTD5 | 0.037642 | 0.512324 | 2.529642 |
| ACAD8 | 0.037644 | 0.512324 | 0.811934 |
| 4921524L21RIK | 0.037649 | 0.512324 | 0.908295 |
| VPS11 | 0.037651 | 0.512324 | 1.636788 |
| LOC100047200 | 0.037682 | 0.512324 | 1.347109 |
| OLFR1331 | 0.037711 | 0.512324 | 1.108109 |
| 0610031J06RIK | 0.037713 | 0.512324 | 2.256886 |
| MTVR2 | 0.037738 | 0.512324 | 1.552687 |
| AARSD1 | 0.037749 | 0.512324 | 0.532382 |
| LOC384315 | 0.037751 | 0.512324 | 0.683873 |
| MYO1B | 0.037757 | 0.512324 | 1.375828 |
| SLC30A10 | 0.037801 | 0.512324 | 0.893868 |
| 2610303G11RIK | 0.037806 | 0.512324 | 1.152793 |
| MSL2L1 | 0.03781 | 0.512324 | 0.764347 |
| UBQLNL | 0.03782 | 0.512324 | 1.130975 |
| 9030624G23RIK | 0.037833 | 0.512324 | 0.80612 |
| 4931412M21 | 0.037843 | 0.512324 | 0.877396 |
| RHBDF2 | 0.03788 | 0.512324 | 1.161133 |
| 4930565A17 | 0.037885 | 0.512324 | 0.867759 |
| HSPD1 | 0.037887 | 0.512324 | 0.378754 |
| SMARCA3 | 0.037887 | 0.512324 | 0.731552 |
| LOC237856 | 0.037905 | 0.512324 | 0.916072 |
| A430024H01RIK | 0.037921 | 0.512324 | 1.183451 |
| A530029A01RIK | 0.037922 | 0.512324 | 1.156074 |
| AI450540 | 0.037926 | 0.512324 | 1.117468 |
| TLR2 | 0.037969 | 0.512324 | 2.050345 |
| QARS | 0.037975 | 0.512324 | 0.700149 |
| LRG1 | 0.038024 | 0.512324 | 1.579134 |
| EG545391 | 0.038033 | 0.512324 | 0.919019 |
| 1110031B06RIK | 0.038054 | 0.512324 | 1.374748 |
| PCDHA2 | 0.03809 | 0.512324 | 1.061987 |
| SCL0003522.1_58 | 0.038096 | 0.512324 | 0.92509 |
| ACP6 | 0.038098 | 0.512324 | 0.714051 |
| GALNT5 | 0.038102 | 0.512324 | 0.880523 |
| SERGEF | 0.038122 | 0.512324 | 0.634547 |
| D030018L15RIK | 0.038126 | 0.512324 | 0.892032 |
| LOC380979 | 0.038136 | 0.512324 | 1.192674 |
| ALDOA | 0.038139 | 0.512324 | 1.4914 |
| 5330439M10RIK | 0.03814 | 0.512324 | 1.112882 |
| OLFR1282 | 0.038176 | 0.512558 | 1.154232 |
| GSTM4 | 0.038191 | 0.512558 | 0.806251 |
| JMJD2B | 0.038235 | 0.512651 | 1.798175 |
| RPL22L1 | 0.038249 | 0.512651 | 0.698565 |
| EFEMP2 | 0.03827 | 0.512651 | 1.966777 |
| HDDC2 | 0.038283 | 0.512651 | 0.602389 |
| LOC380888 | 0.038294 | 0.512651 | 0.808844 |
| ORC6L | 0.038319 | 0.512651 | 0.628753 |
| IL12RB1 | 0.038325 | 0.512651 | 0.825134 |
| SERINC2 | 0.038365 | 0.512651 | 1.098601 |
| MCOLN1 | 0.038366 | 0.512651 | 1.808842 |
| A130076G11RIK | 0.038369 | 0.512651 | 0.840333 |
| RAB11FIP1 | 0.038387 | 0.512651 | 0.853009 |
| LOC382436 | 0.038423 | 0.512651 | 1.12376 |
| WDR72 | 0.038425 | 0.512651 | 0.914888 |
| RNASE11 | 0.038436 | 0.512651 | 1.052607 |
| ULK2 | 0.038463 | 0.512651 | 1.334131 |
| LOC224882 | 0.03849 | 0.512651 | 0.897572 |
| LOC217236 | 0.038572 | 0.512651 | 1.062527 |
| LSM6 | 0.038579 | 0.512651 | 0.729746 |
| DRBP1 | 0.038586 | 0.512651 | 1.403245 |
| SNX27 | 0.038594 | 0.512651 | 0.771872 |
| LOC100045188 | 0.038597 | 0.512651 | 0.923361 |
| RAB13 | 0.038621 | 0.512651 | 1.387929 |
| 2410017P07RIK | 0.038641 | 0.512651 | 0.437868 |
| E030040J04RIK | 0.038735 | 0.512651 | 1.104964 |
| EHD3 | 0.038738 | 0.512651 | 1.087639 |
| NECAP2 | 0.0388 | 0.512651 | 1.114039 |
| 6330408A02RIK | 0.038836 | 0.512651 | 1.224478 |
| NEDL2 | 0.038853 | 0.512651 | 0.858526 |
| 2500002G23RIK | 0.038881 | 0.512651 | 1.059953 |
| OLFR632 | 0.03891 | 0.512651 | 1.074997 |
| GINS1 | 0.03893 | 0.512651 | 0.387544 |
| NMI | 0.038933 | 0.512651 | 1.125865 |
| LOC386018 | 0.038948 | 0.512651 | 1.222471 |
| FRMD4A | 0.038963 | 0.512651 | 1.128338 |
| LOC100044089 | 0.038979 | 0.512651 | 1.138762 |
| 2610204K14RIK | 0.038987 | 0.512651 | 1.156448 |
| BNIP3 | 0.038992 | 0.512651 | 1.173323 |
| 6530439I21 | 0.039002 | 0.512651 | 1.095382 |
| VPS53 | 0.039022 | 0.512651 | 1.626758 |
| B230214G05 | 0.039023 | 0.512651 | 0.809648 |
| TRAP1 | 0.039037 | 0.512651 | 0.338681 |
| FGF9 | 0.03904 | 0.512651 | 0.706225 |
| TJAP1 | 0.039048 | 0.512651 | 1.145756 |
| 9130221D24RIK | 0.039049 | 0.512651 | 1.0814 |
| FAM13B | 0.039123 | 0.512651 | 0.912566 |
| IGHG1_J00453$V00793_IG_HEAVY_CONSTANT_GAMMA_1_792 | 0.039132 | 0.512651 | 1.13892 |
| LOC380688 | 0.039135 | 0.512651 | 0.757036 |
| NMUR1 | 0.03914 | 0.512651 | 1.176254 |
| SCL0001905.1_3 | 0.039142 | 0.512651 | 1.522877 |
| LOC383850 | 0.039169 | 0.512651 | 0.625204 |
| 8430410A17RIK | 0.039175 | 0.512651 | 0.855121 |
| ASZ1 | 0.039182 | 0.512651 | 0.72924 |
| D11BWG0280E | 0.039189 | 0.512651 | 0.870913 |
| EG434858 | 0.039194 | 0.512651 | 0.48375 |
| TMEM186 | 0.039227 | 0.512651 | 0.605906 |
| MBD2 | 0.039247 | 0.512651 | 1.078206 |
| FRAG1 | 0.039281 | 0.512651 | 0.867639 |
| 1110007L15RIK | 0.039303 | 0.512651 | 0.603043 |
| SIAT9 | 0.039331 | 0.512651 | 2.507182 |
| LOC381636 | 0.039336 | 0.512651 | 0.900106 |
| D530018J11RIK | 0.039344 | 0.512651 | 0.720365 |
| MITD1 | 0.039389 | 0.512651 | 0.915797 |
| JAM2 | 0.039396 | 0.512651 | 0.888309 |
| AV273951 | 0.039414 | 0.512651 | 1.150691 |
| TTC27 | 0.039422 | 0.512651 | 0.703619 |
| 9630046H06RIK | 0.039443 | 0.512651 | 1.100913 |
| MAGEA9 | 0.039456 | 0.512651 | 0.874765 |
| CREBBP | 0.039457 | 0.512651 | 1.040084 |
| SSH3 | 0.039458 | 0.512651 | 1.422735 |
| MTDH | 0.03948 | 0.512651 | 0.724438 |
| NFYB | 0.039485 | 0.512651 | 0.581426 |
| TRMT1 | 0.039488 | 0.512651 | 0.806157 |
| CDCA8 | 0.039504 | 0.512651 | 0.272841 |
| SDC3 | 0.039506 | 0.512651 | 1.800004 |
| BAD | 0.039536 | 0.512651 | 1.596449 |
| NOC4L | 0.039563 | 0.512651 | 0.548159 |
| TRIM10 | 0.03958 | 0.512651 | 0.908065 |
| MAPT | 0.039611 | 0.512651 | 0.831756 |
| MT-CYTB | 0.039627 | 0.512651 | 1.398616 |
| ITIH5L | 0.039647 | 0.512651 | 1.141871 |
| 5330406H09RIK | 0.039649 | 0.512651 | 1.151196 |
| MFAP3 | 0.039652 | 0.512651 | 1.524638 |
| SRPK1 | 0.039668 | 0.512651 | 0.645356 |
| ANUBL1 | 0.039689 | 0.512651 | 1.110134 |
| TMEM120A | 0.0397 | 0.512651 | 1.450449 |
| MRAS | 0.039703 | 0.512651 | 1.30041 |
| PDZD4 | 0.03971 | 0.512651 | 1.068905 |
| RNF11 | 0.039719 | 0.512651 | 2.033925 |
| CCNC | 0.039744 | 0.512651 | 0.664635 |
| SCL0001464.1_61 | 0.039784 | 0.512651 | 1.138815 |
| 5730403B10RIK | 0.039798 | 0.512651 | 1.244558 |
| LEP | 0.039815 | 0.512651 | 1.073781 |
| PBK | 0.039871 | 0.512651 | 0.203354 |
| LRSAM1 | 0.039896 | 0.512651 | 1.039315 |
| A930034L24RIK | 0.039948 | 0.512651 | 1.119432 |
| TIRAP | 0.03995 | 0.512651 | 1.099769 |
| OLFR154 | 0.03996 | 0.512651 | 1.055456 |
| ZFP52 | 0.039992 | 0.512651 | 1.401171 |
| LOC385809 | 0.039999 | 0.512651 | 1.110826 |
| STX3 | 0.040009 | 0.512651 | 1.215121 |
| GSDMDC1 | 0.040009 | 0.512651 | 1.103434 |
| 2810410M20RIK | 0.040012 | 0.512651 | 0.576157 |
| LGI2 | 0.040031 | 0.512651 | 1.080151 |
| MUC15 | 0.040048 | 0.512651 | 1.110878 |
| D19BWG1357E | 0.040054 | 0.512651 | 0.630106 |
| LOC381378 | 0.040105 | 0.512651 | 0.937246 |
| P4HB | 0.040121 | 0.512651 | 1.411928 |
| CRIP2 | 0.040125 | 0.512651 | 1.494159 |
| MJ-1000-77_495 | 0.040136 | 0.512651 | 1.131419 |
| TSC22D2 | 0.040148 | 0.512651 | 1.679735 |
| LOC630320 | 0.04016 | 0.512651 | 0.879791 |
| GM169 | 0.040175 | 0.512651 | 0.606817 |
| LOC100048845 | 0.040215 | 0.512651 | 1.215233 |
| VPS36 | 0.040247 | 0.512651 | 0.565801 |
| FOXB1 | 0.040264 | 0.512651 | 0.750037 |
| 2810003O05RIK | 0.040266 | 0.512651 | 0.965735 |
| D10ERTD610E | 0.040281 | 0.512651 | 1.66713 |
| PIAS3 | 0.040284 | 0.512651 | 1.245853 |
| SCL000530.1_2 | 0.040288 | 0.512651 | 1.16226 |
| NFKBIZ | 0.040298 | 0.512651 | 2.276105 |
| E030024M20RIK | 0.04033 | 0.512651 | 1.159926 |
| CCT5 | 0.040347 | 0.512651 | 0.537785 |
| CNP | 0.040353 | 0.512651 | 1.14203 |
| 1700034F02RIK | 0.040361 | 0.512651 | 0.913304 |
| RELB | 0.040369 | 0.512651 | 1.925278 |
| 6530402F18RIK | 0.040394 | 0.512651 | 1.084878 |
| LOC241385 | 0.040402 | 0.512651 | 1.444096 |
| SDK1 | 0.040405 | 0.512651 | 0.893207 |
| CCNH | 0.040418 | 0.512651 | 0.593341 |
| 1700027D21RIK | 0.040418 | 0.512651 | 0.877802 |
| TREX1 | 0.040428 | 0.512651 | 0.862144 |
| LOC100048796 | 0.040473 | 0.512651 | 1.557286 |
| PPAT | 0.040497 | 0.512651 | 0.665511 |
| 5730575G16RIK | 0.04051 | 0.512651 | 0.839635 |
| NCOA6 | 0.040511 | 0.512651 | 0.752345 |
| NDC80 | 0.040528 | 0.512651 | 0.25015 |
| TOPBP1 | 0.040535 | 0.512651 | 0.858189 |
| D530024B08RIK | 0.040538 | 0.512651 | 0.881725 |
| D130051G04RIK | 0.040548 | 0.512651 | 0.80322 |
| A930006D20RIK | 0.040571 | 0.512651 | 1.155246 |
| SLITRK6 | 0.040611 | 0.512651 | 1.192399 |
| A930104D05RIK | 0.040631 | 0.512651 | 1.15757 |
| THOP1 | 0.040636 | 0.512651 | 0.466323 |
| 4930526D03RIK | 0.040651 | 0.512651 | 1.100023 |
| 2310016E02RIK | 0.040673 | 0.512651 | 1.820204 |
| PPT1 | 0.040679 | 0.512651 | 1.503961 |
| HTRA4 | 0.040682 | 0.512651 | 1.085681 |
| RAD50 | 0.040692 | 0.512651 | 0.689394 |
| 4933432P15RIK | 0.040696 | 0.512651 | 0.91322 |
| EG433229 | 0.040722 | 0.512651 | 1.187313 |
| LOC380748 | 0.040723 | 0.512651 | 0.845397 |
| ATXN10 | 0.040734 | 0.512651 | 0.839343 |
| TIPIN | 0.040748 | 0.512651 | 0.927166 |
| AURKA | 0.040756 | 0.512651 | 0.259991 |
| PSMD14 | 0.040779 | 0.512723 | 0.701687 |
| BC006662 | 0.040827 | 0.513127 | 1.250842 |
| PRKAA2 | 0.040848 | 0.513182 | 0.621087 |
| LOC386405 | 0.040903 | 0.513661 | 0.26137 |
| GPR77 | 0.040926 | 0.513706 | 0.931073 |
| IGHV1S59_L17134_IG_HEAVY_VARIABLE_1S59_150 | 0.04094 | 0.513706 | 0.84655 |
| LOC671761 | 0.040995 | 0.514005 | 0.846941 |
| 2900016G23RIK | 0.040997 | 0.514005 | 1.454712 |
| LY6E | 0.041015 | 0.514014 | 1.406718 |
| 3830425H19RIK | 0.041097 | 0.514837 | 0.946889 |
| 2610003J06RIK | 0.041144 | 0.515194 | 1.161107 |
| PGK2 | 0.041168 | 0.515194 | 1.088896 |
| GM1752 | 0.041269 | 0.515194 | 1.023303 |
| SOX12 | 0.041276 | 0.515194 | 0.656545 |
| BC022765 | 0.041309 | 0.515194 | 1.13435 |
| UNC5H3 | 0.041318 | 0.515194 | 0.868481 |
| PCDHGB4 | 0.041319 | 0.515194 | 1.319325 |
| DDX59 | 0.041323 | 0.515194 | 0.853009 |
| C230029F24RIK | 0.04133 | 0.515194 | 0.865077 |
| SRPR | 0.041336 | 0.515194 | 1.307701 |
| 2410170E21RIK | 0.041358 | 0.515194 | 0.783189 |
| A930023F12RIK | 0.041389 | 0.515194 | 0.880564 |
| IFNA4 | 0.041389 | 0.515194 | 0.945293 |
| LOC381889 | 0.0414 | 0.515194 | 0.761544 |
| CHD4 | 0.041401 | 0.515194 | 0.871899 |
| SRMS | 0.041402 | 0.515194 | 0.891249 |
| WHSC2 | 0.041437 | 0.515194 | 0.723317 |
| TAAR7B | 0.041442 | 0.515194 | 0.73855 |
| NCAML-PENDING | 0.041443 | 0.515194 | 0.838742 |
| PSMC6 | 0.041517 | 0.515586 | 0.786036 |
| 2810025M15RIK | 0.041524 | 0.515586 | 0.524616 |
| FSHR | 0.041524 | 0.515586 | 1.161992 |
| PPP1R3C | 0.041548 | 0.515669 | 1.170831 |
| GRN | 0.041599 | 0.515745 | 1.510472 |
| 1810019J16RIK | 0.041627 | 0.515745 | 1.024226 |
| 1200017F15RIK | 0.041638 | 0.515745 | 1.092904 |
| ZFP296 | 0.041649 | 0.515745 | 0.757823 |
| YIPF2 | 0.041674 | 0.515745 | 0.746648 |
| KERA | 0.041674 | 0.515745 | 0.871839 |
| 6430518K01RIK | 0.041676 | 0.515745 | 0.947917 |
| LOC332100 | 0.041703 | 0.515745 | 0.915586 |
| KNG1 | 0.041719 | 0.515745 | 3.083993 |
| ABCG3 | 0.041721 | 0.515745 | 0.887919 |
| 2310042E22RIK | 0.04174 | 0.515775 | 0.914824 |
| PPIC | 0.041832 | 0.516707 | 0.808134 |
| RRP1B | 0.04186 | 0.516843 | 0.528106 |
| BC023488 | 0.041977 | 0.517819 | 0.657714 |
| RARG | 0.042013 | 0.517819 | 1.238248 |
| CDCA3 | 0.042013 | 0.517819 | 0.255064 |
| CEP350 | 0.042017 | 0.517819 | 0.782828 |
| PDLIM5 | 0.042035 | 0.517819 | 0.901376 |
| CCDC65 | 0.04204 | 0.517819 | 1.221652 |
| DIMT1 | 0.042088 | 0.518167 | 0.824029 |
| LITAF | 0.042102 | 0.518167 | 1.573161 |
| GOLGA5 | 0.042143 | 0.518473 | 1.280937 |
| RNF5 | 0.042195 | 0.518538 | 1.086861 |
| 4930544G11RIK | 0.042207 | 0.518538 | 0.916835 |
| MECP2 | 0.042218 | 0.518538 | 1.590742 |
| C630007B19RIK | 0.042222 | 0.518538 | 0.919847 |
| EIF3S10 | 0.042238 | 0.518538 | 0.789585 |
| DTX1 | 0.042249 | 0.518538 | 0.841363 |
| LOC639609 | 0.04229 | 0.518829 | 1.129851 |
| 2610209A20RIK | 0.042307 | 0.518829 | 0.832006 |
| ESX1 | 0.042334 | 0.518956 | 1.117933 |
| A430038C16RIK | 0.042355 | 0.519005 | 0.93716 |
| OLFR905 | 0.042406 | 0.519426 | 0.883213 |
| GRSF1 | 0.042429 | 0.519441 | 0.655803 |
| LOC381212 | 0.042441 | 0.519441 | 0.849881 |
| HIST1H1C | 0.042472 | 0.519619 | 3.052518 |
| MAML2 | 0.042532 | 0.520141 | 1.513722 |
| LOC381727 | 0.04259 | 0.520203 | 1.140685 |
| SLC38A6 | 0.042592 | 0.520203 | 1.293967 |
| IGH-VS107 | 0.042603 | 0.520203 | 0.786344 |
| UGT1A10 | 0.042609 | 0.520203 | 1.267513 |
| B130045C05RIK | 0.042653 | 0.520203 | 0.850078 |
| LOC675735 | 0.042657 | 0.520203 | 0.881012 |
| CCDC109A | 0.042664 | 0.520203 | 0.645147 |
| 2810046L04RIK | 0.042677 | 0.520203 | 1.460201 |
| RSN | 0.042706 | 0.520203 | 1.937819 |
| NCL | 0.042709 | 0.520203 | 0.416831 |
| SUSD2 | 0.042722 | 0.520203 | 0.873109 |
| ATP8B2 | 0.042748 | 0.520242 | 1.215149 |
| NPCD | 0.042766 | 0.520242 | 0.853127 |
| SPATA2L | 0.042776 | 0.520242 | 1.613582 |
| MIC2L1 | 0.042844 | 0.520306 | 1.39571 |
| GTF2H2 | 0.042861 | 0.520306 | 0.889747 |
| LOC669520 | 0.042875 | 0.520306 | 0.955879 |
| LOC381330 | 0.042879 | 0.520306 | 0.600124 |
| LOC634731 | 0.042921 | 0.520306 | 2.674064 |
| C230080I20RIK | 0.042928 | 0.520306 | 0.94113 |
| LOC386279 | 0.042955 | 0.520306 | 1.088519 |
| RPS6KA6 | 0.042998 | 0.520306 | 1.046133 |
| RPP30 | 0.04303 | 0.520306 | 0.596241 |
| A730042J05RIK | 0.043043 | 0.520306 | 0.826145 |
| SH3BGRL3 | 0.043047 | 0.520306 | 1.6752 |
| ZFP367 | 0.04305 | 0.520306 | 0.656015 |
| LOC385828 | 0.043064 | 0.520306 | 0.832949 |
| MRGPRE | 0.043095 | 0.520306 | 1.23945 |
| NSBP1 | 0.043097 | 0.520306 | 0.37354 |
| STX1B | 0.043112 | 0.520306 | 0.953056 |
| 9430079A18RIK | 0.043117 | 0.520306 | 1.118811 |
| SEC61A2 | 0.043128 | 0.520306 | 0.689936 |
| SLC31A2 | 0.043137 | 0.520306 | 1.278749 |
| PPME1 | 0.043146 | 0.520306 | 1.626007 |
| LOC234081 | 0.043156 | 0.520306 | 0.383846 |
| TPBG | 0.043165 | 0.520306 | 2.158456 |
| IDH1 | 0.043169 | 0.520306 | 1.459627 |
| BRF2 | 0.043225 | 0.520782 | 1.063829 |
| 5730408I11RIK | 0.043286 | 0.521304 | 0.880666 |
| BOLA2 | 0.043323 | 0.521526 | 0.750539 |
| NDUFA10 | 0.043367 | 0.521526 | 0.700602 |
| TCF25 | 0.043378 | 0.521526 | 1.697407 |
| LOC386194 | 0.043379 | 0.521526 | 1.144513 |
| WFDC6B | 0.04341 | 0.521526 | 1.109569 |
| CLASP2 | 0.043419 | 0.521526 | 0.733651 |
| CCDC5 | 0.043422 | 0.521526 | 0.481831 |
| LOC384109 | 0.043445 | 0.521544 | 0.924962 |
| 1810013D10RIK | 0.043521 | 0.521544 | 1.251508 |
| 2900010M23RIK | 0.043525 | 0.521544 | 0.858764 |
| 4432406C05RIK | 0.043579 | 0.521544 | 0.716266 |
| LCE1G | 0.0436 | 0.521544 | 1.168345 |
| LOC433745 | 0.043625 | 0.521544 | 0.780426 |
| LOC383280 | 0.043638 | 0.521544 | 1.056969 |
| KCNU1 | 0.043653 | 0.521544 | 0.956697 |
| MCPH1 | 0.043654 | 0.521544 | 0.790078 |
| SARS2 | 0.043663 | 0.521544 | 0.950374 |
| SGPL1 | 0.043667 | 0.521544 | 1.340496 |
| 6330503K22RIK | 0.043699 | 0.521544 | 0.88796 |
| 4933412A08RIK | 0.043723 | 0.521544 | 1.139473 |
| SLAMF7 | 0.043742 | 0.521544 | 0.949781 |
| LOC627199 | 0.043743 | 0.521544 | 0.861248 |
| 2410095B20RIK | 0.043748 | 0.521544 | 0.942589 |
| 3110023B02RIK | 0.043749 | 0.521544 | 0.596558 |
| DNAHC5 | 0.043765 | 0.521544 | 1.072616 |
| ERICH1 | 0.043767 | 0.521544 | 1.390561 |
| AURKB | 0.043778 | 0.521544 | 0.218706 |
| PODXL2 | 0.043789 | 0.521544 | 2.036794 |
| WTAP | 0.043808 | 0.521544 | 0.903794 |
| 1700041B20RIK | 0.043813 | 0.521544 | 0.753372 |
| RBPJL | 0.043923 | 0.522107 | 1.102797 |
| HIST1H2BJ | 0.043931 | 0.522107 | 2.719484 |
| LRRC18 | 0.043939 | 0.522107 | 1.0894 |
| MAP3K9 | 0.04397 | 0.522107 | 1.094698 |
| SLC10A2 | 0.043971 | 0.522107 | 0.844635 |
| 6330407O08RIK | 0.043981 | 0.522107 | 0.816693 |
| LOC434609 | 0.044005 | 0.522107 | 1.163362 |
| SLC10A1 | 0.044007 | 0.522107 | 0.9334 |
| A630034I12RIK | 0.044032 | 0.522107 | 0.483046 |
| ERCC1 | 0.044046 | 0.522107 | 1.217116 |
| STOML1 | 0.044046 | 0.522107 | 1.461821 |
| LOC100038860 | 0.044145 | 0.522927 | 0.82886 |
| STAP2 | 0.044162 | 0.522927 | 1.67989 |
| GMPS | 0.044166 | 0.522927 | 0.542464 |
| ZFP110 | 0.044204 | 0.522983 | 1.097129 |
| TRIM66 | 0.044235 | 0.522983 | 1.160275 |
| HNRNPK | 0.044238 | 0.522983 | 0.870973 |
| IGTP | 0.044239 | 0.522983 | 1.304141 |
| RPL10A | 0.044257 | 0.522998 | 0.880665 |
| YME1L1 | 0.04429 | 0.523184 | 0.817864 |
| C730026J16 | 0.044338 | 0.523347 | 1.788024 |
| ZFP473 | 0.044338 | 0.523347 | 0.58054 |
| LOC381144 | 0.044354 | 0.523347 | 0.956343 |
| IKBKE | 0.044403 | 0.523676 | 1.111006 |
| NDUFA12L | 0.044416 | 0.523676 | 0.688661 |
| PCP4 | 0.044438 | 0.523736 | 0.918658 |
| PSAP | 0.044487 | 0.523862 | 1.935894 |
| USP49 | 0.044515 | 0.523862 | 0.927145 |
| APPBP2 | 0.044521 | 0.523862 | 0.876606 |
| KRT9 | 0.044551 | 0.523862 | 1.07736 |
| MTL5 | 0.044565 | 0.523862 | 0.838743 |
| BCL7B | 0.04457 | 0.523862 | 1.261465 |
| GI_39893_EMB_X17013.1_BSDPD_1251 | 0.0446 | 0.523862 | 0.887099 |
| PCP2 | 0.044613 | 0.523862 | 2.035383 |
| OLFR90 | 0.044616 | 0.523862 | 1.124461 |
| PIGL | 0.044648 | 0.523862 | 0.643569 |
| D930014A20RIK | 0.044711 | 0.523862 | 0.954511 |
| SMAD3 | 0.044712 | 0.523862 | 1.675548 |
| 4930579C15RIK | 0.044712 | 0.523862 | 1.062503 |
| D15WSU169E | 0.044723 | 0.523862 | 0.821273 |
| LOC640196 | 0.044725 | 0.523862 | 0.825363 |
| KLHDC3 | 0.044824 | 0.523862 | 1.304955 |
| USP20 | 0.044831 | 0.523862 | 1.216891 |
| TPX2 | 0.044836 | 0.523862 | 0.268427 |
| SCL0002976.1_36 | 0.044843 | 0.523862 | 1.061031 |
| 9530051K01RIK | 0.044852 | 0.523862 | 1.590889 |
| MIF4GD | 0.044876 | 0.523862 | 1.387223 |
| LOC385359 | 0.044881 | 0.523862 | 0.868341 |
| C730029F17RIK | 0.044888 | 0.523862 | 0.474474 |
| DDX39 | 0.044911 | 0.523862 | 0.767816 |
| CRLS1 | 0.044918 | 0.523862 | 0.763641 |
| SDC2 | 0.044924 | 0.523862 | 1.278424 |
| PSKH1 | 0.044936 | 0.523862 | 1.275149 |
| NCAPH | 0.044942 | 0.523862 | 0.230536 |
| THBS1 | 0.044965 | 0.523862 | 2.273109 |
| UFC1 | 0.044976 | 0.523862 | 1.326539 |
| TAAR7E | 0.044985 | 0.523862 | 1.09794 |
| IER3 | 0.045011 | 0.523862 | 2.611115 |
| KIF4 | 0.04502 | 0.523862 | 0.422913 |
| CCDC97 | 0.045026 | 0.523862 | 1.261523 |
| C130040D06RIK | 0.045045 | 0.523889 | 1.145571 |
| 9430076C15RIK | 0.045083 | 0.524133 | 0.924663 |
| OLFR543 | 0.045119 | 0.524346 | 0.847293 |
| 9430025L01RIK | 0.045182 | 0.524497 | 0.918022 |
| PEX10 | 0.045184 | 0.524497 | 0.673788 |
| HPN | 0.045202 | 0.524497 | 1.267776 |
| FAM114A2 | 0.04522 | 0.524497 | 1.317375 |
| DNM1L | 0.045247 | 0.524497 | 0.664297 |
| FLII | 0.045262 | 0.524497 | 1.434518 |
| DACH1 | 0.045267 | 0.524497 | 0.94954 |
| FOXRED1 | 0.045268 | 0.524497 | 0.620284 |
| NME4 | 0.045313 | 0.524827 | 0.808302 |
| TTC9 | 0.045367 | 0.525012 | 1.061325 |
| 1100001J08RIK | 0.045382 | 0.525012 | 0.709217 |
| UBAC1 | 0.045397 | 0.525012 | 0.677394 |
| A030004J04RIK | 0.045414 | 0.525012 | 1.079453 |
| SLC25A14 | 0.045427 | 0.525012 | 1.149256 |
| CUEDC1 | 0.045466 | 0.525012 | 1.436608 |
| LOC667005 | 0.045522 | 0.525012 | 0.233625 |
| A730045E13RIK | 0.045531 | 0.525012 | 0.954577 |
| CTSE | 0.045573 | 0.525012 | 0.950242 |
| FKBP5 | 0.045573 | 0.525012 | 0.688868 |
| MRFAP1 | 0.045586 | 0.525012 | 1.101218 |
| TIMM50 | 0.045588 | 0.525012 | 0.627535 |
| 4732496O08RIK | 0.045622 | 0.525012 | 1.402629 |
| D330003G07RIK | 0.045629 | 0.525012 | 0.696856 |
| MFAP2 | 0.04563 | 0.525012 | 1.170804 |
| SLC25A24 | 0.045632 | 0.525012 | 1.045964 |
| 8430407G10RIK | 0.045635 | 0.525012 | 0.657213 |
| H28 | 0.045635 | 0.525012 | 1.120855 |
| LOC236311 | 0.04566 | 0.525098 | 1.099794 |
| SLC39A2 | 0.045683 | 0.525164 | 1.126775 |
| 4733401O04RIK | 0.045723 | 0.525394 | 0.828094 |
| 4930535I16RIK | 0.045737 | 0.525394 | 1.096141 |
| CHD1L | 0.045756 | 0.525394 | 0.524749 |
| PRR11 | 0.045771 | 0.525394 | 0.388486 |
| LOC385414 | 0.045793 | 0.5254 | 1.091264 |
| WDR51B | 0.045805 | 0.5254 | 0.742862 |
| LOC235243 | 0.045871 | 0.52562 | 1.066364 |
| 9530058K19RIK | 0.045871 | 0.52562 | 1.029588 |
| 9430018C17RIK | 0.045885 | 0.52562 | 0.899939 |
| LOC213440 | 0.045895 | 0.52562 | 0.930514 |
| SEMA6B | 0.04591 | 0.52562 | 0.857673 |
| 2610024G14RIK | 0.045927 | 0.52562 | 0.561374 |
| FERT2 | 0.04595 | 0.525689 | 1.566198 |
| PPIF | 0.045986 | 0.525905 | 0.582044 |
| HMOX1 | 0.046042 | 0.5262 | 2.327745 |
| LIPN | 0.0461 | 0.5262 | 1.076315 |
| A130095C03RIK | 0.046105 | 0.5262 | 0.872181 |
| PSG26 | 0.046137 | 0.5262 | 0.949518 |
| LOC236356 | 0.046142 | 0.5262 | 1.086609 |
| USMG3 | 0.046143 | 0.5262 | 1.559842 |
| 4930471M23RIK | 0.046158 | 0.5262 | 1.288358 |
| OLFR121 | 0.046162 | 0.5262 | 0.931654 |
| LOC671564 | 0.046165 | 0.5262 | 1.17251 |
| PRSS29 | 0.046197 | 0.52629 | 0.901084 |
| 2310051N18RIK | 0.04622 | 0.52629 | 0.643257 |
| 6330407A03RIK | 0.046243 | 0.52629 | 0.879771 |
| ANKRD29 | 0.046265 | 0.52629 | 0.926545 |
| PSMD11 | 0.046278 | 0.52629 | 0.875897 |
| 4831413G18RIK | 0.046299 | 0.52629 | 1.062748 |
| UBL5 | 0.046306 | 0.52629 | 1.543637 |
| GDAP4 | 0.046309 | 0.52629 | 0.826221 |
| 2310031L18RIK | 0.046339 | 0.526433 | 0.479987 |
| CD47 | 0.046418 | 0.527012 | 1.379775 |
| 1810030N24RIK | 0.046424 | 0.527012 | 1.396453 |
| AGTR1A | 0.046444 | 0.527044 | 0.858307 |
| OLFR559 | 0.046467 | 0.527109 | 0.825286 |
| LRFN1 | 0.046502 | 0.527312 | 1.171751 |
| BUB1B | 0.04654 | 0.527547 | 0.194899 |
| PARP3 | 0.046571 | 0.527695 | 1.644558 |
| NRP | 0.046625 | 0.527695 | 1.414148 |
| PNMT | 0.046635 | 0.527695 | 0.808377 |
| STC2 | 0.046649 | 0.527695 | 0.394064 |
| RNASE6 | 0.046661 | 0.527695 | 0.904171 |
| SLC7A9 | 0.04667 | 0.527695 | 1.048602 |
| SH3BP5L | 0.046673 | 0.527695 | 1.537336 |
| KIRREL3 | 0.04669 | 0.527695 | 1.802418 |
| ELL2 | 0.046708 | 0.527714 | 1.128964 |
| SAMD9L | 0.046753 | 0.52787 | 2.284693 |
| D930048N14RIK | 0.046756 | 0.52787 | 0.792418 |
| ZBTB6 | 0.046808 | 0.527904 | 0.929912 |
| FADS6 | 0.046838 | 0.527904 | 1.055164 |
| P4HA2 | 0.04686 | 0.527904 | 1.678299 |
| LOC100039496 | 0.046863 | 0.527904 | 1.656839 |
| 2300004C15RIK | 0.046867 | 0.527904 | 1.315489 |
| SLC1A2 | 0.046877 | 0.527904 | 0.859081 |
| COL6A2 | 0.046884 | 0.527904 | 1.326508 |
| FKBP11 | 0.046896 | 0.527904 | 0.445604 |
| PDXDC1 | 0.046932 | 0.528006 | 1.127453 |
| 4930533K18RIK | 0.046953 | 0.528006 | 1.932319 |
| LENEP | 0.046967 | 0.528006 | 1.046931 |
| 4931406P16RIK | 0.046987 | 0.528006 | 1.337897 |
| MTDNA_ND4 | 0.046991 | 0.528006 | 1.757267 |
| PRRG2 | 0.047103 | 0.528614 | 1.722615 |
| ACTN1 | 0.047151 | 0.528614 | 1.483495 |
| CKLF | 0.04716 | 0.528614 | 1.088318 |
| TMCO3 | 0.047183 | 0.528614 | 1.9227 |
| PNLIPRP1 | 0.047198 | 0.528614 | 0.836652 |
| AMD2 | 0.047199 | 0.528614 | 0.755411 |
| UBE2H | 0.047201 | 0.528614 | 1.23799 |
| RTN4R | 0.047203 | 0.528614 | 1.062307 |
| 2310051F07RIK | 0.047206 | 0.528614 | 1.467438 |
| ITGA6 | 0.047216 | 0.528614 | 1.108954 |
| ST5 | 0.047275 | 0.528778 | 1.930534 |
| PUM2 | 0.047279 | 0.528778 | 0.796456 |
| BC088983 | 0.047285 | 0.528778 | 0.486732 |
| EG433144 | 0.047312 | 0.528778 | 0.640735 |
| 6430407D20RIK | 0.047334 | 0.528778 | 0.769148 |
| OBRGRP | 0.047334 | 0.528778 | 1.872241 |
| LOC382758 | 0.047364 | 0.528923 | 0.902334 |
| PEX11B | 0.047416 | 0.529183 | 0.659723 |
| TNFAIP2 | 0.047438 | 0.529183 | 1.479353 |
| FBXL17 | 0.047442 | 0.529183 | 1.074998 |
| HIBADH | 0.047456 | 0.529183 | 0.525829 |
| CDT1 | 0.047487 | 0.529221 | 0.299861 |
| DUS1L | 0.047508 | 0.529221 | 0.703538 |
| MCMDC1 | 0.047512 | 0.529221 | 1.127479 |
| AU022252 | 0.047543 | 0.529221 | 1.443896 |
| PAOX | 0.047555 | 0.529221 | 1.401527 |
| ECAC2 | 0.047572 | 0.529221 | 1.12127 |
| LOC546090 | 0.047615 | 0.529221 | 0.667266 |
| METTL3 | 0.047639 | 0.529221 | 0.632206 |
| 2610024H22RIK | 0.047641 | 0.529221 | 1.315247 |
| CMTM6 | 0.047644 | 0.529221 | 1.530426 |
| CDCA2 | 0.047659 | 0.529221 | 0.196305 |
| PHGDH | 0.047665 | 0.529221 | 0.330755 |
| MSI1H | 0.047711 | 0.529384 | 0.87194 |
| UGCG | 0.047735 | 0.529384 | 2.056228 |
| AI256775 | 0.047741 | 0.529384 | 1.340589 |
| AP1S1 | 0.047748 | 0.529384 | 1.308669 |
| 4921507P07RIK | 0.047833 | 0.530133 | 0.72892 |
| A930009N24 | 0.047906 | 0.530748 | 1.258234 |
| LIPH | 0.047935 | 0.530882 | 1.079478 |
| ORF61 | 0.047981 | 0.530914 | 1.309455 |
| 1700063I17RIK | 0.047994 | 0.530914 | 0.799905 |
| 9790357_3511_RC | 0.048003 | 0.530914 | 0.942632 |
| LOC382010 | 0.048021 | 0.530914 | 0.528387 |
| BC051665 | 0.048036 | 0.530914 | 0.937376 |
| ANXA4 | 0.048099 | 0.530914 | 1.293757 |
| 1300006C19RIK | 0.048118 | 0.530914 | 0.818299 |
| TAF1 | 0.048122 | 0.530914 | 0.846902 |
| PDIK1L | 0.04814 | 0.530914 | 0.870812 |
| C030003H22RIK | 0.048186 | 0.530914 | 1.509252 |
| SCL0002791.1_134 | 0.048189 | 0.530914 | 0.615885 |
| MAFB | 0.04821 | 0.530914 | 0.86308 |
| CAPPA2 | 0.048249 | 0.530914 | 1.098677 |
| MYEF2 | 0.048315 | 0.530914 | 0.605962 |
| HOXA13 | 0.048331 | 0.530914 | 1.100251 |
| CCDC83 | 0.048342 | 0.530914 | 0.921187 |
| MCART1 | 0.048373 | 0.530914 | 0.827444 |
| FAIM2 | 0.048406 | 0.530914 | 1.201275 |
| NCSTN | 0.048449 | 0.530914 | 1.357953 |
| LOC100048504 | 0.048462 | 0.530914 | 1.482844 |
| MBTPS2 | 0.048469 | 0.530914 | 0.739523 |
| DARS | 0.048483 | 0.530914 | 0.769326 |
| D430014P18RIK | 0.048489 | 0.530914 | 0.933119 |
| ACYP2 | 0.048495 | 0.530914 | 1.222584 |
| RNASEH2C | 0.048498 | 0.530914 | 0.730235 |
| COMT | 0.04851 | 0.530914 | 1.054847 |
| 3830430K15RIK | 0.048512 | 0.530914 | 0.501736 |
| 4930557F10RIK | 0.048521 | 0.530914 | 1.069745 |
| LATS1 | 0.04853 | 0.530914 | 0.871235 |
| TMEM147 | 0.048535 | 0.530914 | 0.653383 |
| AK3 | 0.048542 | 0.530914 | 0.753458 |
| UNC50 | 0.048568 | 0.530914 | 1.058876 |
| EGLN2 | 0.048571 | 0.530914 | 1.287197 |
| WDR4 | 0.048592 | 0.530914 | 0.805394 |
| MSR2 | 0.048616 | 0.530914 | 1.074948 |
| C130071N01RIK | 0.048621 | 0.530914 | 1.127374 |
| 4933413G19RIK | 0.04863 | 0.530914 | 1.0345 |
| FAH | 0.048662 | 0.530914 | 1.504586 |
| NARF | 0.048668 | 0.530914 | 1.509251 |
| EG635919 | 0.048675 | 0.530914 | 1.101244 |
| 4831422N07RIK | 0.048679 | 0.530914 | 1.183615 |
| ERAF | 0.048681 | 0.530914 | 1.144751 |
| OLFR1209 | 0.0487 | 0.530914 | 1.058778 |
| 6430531B16RIK | 0.048711 | 0.530914 | 1.124643 |
| EFNA4 | 0.048712 | 0.530914 | 1.986093 |
| C030003D03RIK | 0.048741 | 0.531013 | 1.28949 |
| LOC381195 | 0.048756 | 0.531013 | 0.89608 |
| D030049N18RIK | 0.048815 | 0.531207 | 0.85805 |
| NEDD1 | 0.048818 | 0.531207 | 0.750297 |
| D15BWG0759E | 0.048838 | 0.531207 | 1.437571 |
| 2510040D07RIK | 0.048861 | 0.531207 | 0.667358 |
| RNPEPL1 | 0.048872 | 0.531207 | 1.510822 |
| RINT1 | 0.048885 | 0.531207 | 0.945031 |
| SYCP2 | 0.048894 | 0.531207 | 0.881847 |
| PROM1 | 0.048921 | 0.531314 | 1.076464 |
| CENPA | 0.048989 | 0.53158 | 0.284927 |
| LOC232745 | 0.048995 | 0.53158 | 0.592834 |
| D430039N05RIK | 0.048997 | 0.53158 | 0.934781 |
| LOC242987 | 0.049025 | 0.531689 | 0.91921 |
| 4930488B01RIK | 0.049065 | 0.531936 | 1.045119 |
| CEP78 | 0.049126 | 0.532273 | 0.613783 |
| PCDHB2 | 0.049152 | 0.532273 | 1.070017 |
| HPRT1 | 0.049158 | 0.532273 | 0.64806 |
| TRAF5 | 0.049182 | 0.532273 | 1.230291 |
| ABCB7 | 0.049182 | 0.532273 | 0.469501 |
| EIF3S4 | 0.049203 | 0.532296 | 0.82169 |
| LRRC48 | 0.049232 | 0.532296 | 0.665803 |
| RBM13 | 0.049236 | 0.532296 | 0.894756 |
| EG627022 | 0.049322 | 0.533045 | 0.520824 |
| PDGFRB | 0.049346 | 0.533116 | 1.918218 |
| ELP2 | 0.049391 | 0.533363 | 0.755289 |
| IFT140 | 0.04943 | 0.533363 | 1.076937 |
| B930030P05RIK | 0.049433 | 0.533363 | 0.89232 |
| RAB22A | 0.049492 | 0.533363 | 1.513442 |
| 3000004C01RIK | 0.049501 | 0.533363 | 0.283522 |
| GPX1 | 0.04952 | 0.533363 | 1.246025 |
| RAB40C | 0.049545 | 0.533363 | 0.867739 |
| IL3RA | 0.049546 | 0.533363 | 1.249196 |
| MRPL22 | 0.049553 | 0.533363 | 0.66705 |
| GFM1 | 0.049558 | 0.533363 | 0.756826 |
| ARFRP1 | 0.049559 | 0.533363 | 0.62493 |
| LOC100039649 | 0.0496 | 0.533614 | 0.906786 |
| 1810012P15RIK | 0.049652 | 0.533766 | 0.884602 |
| ATP5F1 | 0.049669 | 0.533766 | 0.668639 |
| D630028G08RIK | 0.049677 | 0.533766 | 1.139763 |
| D630033O11RIK | 0.049685 | 0.533766 | 0.862442 |
| EG622320 | 0.049707 | 0.533766 | 1.427938 |
| NUP133 | 0.049718 | 0.533766 | 1.072293 |
| E330021D16RIK | 0.049778 | 0.534126 | 0.691771 |
| INCENP | 0.049786 | 0.534126 | 0.350026 |
| FLT1 | 0.049823 | 0.534251 | 1.859738 |
| 2610010A15RIK | 0.049835 | 0.534251 | 0.73086 |
| LAD1 | 0.04985 | 0.534251 | 1.169047 |
| IGF2 | 0.049867 | 0.534251 | 2.166951 |
| DEFB30 | 0.049911 | 0.534535 | 1.123318 |
| ACTN4 | 0.049953 | 0.534665 | 1.560491 |
| AI553587 | 0.049971 | 0.534665 | 0.909219 |
| IFT74 | 0.049986 | 0.534665 | 0.801977 |
| IGHA_J00475$V00785_IG_HEAVY_CONSTANT_ALPHA_135 | 0.049992 | 0.534665 | 1.093737 |
