# Supplementary material for: Anti-miR-17 therapy delays tumorigenesis in MYC-driven hepatocellular carcinoma (HCC)
Source: Oncotarget. 2017 Nov 9;9(5):5517–28. doi: 10.18632/oncotarget.22342 (PMC5814155; doi:10.18632/oncotarget.22342)
Supplement: Supplementary file 5 [file oncotarget-09-5517-s005.docx]

**Supplementary Table 4: Genes that are targets of miR-17 and are differentially expressed both upon anti miR-17 treatment and upon MYC inactivation**

| **Symbol** | **Entrez Gene Name** |
| --- | --- |
| ARHGEF18 | Rho/Rac guanine nucleotide exchange factor 18 |
| ARHGEF3 | Rho guanine nucleotide exchange factor 3 |
| ATXN1 | ataxin 1 |
| ATXN1L | ataxin 1 like |
| BTBD10 | BTB domain containing 10 |
| CC2D1A | coiled-coil and C2 domain containing 1A |
| E2F5 | E2F transcription factor 5 |
| EGLN3 | egl-9 family hypoxia inducible factor 3 |
| FANCI | Fanconi anemia complementation group I |
| FRMD4A | FERM domain containing 4A |
| FYCO1 | FYVE and coiled-coil domain containing 1 |
| ITPRIPL2 | inositol 1,4,5-trisphosphate receptor interacting protein like 2 |
| MASTL | microtubule associated serine/threonine kinase like |
| NAGK | N-acetylglucosamine kinase |
| PKD1 | polycystin 1, transient receptor potential channel interacting |
| PLEKHA3 | pleckstrin homology domain containing A3 |
| POLR3G | RNA polymerase III subunit G |
| PTPN21 | protein tyrosine phosphatase, non-receptor type 21 |
| RAB11FIP5 | RAB11 family interacting protein 5 |
| RAB22A | RAB22A, member RAS oncogene family |
| SLC36A1 | solute carrier family 36 member 1 |
| STAT3 | signal transducer and activator of transcription 3 |
| Susd6 | sushi domain containing 6 |
| SYAP1 | synapse associated protein 1 |
| TRIM3 | tripartite motif containing 3 |
| TRIM37 | tripartite motif containing 37 |
| TRIP10 | thyroid hormone receptor interactor 10 |
| TSC22D2 | TSC22 domain family member 2 |
| VEGFA | vascular endothelial growth factor A |
| VLDLR | very low density lipoprotein receptor |
| ZFP367 | zinc finger protein 36 |
| ZHX2 | zinc fingers and homeoboxes 2 |
| ZNFX1 | zinc finger NFX1-type containing 1 |
